# Generation of RNA aptamers against chikungunya virus E2 envelope protein

**DOI:** 10.1128/jvi.02095-24

**Published:** 2025-02-10

**Authors:** Kaku Goto, Ryo Amano, Akiko Ichinose, Akiya Michishita, Michiaki Hamada, Yoshikazu Nakamura, Masaki Takahashi

**Affiliations:** 1 Project Division of RNA Medical Science, The Institute of Medical Science, The University of Tokyo 26430 , Tokyo, Japan; 2 Graduate School of Advanced Science and Engineering, Waseda University 214339 , Tokyo, Japan; 3 RIBOMIC Inc., Tokyo, Japan; University of North Carolina at Chapel Hill, Chapel Hill, North Carolina, USA

**Keywords:** aptamer, CHIKV, neutralization

## Abstract

Nucleic acid aptamers are a promising drug modality, whereas the generation of virus-neutralizing aptamers has remained difficult due to the lack of a robust system for targeting the viral particles of interest. Here, we took advantage of our latest platform technology of Systematic Evolution of Ligands by EXponential enrichment (SELEX) with virus-like particles (VLPs) and targeted chikungunya virus (CHIKV) as a model, the pathogenic reemerging virus with an unmet need for control. The identified aptamer against CHIKV-VLPs, Apt#1, and its truncated derivatives showed neutralizing activity with nanomolar IC50 values in a cell-based assay system using a pseudoviral particle of CHIKV (CHIKVpp). An antiviral-based chemical genetics approach revealed significant competition of Apt#1 with suramin, a reported interactant with domain A of the E2 envelope protein (E2DA), in both CHIKVpp and surface plasmon resonance (SPR) analyses, predicting E2DA to be the Apt#1 interface. In addition, Apt#1 interfered with the attachment of CHIKVpp, collectively suggesting its property as an attachment inhibitor via E2DA of CHIKV. Thus, the generation of the VLP-targeted aptamers proved to contribute to anti-CHIKV strategies and confirmed the utility of the platform as a novel and viable option for the development of neutralizing agents against viral particles of interest.

**IMPORTANCE:**

Our latest SELEX technology using VLPs has generated aptamers that bind the native conformation of the incorporated envelope protein and achieve the virus binding and neutralizing effects. Indeed, the aptamer-probed target E2DA is a representative neutralization site on the surface of the viral particle, validating the utility of the VLP-driven procedure. Simultaneously, the enhanced antiviral effects of the aptamer in combination with approved drugs using the CHIKVpp assay with human cells indicated potential therapeutic strategies that are expected to help address unmet needs in CHIKV control. The robust affinity of the aptamer to viral particles demonstrated by SPR analysis can also lead to conjugates with antivirals as guiding molecules and aptasensors for diagnostic tools. Overall, our VLP-based method provided anti-CHIKV as well as a versatile platform applicable to other emerging and reemerging viruses, in preparation for outbreaks with the need for rapid development of antiviral strategies as next-generation theranostics.

## INTRODUCTION

Nucleic acid aptamers are single-stranded oligonucleotides with diverse conformations that bind to targets of interest with relatively high shape complementarity (1). They are considered a promising drug modality because of favorable features compared to antibodies, including chemical synthesis, low antigenicity, and the availability of computational science for screening (2–4). Since the first approval of an aptamer as an anti-vascular endothelial growth factor agent for age-related macular disease in 2004 (pegaptanib; Macgen), a number of aptamers have been developed and are being evaluated in preclinical studies and clinical trials (5). For aptamer development, candidate sequences are identified from a combinatorial library consisting of approximately one quadrillion molecules using the Systematic Evolution of Ligands by EXponential Enrichment (SELEX) technology (6, 7). Although the conceptual method was proposed in 1990, a robust selection system applicable even to unstable cell-surface proteins has not been established due to the difficulty of preparing suitable selection materials with the intact conformation of the target membrane proteins (8). To address this issue, we have recently established and reported an alternative method to generate aptamers exemplarily targeting G-protein-coupled receptors using virus-like particles (VLPs) as structurally stabilizing materials for membrane proteins to maintain their physiological structures (9, 10). Indeed, the VLP-based approach has successfully discovered ligands for human cell surface proteins and naturally raised viral membrane proteins in VLPs as more feasible and in-demand targets. Until now, aptamers against viruses such as human immunodeficiency virus (HIV) and severe acute respiratory syndrome coronavirus 2 (SARS-CoV-2) have been generated using recombinant proteins (11, 12). Their neutralizing activities against authentic viruses led to the recognition of the neutralizing capabilities of aptamers. It is, therefore, important to facilitate the generation of antiviral aptamers by all possible methods, including the VLP-based way, leading to neutralizing molecules against various viruses.

Chikungunya virus (CHIKV) is a mosquito-borne alphavirus that causes various symptoms in humans, including fever, rash, severe arthritis lasting for months to years, and even fatal encephalitis (13). To date, CHIKV outbreaks have mainly occurred in tropical regions such as India, Africa, and South and Southeast Asia (14). During the 2004 outbreak, there were an estimated 6 million cases, and the virus was circulating in almost 40 countries (15–18). Recently, CHIKV disease has spread to Europe and the Americas and is no longer confined to tropical areas, perhaps due to global warming, which has led to an expansion of mosquito habitats (15, 16, 18, 19). Although more than 70 years have passed since CHIKV was first identified in Tanzania in 1952 (20), there is still no approved therapeutic vaccine or medicine for CHIKV disease, with the exception of the recent preventive vaccine Ixchiq (21). In the field of CHIKV-targeted aptamers, several molecules have been isolated, but their use has been limited to diagnosis, without therapeutic elucidation in cell culture or *in vivo* (22). Therefore, it is imperative to gain more insights into the mechanism of infection and accelerate drug discovery using various drug modalities to achieve treatment of CHIKV disease.

In this study, to evaluate the feasibility of the VLP-based platform for the generation of virus-neutralizing aptamers, we selected CHIKV as a model target and used CHIKV-VLPs for screening. We also implemented the CHIKV pseudoparticle (CHIKVpp) assay and a chemical genetics approach to investigate the neutralizing activity of the aptamers.

## RESULTS

### SELEX targeting CHIKV-VLPs

**Fig 1:**
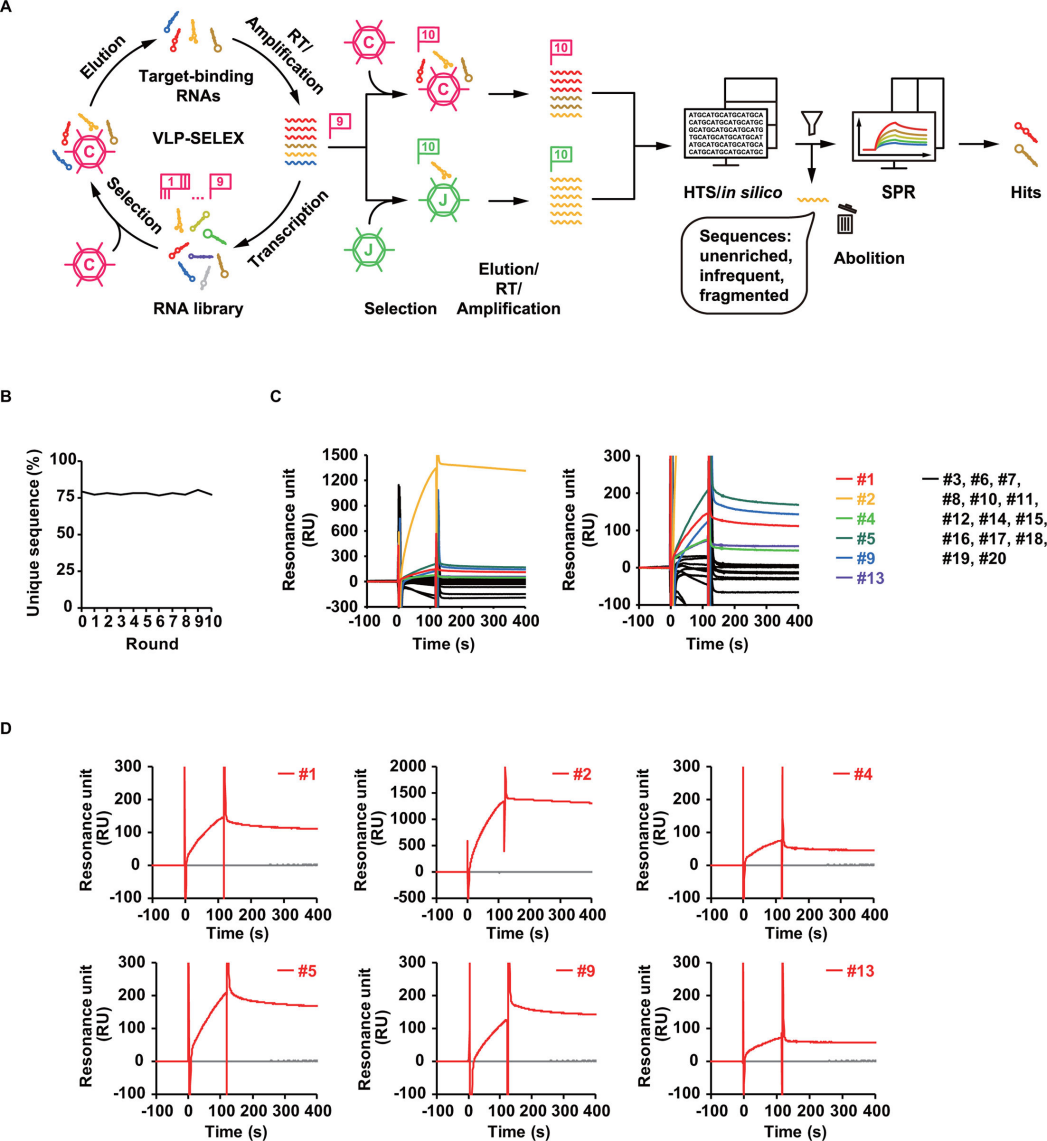
SELEX against CHIKV-VLPs. (**A**) Schema of the entire aptamer screening process. Selection was performed with CHIKV-VLPs for up to nine rounds, and the library was mixed with the target CHIKV-VLPs and nontarget JEV-VLPs in the final 10th round to predict specific and nonspecific sequences bound to the target. The sequences in the final round were examined by HTS and *in silico* with FASTAptamer, comparing those selected with each type of VLP, and the hit sequences bound to the target were identified by SPR analysis. (**B**) The number of unique sequences as a criterion for enrichment in each round of SELEX was examined using RaptRanker. (**C**) An integrated sensorgram of the 20 most frequent clones binding to CHIKV-VLPs, with local magnification on the right. (**D**) Binding assay of aptamers by SPR analysis. The affinity of isolated aptamers to CHIKV-VLPs was investigated by SPR analysis with or without aptamers Apt#1, #2, #4, #5, #9, and #13 immobilized on sensor chips shown in red and gray, respectively.

In accordance with our previous report (9), CHIKV-VLPs were subjected to a total of 10 rounds of SELEX (Fig. 1A; Table 1). In the final round, the resulting library was mixed with the target CHIKV-VLPs or non-target Japanese encephalitis virus VLPs (JEV-VLPs), and the sequences were examined by high-throughput sequencing (HTS). The data obtained were analyzed using RaptRanker and FASTAptamer (Fig. 1B; Table S1) (3, 23). Due to the inadequacy of pronounced library enrichment and characteristic motifs, the top 20 sequences with the highest number of reads per million (PRM) in each of the 20 individual clusters defined by more than five base differences, after elimination of aberrant short sequences, were examined by surface plasmon resonance (SPR) analysis (Fig. 1C). Among 20 candidates Apt#1 to #20, six sequences Apt#1, #2, #4, #5, #9, and #13 showed binding ability to CHIKV-VLPs in SPR analysis using an aptamer-immobilized sensor chip (Fig. 1D) and were selected for further evaluation.

**TABLE 1:** SELEX condition^*a*^

Round	VLP (μg)	ssRNA (μg)	PS (min)^*b*^	CS^*c*^	Wash (times)
1	1	10	60	-	5
2	1	10	30	+	8
3	0.5	10	30	+	8
4	0.25	10	20	+	8
5	0.125	10	20	+	10
6	0.125	10	10	+	10^*d*^
7	0.125	10	10	+	10
8	0.125	5	10	+	10
9	0.125	2.5	10	+	10
10	0.125	1.25	10	+	10

^*a*^ PS, positive selection; CS, counter-selection.

^*b*^ Positive selection with CHIKV-VLPs for 10 to 60 minutes, as indicated.

^*c*^ Counter-selection using the ultrafiltration column, as explained in Materials and Methods. +, performed ; -, not performed.

^*d*^ Gel collection.

### Evaluation of the neutralizing activity of isolated aptamers using CHIKVpp

**Fig 2:**
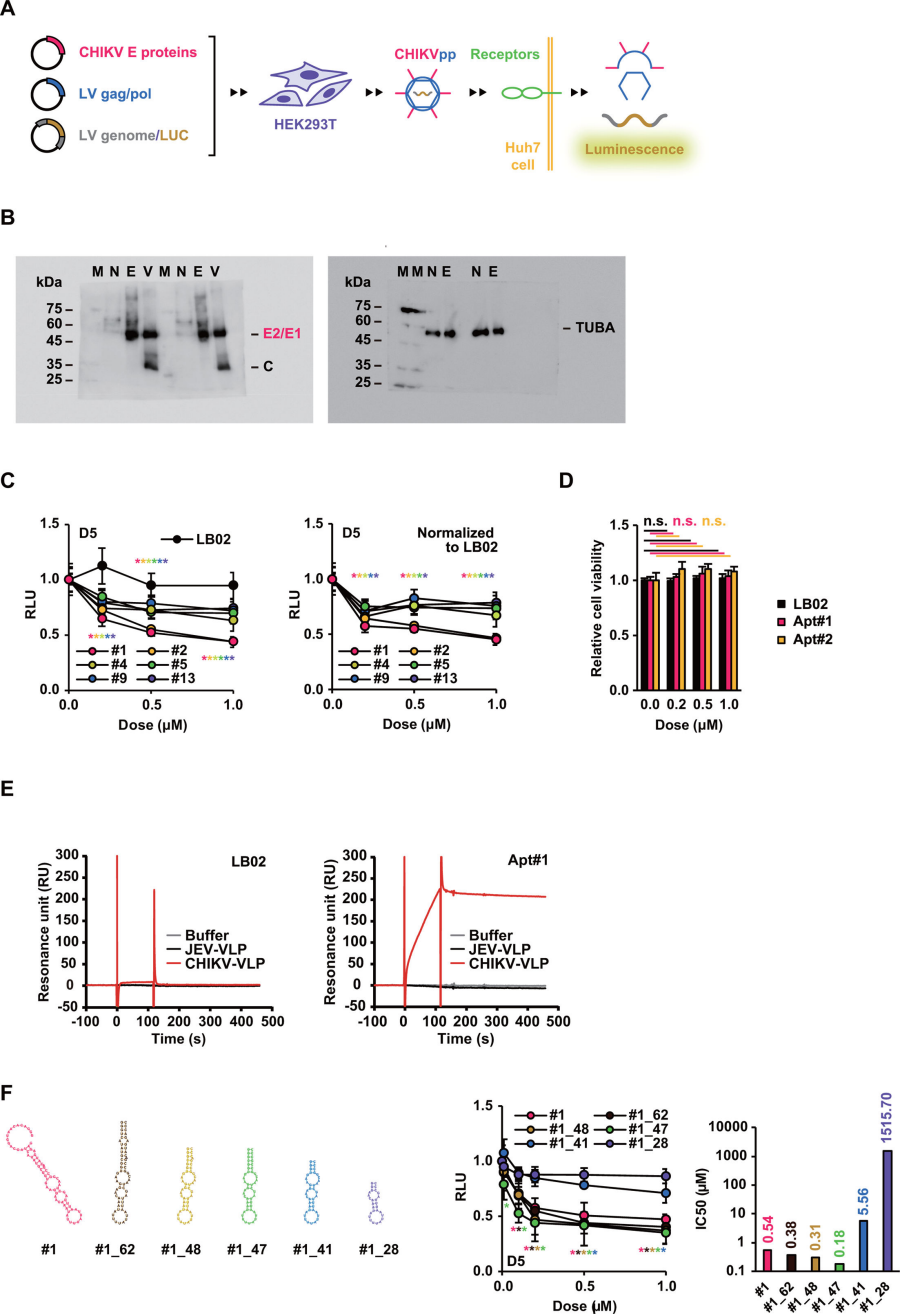
Inhibitory effects of aptamers on CHIKVpp entry. (**A**) Production of CHIKVpp. CHIKVpp was produced from HEK293T cells transfected with the plasmids expressing CHIKV E proteins, lentiviral packaging proteins, and a lentiviral luciferase reporter gene. Huh7 cells were transduced with CHIKVpp in the presence or absence of aptamers, and the luciferase activity was measured at 5 days post-transduction. (**B**) CHIKV E2 and E1 proteins in pEBMN-CHIKV-E-transfected HEK293T cells and VLPs were detected by Western blot using rabbit anti-chikungunya 181/25 polyclonal antibody (left), and TUBA expression was similarly detected as an internal control (right). The banding patterns of the E2/E1, C, and TUBA proteins are illustrated on the right. The identical set of samples was detected twice with the size markers at the indicated molecular weights, and the lanes M, N, E, and V denote marker, nontransfected cells, pEBMN-CHIKV-E-transfected cells, and VLPs, respectively. (**C**) The dose responses of the six CHIKV-VLP-binding aptamers and a random library LB02 were investigated in the CHIKVpp entry assay at 5 days post-transduction (D5) as shown in panel A (left). The relative light unit (RLU) was calculated by normalization to cell viability and comparison with no treatment. The individual values were then normalized to LB02 (right). Data are presented as mean ± SD (*n* = 3 to 4). (**D**) Relative cell viability was measured in the presence of LB02, Apt#1, and Apt#2 as in panel C compared to no treatment. Data are presented as mean ± SD (*n* = 4). (**E**) The binding of a random library LB02 (left) or Apt#1 (right) to CHIKV-VLPs or JEV-VLPs was evaluated by SPR analysis. (**F**) Truncated forms of Apt#1 were generated (left), and the inhibitory dose response as in panel C (middle) and IC50 values (right) were determined. Data are presented as mean ± SD (*n* = 3 to 4). Statistical differences among treatment groups were examined by two-way analysis of variance (ANOVA) and then by Tukey–Kramer test. ^*^ *P*<0.05 for effects of color-indicated aptamers compared to controls.

To investigate the neutralizing activity of the aptamers against CHIKV, we established a cell assay system using CHIKVpp with the envelope proteins of the West African strain 37997 expressing the *firefly luciferase* gene to estimate CHIKV entry into a virus-permissive hepatocellular carcinoma (HCC) cell line Huh7 cells (Fig. 2A and B). Among the aptamers tested, Apt#1 and Apt#2 blocked CHIKVpp in dose-dependent manners with 50% inhibitory concentration (IC50) values of 539 nM and 964 nM, respectively, while a random library LB02 as a negative control did not (Fig. 2C). Concomitantly, dose dependencies and statistically significant effects of Apt#1 and Apt#2 were also shown after normalization to LB02 (Fig. 2C), and none of Apt#1, Apt#2, or LB02 affected cell viability at the indicated doses with statistical significance (Fig. 2D), thus confirming the validity of the evaluation. In addition, SPR analysis revealed no affinity of a random library for CHIKV-VLPs and Apt#1 for JEV-VLPs, suggesting that Apt#1 interacts specifically with CHIKV-VLPs/CHIKVpp independently of the physical properties of the nucleic acids (Fig. 2E). Subsequently, Apt#1, with the best IC50 value, was optimized by truncation (Fig. 2F; Table 2). A truncated sequence of 47 nucleotides (nt) in length, Apt#1_47, exhibited the highest activity of the sequences examined, with an IC50 value of 180 nM, exceeding that of the parent full-length aptamer, Apt#1. Therefore, Apt#1 and its truncated form Apt#1_47 were subjected to detailed functional analysis.

**TABLE 2:** Sequences of truncated aptamer Apt#1

Name	Sequence (5'-sequence-3')
#1	GGGACACAATGGACGATCATATGCGGTGAAGGAACAAATATTTTATTAATTTAGCTAACGGCCGACATGAGAG
#1_62	GGGTCATATGCGGTGAAGGAACAAATATTTTATTAATTTAGCTAACGGCCGACATGAGACCC
#1_48	GGGCGGTGAAGGAACAAATATTTTATTAATTTAGCTAACGGCCGACCC
#1_47	GGGCGGTGAAGGAACAAATATTTTATTAATTTAGCTAACGGCCGCCC
#1_41	GGGTGAAGGAACAAATATTTTATTAATTTAGCTAACGGCCC
#1_28	GGGAACAAATATTTTATTAATTTAGCCC

### Characterization of aptamer properties by antiviral-based chemical genetic analysis

**Fig 3:**
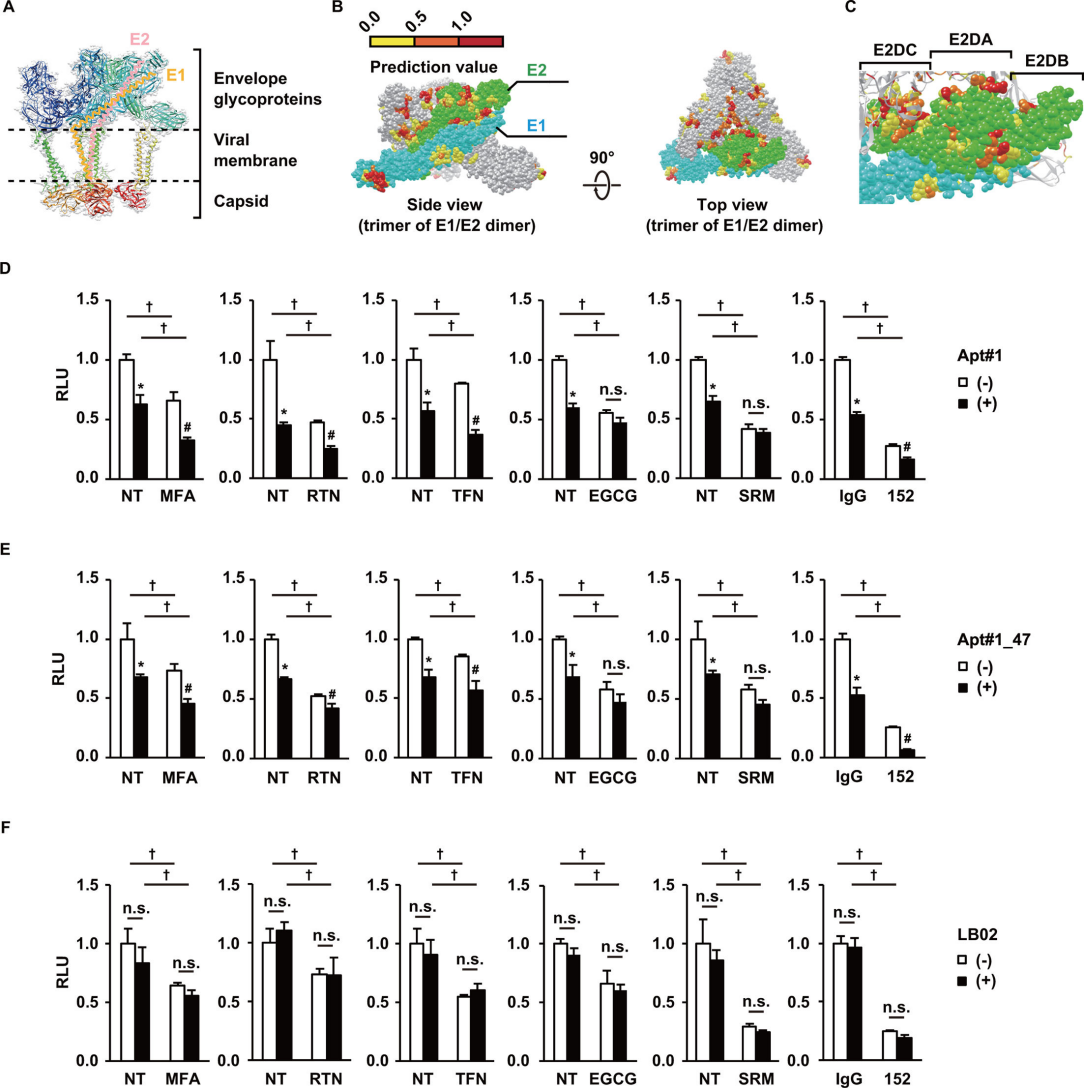
Characterization of aptamers using an antiviral-based chemical genetics approach. (**A**) Structure of a spike unit of CHIKV, based on PDB entry 3J2W. The approximate orientation of the E1 and E2 proteins is indicated in orange and pink, respectively. For clarity, reference can be made to the model in Fig. 5G. (**B**) Predicted RNA interface on a CHIKV spike unit. Side (left) and top (right) views of the trimer of the E1/E2 heterodimers are shown, with the first E1 and E2 proteins from the front indicated in cyan and green, respectively. As described in Materials and Methods, amino acids predicted by the KYG program (http://cib.cf.ocha.ac.jp/KYG/index.php) to be the RNA interface are shown as colored spheres in yellow, orange, or red over the trimer structure according to their prediction values greater than 0. (**C**) Enlarged view of the E2 protein showing domains A (E2DA), B (E2DB), and C (E2DC). (**D**) Effects of co-treatment with antivirals and Apt#1. Briefly, Huh7 cells were transduced with CHIKVpp preincubated with Apt#1 at 1 µM in the presence of MFA at 100 µM, RTN at 1000 µM, TFN at 400 µM, EGCG at 100 µM, SRM at 50 µM, or IgG or CHK152 monoclonal antibody (mAb) at 10 ng/µL (67 nM), and the medium was changed 6 hours after transduction, followed by harvesting 3 days post-transduction. The RLUs were determined as in Fig. 2C. Data are presented as mean ± SD (*n* = 3 to 4). (**E**) The effects of co-treatment with antivirals and Apt#1_47 were evaluated as in panel D. Data are presented as mean ± SD (*n* = 3 to 5). (**F**) The effects of co-treatment with antivirals and LB02 were evaluated, as in panel D. Data are presented as mean ± SD (*n* = 3 to 5). Statistical differences among treatment groups were examined by two-way ANOVA and then by Tukey–Kramer test. ^*^ *P*<0.05 for aptamer effects in the absence of antivirals, ^#^ *P* < 0.05 for aptamer effects in the presence of antivirals, ^†^ *P* < 0.05 for antiviral effects in the presence or absence of aptamers, and n.s., no significant difference between the indicated groups.

To elucidate the antiviral mechanism of the aptamers in a chemical–genetic way, CHIKVpp was treated with Apt#1 or Apt#1_47 in combination with several antivirals known to target CHIKV envelope proteins, and the aptamer-binding domains were evaluated based on the competitiveness of aptamers and antivirals. Initially, the E2 protein was analyzed *in silico* for potential target sites in accordance with its exposure on the surface of the virus particle. However, no RNA interface residues were predicted because the charge and hydrophobicity were not favorable for nucleic acid aptamer binding (Fig. 3A through C). Then, in lieu of inhibitors directed to specific spots, a range of antivirals with diverse target sites for the E2 and/or E1 proteins were exploited (Table 3). The antiviral effects of Apt#1 (Fig. 3D) or Apt#1_47 (Fig. 3E) were maintained in combination with mefenamic acid (MFA), rutin (RTN), taxifolin (TFN), or the representative neutralizing antibody CHK152 (24). However, suramin (SRM), suggested to interact with the E2 Domain A (E2DA) (25), and epigallocatechin gallate (EGCG), impeding E2-mediated attachment, interfered with the aptamers (Fig. 3D and E), suggesting that the aptamers, SRM, and EGCG competitively target the similar epitopes on CHIKV. On the other hand, a random library did not thwart the antivirals against CHIKVpp entry (Fig. 3F).

**TABLE 3:** Reported anti-CHIKV and therapeutic properties of the antivirals used in this study

Name	Therapeutic property	Antiviral targets	Reference
Mefenamic acid (MFA)	Approved, e.g., rheumatoid arthritis	Envelope proteins at entry	(26)
Rutin (RTN)	Dietary supplement; Phase I, e.g., COVID-19	E1DII and E2DA at fusion	(27)
Taxifolin (TFN)	Dietary supplement; Phase study, e.g., COVID-19	E1DII/Fusion loop E2DA/DB at fusion	(27)
Epigallocatechin gallate (EGCG)	Dietary supplement; Phase III, e.g., COVID-19	E2 at attachment	(28)
Suramin (SRM)	Approved, e.g., trypanosome-caused river blindness	E2DA at attachment	(25)
CHK152 antibody	Anti-CHIKV activity *in vivo*	E2DA/DB at fusion	(24)
Chlorpromazine (CPH)	Approved: psychotic disorders	Clathrin-mediated endocytosis at entry	(29)
Imipramine (IPH)	Approved, e.g., depression	Cholesterol trafficking at entry	(30)

### Biophysical elucidation of aptamer binding

**Fig 4:**
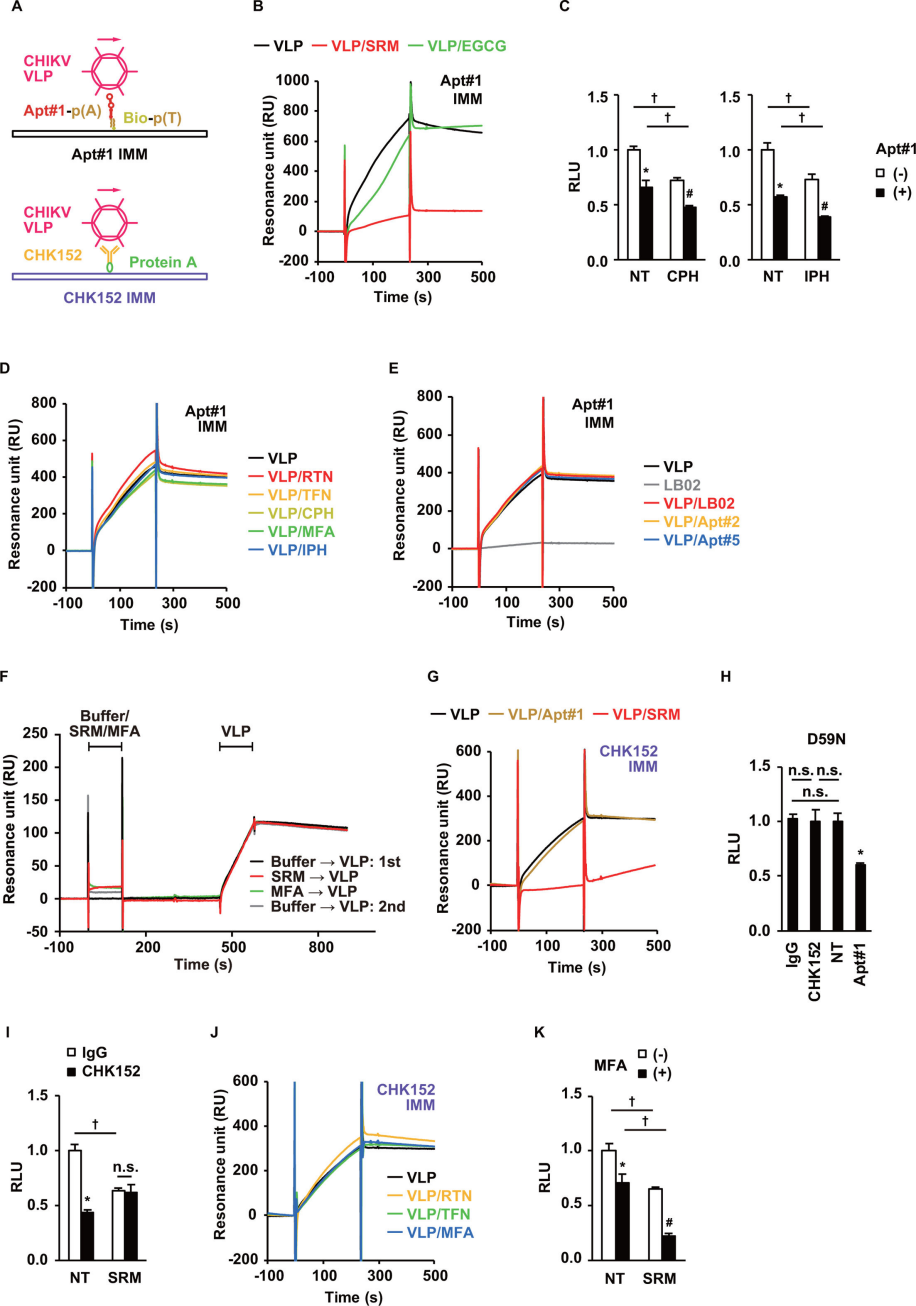
Biophysicochemical elucidation of aptamer functions. (**A**) Scheme of SPR analysis. In the upper panel, Apt#1 has been immobilized on a streptavidin sensor chip (Apt#1 IMM). In the lower panel, CHK152 antibodies have been immobilized on a CM5 sensor chip (CHK152 IMM). Details are described in the *Methods* section. (**B**) Effect of preincubation with SRM and EGCG on the interaction of Apt#1 with CHIKV-VLPs. The mixture of CHIKV-VLPs with either SRM or EGCG was assessed as an analyte on the Apt#1 IMM sensor chip. (**C**) Effects of cotreatment with CPH at 40 µM or IPH at 100 µM and Apt#1 at 1 µM as in Fig. 3D. Data are presented as mean ± SD (*n* = 3 to 4). ^*^ *P* < 0.05 for effects of Apt#1 in the absence of CPH or IPH. ^#^ *P* < 0.05 for effects of Apt#1 in the presence of CPH or IPH. ^†^ *P* < 0.05 for effects of CPH or IPH in the presence or absence Apt#1. (**D**) The effects of preincubation with RTN, TFN, CPH, MFA, and IPH on the interaction of Apt#1 with CHIKV-VLPs were assessed, as in panel B. (**E**) Sensorgrams of Apt#1-immobilized sensor chips with a mixture of CHIKV-VLPs and either RNA pool from LB02, Apt#2, or Apt#5 as analytes are shown as in panel B. Sensorgrams of Apt#1-immobilized sensor chips with only CHIKV-VLPs or LB02 RNA pool as analytes are shown in black and gray, respectively. (**F**) The Apt#1-immobilized sensor chip was pretreated by injection of SELEX buffer, SRM, or MFA and then treated with the running buffer, followed by the injection of CHIKV-VLPs to evaluate the binding ability of Apt#1 to CHIKV-VLPs. The injection times of buffer, SRM, or MFA and CHIKV-VLPs are shown in the graph. Evaluation of CHIKV-VLPs with SELEX buffer pretreatment as negative controls was performed before (first) and after (second) evaluation of CHIKV-VLPs with SRM or MFA pretreatment. (**G**) Effect of preincubation of Apt#1 and SRM on the interaction of CHK152 with CHIKV-VLPs. The mixture of CHIKV-VLPs with either SRM or Apt#1 was assessed as an analyte on the CHK152 IMM sensor chip. (**H**) The effects of IgG or CHK152 at 10 ng/µL and Apt#1 at 1 µM were investigated in the entry assay of CHIKVpp harboring the E2 D59N mutation, as in Fig. 2A. Data are presented as mean ± SD (*n* = 4). (**I**) The effect of preincubation of SRM on CHK152 antibody-mediated inhibition of CHIKVpp entry was examined, as shown in Fig. 3D. The final concentrations of SRM and both IgG and CHK152 are 20 µM and 10 ng/µL, respectively. Data are presented as mean ± SD (*n* = 4). ^*^ *P* < 0.05 for CHK152 effects in the absence of SRM, ^†^ *P* < 0.05 for SRM effects in the absence of CHK152, and n.s., no significant difference between the indicated groups. (**J**) The effects of preincubation with RTN, TFN, and MFA on the interaction of CHK152 with CHIKV-VLPs were assessed, as in panel G. (**K**) The effect of SRM in the absence or presence of MFA, denoted as MFA (-) or MFA (+), respectively, on CHIKVpp entry was investigated, as shown in Fig. 3D. The final concentrations of SRM and MFA are 20 µM and 100 µM, respectively. Data are presented as mean ± SD (*n* = 3 to 4). ^*^ *P* < 0.05 for effects of MFA in the absence of SRM. ^#^ *P* < 0.05 for effects of MFA in the presence of SRM. ^†^ *P* < 0.05 for effects of MFA in the presence or absence SRM. Statistical differences among treatment groups were examined by two-way ANOVA and then by Tukey–Kramer test.

To decipher the interaction of the aptamers with CHIKV at the biophysical level, the competitiveness of Apt#1 with SRM, EGCG, and other antivirals was examined by SPR analysis. Using an Apt#1-immobilized sensor chip (Fig. 4A), the pretreatment of CHIKV-VLPs with SRM significantly inhibited the interaction of Apt#1 with CHIKV-VLPs (Fig. 4B); this was not the case with the other compounds including EGCG, MFA, RTN, TFN, and the host cell-targeted viral entry inhibitors chlorpromazine (CPH) and imipramine (IPH) inhibiting clathrin-dependent endocytosis (31, 32) and endolysosome (33, 34), respectively (Table 3), without competition with Apt#1 (Fig. 4C), and a random library as a negative control (Fig. 4B, D and E). A marginal interference with the association phase by EGCG was observed (Fig. 4B), but at least clearly distinguishable from the marked competition by SRM. Concurrently, pretreatment of immobilized Apt#1 itself with SRM did not affect the affinity of Apt#1 for CHIKV-VLPs (Fig. 4F), confirming the interference of SRM with Apt#1 activity via CHIKV-VLPs. Thus, the SPR analysis data suggested that Apt#1 and SRM evidently compete for binding to CHIKV, consistent with the observation on CHIKVpp (Fig. 3D).

In contrast, SPR analysis using a CHK152 antibody-immobilized sensor chip (Fig. 4A) showed no competition between Apt#1 and CHK152 antibody (Fig. 4G). Furthermore, Apt#1 inhibited CHIKVpp carrying a CHK152-resistant mutation D59N in E2DA (35) (Fig. 4H), suggesting that Apt#1 targets CHIKV differently from the CHK152 antibody. On the other hand, the pretreatment of CHIKV-VLPs with SRM was found to inhibit the interaction of CHK152 with the CHIKV-VLPs (Fig. 4G), which was then well-confirmed in the CHIKVpp entry assay (Fig. 4I). Considering that SRM did not compete with other agents tested, such as MFA (Fig. 4J and K), the results suggest that SRM binds the region close to the target sites of CHK152 and Apt#1, acting as their direct competitor.

### Aptamer target regions and antiviral mode

**Fig 5:**
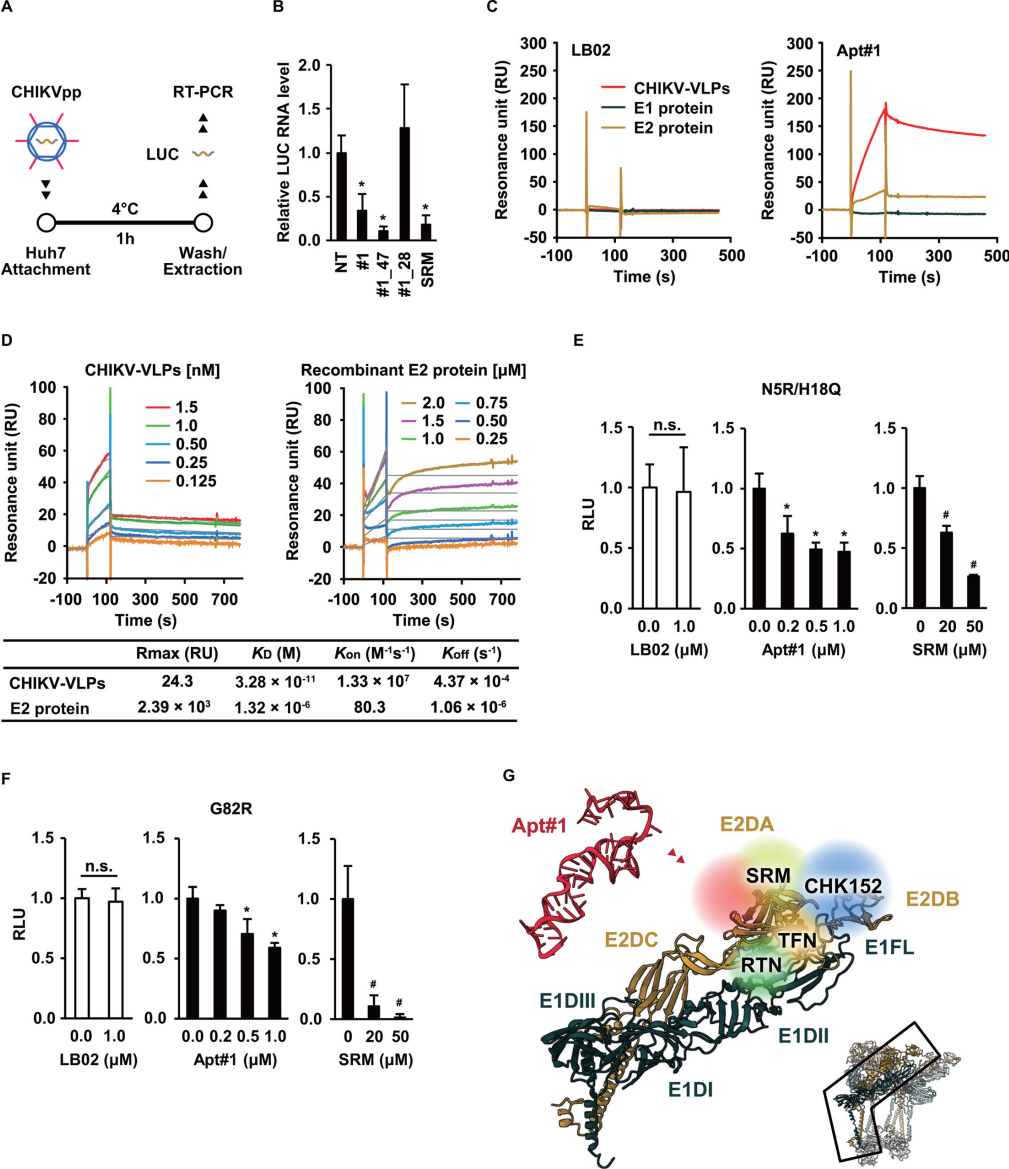
Aptamer-impeded viral attachment. (**A**) Scheme of viral attachment assay. Huh7 cells were transduced with CHIKVpp with the envelope proteins of the strain 37997 preincubated with aptamers or SRM at final concentrations of 1 µM or 20 µM, respectively, for 1 hour at 4°C, and total RNA was extracted after washing. (**B**) Messenger RNA levels of the *luciferase* gene were normalized to those of *GAPDH*, and then the relative values were compared with those of control NT. Data are presented as mean ± SD (*n* = 6). Statistical differences among treatment groups were examined by two-way ANOVA and then by Tukey–Kramer test. ^*^ *P* < 0.05 versus NT. (**C**) A random LB02 library (left) or Apt#1 (right) was subjected to SPR analysis to determine its affinity to recombinant E1 and E2 proteins and CHIKV-VLPs. (**D**) The dissociation constant of Apt#1 was evaluated; the binding ability of Apt#1 to different amounts of CHIKV-VLPs (left) and recombinant E2 proteins (right) was investigated using a BIAcore system, and the estimated parameters, Rmax, Kon, Koff, and KD, are analyzed by a univalent fitting model. The values obtained from CHIKV-VLPs should be taken as a reference as the stoichiometry of the aptamer to VLP is unknown. (**E**) The entry of CHIKVpp with the envelope proteins of the strain 37997 harboring the E2-N5R/H18Q mutations was evaluated in the presence of LB02, Apt#1, or SRM at the indicated doses, as in Fig. 2A. Data are presented as mean ± SD (*n* = 4). Statistical differences among treatment groups were examined by two-way ANOVA and then by Tukey–Kramer test. ^*^ *P* < 0.05 vs 0 µM; n.s., no significant difference between the indicated groups. (**F**) The entry of CHIKVpp with the envelope proteins of the strain 37997 harboring the E2-G82R mutation was evaluated in the presence of LB02, Apt#1, or SRM at the indicated doses, as in panel E. Data are presented as mean ± SD (*n* = 4) with statistical analyses, as in panel E. (**G**) Predicted Apt#1-interacting region of CHIKV. The E1/E2 dimer of the CHIKV spike unit surrounded by magenta lines (PDB: 6NK5, bottom right) is magnified in the center. E1 and E2 proteins are depicted in dark green and gold, respectively, and individual domains including the fusion loop (FL) in E1 are shown around the corresponding area. Reported antiviral-interacting and the predicted Apt#1-binding sites are superimposed on the image of the E1/E2 dimer.

Predicated on the competition between Apt#1 and SRM, which primarily targets E2DA on the surface of the spike unit (Fig. 3A through C), the effect of Apt#1 on the attachment of CHIKVpp to cells was assessed in cell culture, as reported (28, 36). Briefly, cells were incubated with CHIKVpp in the absence or presence of aptamers at 4°C to prevent uptake of viral particles attached to the cells (Fig. 5A). After washing, total RNA was extracted from the treated cells and subjected to real-time PCR to detect the *firefly luciferase* gene in CHIKVpp. The relative level of *luciferase* mRNA in cell-attached CHIKVpp was decreased by Apt#1, as in the case of SRM (Fig. 5B), but not in the absence of the aptamer, with a random library, and with Apt#1_28, which lacks neutralizing activity (Fig. 2F), indicating antiviral activity of the aptamers by inhibiting virus–host cell attachment. In addition, detailed SPR analysis revealed that Apt#1 binds not to E1 but to the E2 protein (Fig. 5C and D). In parallel, the effect of several E2 mutations at the known SRM target sites on the neutralizing activity of Apt#1 was investigated. The moderate resistance reportedly conferred by the double mutation N5R/H18Q (25) was not really observed against SRM as well as Apt#1 in our assay system (Fig. 5E). Alternatively, the G82R mutation conferred increased sensitivity to SRM as reported (25), while not to Apt#1 (Fig. 5F). Collectively, Apt#1 was indicated to target other sites in the E2 protein than reported, such as N5, H18, and G82. Overall, Apt#1 was demonstrated to inhibit CHIKV entry by blocking the viral attachment to cells via interaction with domain A of the E2 protein in an SRM-competitive manner (Fig. 5G).

### Broad potential of aptamer

**Fig 6:**
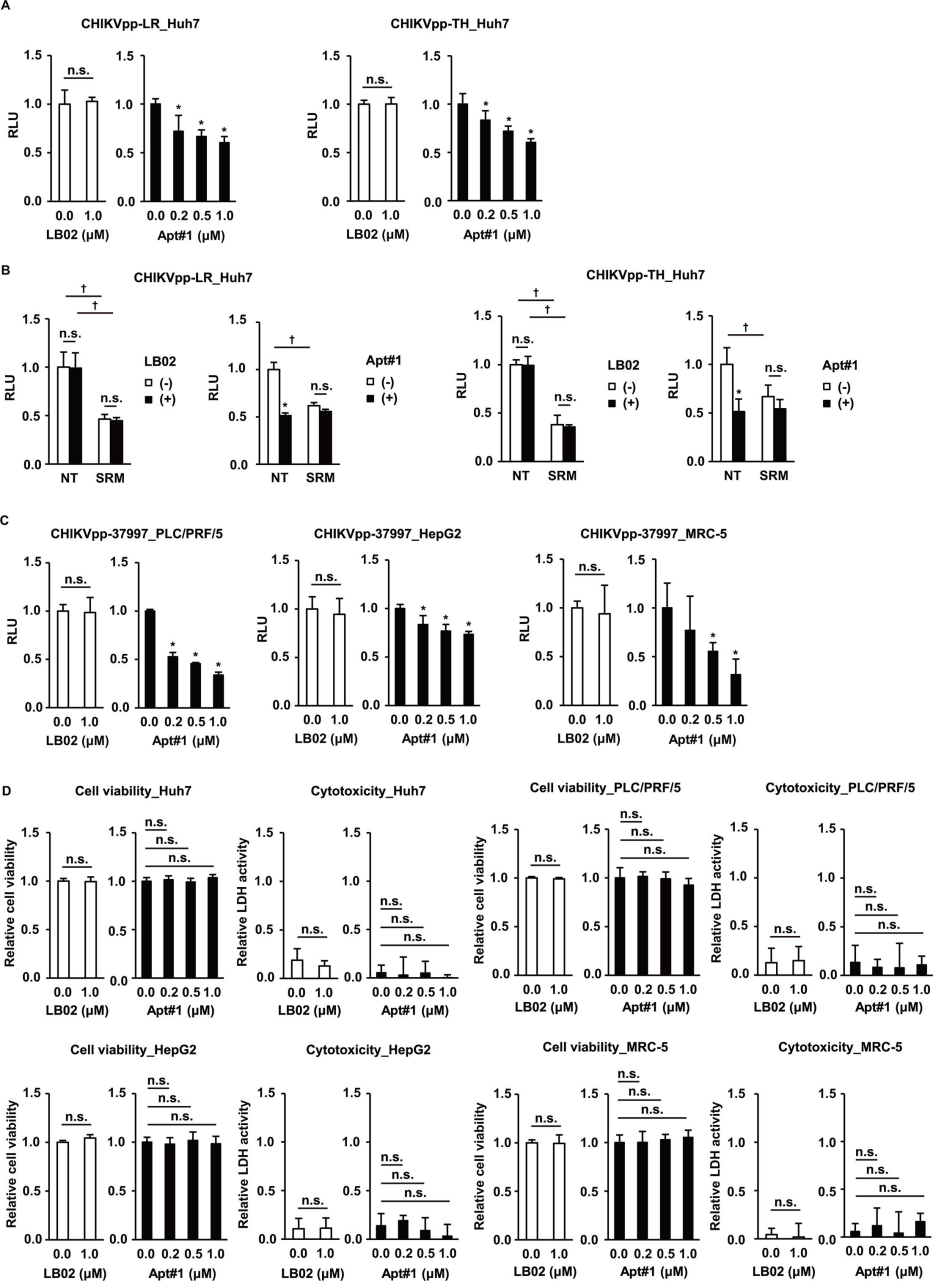
Neutralizing activity and cytotoxic effect of the aptamer across viral strains and cell types. (**A**) The entry of CHIKVpp with the envelope proteins of the strain LR, and TH was evaluated in Huh7 cells in the presence of LB02 or Apt#1, as in Fig. 2A. Data are presented as mean ± SD (*n* = 4). ^*^ *P* <0.05 versus 0 µM; n.s., no significant difference between the indicated groups. (**B**) The effects of co-treatment with LB02 or Apt#1 at 1 µM and SRM at 20 µM on the entry of CHIKVpp with the envelope proteins of the strain LR and TH were examined in Huh7 cells, as in Fig. 3D. Data are presented as mean ± SD (*n* = 4). ^*^ *P* <0.05 for the effect of Apt#1 in the absence of SRM; n.s., no significant difference between the indicated groups. (**C**) The entry of CHIKVpp with the envelope proteins of the strain 37997 was evaluated in PLC/PRF/5, HepG2, and MRC-5 cells in the presence of LB02 or Apt#1, as in Fig. 2C. Data are presented as mean ± SD (*n* = 4). ^*^ *P* <0.05 versus 0 µM; n.s., no significant difference between the indicated groups. (**D**) After the treatment of Huh7, PLC/PRF/5, HepG2, or MRC-5 cells for 5 days, the effects on cell viability and cytotoxicity of Apt#1 were determined by the levels of dehydrogenase-converted tetrazolium salt and LDH release, respectively. The relative cell viability was calculated in comparison to 0 µM, and the relative LDH release was calculated in comparison to the high control produced by the manufacturer-provided cell lysis buffer. Data are presented as mean ± SD (*n* = 4). n.s., no significant difference between the indicated groups. Statistical differences among treatment groups were examined by two-way ANOVA and then by Tukey–Kramer test.

Finally, we have investigated the inhibitory potential of the aptamer in various biological settings. More contemporary and pathogenic strains represented by Indian Ocean Lineage (IOL) Reunion_05-AM258990-2005 (LR) with an adaptive mutation E1-A226V and a sub-lineage Thailand-BHTD20-WM48-2020 (TH) with novel adaptive mutations E1-K211E and E2-V264A have been utilized (37). In fact, Apt#1 has blocked the entry of CHIKVpp with the envelope proteins of these strains (Fig. 6A) in an SRM-competitive manner (Fig. 6B).

Meanwhile, the investigation was extended to additional cell lines, and Apt#1 did, indeed, neutralize the CHIKVpp with the envelope proteins of the strain 37997 in additional liver cancer cell lines PLC/PRF/5 and HepG2 cells (Fig. 6C). The same was true for MRC-5 cells (Fig. 6C) with notably abundant cell surface expression of matrix remodeling-associated 8 (MXRA8), a critical entry receptor for CHIKV (38). Here, cell viability and cytotoxicity were not affected by Apt#1 or a random library LB02 (Fig. 6D). Altogether, the aptamer was demonstrated to be capable of impeding CHIKVpp entry across viral and cell types, implying its broad potential.

## DISCUSSION

The generation and characterization of various antivirals, regardless of drug modality, is essential to find a treatment for CHIKV infection. In the present study, we developed an RNA aptamer targeting CHIKV using VLPs and characterized its properties using CHIKVpp. By combining Apt#1 with anti-CHIKV agents, we found that Apt#1 inhibits CHIKVpp entry through the attachment of the E2 envelope protein to cells and exerts additional effects with individual anti-CHIKV agents other than SRM, conceivably targeting different sites of interaction. As the E2 protein plays an essential role in the attachment of CHIKV to cells (39), it is reasonable that Apt#1, which was indicated to interact with the E2 protein, would inhibit attachment. For the estimation of its Apt#1-binding site, the KYG software estimated potential RNA interface residues in the E2 protein, consistent with the results of the CHIKVpp analysis. Given the charge and hydrophobicity of the spike units of CHIKV, there does not appear to be a favorable site(s) for RNA binding on the spike units. These findings suggest that Apt#1 binds to the CHIKV E protein based on shape complementarity, which is facilitated by the intricate 3D conformation and high flexibility of the aptamer and is advantageous for targeting a structure as complex as the viral particle (40). To predict the interaction interfaces with an atypical RNA molecule such as the aptamer above, the KYG software can be useful; the program uses 3D structures of proteins for the prediction, relying not on the typical structural features of common RNA molecules but on the spatial arrangement of atoms in the protein and RNA molecules (41). Nevertheless, the actual interaction site of Apt#1 with CHIKV apparently remains to be identified by structural analysis.

As oligonucleotide therapeutics, aptamers have several advantages over other agents, including high affinity based on avidity, chemical synthesis, and low antigenicity. Although virus-targeting aptamers have been developed (42, 43), few aptamers possess virus-neutralizing activity at nanomolar concentrations. One of the barriers to the successful development of aptamers against viruses could be the dependence of the SELEX method on recombinant proteins. They have been widely used for the generation of antiviral aptamers (11, 44), but may be inadequate for SELEX targeting viral membrane proteins, as a potential failure to recapitulate the native conformation on the viral surface may allow aptamers to bind to unexposed regions and/or proteins. VLPs, in terms of conformation, appear to be superior to recombinant proteins as SELEX baits for viral membrane proteins. In addition, single-round infectious particles have recently become available as readymade products. Taking advantage of these materials, our VLP-exploited platform applied to different virus particles will provide a more viable option for the future generation of antiviral aptamers.

The isolated aptamer Apt#1 was analogous to SRM in terms of target sites and inhibition of viral attachment to cells but had a lower nanomolar IC50 value and further combinatorial activity with anti-CHIKV agents (45). Furthermore, the mutations on the SRM target sites in the E2 protein did not affect the neutralizing activity of Apt#1 (Fig. 5E and F). G82 is located at the center of the spike where the A domains of three E2 subunits are found and maps to the area that interacts with the entry receptor MXRA8 (25). Given that G82R increases the affinity for SRM and blocks the viral attachment, Apt#1 appears to interfere with entry mechanisms other than the E2-MXRA8 axis. Also, Apt#1 was irrelevant to N5R/H18Q indicated to sidetrack SRM from the core of E1/E2 heterodimer/spike to facilitate fusion. Concurrently, the lack of SRM resistance could be due to strain differences as the mutations were selected in the strain LS3 (25), whose E2 protein sequences differ by more than 5% from those of the strain 37997 used in our study. Thus, Apt#1 was shown to behave similarly to SRM, but differently, and a full screen for the elucidation of specific targets is warranted in the forthcoming study. Collectively, these observations presented Apt#1 as a unique antiviral for CHIKV, not just an alternative to SRM.

In the chemical genetic approach and SPR analysis (Fig. 3D and 4D), RTN and TFN, antiviral agents predicted to interact with E1 and E2 proteins, did not significantly affect the interaction of Apt#1 with CHIKVpp/-VLPs. A recent high-throughput computational screening of four datasets from ZINC, ChemDiv, and DrugBank targeting the CHIKV E1/E2 dimer revealed that RTN and TFN possessed highest affinities (27). The docking sites of RTN and TFN were calculated to be a narrow channel surrounded by a fusion loop in E1, E2DA/DB, and a cavity between E1DII and the β-ribbon of the E2 protein (Fig. 5G); accordingly, Apt#1 was indicated to target physically distinct regions. Meanwhile, both RTN and TFN have been proposed to stabilize the E1/E2 dimer through dockings, thereby preventing the subsequent fusion process. As Apt#1 suppressed CHIKVpp entry in cell culture without interruption by RTN or TFN, it was rationalized that the antiviral activity might be exerted by mechanisms other than fusion inhibition. Pharmacologically, RTN and TFN are derivatives of quercetin, a flavonoid found in foods, and used as dietary supplements for their many health benefits, including antioxidant and anti-inflammatory properties (46, 47). The therapeutic potential is widely recognized in various diseases ranging from cancer to viral infections, and both molecules are in clinical trials for the treatment of coronavirus disease 2019 (COVID-19). Optimized and combined administration of RTN/TFN and Apt#1 may, therefore, provide therapeutic and prophylactic strategies and may even overcome viral resistance.

CPH and IPH are well-known entry inhibitors of various RNA viruses including CHIKV and pseudotypes (48, 49), via inhibition of clathrin-dependent endocytosis (31, 32) and endolysosome functions (33, 34), respectively. Our combinatorial treatments, therefore, confirmed that aptamers interfered with E proteins at steps other than those targeted by CPH/IPH, subsequently leading to the identification of aptamer-intercepted viral attachment indeed. Admittedly, the potential relationship between the antiviral modes of action of aptamers on E proteins and additional entry pathways (50) remains to be assessed, and a more in-depth study in the context of virus–host interaction would be warranted in the future.

The attractiveness of aptamers has also been demonstrated by their broad potential in various biological settings. In fact, Apt#1 also neutralized IOL strains with adaptive mutations and genetically distant and different in E2 protein sequence by 6%–7% from strain 37997 (Fig. 6A). Hence, the multicontact nature of the aptamer to the target would overcome such a degree of strain diversity as above and retain the ability to bind to the E2 protein. This property may hold promise for intervention as mutations in E2 often lead to escape from specific neutralizing antibodies (35). In addition, the aptamer-mediated blockade of CHIKVpp entry, primarily monitored in Huh7 cells, was also observed in several cell lines. Of the liver cancer cells in our possession, HepG2 cells are a hepatoblastoma-derived cell line (51) with reported susceptibility to CHIKV (52), while PLC/PRF/5 cells are a hepatoma cell line (53) with hepatitis B virus integration (54), each with very unique properties. MRC-5 cells are a human primary lung fibroblast (55) and another representative CHIKV-permissive cell line (38). These cell lines have distinct characteristics, including viral tropism, which suggests differential expression of cell surface receptors. Indeed, MXRA8 is known to be highly expressed in MRC-5 (38) and HepG2 (56) cells, although levels are relatively low in Huh7 cells (38). Apt#1 could, therefore, interfere with the CHIKV entry independent of the MXRA8 expression, and such relief from viral and cellular factors as described above, together with the demonstrated cytosafety (Fig. 6D), would be strategically advantageous.

Limitations of the current study include the lack of evaluation of the aptamer using an authentic CHIKV challenge. While high containment of the virus is a technical hurdle, corroboration of the neutralizing effect in cell culture and even *in vivo* would be preferable to practical application of the fundamental findings presented in this study. Moreover, genuine viral morphology and fusion kinetics would provide improved insights into the antiviral modes of target shape-sensitive aptamers and will be investigated further. Also, the aptamers selected this time missed really prominent motif families and the neutralizing potency was at modestly below micromolar IC50. The complex surface structures of viral particles, comprising multiple proteins, present a challenge for aptamers in terms of identifying specific binding sites promptly. This complexity may necessitate the implementation of additional rounds of selection for enrichment to isolate more potent clones, leaving room for further extensive characterization and optimization of SELEX in future studies. Moreover, the findings from the qualitative SPR analyses in concert with the quantitative CHIKVpp assays have paved the way for mechanistic investigation of neutralizing agents. Particularly, detailed chemical kinetics of inhibition elucidated by quantitative SPR analyses will reveal the mode of neutralization and serve to develop new anti-CHIKV strategies using aptamers. At the same time, our assessment of E2DA as the interface for the aptamers *in silico* and in cell culture would be largely confirmed by the resolution of the complex structure. Answering these questions will facilitate the efficiency and endorse the validity of our technology to generate virus-neutralizing aptamers against other viral particles of interest beyond this achievement.

In conclusion, we have established a combined system for the discovery of aptamers to CHIKV-VLPs and the evaluation of neutralizing activity by CHIKVpp. The successfully raised aptamer possessed neutralizing activity, which was suggested to be mediated by CHIKV E2DA through a chemical genetic approach. The common availability of the experimental tools, i.e., VLPs, pseudoparticles, and chemicals, will constitute a versatile platform conducive to the discovery of neutralizing molecules as well as the development of strategies against emerging, reemerging, and other viruses of interest.

## MATERIALS AND METHODS

### Compounds and cells

VLPs of CHIKV and Japanese encephalitis virus (JEV) were purchased from The Native Antigen Company (Kidlington, UK). Mefenamic acid (MFA) was purchased from Tokyo Chemical Industry (Tokyo, Japan). Epigallocatechin gallate (EGCG) and suramin (SRM) were purchased from Cayman Chemical (Ann Arbor, MI, USA). Rutin (RTN) and taxifolin (TFN) were purchased from Selleck Chemicals (Houston, TX, USA). Chlorpromazine (CPH) and imipramine (IPH) were purchased from NACALAI TESQUE, INC. (Kyoto, Japan). HEK293T cells were obtained from the American Type Culture Collection (Manassas, VA, USA), and Huh7 cells, PLC/PRF/5 cells, HepG2 cells, and MRC-5 cells were obtained from Japanese Collection of Research Bioresources Cell Bank (Osaka, Japan) and cultured according to the individual protocols (https://cellbank.nibiohn.go.jp/~cellbank/en/search_res_det.cgi?ID=385; https://cellbank.nibiohn.go.jp/~cellbank/en/search_res_det.cgi?ID=1794; https://cellbank.nibiohn.go.jp/~cellbank/cgi-bin/search_res_det.cgi?ID=2936; https://cellbank.nibiohn.go.jp/~cellbank/cgi-bin/search_res_det.cgi?ID=1798). Recombinant E1 and E2 envelope proteins in CHIKV were purchased from The Native Antigen Company (Kidlington, UK) and Sino Biological, Inc (Beijing, China), respectively. Cytotoxicities of aptamers were determined with the lactate dehydrogenase (LDH) cytotoxicity detection kit (NACALAI TESQUE, INC.).

### SELEX

Aptamer selection was carried out as reported previously, and detailed conditions are described in Table 1 (9). Briefly, the LB02 library was amplified by PCR with ExTaq DNA polymerase (TaKaRa Bio, Shiga, Japan). The library and primer set are as follows: LB02, 5′-CTC TCA TGT CGG CCG TTA [N40] CGT CCA TTG TGT CC C-3′ (where N40 stand for 40-nucleotide random sequence); Lb02 forward primer, 5′-TAA TAC GAC TCA CTA TAG GGA CAC AAT GGA CG-3′; Lb02 reverse primer, 5′-CTC TCA TGT CGG CCG TTA-3′ (T7 promoter sequence is underlined). The library was purchased from GeneDesign (Ibaraki, Osaka, Japan). The resultant double-stranded DNAs were subjected to *in vitro* transcription using 2'-fluoro-CTP, 2' -fluoro-UTP, ATP, and GTP (at final concentration of 2.5 mM each) and Y639F mutant T7 RNA polymerase (at final concentration of 40 ng/µL). After an overnight incubation at 37°C, single-stranded RNAs (ssRNAs) were purified with phenol/chloroform (NACALAI TESQUE, INC.) to remove proteins and further purified with ultrafiltration column, Amicon Ultra 0.5 mL (molecular weight cutoff (MWCO) 30 kDa [Millipore, Burlington, MA, USA]) to remove NTPs by centrifugation at 14,000 × *g* for 5 minutes. After discarding flow through, distilled water was added in the device up to 500 µL, and this washing procedure (centrifugation) was repeated five times. Before selection, RNA pools were denatured at 95°C for 5 minutes in 100 µL SELEX buffer (145 mM NaCl, 5.4 mM KCl, 0.8 mM MgCl_2_, 1.8 mM CaCl_2_, and 20 mM Tris HCl pH 7.6) in the presence of heparin at a final concentration of 2 mg/mL and yeast tRNA at a final concentration of 0.05 mg/mL, followed by cooling on ice for 3 minutes. The refolded RNA pool and the VLPs were mixed and incubated for 30 minutes with agitation at 1,500 rpm using PowerBLOCK Shaker (ATTO Corporation, Tokyo, Japan). For separation of aptamers complexed with VLPs from free RNA sequences, an ultrafiltration column, Vivaspin 500 with 100 K MWCO polyether sulfone (PES) (Sartorius AG, Goettingen, Germany), was used for the separation by centrifugation at 14,000 × *g* for 1 minute. After discarding flow through, 500 µL of SELEX buffer was added, followed by centrifugation several times as a washing process (Table 1). Retained solution-containing aptamers complexed with VLPs in the column were recovered and added to phenol/chloroform (NACALAI TESQUE, INC.) for extraction of bound RNA sequences. After ethanol precipitation with Dr. GenTLE Precipitation Carrier (TaKaRa Bio), the entire amount of the collected RNAs was subjected to reverse transcription with ThermoScript Reverse Transcriptase (LifeTechnologies, Carlsbad, CA, USA) according to the manufacturer’s protocols, and then the ssDNAs were subjected to PCR amplification with ExTaq DNA polymerase (TaKaRa Bio) until appropriate PCR cycles to avoid the appearance of nonspecific DNA fragments; the number of PCR cycles, approximately 12 to 15, was determined by actually amplifying a fraction of ssDNA each time and examining the banding by electrophoresis. The amplified double-stranded DNAs were transcribed with Y639F mutant T7 RNA polymerase and modified NTPs described above. After the first round, refolded RNA pools were passed through Amicon Ultra 0.5-mL 30K ultrafiltration column at 14,000 × *g* for 10 minutes before selection with VLP to reduce sequences bound nonspecifically to the column. At the final round, the enriched RNA library was mixed with CHIKV-VLPs as well as JEV-VLPs as a positive and negative target, respectively. Then, RNA sequences bound to target and nontarget VLPs were subjected to HTS for efficient *in silico* analysis.

### *In silico* analysis

The sequencing procedure was carried out using the Ion PGM system with an Ion 314 chip according to the manufacturer’s protocols (LifeTechnologies). Sequencing data were analyzed with RaptRanker (3) and FASTAptamer (23), as described previously (9). Briefly, after trimming the accessory sequences such as a barcode, adapter, and T7 promoter sequence, sequences coding aptamers were analyzed. Furthermore, sequences of less than four reads were removed in this analysis. Subsequently, cluster analysis was carried out with an edit distance set to 6; thus, sequences possessing fewer than six base differences were assigned into an identical cluster. Then, the sequences with highest read number or read per million (RPM) in each cluster were extracted as a representative sequence in each cluster. Furthermore, the candidates were narrowed down to sequences with more than 100 RPM in the positive data. Of those, sequences showing enrichment values 0.5 or less were identified as final candidates. The enrichment value “y/x” 0.5 or less means that the RPM value of the sequence was more than twice as enriched as in the positive data (set to “x”) obtained from CHIKV-VLPs compared to the false-positive data (set to “y”) obtained from JEV-VLPs (Table S1).

### Surface plasmon resonance (SPR)

SPR analyses were carried as previously described using BIAcore 2000 instrument (Cytiva, Marlborough, MA, USA) (57). Briefly, to examine the binding ability of aptamers to CHIKV-VLPs, around 1,000 resonance units (RUs) of aptamer with poly(A)_16_ tail at the 3’ end was immobilized through oligo d(T)_16_ labeled with biotin at the 3’ end onto a streptavidin (SA) sensor chip (Cytiva). After immobilization of the aptamer, CHIKV-VLPs at a final concentration of 20 ng/µL were injected for 120 seconds. SELEX buffer was employed as running buffer. To regenerate sensor chips, a solution consisting of 4 M urea was injected for 1 minute in the regeneration process. To examine the inhibitory effects of aptamers on antibody–VLP interaction, about 1,500 RUs of a protein A was immobilized onto a CM5 sensor chip (Cytiva) by amino coupling. After immobilization of a protein A, CHK152 at a final concentration of 100 nM was injected, resulting in immobilization at about 100 RUs. Subsequently, premixtures of CHIKV-VLPs with compounds and aptamers at indicated concentrations were further injected. In the regeneration process, 10 mM glycine-HCl solution was used. A mixture of CHIKV-VLPs with either SRM, RTN, TFN, or MFA at a final concentration of 50 µM, 1 mM, 400 µM, or 200 µM, respectively, was assessed as the analyte on the Apt#1-immobilized sensor chip. A mixture of CHIKV-VLPs with either SRM, RTN, TFN, MFA, or Apt#1 at a final concentration of 50 µM, 1 mM, 400 µM, 200 µM, or 1 µM, respectively, was assessed as the analyte on the CHK152-immobilized sensor chip.

As for the data in Fig. 4F, Apt#1 was immobilized onto the SA sensor chip mediating oligo d(T)_16_ labeled with biotin, as described above. After immobilization of Apt#1, SRM at a final concentration of 50 µM, MFA at a final concentration of 1,000 µM, and a running buffer as control were injected, and then CHIKV-VLPs at a final concentration of 20 ng/µL were injected.

As for the data in Fig. 5C, Apt#1 and a random library LB02 were immobilized into the SA chip in the same protocols. After immobilization, both recombinant E1 and E2 proteins at final concentrations of 1 µM and CHIKV-VLPs at a final concentration of 20 ng/µL were injected to examine the affinity of Apt#1 to CHIKV relevant materials. Regarding Fig. 5D, Apt#1 was similarly immobilized, and then the indicated concentrations of CHIKV-VLPs and recombinant E2 proteins were injected at a flow rate of 40 µL/minute to estimate several affinity parameters. The parameters such as dissociation constant were calculated by BIAevaluation software (Cytiva).

### DNA and RNA oligonucleotides

DNA and RNA oligonucleotides were synthesized by FASMAC Co., Ltd. (Atsugi, Kanagawa, Japan).

### Plasmids

The structural polyprotein ranging from E3 to E1 of CHIKV strains 37997, Reunion_05-AM258990-2005, and Thailand-BHTD20-WM48-2020 were codon-optimized and synthesized (FASMAC Co., Ltd.) and amplified as follows: forward primer, 5'- CCC CCT CGA GGT CGA CGG CCA CCA TGA GCC TGG CCC TCC CTG TCC TTT GCC TC-3′; reverse primer, 5′-TAT CAA GCT TAT CGA TTC AGT GTC TGG AGA AGG ACA CAC AAA GCA C-3′. The fragments were cloned into pEBMulti-Neo (FUJIFILM Wako Pure Chemical Corporation, Osaka, Japan) using the In-Fusion HD cloning kit (TaKaRa Bio), producing pEBMN-CHIKV-E, pEBMN-CHIKV-LR-E, and pEBMN-CHIKV-TH-E. The mutations in E2 were introduced by the primers above with 5′-GGT ATT AAG ACA AAC GAC AGT CAC GAT TGG-3′ and 5′-CCA ATC GTG ACT GTC GTT TGT CTT AAT ACC-3′ for D59N, 5′-CAA CCA AGG ACC GGT TCA ACG TCT ACA AGG-3′ and 5′-CCT TGT AGA CGT TGA ACC GGT CCT TGG TTG-3′ for N5R, 5′-CAT ATC TGG CCC AGT GCC CCG ACT GCG GAG-3′ and 5′-TTC TCC GCA GTC GGG GCA CTG GGC CAG ATA TGG CC-3′ for H18Q, and 5′-ATG CCG AGA GAG CTA GGC TTC TCG TGC GAA-3′ and 5′-TTC GCA CGA GAA GCC TAG CTC TCT CGG CAT-3′ for G82R, followed by cloning similarly as above into pEBMulti-Neo producing pEBMN-CHIKV-E (E2-D59N), pEBMN-CHIKV-E (E2-N5R), pEBMN-CHIKV-E (E2-H18Q), and pEBMN-CHIKV-E (E2-G82R). A fragment was then amplified using the above primer sets for the structural polyprotein and N5R and pEBMN-CHIKV-E (E2-H18Q) as a template, followed by cloning into pEBMulti-Neo in a similar manner as above, resulting in pEBMN-CHIKV-E (E2-N5R/H18Q). The lentiviral luciferase expression vector pLenti CMV Puro LUC (w168-1) (#17477) and the lentiviral packaging plasmid psPAX2 (#12260) were obtained through Addgene (www.addgene.org).

### Western blotting

Total protein was resolved by SDS-PAGE and subjected to Western blotting as described previously (57). CHIKV E proteins were detected using rabbit anti-chikungunya 181/25 polyclonal antibody (IBT BIOSERVICES, Rockville, MD, USA) and horseradish peroxidase (HRP)-conjugated anti-rabbit IgG antibody (Jackson ImmunoResearch Laboratories, West Grove, PA, USA). Alpha tubulin proteins (TUBA) were detected as an internal control using mouse monoclonal anti-alpha tubulin antibody (Millipore) and HRP-conjugated anti-mouse IgG antibody (Jackson ImmunoResearch Laboratories).

### CHIKVpp production

As described previously (58), HEK293T cells were transfected with 2.5 µg of the CHIKV structural polyprotein expression vector (pEBMN-CHIKV-E), 5 µg of the luciferase reporter vector (pLenti CMV Puro LUC (w168-1)), and 5 µg of packaging vector (psPAX2) by Lipofectamine 3000 (LifeTechnologies), with the medium replaced at 6 hours post-transfection. The supernatant was harvested 2 days later, filtered through a 0.22-µm syringe filter (Pall, Port Washington, NY, USA), and stored at −80°C.

### CHIKVpp transduction

CHIKVpp was incubated with or without compounds, antibodies, or aptamers at 37°C for 1 hour and added to 1 × 10^4^ Huh7, PLC/PRF/5, HepG2, and MRC-5 cells in a 96-well plate, with the medium replaced at 6 hours post transduction. The cells were incubated for 3 or 5 days depending on assays, and luciferase activity was measured using the Bright-Glo luciferase assay system (Promega, WI, USA), normalized to cell viability determined by cell counting kit-8 (Dojindo, Kumamoto, Japan) immediately before harvesting and producing relative luciferase activity. Anti-Chikungunya E2 protein monoclonal antibody CHK152 (Antibody Research, MO, USA) as a neutralizing antibody and mouse IgG2B isotype control (R&D Systems, Minneapolis, MN, USA) were utilized.

### Prediction of RNA interface

To predict the RNA interface in CHIKV-VLPs and -pp, the KYG program (http://cib.cf.ocha.ac.jp/KYG/index.php) was used (1). Briefly, the CHIKV structure was obtained from PDB (ID 3J2W), and the data were limited in the trimer of the E1/E2 dimer by suing the UCSF Chimera (https://www.cgl.ucsf.edu/chimera/). The data for the trimer were examined by the KYG, and then residues whose prediction values are more than 0 are denoted as colored amino acids in Fig. 3 depending on their prediction values. The structures of the E1/E2 dimer of the CHIKV spike unit (PDB ID: 6NK5) (Fig. 5G) and Apt#1 calculated by HDOCK (http://hdock.phys.hust.edu.cn/) (Fig. 5G) were also depicted using UCSF Chimera.

### CHIKVpp attachment analysis

In brief, as described previously (2), 1 × 10^4^ Huh7 cells in a 96-well plate were transduced with CHIKVpp preincubated with SRM or aptamers at 4°C for 1 hour. Subsequently the cells were washed three times, and then total RNA suspected of including the luciferase reporter gene was extracted for RT-PCR.

### RT-PCR

Total RNA was extracted and subjected to cDNA synthesis using SuperPrep Cell Lysis & RT Kit for qPCR (Toyobo, Osaka, Japan), followed by real-time PCR using the QuantStudio 1 real-time PCR System (ThermoFisher SCIENTIFIC, Waltham, MA, USA) and PowerUp SYBR Green Master Mix (ThermoFisher SCIENTIFIC). The primers were used as follows: 5′-CTG GGC TAC ACT GAG CAC C-3′ and 5′-AAG TGG TCG TTG AGG GCA ATG-3′ for the GAPDH gene; 5'- ACT GGG ACG AAG ACG AAC AC-3′ and 5′-GGG TGT TGG AGC AAG ATG GA-3′ for the firefly luciferase gene. RNA levels of the luciferase gene were normalized to the expression levels of the GAPDH gene by using the delta–delta Ct method, and then the normalized values of the luciferase gene in various treated cells were expressed as RNA levels relative to those of the control group in Huh7 cells without any treatments as one.

### Statistical analysis

The bar graphs are presented as means ± standard deviations (SDs). Statistical differences among treatment groups were examined by two-way analysis of variance (ANOVA) and then by Tukey–Kramer test. In Fig. 2C and F, the statistical significance of the aptamer treatment at the indicated concentrations is shown by the colored asterisks corresponding to the individual aptamers. In Fig. 2D, no statistical significance of the aptamer treatment at the indicated concentrations is shown by the colored bars and n.s. corresponding to the individual aptamers. In Fig. 3D, E and F, the statistical significance of the aptamer treatment in the absence and presence of the antivirals is indicated by the asterisks and hashes, respectively, and that of the antiviral treatment in the absence and presence of the aptamer is indicated by daggers. In Fig. 4C, the statistical analyses were performed as in Fig. 3D. In Fig. 4H, the statistical significance of the aptamer treatment compared to no treatment is indicated by an asterisk, and no statistical significance between the indicated comparisons is shown by n.s. In Fig. 4I, the statistical significance of CHK152 treatment in the absence of SRM is indicated by an asterisk, and that of SRM treatment in the absence of CHK152 is indicated by a dagger. In Fig. 4K, the statistical significance of MFA treatment in the absence and presence of SRM is indicated by an asterisk and a hash, respectively, and that of SRM treatment is indicated by daggers. In Fig. 5B, the statistical significance of the aptamer treatment is indicated by asterisks, and no statistical significance between the indicated comparisons is shown by n.s. In Fig. 5E and F, the statistical significance of the aptamer treatment is indicated by asterisks, and that of the SRM treatment is indicated by hash marks. In Fig. 6A and C, the statistical significance of the aptamer treatment is indicated by asterisks, and no statistical significance between the indicated comparisons is shown by n.s. In Fig. 6B, the statistical significance of the aptamer treatment is indicated by asterisks, and that of the SRM treatment is indicated by daggers. No statistical significance between the indicated comparisons is shown by n.s. In Fig. 6D, no statistical significance between the indicated comparisons is shown by n.s. For all statistical analyses, the alpha value was set at 0.05.

## Data Availability

All data associated with this study are present in the paper and the supplemental material.

## SUPPLEMENTAL MATERIAL

Table S1 jvi.02095-24-s0001.pdf HTS-retrieved sequences analyzed by FASTAptamer.

ASM does not own the copyrights to Supplemental Material that may be linked to, or accessed through, an article. The authors have granted ASM a non-exclusive, world-wide license to publish the Supplemental Material files. Please contact the corresponding author directly for reuse.
