# Supplementary material for: Generation of RNA aptamers against chikungunya virus E2 envelope protein
Source: J Virol. 2025 Feb 10;99(3):e02095-24. doi: 10.1128/jvi.02095-24 (PMC11915788; doi:10.1128/jvi.02095-24)
Supplement: Table S1 — HTS-retrieved sequences analyzed by FASTAptamer. [file jvi.02095-24-s0001.pdf]

Supplemental Table S1. HTS-retrieved sequences analyzed by FASTAptamer

ID[a]	Sequence	Length	Rank (x)	Reads (x)	RPM (x)[b]	Cluster (x)	Rank in Cluster (x)	Edit Distance (x)	Rank (y)	Reads (y)	RPM (y)[b]	Cluster (y)	Rank in Cluster (y)	Edit Distance (y)	Enrichment (y/x)
#1	GGGACACAATGGACGATCATATGCGGTGAAGGAACAAATATTTTATTAATTTAGCTAACGGCCGACATGAGAG	73	1	41	447.52	1	1	0							
N/E	GGGACACAATGGACGGATAGTTCTAGAATATATATAATAGTAACGGCCGACATGAGAG	58	2	34	371.11	2	1	0							
N/E	GGGACACAATGGAC	14	3	32	349.28	3	1	0	1	55	376.7	1	1	0	1.0785
#2	GGGACACAATGGACGATTTAACTTTGTTACCGACAGTCAGCCTGTTATAGGAAGGTAACGGCCGACATGAGAG	73	4	28	305.62	4	1	0							
#3	GGGACACAATGGACGATATACATTGATATATCATATTTGGCTGTGGGCTTCTAGCTAACGGCCGACATGAGAG	73	5	27	294.71	5	1	0							
#4	GGGACACAATGGACGTTACCTGCAATATACCTTTATTTTATAAGGAGTTGGTGAATAACGGCCGACATGAGAG	73	6	25	272.88	6	1	0							
N/E	GGGACACAATGGACGTTGAATGAGTTATATTTTAACGGCCGACATGAGAG	50	7	24	261.96	7	1	0							
#5	GGGACACAATGGACGTAGTGAAAGACTATAATAGGAATGTATGTGCTCGTTATATTAACGGCCGACATGAGAG	73	8	23	251.05	8	1	0							
#6	GGGACACAATGGACGCCAGCTACAGGAGAGTTAGTTTTATTACATTATTCTTTGGTAACGGCCGACATGAGAG	73	9	22	240.13	11	1	0							
#7	GGGACACAATGGACGACCGATGTATTTTTATGACCTGATATTTTTTGGGAGTAGGATAACGGCCGACATGAGAG	74	9	22	240.13	12	1	0							
#8	GGGACACAATGGACGGAATAATGTATTCTTACCAGCGCAAGCTGTGAGACATATGTAACGGCCGACATGAGAG	73	9	22	240.13	10	1	0							
N/E	GGGACACAATGGACGCGATACGAACAAAATCACTTATTATAACGGCCGACATGAGAG	57	9	22	240.13	9	1	0							
#9	GGGACACAATGGACGTAGTAATCACGTTAGAAGTGGGACATGATAGGCGTATGTATAACGGCCGACATGAGAG	73	13	21	229.22	16	1	0							
#10	GGGACACAATGGACGCTTTAGCAACAAGTGGAAGATTATACTTGACACTGATCTGTAACGGCCGACATGAGAG	73	13	21	229.22	13	1	0							
N/E	GGGACACAATGGACGCTTAGAAAAACACTTAAGCAGTTAACGGCCGACATGAGAG	55	13	21	229.22	14	1	0							
#11	GGGACACAATGGACGTATAAGTAGTATATATGAACAAGATTTAATTATAATATGATAACGGCCGACATGAGAG	73	13	21	229.22	15	1	0							
#12	GGGACACAATGGACGAATAGTTATTGATCGGAATTATAAAAAGACAAGGAGGCTAACGGCCGACATGAGAG	71	17	20	218.3	18	1	0							
#13	GGGACACAATGGACGAATACATAATATACAATTGACGGTGCATGCGGTTTCGTAATAACGGCCGACATGAGAG	73	17	20	218.3	19	1	0							
#14	GGGACACAATGGACGTAGAATGAGATATGCAACAAATGAGTTGTTGTTAAATTGATAACGGCCGACATGAGAG	73	17	20	218.3	17	1	0							
N/E	GGGACACAA	9	17	20	218.3	3	2	5	24	22	150.68	1	6	5	0.6902
#15	GGGACACAATGGACGCCAGAAGAAGTGATTAAACAGTAATAAACGCTAATTTTAACGGCCGACATGAGAG	70	17	20	218.3	20	1	0	3	38	260.27	3	1	0	1.1923
#16	GGGACACAATGGACGTAAGTAAATAAACCCTTTTTGGTCGATAACTAGCGAAGGCTAACGGCCGACATGAGAG	73	22	19	207.39	24	1	0							
#17	GGGACACAATGGACGTAAACGAGCGAACAGTTTTATCACTTATTCCTTCGTATGGTAACGGCCGACATGAGAG	73	22	19	207.39	25	1	0							
#18	GGGACACAATGGACGATGATCTCTTATATTTGACAAAAACCTATAGAGTGGATCTAACGGCCGACATGAGAG	72	22	19	207.39	22	1	0							
#19	GGGACACAATGGACGAATAACCCTTTGAAGAACCAATCTAATCAAGGCTGAGACGTAACGGCCGACATGAGAG	73	22	19	207.39	26	1	0							
#20	GGGACACAATGGACGTAACGTACACATTTGAAGCCGTTCTAGGATTTAACGATAGTAACGGCCGACATGAGAG	73	22	19	207.39	23	1	0							
N/A	GGGACACAATGGACGTGATAACAAAACCAAGTTTTAGGGTTGAGGACAACGAATCTAACGGCCGACATGAGAG	73	22	19	207.39	21	1	0							
N/A	GGGACACAATGGACGCTAGATCACAGCCGCATAAACTAAGGATTAATTGCAGTTATAACGGCCGACATGAGAG	73	28	18	196.47	27	1	0							
N/A	GGGACACAATGGACGCGTAAGCAAAAGGCGTAATTTACATAGAATACGCTAGATGTAACGGCCGACATGAGAG	73	28	18	196.47	29	1	0							
N/A	GGGACACAATGGACGGTAATACGTGAGAGCAGACATATGATTTTTTGATGAAAGCTAACGGCCGACATGAGAG	73	28	18	196.47	28	1	0							
N/A	GGGACACAATGGACGGTGATTATAACTGAGATTGAAATGGACCTGTAGGCACGTATAACGGCCGACATGAGAG	73	31	17	185.56	31	1	0							
N/A	GGGACACAATGGACGGTTTCAAAATTGTATTGCATTATATATTGAGGCAATGTAGTAACGGCCGACATGAGAG	73	31	17	185.56	39	1	0							
N/A	GGGACACAATGGACGACCAAAATATGTATCGAGGAACTTTACCTCCGTGTAGATAACGGCCGACATGAGAG	71	31	17	185.56	30	1	0							
N/A	GGGACACAATGGACGATACTACCTTAACAAAGACAGCTAACGGCCGACATGAGAG	55	31	17	185.56	37	1	0							
N/A	GGGACACAATGGACGCGATGCTTCAAAGTTTAGTACGAGAGTATTATGGAGTAAGTAACGGCCGACATGAGAG	73	31	17	185.56	32	1	0							
N/A	GGGACACAATGGACGCTAAAATTAAATGTGAAACACTACAATGCTATGTTACCATAACGGCCGACATGAGAG	72	31	17	185.56	38	1	0							
N/A	GGGACACAATGGACGGCATTAAAGGTTAGGCATTAACTATTAAACCTTGCTAACATAACGGCCGACATGAGAG	73	31	17	185.56	36	1	0							
N/A	GGGACACAATGGACGATAGAACTGTGAGAAGTTTAACTTGAGTCATCATAAGGCTTAACGGCCGACATGAGAG	73	31	17	185.56	33	1	0							
N/A	GGGACACAATGGACGGATAATAAGAAGTTGATACAATTTAGGATATCAGGTTTTCTAACGGCCGACATGAGAG	73	31	17	185.56	40	1	0							
N/A	GGGACACAATGGACGGAAATACATAAATCTAATAATCTTAAACGAGATAGGTATATAACGGCCGACATGAGAG	73	31	17	185.56	34	1	0							
N/A	GGGACACAATGGACGAATTTTGACACTATGATAAGAGCATAGCACGTGGTCGGATTAACGGCCGACATGAGAG	73	31	17	185.56	35	1	0							
N/A	GGGACGCAATGGACGGGCTGTATCAAATATTTGATTTTAACAAAGACAAAATGGATAACGGCCGACATGAGAG	73	42	16	174.64	42	1	0							
N/A	GGGACACAATGGACGTTTGAATTCAGGATAGATCCACACACCGAATCATGAGCCTTAACGGCCGACATGAGAG	73	42	16	174.64	41	1	0							
N/A	GGGACACAATGGACGTTAATTATGTGCTTTACTGGTAACGGCCGACATGAGAG	53	42	16	174.64	44	1	0							
N/A	GGGACACAATGGACGCCTAACTTTAAGTTAGCTGAAATTAGTGCACCTACTCGTGTAACGGCCGACATGAGAG	73	42	16	174.64	43	1	0							
N/A	GGGACACAATGGACGTTGCTAACTACCAACCTCATCAGGAATGATGGGTATAATCTAACGGCCGACATGAGAG	73	42	16	174.64	49	1	0							
N/A	GGGACACAATGGACGACCAACAGTTTAATACTTTGTTGCGATAGGAACAGTAGGCTAACGGCCGACATGAGAG	73	42	16	174.64	46	1	0							
N/A	GGGACACAATGGACGCAGTTAATTTAAAATGCAATTTAGTATAACGGCCGACATGAGAG	59	42	16	174.64	45	1	0							
N/A	GGGACACAATGGACGATTAGGTTTGTTACATCTAGCATTAGGCAATGAGAGGTAGTAACGGCCGACATGAGAG	73	42	16	174.64	48	1	0							
N/A	GGGACACAATGGACGTTTGAAACCAAGATACGCAACATGTAAGCTCAATGTATGTAACGGCCGACATGAGAG	72	42	16	174.64	47	1	0	16	24	164.38	14	1	0	0.9413
N/A	GGGACACAATGGACGCCAAAGTCGATCATACCTATAAATAATACTATGGGCAGGATAACGGCCGACATGAGAG	73	51	15	163.73	53	1	0							
N/A	GGGACACAATGGACGTTAATAATCATAAAAATGCTGCAATTATTGCTGTAAAGAATAACGGCCGACATGAGAG	73	51	15	163.73	51	1	0							
N/A	GGGACACAATGGACGTATTCTAAGATTTAGTGTATTTAAAACTAACTAAGTAACGGCCGACATGAGAG	68	51	15	163.73	57	1	0							
N/A	GGGACACAATGGACGGACGGAGAGCTTACTTATTATTCTATGGGCAAGTTTCACATAACGGCCGACATGAGAG	73	51	15	163.73	54	1	0							
N/A	GGGACACAATGGACGAATAACAAAGTTTGACGGTTAGCTAAGCCAGAAATGCGCATAACGGCCGACATGAGAG	73	51	15	163.73	56	1	0							

N/A	GGGACACAATGGACGACAATATGAGAACCGGAGTTGTTACTGCAATGTTTGTTCGTAACGGCCGACATGAGAG	73	51	15	163.73	59	1	0							
N/A	GGGACACAATGGACGAAGATTGGCTGCAAGGTACGATTAACGAATCACGTATTACTAACGGCCGACATGAGAG	73	51	15	163.73	55	1	0							
N/A	GGGACACAATGGACGTTTCGGTACGGCTACGGAATGTATGCTTTATTTATTGACCTAACGGCCGACATGAGAG	73	51	15	163.73	58	1	0							
N/A	GGGACACAATGGACGAAAGAAACTAACAAAAACGGTCAGCATGTTTGCATAATATGTAACGGCCGACATGAGAG	74	51	15	163.73	52	1	0							
N/A	GGGACACAATGGACGCCAAAAATATAATGGGGGTTTTATAAAGGACTTCACGTTAACGGCCGACATGAGAG	71	51	15	163.73	50	1	0							
N/A	GGACACAATG	10	51	15	163.73	3	4	4	756	7	47.94	1	12	4	0.2928
N/A	GGGACACA	8	51	15	163.73	3	5	6	7	28	191.78	1	3	6	1.1713
N/A	GGGACACAATGG	12	51	15	163.73	3	3	2	6	29	198.62	1	2	2	1.2131
N/A	GGGACACAATGGACGGAATAGTTACTTTTAAGGTAATGAGTATATATGTAAATCATAACGGCCGACATGAGAG	73	64	14	152.81	60	1	0							
N/A	GGGACACAATGGACGTATAAAGTACAATCAAAACACGAATGGGATGATAGCATTATAACGGCCGACATGAGAG	73	64	14	152.81	67	1	0							
N/A	GGGACACAATGGACGTGGTATCACATCATGAGGCGTAAATGAATGAATTAATTACTAACGGCCGACATGAGAG	73	64	14	152.81	70	1	0							
N/A	GGGACACAATGGACGATGTTTTGTCTGTAGGGTGACATAGGTTGGTAATTAGCCTTAACGGCCGACATGAGAG	73	64	14	152.81	72	1	0							
N/A	GGGACACAATGGACGTTTAGGACTTCAAACTAATTTGTACTAAAGAAAAGTGGGCTAACGGCCGACATGAGAG	73	64	14	152.81	64	1	0							
N/A	GGGACACAATGGACGTTTATATTTCATATGGAACCTTGAGCCGATGTCTAGAACATAACGGCCGACATGAGAG	73	64	14	152.81	62	1	0							
N/A	GGGACACAATGGACGATCTTATTAATACTCGTAGACATTCACCGAAGATGAGGCGTAACGGCCGACATGAGAG	73	64	14	152.81	63	1	0							
N/A	GGGACACAATGGACGCTAAACTATTGATCATCTATACTAAGGTGATGATATGTAACGGCCGACATGAGAG	70	64	14	152.81	73	1	0							
N/A	GGGACACAATGGACGTATGTTATAATTACTCCTGATGATAGCCGGGTAACGGCCGACATGAGAG	64	64	14	152.81	65	1	0							
N/A	GGGACACAATGGACGTTTTTGATAATACGGCTGATCTAACTGCGAAGTAAGACGGTAACGGCCGACATGAGAG	73	64	14	152.81	61	1	0							
N/A	GGGACACAATGGACGTACAACACTGTTCTATAATAATCCCGAAGTGTTTTTGGTCTTAACGGCCGACATGAGAG	74	64	14	152.81	71	1	0							
N/A	GGGACACAATGGACGCTATAGAATATACTGTCTTACGTGAAGTGTAACGGCCGACATGAGAG	62	64	14	152.81	68	1	0							
N/A	GGGACACAATGGACGGAACAGCTTTATGACATATTTTAAATGTGACGAACTGTACTAACGGCCGACATGAGAG	73	64	14	152.81	69	1	0							
N/A	GGGACACAATGGACGATCTATTTTGAATGAGACAATATGTGGCGAATTATGTCCATAACGGCCGACATGAGAG	73	64	14	152.81	66	1	0							
N/A	GGGACACAATGGACGGAAATGAGTTAAGGTTTATAACGGCCGACATGAGAG	51	78	13	141.9	102	1	0							
N/A	GGGACACAATGGACGGGTAATTAGAGCGATTAAGTATCAATTTCATGATGAAATCTAACGGCCGACATGAGAG	73	78	13	141.9	98	1	0							
N/A	GGGACACAATGGACGTTAATGGAGAAATGAATATCCGTAAAATAACGGCCGACATGAGAG	60	78	13	141.9	80	1	0							
N/A	GGGACACAATGGACGGAGAAGGTTATAGAGTATTATTATCAATATAGTGTACACATAACGGCCGACATGAGAG	73	78	13	141.9	94	1	0							
N/A	GGGACACAATGGACGTTTAGTGAGTAGATTATACTTCAATCAAAATGCGCTAACGGCCGACATGAGAG	68	78	13	141.9	89	1	0							
N/A	GGGACACAATGGACGGTATTTAATGATTTCTGGAGGCAATGGACCTTAAGGGATCTAACGGCCGACATGAGAG	73	78	13	141.9	95	1	0							
N/A	GGGACACAATGGACGATGATTCTATGTACCCATGACGGTGATAACGGCCGACATGAGAG	59	78	13	141.9	76	1	0							
N/A	GGGACACAATGGACGGTTATAACTAAACTATAGCACACTAGGATTTATGTTAGGTTAACGGCCGACATGAGAG	73	78	13	141.9	91	1	0							
N/A	GGGACACAATGGACGAGTCTCACTAAGCATGTAAGCATTATCAACATTAAGAAGGTAACGGCCGACATGAGAG	73	78	13	141.9	84	1	0							
N/A	GGGACACAATGGACGGTAAAGTATTGTACTTATTCAGAAGGAACGAAGCATTATATAACGGCCGACATGAGAG	73	78	13	141.9	96	1	0							
N/A	GGGACACAATGGACGACCAAGATACTTATTTATATGTAACGGCCGACATGAGAG	54	78	13	141.9	77	1	0							
N/A	GGGACACAATGGACGACATCTACGGTCATAGGAGTAAAAATCTAACGGTTTGCACTAACGGCCGACATGAGAG	73	78	13	141.9	88	1	0							
N/A	GGGACACAATGGACGATTGATTATGAACATGCACACAGGATTGTGGGTTTTCGCATAACGGCCGACATGAGAG	73	78	13	141.9	97	1	0							
N/A	GGGACACAATGGACGAGCTGCCAAAGGAAGGAGTTATATATATTCCTATTCAATTAACGGCCGACATGAGAG	72	78	13	141.9	99	1	0							
N/A	GGGACACAATGGACGCTGATAATCTACATGTGAGCTTTACAACAGGTGAGGATTAACGGCCGACATGAGAG	71	78	13	141.9	86	1	0							
N/A	GGGACACAATGGACGAAAATACAATCGGAATATCACCATTCTCTTAATGTCAGGTAACGGCCGACATGAGAG	72	78	13	141.9	87	1	0							
N/A	GGGACACAATGGACGCTGTTAAGCTGATTATATTCTACTTTTACAAGCAGAACGCTAACGGCCGACATGAGAG	73	78	13	141.9	90	1	0							
N/A	GGGACACAATGGACGGATACGTAAATACACAGGTGAGACAAGCTGAGTGAAATGCTAACGGCCGACATGAGAG	73	78	13	141.9	75	1	0							
N/A	GGGACACAATGGACGTGACTTACGAAGCTGAAGTATTTAGAGTCAGAACGCAACTTAACGGCCGACATGAGAG	73	78	13	141.9	83	1	0							
N/A	GGGACACAATGGACGCTAATTAGTAACCGTATAAACTATCAAACGTATGGGTTAGTAACGGCCGACATGAGAG	73	78	13	141.9	78	1	0							
N/A	GGGACACAATGGACGTATCATAGTTTTATTTAGATAACGGCCGACATGAGAG	52	78	13	141.9	81	1	0							
N/A	GGGACACAATGGACGGTTGGGCTGAGCATTAAAGTACTAATATTACTGATAGCATAACGGCCGACATGAGAG	72	78	13	141.9	74	1	0							
N/A	GGGACACAATGGACGCTAATATACGAATGTTAAACTTGCATTTAACGTTGCGAACTAACGGCCGACATGAGAG	73	78	13	141.9	101	1	0							
N/A	GGGACACAATGGACGAAGGATTTTGACAGGATTCATTTGTGGGTCTTAGTGCGGCTAACGGCCGACATGAGAG	73	78	13	141.9	82	1	0							
N/A	GGGACACAATGGACGCATATTTATGAATTCAGGAAGATTACAAACCTGTTAGAAATAACGGCCGACATGAGAG	73	78	13	141.9	85	1	0							
N/A	GGGACACAATGGACGGCAGAAATATAAAATAAAGAATGATTACGGCAATTTAACTTAACGGCCGACATGAGAG	73	78	13	141.9	103	1	0							
N/A	GGGACACAATGGACGAGAAAAGTTGGACTCGAATTTTTGTATAACCGCATGTCGAGTAACGGCCGACATGAGAG	74	78	13	141.9	100	1	0							
N/A	GGGACACAATGGACGATACTTGTAAGTAAGGAATTATGAACTGATGTTGAGCTTTAACGGCCGACATGAGAG	72	78	13	141.9	79	1	0							
N/A	GGGACACAATGGACGATTGAATTAAATGTTTAACGGCCGACATGAGAG	48	78	13	141.9	104	1	0							
N/A	GGGACACAATGGACGATTTACTCATTCAATTGATGGACTTATAGTGTGGGCAATGTAACGGCCGACATGAGAG	73	78	13	141.9	93	1	0	995	6	41.09	1192	1	0	0.2896
N/A	GGGACAC	7	78	13	141.9	92	1	0	103	15	102.74	98	1	0	0.724
N/A	GGGACACAATGGACG	15	78	13	141.9	3	7	1	86	16	109.59	1	8	1	0.7723
N/A	GGGACACAATGGA	13	78	13	141.9	3	6	1	42	19	130.13	1	7	1	0.9171
N/A	GGGACACAATG	11	78	13	141.9	3	8	3	13	25	171.23	1	5	3	1.2067
N/A	GGGACACAATGGACGCATAACAAATGTTGGACTGTTTAGTCATGGCACAATATATTAACGGCCGACATGAGAG	73	112	12	130.98	115	1	0							
N/A	GGGACACAATGGACGTATAGTATTAAGCATATAATGGTTAATGGCTGTATTATACTAACGGCCGACATGAGAG	73	112	12	130.98	122	1	0							
N/A	GGGACACAATGGACGCCGTTTAGTTTAGTTTTGAAATAAGCTTACAATGCTGAGGTAACGGCCGACATGAGAG	73	112	12	130.98	117	1	0							
N/A	GGGACACAATGGACGGTGATTTGAATTTAACGGTAGATAATAATCGTACGCTAGATAACGGCCGACATGAGAG	73	112	12	130.98	109	1	0							

N/A	GGGACACAATGGACGTAAGGCTAGTATTGAAATAAACCAGGAATGGATAAAGATGTAACGGCCGACATGAGAG	73	112	12	130.98	112	1	0							
N/A	GGGACACAATGGACGTTGGTTTGATATATGAGTAGTTTGGCGAAAGCGACTGGTATAACGGCCGACATGAGAG	73	112	12	130.98	114	1	0							
N/A	GGGACACAATGGACGGTCTAGAATACACGTAATTCAACATTACGGATGCATGGGATAACGGCCGACATGAGAG	73	112	12	130.98	107	1	0							
N/A	GGGACACAATGGACGGTATTCGGATTTAACTAAAGAACTAAACATATAGTTTTTGTAACGGCCGACATGAGAG	73	112	12	130.98	120	1	0							
N/A	GGGACACAATGGACGGTATAGGTAAAGGTTTTATGTATTAGTAGAACCCGGATTCTAACGGCCGACATGAGAG	73	112	12	130.98	118	1	0							
N/A	GGGACACAATGGACGTAAAAATAAATGACCCTACAGGCAAAAGTTAACGAGCAACTAACGGCCGACATGAGAG	73	112	12	130.98	111	1	0							
N/A	GGGACACAATGGACGTACTACCAACAACTATCCAAATGAGATGTAATCTTGAAGGTAACGGCCGACATGAGAG	73	112	12	130.98	106	1	0							
N/A	GGGACACAATGGACGGTTGACTAATGAATAATCTTACTGGTTATTTTCACTGGGATAACGGCCGACATGAGAG	73	112	12	130.98	124	1	0							
N/A	GGGACACAATGGACGTTTCATTCATTATAACCTATGGCACTTTCGGTAGGACCGGTAACGGCCGACATGAGAG	73	112	12	130.98	121	1	0							
N/A	GGGACACAATGGACGTTAATGTATTTTTAACGGCCGACATGAGAG	45	112	12	130.98	7	2	5							
N/A	GGGACACAATGGACGTTGGGGTAAAATAATATAACCACGTTATTCGATGTACGAATAACGGCCGACATGAGAG	73	112	12	130.98	125	1	0							
N/A	GGGACACAATGGACGTTTGAAATGACTTGGATTATATACATACCAGCAGGGTTTTAACGGCCGACATGAGAG	72	112	12	130.98	110	1	0							
N/A	GGGACACAATGGACGCCTGGAAAATCTGAATGCAGGACGATATGACGTTTACTGATAACGGCCGACATGAGAG	73	112	12	130.98	108	1	0							
N/A	GGGACACAATGGACGGTGATGTTATTCATATAATTTAGAAATAGATGTATGGGGTTAACGGCCGACATGAGAG	73	112	12	130.98	123	1	0							
N/A	GGGACACAATGGACGTAAATAGTTAACTAAGGATATTATAACGGCCGACATGAGAG	56	112	12	130.98	116	1	0							
N/A	GGGACACAATGGACGCATCAGGATGTTACTTAATTTGTTTATAGGTAACTGGTTCTAACGGCCGACATGAGAG	73	112	12	130.98	113	1	0							
N/A	GGGACACAATGGACGTATTCAATTTTTAGGAATATAAGCAACAATGTAGGGAGGCTAACGGCCGACATGAGAG	73	112	12	130.98	119	1	0							
N/A	GGGACACAATGGACGAAGATTTACATGATATTCGAAGATTAGAACCTCATTGGCTTAACGGCCGACATGAGAG	73	112	12	130.98	105	1	0	756	7	47.94	787	1	0	0.366
N/A	GGGACACAATGGACGTATTTATATCAATTGTAGAGGTAGATAGTGGTTAAACGCTAACGGCCGACATGAGAG	72	134	11	120.07	154	1	0							
N/A	GGGACACAATGGACGCAAACTTTAATATAATAGAGAATAACGGCCGACATGAGAG	55	134	11	120.07	153	1	0							
N/A	GGGACACAATGGACGGTCGGGCAAATTATTAGCACATTTCTGATGAGCTTTTTGATAACGGCCGACATGAGAG	73	134	11	120.07	132	1	0							
N/A	GGGACACAATGGACGAAGGCATATCAAAATAATAATAAGATAAATAGTGTTGGGCTAACGGCCGACATGAGAG	73	134	11	120.07	129	1	0							
N/A	GGGACACAATGGACGTTATAGATTAGTGGTCGCTATTAATGTTTCACAGATTTGATAACGGCCGACATGAGAG	73	134	11	120.07	161	1	0							
N/A	GGGACACAATGGACGGACTACCAATAAAGTTCATCTATCGTGTTTTTTGCGGCTTAACGGCCGACATGAGAG	72	134	11	120.07	147	1	0							
N/A	GGGACACAATGGACGGACACATTATCGTATATGTATTTGAGTGTATTATGGATGATAACGGCCGACATGAGAG	73	134	11	120.07	136	1	0							
N/A	GGGACACAATGGACGTGAATTATATCATTCGAAAGATGTATATCGAGCGAATAGCTAACGGCCGACATGAGAG	73	134	11	120.07	137	1	0							
N/A	GGGACACAATGGACGTGAATGGAACGAATTACCTATATAATGGAAAAAGTTTGACTAACGGCCGACATGAGAG	73	134	11	120.07	131	1	0							
N/A	GGGACACAATGGACGTAGAGGCTAATCTAAATGTAAGAAGAAGAGTTTAAATGTCTAACGGCCGACATGAGAG	73	134	11	120.07	135	1	0							
N/A	GGGACACAATGGACGCTGTATGTAAGGATTTGAACGTATAATGGCTGCATAGTTATAACGGCCGACATGAGAG	73	134	11	120.07	134	1	0							
N/A	GGGACACAATGGACGATGAAAAAGTCGGCAGTATGTATTAATTACCTAGCTGACATAACGGCCGACATGAGAG	73	134	11	120.07	149	1	0							
N/A	GGGACACAATGGACGTTAAGATTATTACAAATAAGACCAGCACATATCGTGCGACTAACGGCCGACATGAGAG	73	134	11	120.07	156	1	0							
N/A	GGGACACAATGGACGTTTACAATAATGAACAATTTATGTTATGACAGTTGGAGAATAACGGCCGACATGAGAG	73	134	11	120.07	152	1	0							
N/A	GGGACACAATGGACGCATTAACTTGGTAAATAATAGGATACTGCACTGTCGAGCTTAACGGCCGACATGAGAG	73	134	11	120.07	144	1	0							
N/A	GGGACACAATGGACGGCATTTACTATAGAAAAGACACTGATTGCTGGTCGGACTATAACGGCCGACATGAGAG	73	134	11	120.07	146	1	0							
N/A	GGGACACAATGGACGTACAGTATCACTTGCGAAATTATAACGCATTGAAGTATTATAACGGCCGACATGAGAG	73	134	11	120.07	141	1	0							
N/A	GGGACACAATGGACGAGTTTGGAGGCCGGATTTGTAACATAGTATAGTTATAAAGTAACGGCCGACATGAGAG	73	134	11	120.07	143	1	0							
N/A	GGGACACAATGGACGATAATTTACAATGTTACCCAAACTGACGGCTAATTTAGGATAACGGCCGACATGAGAG	73	134	11	120.07	151	1	0							
N/A	GGGACACAATGGACGATCAAAGTAACACACGATTTTTCAGAGAGAACCTAAGGCTTAACGGCCGACATGAGAG	73	134	11	120.07	128	1	0							
N/A	GGGACACAATGGACGAGGAATTGACATTATATTGATCCAGGCTGACTACACAAGTAACGGCCGACATGAGAG	72	134	11	120.07	140	1	0							
N/A	GGGACACAATGGACGTAATAGAAGGTAAACACACAGCTGAGTTGTGATGTATCCTTAACGGCCGACATGAGAG	73	134	11	120.07	155	1	0							
N/A	GGGACACAATGGACGACGATGTAGAGTATTAACAATATCACTAGGGTTTGGGCCTTAACGGCCGACATGAGAG	73	134	11	120.07	145	1	0							
N/A	GGGTCTTGTTTCTTCTCTGTGTGTACAGGTGAACGTTAGATTTAGCAATCGACCGACACCCTACTCGTCACAACTCA	81	134	11	120.07	158	1	0							
N/A	GGGACACAATGGACGGTATAAAACTATGAAGTAATCAAAATTCTTGGATATTAGTCTAACGGCCGACATGAGAG	74	134	11	120.07	162	1	0							
N/A	GGGACACAATGGACGAGTATTAGATCAAAATTCATAATGTATTCCGGATGGCGGATAACGGCCGACATGAGAG	73	134	11	120.07	159	1	0							
N/A	GGGACACAATGGACGCGCCTAAAGCTATTTATGCTAACGGCCGACATGAGAG	52	134	11	120.07	163	1	0							
N/A	GACACAATGGACGCACCTAGAGGAACTTAGAATATTAAGGAAACGCTAACGGCCGACATGAGAG	64	134	11	120.07	127	1	0							
N/A	GGGACACAATGGACGTATAGTAACATGAGTGATTGATACAGAAAACTTTAGGACTAACGGCCGACATGAGAG	72	134	11	120.07	126	1	0							
N/A	GGGACACAATGGACGTTGACAATTATATACACATTATGTGCTAATACATAGGGGTTAACGGCCGACATGAGAG	73	134	11	120.07	142	1	0							
N/A	GGGACACAATGGACGGTATGGATGACGGAGGTTTTCTTTTTGGATAACTGTAGGGTAACGGCCGACATGAGAG	73	134	11	120.07	160	1	0							
N/A	GGGACACAATGGACGTAAATTTATTGAGAATGAAGGCTTTGATGGGTTGAATACCTAACGGCCGACATGAGAG	73	134	11	120.07	138	1	0							
N/A	GGGACACAATGGACGCCAGAGAAATCTTATATTCTGTATGAACTCAGGATTTTGAGTAACGGCCGACATGAGAG	74	134	11	120.07	133	1	0							
N/A	GGGACACAATGGACGAACAATGAACGGTATTCCGTATAGTCTTTCTTACAGTCTGTAACGGCCGACATGAGAG	73	134	11	120.07	139	1	0							
N/A	GGGACACAATGGACGGGGTACAAAGTTATTGAGTGAATGAAGGAGATACACCAGATAACGGCCGACATGAGAG	73	134	11	120.07	157	1	0							
N/A	GGGACACAATGGACGAAGTGACCTTCACATACACTAACAGTTGAGCCAACACACGTAACGGCCGACATGAGAG	73	134	11	120.07	130	1	0							
N/A	GGGACACAATGGACGTTAGGTCTGATGAACATCATCACAACACAATTAGTTGTACTAACGGCCGACATGAGAG	73	134	11	120.07	148	1	0							
N/A	GGGACACAATGGACGCGGGCATACCGGATATATCTAATTTGAACGATATAGAGTATAACGGCCGACATGAGAG	73	134	11	120.07	150	1	0							
N/A	GGGACACAAT	10	134	11	120.07	3	9	4	13	25	171.23	1	4	4	1.4261
N/A	GGGACACAATGGACGGTATTAAGGTTATACTTCGGCTAGTAAAATGTACGATGTTAACGGCCGACATGAGAG	72	173	10	109.15	191	1	0							
N/A	GGGACACAATGGACGAGTACCTCTGACGATTATTATATTAGATGGTACGAGCTTCTAACGGCCGACATGAGAG	73	173	10	109.15	170	1	0							
N/A	GGGACACAATGGACGGTTGATTCACGACGTAAAGATTTGTAACTCTGAGTTGTAATAACGGCCGACATGAGAG	73	173	10	109.15	214	1	0							

N/A	GGGACACAATGGACGCTTTCAACCGATAGCAAATGAAAACACAATGTTTATGGGTTAACGGCCGACATGAGAG	73	173	10	109.15	207	1	0							
N/A	GGGACACAATGGACGGTGCAGTGAGCAATTGAAGACTAGATAGGTTTTAATGCTATAACGGCCGACATGAGAG	73	173	10	109.15	201	1	0							
N/A	GGGACACAATGGACGTTAAAACATAATCATATACGAAAAATGCAGGCTATGCAGATAACGGCCGACATGAGAG	73	173	10	109.15	192	1	0							
N/A	GGGACACAATGGACGACCAACATAGATATAACTTGCACTATTAACGGCCGACATGAGAG	59	173	10	109.15	200	1	0							
N/A	GGGACACAATGGACGCTGTGGTGAGATAGTAAACAAGATGATTGATTAGATACGCTAACGGCCGACATGAGAG	73	173	10	109.15	165	1	0							
N/A	GGGACACAATGGACGTAAAGACGAACCAATAGTGAATAATAAATGTATGGCATCTTAACGGCCGACATGAGAG	73	173	10	109.15	172	1	0							
N/A	GGGACACAATGGACGATGGTATCTACAGCATAATTTAAATGACAAAAGCTGACATTAACGGCCGACATGAGAG	73	173	10	109.15	185	1	0							
N/A	GGGACACAATGGACGAGTATTCATTTTCAACCAATGTGTATACTAATGGTGGGTCTAACGGCCGACATGAGAG	73	173	10	109.15	216	1	0							
N/A	GGGACACAATGGACGAACCATGGTAACATATGATTGCAAGAATCAGTACCGGGACTAACGGCCGACATGAGAG	73	173	10	109.15	195	1	0							
N/A	GGGACACAATGGACGCACAACAATGCATCTGAAGACAAAGTATCCTACTGGCTTAACGGCCGACATGAGAG	71	173	10	109.15	174	1	0							
N/A	GGGACACAATGGACGTTTATTAAATGATAAAGAAAAACGATCAAACCACTGAGGCTAACGGCCGACATGAGAG	73	173	10	109.15	177	1	0							
N/A	GGGACACAATGGACGATTCAATAATCAGATCATCTAAGTACCTAACGACGGCTAGTAACGGCCGACATGAGAG	73	173	10	109.15	206	1	0							
N/A	GGGACACAATGGACGCGATAATATATGGAATCTATATACATAAAAAGATTATCAGTAACGGCCGACATGAGAG	73	173	10	109.15	171	1	0							
N/A	GGGACACAATGGACGTGATTTGATCGGATATAGTTTTAACGGCCGACATGAGAG	54	173	10	109.15	179	1	0							
N/A	GGGACACAATGGACGTTGGCAAGGTTATATATTTGAATGAGTAACGGCCGACATGAGAG	59	173	10	109.15	217	1	0							
N/A	GGGACACAATGGACGGAGAATTTAGTATATGGTGATTGATATTGTACCAGCGTAACGGCCGACATGAGAG	70	173	10	109.15	183	1	0							
N/A	GGGACACAATGGACGGTTTATACAAGAAATCTTTGATAACGGCCGACATGAGAG	54	173	10	109.15	188	1	0							
N/A	GGGACACAATGGACGTTGAGGGTTTAGTAGTAAACATATTACTAATTCAGGACTTAACGGCCGACATGAGAG	72	173	10	109.15	169	1	0							
N/A	GGGACACAATGGACGCTAGTAATTTTAGACTGATTTCGACATCAGACAGTACGGTCTAACGGCCGACATGAGAG	74	173	10	109.15	176	1	0							
N/A	GGGACACAATGGACGAGTTGCCGAAGAGTCGACGTATACTAATTACTTTATTAGCTAACGGCCGACATGAGAG	73	173	10	109.15	168	1	0							
N/A	GGGACACAATGGACGGTGCGGTTGATTAATCAAGGTAAATATATGCTTTGGAATCTAACGGCCGACATGAGAG	73	173	10	109.15	209	1	0							
N/A	GGGACACAATGGACGGATTTGAGATGTAATAATGAAGGATATCTAACGGCCGACATGAGAG	61	173	10	109.15	211	1	0							
N/A	GGGACACAATGGACGAATTAAACGCTTTACTATTGTAGATGCCAAATGTACGGTTTAACGGCCGACATGAGAG	73	173	10	109.15	187	1	0							
N/A	GGGACACAATGGACGATAGAACGACAAATATACTAGAAACATATAAAGATGTATCTAACGGCCGACATGAGAG	73	173	10	109.15	164	1	0							
N/A	GGGACACAATGGACGGTAATTTAAATCTTTTTGCGATTAGTTAACGGCCGACATGAGAG	59	173	10	109.15	208	1	0							
N/A	GGGACACAATGGACGCTGTTTAGGTGAGTAGAGAACATTAAGAATATACACCAGGTAACGGCCGACATGAGAG	73	173	10	109.15	193	1	0							
N/A	GGGACACAATGGACGGTCACATATTTATGAATTAGACAACTTGAAAAAAACATACGATATAACGGCCGACATGAGA	77	173	10	109.15	194	1	0							
N/A	GGGACACAATGGACGTTACTAGACAGATTTTGGAATATTGGATACTGCATGTAGGTAACGGCCGACATGAGAG	73	173	10	109.15	213	1	0							
N/A	GGGACACAATGGACGAGGCGTAATGTTTCTCATTGTAATATCAGTGTTTGGGCAATAACGGCCGACATGAGAG	73	173	10	109.15	180	1	0							
N/A	GGGACACAATGGACGTGAATCTCGGTTTGAATATGATGGAATAAGTAATGCATCGTAACGGCCGACATGAGAG	73	173	10	109.15	198	1	0							
N/A	GGGACACAATGGACGTTGAATAGGTTTAATTGATTGTTTAGATCATGGCACGGTATAACGGCCGACATGAGAG	73	173	10	109.15	166	1	0							
N/A	GGGACACAATGGACGATGCAAGTATTTACTGATGGATATATTTTCAGGAAGGCTTAACGGCCGACATGAGAG	72	173	10	109.15	178	1	0							
N/A	GGGACACAATGGACGCTAGAAATACTGGACTTTATTCTTATTGTCATGTATAATATAACGGCCGACATGAGAG	73	173	10	109.15	181	1	0							
N/A	GGGACACAATGGACGATTGAAATAGTTAAATCACCTAGTTAACAAATATTGGCAATAACGGCCGACATGAGAG	73	173	10	109.15	173	1	0							
N/A	GGGACACAATGGACGTAAGTATTTATATGCGCAGATTTAGTTTATGCATAATACCTAACGGCCGACATGAGAG	73	173	10	109.15	190	1	0							
N/A	GGGACACAATGGACGTTAGATCAATTACCACACTACGATGAAACTTGTAGGCTTCTAACGGCCGACATGAGAG	73	173	10	109.15	210	1	0							
N/A	GGGACACAATGGACGGTAAATACTTATTATACGGAACCGCAAGGGAAAGGAAGGTAACGGCCGACATGAGAG	72	173	10	109.15	189	1	0							
N/A	GGGACACAATGGACGATAAAGTAACTTAGTGGGACGACGTATAATTGAGGCGTAGTAACGGCCGACATGAGAG	73	173	10	109.15	215	1	0							
N/A	GGGACACAATGGACGCCGATAAATGAATTACGAATGCATAATGTTTGGACACTAGTAACGGCCGACATGAGAG	73	173	10	109.15	197	1	0							
N/A	GGGACACAATGGACGTTCGAAACCATCGAGAAGGCCACTATGAATTTTAAGTAGATAACGGCCGACATGAGAG	73	173	10	109.15	218	1	0							
N/A	GGGACACAATGGACGATTATTTGAAATTGAGTGATAACGGAAATCTGTTCGGGCTTAACGGCCGACATGAGAG	73	173	10	109.15	186	1	0							
N/A	GGGACACAATGGACGGTTAGAAGCTTAATTTCAACGCTAACGGTAAGTTAAATGCTAACGGCCGACATGAGAG	73	173	10	109.15	199	1	0							
N/A	GGGACACAATGGACGTAATATAAGTTGACATTAAACATATCAGTGCCGAGACTACTAACGGCCGACATGAGAG	73	173	10	109.15	202	1	0							
N/A	GGGACACAATGGACGCTGTAAAAGACATTAAGGATTTAATATGCTTAAGCAGGTATAACGGCCGACATGAGAG	73	173	10	109.15	182	1	0							
N/A	GGGACACAATGGACGGTGCTTACACTAGAAACTAGGCATATATTAAATTGCAAGCTAACGGCCGACATGAGAG	73	173	10	109.15	204	1	0							
N/A	GGGACACAATGGACGTACTAATTTGCCATGAAGATTCAAGTTTGAACCAGGCACATAACGGCCGACATGAGAG	73	173	10	109.15	203	1	0							
N/A	GGGACACAATGGACGGACTGGTATTATCTTAGATGTTTTTTTCTAGGCTATAACGGCCGACATGAGAG	68	173	10	109.15	205	1	0							
N/A	GGGACACAATGGACGGTAATAACGGAATTTTACAGCGATGAGCGCTTTCGGAAAATAACGGCCGACATGAGAG	73	173	10	109.15	167	1	0							
N/A	GGGACACAATGGACGTGAATACGGTTAATGAAAGCTGGATATAAAATGCTGTTATAACGGCCGACATGAGAG	72	173	10	109.15	212	1	0							
N/A	GGGACACAATGGACGTTTATCAATACAACTAGGTTTTAATGAATCCGAAATGATATAACGGCCGACATGAGAG	73	173	10	109.15	175	1	0							
N/A	GGGACACAATGGACGATTATAAAGACATATATGTTCTACGGTGAAGGCTGTGACATAACGGCCGACATGAGAG	73	173	10	109.15	196	1	0	568	8	54.79	693	1	0	0.502
N/A	GGACACAAT	9	173	10	109.15	3	10	5	418	9	61.64	1	10	5	0.5647
N/A	GGGACACAATGGACGTATGTTTAACGGTATGAATTGATTCCACATGTGAGGCATAACGGCCGACATGAGAG	71	173	10	109.15	184	1	0	324	10	68.49	386	1	0	0.6275
N/A	GGGACACAATGGACGCTATTCAATTCAATGAGCTTTCCCAGATTTGGAAGCTGTATAACGGCCGACATGAGAG	73	229	9	98.24	221	1	0							
N/A	GGGACACAATGGACGCTAGGTTTCTTGTAAAAAGTAATTGACACGATATACGGGCTAACGGCCGACATGAGAG	73	229	9	98.24	256	1	0							
N/A	GGGACACAATGGACGCCGGGGACGATGTTGACATATATTAGGTTGTTACTTTCACTAACGGCCGACATGAGAG	73	229	9	98.24	272	1	0							
N/A	GGGACACAATGGACGTACGAGGGGTACTAAAGTAGAAATACAATAGCTCCAAATGTAACGGCCGACATGAGAG	73	229	9	98.24	242	1	0							
N/A	GGGACACAATGGACGATTTTCATACACTGGTCGCAAGATATCATATTGGGCGATATAACGGCCGACATGAGAG	73	229	9	98.24	254	1	0							
N/A	GGGACACAATGGACGAAGGTCGGATCTACGGTTTGAACTAATACATTTCAAGCTTTAACGGCCGACATGAGAG	73	229	9	98.24	230	1	0							
N/A	GGGACACAATGGACGTAGAGCATAACTTACAGAAGGTATATAACTGACTGACGCTAACGGCCGACATGAGAG	72	229	9	98.24	265	1	0							

N/A	GGGACACAATGGACGGTCAATATAAACTTAGATTGCAAACATATTATCGTTTGGCTAACGGCCGACATGAGAG	73	229	9	98.24	285	1	0
N/A	GGGACACAATGGACGTATACTGAATTAATAAATTTTTGATAACGGCCGACATGAGAG	57	229	9	98.24	273	1	0
N/A	GGGACACAATGGACGTTAATTATGGAGTATGTGAAGATACCACTTGTTTATGCAATAACGGCCGACATGAGAG	73	229	9	98.24	262	1	0
N/A	GGGACACAATGGACGCTATTTTGAAATCCATGCAATGTAACTGGAGTGCGGGTCTTAACGGCCGACATGAGAG	73	229	9	98.24	280	1	0
N/A	GGGACACAATGGACGAAGTCGATCTACCAAGCATTATTATTTTGCCGGGTAACGGCCGACATGAGAG	67	229	9	98.24	271	1	0
N/A	GGGACACAATGGACGTTGAATAATAAATGTCGAGCAATCGAAAACCTAACAAGAATAACGGCCGACATGAGAG	73	229	9	98.24	259	1	0
N/A	GGGACACAATGGACGTAAACTAACGTGCTACTTTTTAAAAAAGCTCGAATGCCAGCATAACGGCCGACATGAGAG	75	229	9	98.24	250	1	0
N/A	GGGACACAATGGACGCCGATGCATGTCTACATAGAATGTGTATGAGACATATTAGTAACGGCCGACATGAGAG	73	229	9	98.24	274	1	0
N/A	GGGACACAATGGACGGAGACGTAAAGATTATGATAATCAAGGCCACTAGAATAGTAACGGCCGACATGAGAG	72	229	9	98.24	227	1	0
N/A	GGGACACAATGGACGTACGATTGAGATTAACGAAATATTCTATATCACAAGACTAACGGCCGACATGAGAG	71	229	9	98.24	287	1	0
N/A	GGGACACAATGGACGTTAATCTTGTAATCACCAAACGTGGACGTAATGTCTAGGCTAACGGCCGACATGAGAG	73	229	9	98.24	289	1	0
N/A	GGGACACAATGGACGTGAAAATCCCATCGGAAAGAATGTACAGGCTTGTAGATATTAACGGCCGACATGAGAG	73	229	9	98.24	257	1	0
N/A	GGGACACAATGGACGTATTACGTAGAGGTCAGTAATTGGTAAGCAAAATTCACCTATAACGGCCGACATGAGAG	74	229	9	98.24	237	1	0
N/A	GGGACACAATGGACGGAGAGAAAATAATTGAGAGCTTACAGGCTTCCATAAAACTTAACGGCCGACATGAGAG	73	229	9	98.24	282	1	0
N/A	GGGACACAATGGACGTGAACCAAAATGACCGACCGTATTTCTAGCTTGATTAATGTAACGGCCGACATGAGAG	73	229	9	98.24	226	1	0
N/A	GGGACACAATGGACGTTATTTGGTAATATAAGTTTATATAACGGCCGACATGAGAG	56	229	9	98.24	275	1	0
N/A	GGGACACAATGGACGACAAAAGATGAAAATTGAGTTATAAGGCTGGTACCGTATGTAACGGCCGACATGAGAG	73	229	9	98.24	240	1	0
N/A	GGGACACAATGGACGACGTGAACTGATATAATAGTACAGTATTTTGAGTACGCAGCTAACGGCCGACATGAGAG	74	229	9	98.24	268	1	0
N/A	GGGACACAATGGACGTAATCTATAACCCACAATGCAAGCCAGACACACAGAAATGTAACGGCCGACATGAGAG	73	229	9	98.24	278	1	0
N/A	GGGACACAATGGACGATGAAAACATGTACGAACCTTAAAGGCGCGGAACAATTTCTAACGGCCGACATGAGAG	73	229	9	98.24	260	1	0
N/A	GGGACACAATGGACGCTTTTAACTTTAAGACCGGGAAGGTTTTTCCAGGTATAAACTAACGGCCGACATGAGAG	74	229	9	98.24	248	1	0
N/A	GGGACACAATGGACGGTATGGTTCATATTGTTTCACCTATGTCTGGTAGAATACTTAACGGCCGACATGAGAG	73	229	9	98.24	233	1	0
N/A	GGGACACAATGGACGTATTAATCTACTAATGAAGGGTAAACTACATAAATCATTATAACGGCCGACATGAGAG	73	229	9	98.24	220	1	0
N/A	GGGACACAATGGACGCAAGGTAAAAAGCTTCTTGTTAGTGAACAGATGCATAGGCTAACGGCCGACATGAGAG	73	229	9	98.24	239	1	0
N/A	GGGACACAATGGACGACGTAAGTTATATTTTTGGCATAACGGCCGACATGAGAG	54	229	9	98.24	222	1	0
N/A	GGGACACAATGGACGCGTATGTTTAACGGCCGACATGAGAG	41	229	9	98.24	279	1	0
N/A	GGGACACAATGGACGTTAGATTAAATACGATTACAATACTATGAATGTATGATGCTAACGGCCGACATGAGAG	73	229	9	98.24	229	1	0
N/A	GGGACACAATGGACGACGAGTGATTTCTGTGGTTAGATTGAATATCCTGGCCAATTAACGGCCGACATGAGAG	73	229	9	98.24	253	1	0
N/A	GGGACACAATGGACGTTAAGGACATAATGTGATAAGGAATGTACGGTTTCGTGAATAACGGCCGACATGAGAG	73	229	9	98.24	269	1	0
N/A	GGGACACAATGGACGGAAATGTATACAAATGCTCTATATATAAGAGATCATAATGTAACGGCCGACATGAGAG	73	229	9	98.24	267	1	0
N/A	GGGACACAATGGACGCATGAAGTATATATAGATCTGAAGAAGAGTAGCATATCTAACGGCCGACATGAGAG	71	229	9	98.24	225	1	0
N/A	GGGACACAATGGACGTTAGTGCGAACTGATAGATCACTTATTATGCATGAAGTACTAACGGCCGACATGAGAG	73	229	9	98.24	294	1	0
N/A	GGGACACAATGGACGTTTAGCTATATAATGCATTAGGCAATGGTCTACTTAGACATAACGGCCGACATGAGAG	73	229	9	98.24	244	1	0
N/A	GGGACACAATGGACGCGAGTAATCAAAATGAAAATGGAAAGCTTAACGGCCGACATGAGAG	61	229	9	98.24	236	1	0
N/A	GGGACACAATGGACGAAGTATTTAGATAAGTTTACCAGACTGTCATCAGGTCGCTAACGGCCGACATGAGAG	72	229	9	98.24	296	1	0
N/A	GGGACACAATGGACGGGTAGTTAGATCTGTAAGTATTTTTTAAGGACATGGCGACTAACGGCCGACATGAGAG	73	229	9	98.24	281	1	0
N/A	GGGACACAATGGACGAGCAAAATCAATTTATTAACTTGATAACGGCCGACATGAGAG	57	229	9	98.24	255	1	0
N/A	GGGACACAATGGACGTTAAGATTATTTAGAGCCGTAAATGTATGGCGTCTGAGCATAACGGCCGACATGAGAG	73	229	9	98.24	290	1	0
N/A	GGGACACAATGGACGTTGGACGTAAGGATGGATAAGTTAATTAATAGCTACCATATAACGGCCGACATGAGAG	73	229	9	98.24	293	1	0
N/A	GGGACACAATGGACGCCTAGTTTATTTAAGGAAAAGGAGTTTAATCCTCACGAACTAACGGCCGACATGAGAG	73	229	9	98.24	228	1	0
N/A	GGGACACAATGGACGATTTCTGTATATGAAAGGCTGATAACAGTATTATGGATTGTAACGGCCGACATGAGAG	73	229	9	98.24	246	1	0
N/A	GGGACACAATGGACGCGACAGTGGATAATATATTCATGGTTAAAGGGATATACACTAACGGCCGACATGAGAG	73	229	9	98.24	249	1	0
N/A	GGGACACAATGGACGGTCTGGATCTATAATCATAAGAGATATAAAGCTTAGGAGGTAACGGCCGACATGAGAG	73	229	9	98.24	247	1	0
N/A	GGGACACAATGGACGTTTGATTAATATGAATCTTTTCTAACGGCCGACATGAGAG	55	229	9	98.24	266	1	0
N/A	GGGACACAATGGACGTATGTACTTCACAGTATTCAAATGAAACGGTGGAAATCTAACGGCCGACATGAGAG	71	229	9	98.24	292	1	0
N/A	GGGACACAATGGACGACTGTAGAATTATGGAAAACACTAACGGCCGACATGAGAG	55	229	9	98.24	286	1	0
N/A	GGGACACAATGGACGGATTTGTAACTATGAACACCTTTTAACTGGGTTCTATGATAACGGCCGACATGAGAG	72	229	9	98.24	263	1	0
N/A	GGGACACAATGGACGTTTTAAACAATATTGACTATGTAAACCGTAGTTTTCAATATAACGGCCGACATGAGAG	73	229	9	98.24	235	1	0
N/A	GGGACACAATGGACGCCGGACAAAAATTTTAAAAGTGTAAAGACTAAGTTATGCTTAACGGCCGACATGAGAG	73	229	9	98.24	251	1	0
N/A	GGGACACAATGGACGCACAATCACTAGAGAGCTTAGAAATGAGCCTTAGCAATTATAACGGCCGACATGAGAG	73	229	9	98.24	241	1	0
N/A	GGGACACAATGGACGAGGAATACAATATGTATCCTTCATTATGTGATGCGGCTTATAACGGCCGACATGAGAG	73	229	9	98.24	224	1	0
N/A	GGGACACAATGGACGTTAGCAAGCAAAAGACTATAGATAATACTTTAATACTAAGTAACGGCCGACATGAGAG	73	229	9	98.24	291	1	0
N/A	GGGACACAATGGACGGTTTGTAATACATGAGGGCTTACAGTGTTATCACTAACGATAACGGCCGACATGAGAG	73	229	9	98.24	238	1	0
N/A	GGGACACAATGGACGTAGACACTAAACACGTATTGTTACTTGAATGGACAGGCAATAACGGCCGACATGAGAG	73	229	9	98.24	270	1	0
N/A	GGGACACAATGGACGTTTTAGAGTTATGAATAATGAACTAAACCATGGCTTGCCATAACGGCCGACATGAGAG	73	229	9	98.24	288	1	0
N/A	GGGACACAATGGACGTATAACGAAACGGACAAAATCGGTAAGAGATTTTAACGCTAACGGCCGACATGAGAG	72	229	9	98.24	232	1	0
N/A	GGGACACAATGGACGTTAGAAAAGCTTGAATACATATATGTGAATAATCACAATCTAACGGCCGACATGAGAG	73	229	9	98.24	277	1	0
N/A	GGGACACAATGGACGCCTTAGAGCAGGAACATATTTAACATATAAGTTTACGAGCTAACGGCCGACATGAGAG	73	229	9	98.24	234	1	0
N/A	GGGACACAATGGACGTAATGACACTAAATTATCCAATCAAATGGAAGCTGTATGCTAACGGCCGACATGAGAG	73	229	9	98.24	283	1	0
N/A	GGGACACAATGGACGGTAAAGTGCGCTAACTATTATTTAGAATGAGATATATACGTAACGGCCGACATGAGAG	73	229	9	98.24	284	1	0

N/A	GGGACACAATGGACGAAAGGTAAACCAACGAACACCATCAGAATCGAGCTGTGATTAACGGCCGACATGAGAG	73	229	9	98.24	243	1	0							
N/A	GGGACACAATGGACGACTACTAATATAGATTATATAGCGATGAGGCTGGAATTATAACGGCCGACATGAGAG	72	229	9	98.24	231	1	0							
N/A	GGGACACAATGGACGGCATACGTAGGAAACTGAATAGAAAGAATATCAGGCAATGTAACGGCCGACATGAGAG	73	229	9	98.24	219	1	0							
N/A	GGGACACAATGGACGGTTTCAGTTATGTAACTTTTAGAACATAACGACAAAGACGTAACGGCCGACATGAGAG	73	229	9	98.24	245	1	0							
N/A	GGGACACAATGGACGCACTTTAGAACAATTCCATACTAATTGGTGAATTGGCATAACGGCCGACATGAGAG	71	229	9	98.24	264	1	0							
N/A	GGGACACAATGGACGGTTATAGGATATTATTACACTAACGGCCGACATGAGAG	53	229	9	98.24	252	1	0							
N/A	GGGACACAATGGACGGATAGTAATTGATCTTACTTCACCACGTATGTACTGGCTTTAACGGCCGACATGAGAG	73	229	9	98.24	276	1	0							
N/A	GGGACACAATGGACGTAATGAATATTGAAAACATGTGATCATATCGACTGTGGGATAACGGCCGACATGAGAG	73	229	9	98.24	295	1	0							
N/A	GGGACACAATGGACGCTTGTCATAAAACTATTGAGATATAGAGGATGGTTTGTGATAACGGCCGACATGAGAG	73	229	9	98.24	261	1	0							
N/A	GGGACACAATGGACGAGTAGAAAGATACGAGTTTATTATGAATGTACCCGGTGTATAACGGCCGACATGAGAG	73	229	9	98.24	223	1	0							
N/A	GGGACACAATGGACGGTTTAAGTTTGTCATGATCAATAAGTCGACAGAAACGGCATAACGGCCGACATGAGAG	73	229	9	98.24	258	1	0							
N/A	GGGACACAATGGACGG	16	229	9	98.24	3	11	2	263	11	75.34	1	9	2	0.7669
N/A	GGGACACAATGGACGAACGTTTTACATGACATTAGTACAGACACATCACAGGCTTAACGGCCGACATGAGAG	72	308	8	87.32	358	1	0							
N/A	GGGACACAATGGACGCAATCAATTAATGACATGTGAGATAAACTCAAGCAGCTGATAACGGCCGACATGAGAG	73	308	8	87.32	322	1	0							
N/A	GGGACACAATGGACGGAAAAACGGACTGCATCAGATACACATTTTAATGCGGCGATAACGGCCGACATGAGAG	73	308	8	87.32	383	1	0							
N/A	GGGACACAATGGACGTATATTAATTTTAGGCCAAATGCGGTTAGCTTAATGGCATTAACGGCCGACATGAGAG	73	308	8	87.32	400	1	0							
N/A	GGGACACAATGGACGATCGAACGTTATGATTTTTAAACCAATTGACTTTAGCACATAACGGCCGACATGAGAG	73	308	8	87.32	375	1	0							
N/A	GGGACACAATGGACGCCTCAGACAGAAATCTTATTTAACAGGAATCTTCAGTCAGTAACGGCCGACATGAGAG	73	308	8	87.32	369	1	0							
N/A	GGGACACAATGGACGTAAAATATGTAAGCCTATGTTTCGACTGTAAAGTTGGGCTAACGGCCGACATGAGAG	72	308	8	87.32	373	1	0							
N/A	GGGACACAATGGACGGTCGTACAATATATTAGATTATCTAACGGCCGACATGAGAG	56	308	8	87.32	398	1	0							
N/A	GGGACACAATGGACGCAATAACTCGAATATACAATAATTTTTATAAAGTCGTGAGTAACGGCCGACATGAGAG	73	308	8	87.32	318	1	0							
N/A	GGGACACAATGGACGTTGATGCAAACTTTTCAATGAGATTTCAAGCATAAGCATTAACGGCCGACATGAGAG	72	308	8	87.32	392	1	0							
N/A	GGGACACAATGGACGAAACCTTATGTTTTAAAAGACTCTATGTTAACCGGGTTGGTAACGGCCGACATGAGAG	73	308	8	87.32	348	1	0							
N/A	GGGACACAATGGACGAGCCTAATAGTAGGTTGAATTTATATTTGATTTAACACTGTAACGGCCGACATGAGAG	73	308	8	87.32	409	1	0							
N/A	GGGACACAATGGACGAAGAATTCGTTAATTAGAACAGCAATTCGGCATGTAGAACTAACGGCCGACATGAGAG	73	308	8	87.32	406	1	0							
N/A	GGGACACAATGGACGTATTGTAATAAAATATTGAAGACCACCTAAACGAGGCAAGTAACGGCCGACATGAGAG	73	308	8	87.32	303	1	0							
N/A	GGGACACAATGGACGCACAGAAACATATCATTATTATGCTAACGGCCGACATGAGAG	57	308	8	87.32	399	1	0							
N/A	GGGACACAATGGACGTAAAGATCTATATGTTACATTAATACAGAATGATTGGCGTAACGGCCGACATGAGAG	72	308	8	87.32	401	1	0							
N/A	GGGACACAATGGACGTATTCTAAACTAACCTAGAAGATTTGACTAGTATGTAGGATAACGGCCGACATGAGAG	73	308	8	87.32	327	1	0							
N/A	GGGACACAATGGACGCCGAGGCATTATGGCTAATTTATCTTTAGCAGGTTATACTAACGGCCGACATGAGAG	72	308	8	87.32	387	1	0							
N/A	GGGACACAATGGACGAAGTTAGGAACTTATTATCACCAATGGACTGCGTCTCATAACGGCCGACATGAGAG	71	308	8	87.32	329	1	0							
N/A	GGGACACAATGGACGAACAAATTTGAACATACGAACTATGCGGGCTACTTATTATTAACGGCCGACATGAGAG	73	308	8	87.32	372	1	0							
N/A	GGGACACAATGGACGAATGCAAATACATGAGTTTAAAAATGTTTCACCATGTCGTAACGGCCGACATGAGAG	72	308	8	87.32	305	1	0							
N/A	GGGACACAATGGACGCCCGAACTAATAGGAATCATTTAAGTTGAGGTCTACGGTTTAACGGCCGAGATGAGAG	73	308	8	87.32	376	1	0							
N/A	GGGACACAATGGACGTAAATGTAATAGGCTGGAGCTAAAATCTTTCCGATTGACGTAACGGCCGACATGAGAG	73	308	8	87.32	324	1	0							
N/A	GGGACACAATGGACGGATTCAGCAATGTAACTTAACTCTGACATAGAGTGAAAACTAACGGCCGACATGAGAG	73	308	8	87.32	379	1	0							
N/A	GGGACACAATGGACGGAGGTTTTATTTATGAAACAGCTTATCATTACTGGATAGGTAACGGCCGACATGAGAG	73	308	8	87.32	353	1	0							
N/A	GGGACACAATGGACGATGTAAATATTATTGTATGCGTATCAGACTGTCTGGGCTTTAACGGCCGACATGAGAG	73	308	8	87.32	319	1	0							
N/A	GGGACACAATGGACGCTTATAAATCCAAGATGAATACATACATCATTCTGGGACGTAACGGCCGACATGAGAG	73	308	8	87.32	297	1	0							
N/A	GGGACACAATGGACGTAAAGTGAGAAGATAGAAAACTGTATTCATTACACCACCTAACGGCCGACATGAGAG	72	308	8	87.32	307	1	0							
N/A	GGGACACAATGGACGGAAATAATGGATGTTTTTTCATAATAACGGCCGACATGAGAG	57	308	8	87.32	321	1	0							
N/A	GGGACACAATGGACGTGGACGCCGATATGTGATTAGATATTGTTTTTTAGTCTTCGTAACGGCCGACATGAGAG	74	308	8	87.32	302	1	0							
N/A	GGGACACAATGGACGTAAAGAAACACTACATTAATTGCGGACCTGGCTGATCAAATAACGGCCGACATGAGAG	73	308	8	87.32	337	1	0							
N/A	GGGACACAATGGACGAAGGGAATATGGAACTGACTTTATTTGACCAATGTTGGCGTAACGGCCGACATGAGAG	73	308	8	87.32	364	1	0							
N/A	GGGACACAATGGACGATAAAACTAACATTACATTGATAACTGTCACGCGGGCGGATAACGGCCGACATGAGAG	73	308	8	87.32	356	1	0							
N/A	GGGACACAATGGACGTTACATACAATTTCCACCAAAGACGGCATATAGCTTATGATAACGGCCGACATGAGAG	73	308	8	87.32	381	1	0							
N/A	GGGACACAATGGACGAACCATGCTGATAAACGTAACTTAACACGTTATTTGCTGGTAACGGCCGACATGAGAG	73	308	8	87.32	355	1	0							
N/A	GGGACACAATAGACGTTAAAATCGACAGTATACATATTACAACTGTAATTGATTGTAACGGCCGACATGAGAG	73	308	8	87.32	365	1	0							
N/A	GGGACACAATGGACGTAAAGAAGAATTTTTCAATGCTAAACACGAGAGATCTTGTTAACGGCCGACATGAGAG	73	308	8	87.32	349	1	0							
N/A	GGGACACAATGGACGCTATCGTGTTATATACAATGAATTACAAAGGTGTATGACATAACGGCCGACATGAGAG	73	308	8	87.32	347	1	0							
N/A	GGGACACAATGGACGTTGAATATCACACTCAATTCTTATAATACGGGGTGCGTAGTAACGGCCGACAGAGAG	72	308	8	87.32	360	1	0							
N/A	GGGACACAATGGACGCTGAAAGATGAAGAGGAAATGTAGTAAATTTTTTCCCGATGGCTAACGGCCGACATGAGA	76	308	8	87.32	341	1	0							
N/A	GGGACACAATGGACGATTGTAGGGCCGGGATGTATAGGTAATTAATAATTTATCATAACGGCCGACATGAGAG	73	308	8	87.32	320	1	0							
N/A	GGGACACAATGGACGCCTGATTATTATGGGAACTGGAAGATTTAGCCACCTATGTAACGGCCGACATGAGAG	72	308	8	87.32	394	1	0							
N/A	GGGACACAATGGACGTAAGATACTATTGATTAGGCTCTTAAAGAAGGCCACCGTATAACGGCCGACATGAGAG	73	308	8	87.32	326	1	0							
N/A	GGGACACAATGGACGAGATAGAATAATACAATATTATATAACGGCCGACATGAGAG	56	308	8	87.32	359	1	0							
N/A	GGGACACAATGGACGTAACCAACGCGATACTTGAATTATAACGAGAATGCGGAATTAACGGCCGACATGAGAG	73	308	8	87.32	328	1	0							
N/A	GGGACACAATGGACGCTGTGACGTAAGTGGTAATCATATATACAAACCATGACGCTTACGGCCGACATGAGAG	73	308	8	87.32	395	1	0							
N/A	GGGACACAATGGACGCTAGGATAGCAATATGTTGTAGATAAAGTAACGTAGGCTCTAACGGCCGACATGAGAG	73	308	8	87.32	310	1	0							
N/A	GGGACACAATGGACGGAACAATACTTACGCGATTAAGCAATATTACGCGGTGAACATAACGGCCGACATGAGAG	74	308	8	87.32	345	1	0							

N/A	GGGACACAATGGACGATCGAAAATCTAGTAGAAATTCACTAATTTGGTTATGAGATAACGGCCGACATGAGAG	73	308	8	87.32	317	1	0
N/A	GGGACACAATGGACGGTTTCTTTGCATAGGCATAATTTAATCTTTATGGAGACTGTAACGGCCGACATGAGAG	73	308	8	87.32	377	1	0
N/A	GGGACACAATGGACGGTCAGACTATAAACTGAGTAAATAACATGTAATAGTGAATAACGGCCGACATGAGAG	72	308	8	87.32	396	1	0
N/A	GGGACACAATGGACGTAAACAAAGATTGATATTAGCAATGGTATTAGTCGAACCGTAACGGCCGACATGAGAG	73	308	8	87.32	412	1	0
N/A	GGGACACAATGGACGAATGTCAATTATTAAGAATAATAGAGGAGGCCAACAACAATAACGGCCGACATGAGAG	73	308	8	87.32	346	1	0
N/A	GGGACACAATGGACGTTTAAACCAATGAACCATAGAAGACGGAATGAGAGCCGCATAACGGCCGACATGAGAG	73	308	8	87.32	312	1	0
N/A	GGGACACAATGGACGTGTAGGCATTACGTATGGGAGATAAACAGATTCCATAGAATAACGGCCGACATGAGAG	73	308	8	87.32	403	1	0
N/A	GGGACACAATGGACGGTACAATATTATACACTAAAGTAACGGCCGACATGAGAG	54	308	8	87.32	397	1	0
N/A	GGGACACAATGGACGGACTGAATAGTTATGTCGGTATTTTTTACTGATTGAGCTTAACGGCCGACATGAGAG	72	308	8	87.32	330	1	0
N/A	GGGACACAATGGACGATAGTTATATTAAACGAATGATTATTATAGTGAGGTAAACTAACGGCCGACATGAGAG	73	308	8	87.32	411	1	0
N/A	GGGACACAATGGACGCATCGTTATAATTGCGATATTACCCTACAACAGGCTTGAATAACGGCCGACATGAGAG	73	308	8	87.32	342	1	0
N/A	GGGACACAATGGACGGTAGTGAGGCTTACAGACAGGTATTTGTTTATGAAGTCTAACGGCCGACATGAGAG	71	308	8	87.32	402	1	0
N/A	GGGACACAATGGACGGAGACATTACAGTCTGGGATTTAAGTTAGAATCTGGGTTGTAACGGCCGACATGAGAG	73	308	8	87.32	362	1	0
N/A	GGGACACAATGGACGGTGTAGTTAGACAGGAAATTTGATGATGAGTCGCTTGACGTAACGGCCGACATGAGAG	73	308	8	87.32	357	1	0
N/A	GGGACACAATGGACGACTGAGTTTTGTTGAGAATATTGAGTTAACTACTATGGGGCTAACGGCCGACATGAGAG	74	308	8	87.32	374	1	0
N/A	GGGACACAATGGACGGTCACATATTTATGAATTAGACAACTTGAAAAAAAACATACGATATAACGGCCGACATGAG	78	308	8	87.32	194	2	1
N/A	GGGACACAATGGACGTATCGATTTTATCTGAGCGAGTGTTGTTCGAGTGAAGGCTTAACGGCCGACATGAGAG	73	308	8	87.32	389	1	0
N/A	GGGACACAATGGACGGAATAATTTAGCAGATATTTTATATAAGGTGAGGACTTAACGGCCGACATGAGAG	70	308	8	87.32	366	1	0
N/A	GGGACACAATGGACGTTGTTTCTAGTACATAAATATGCACGAATGACTGACGATATAACGGCCGACATGAGAG	73	308	8	87.32	385	1	0
N/A	GGGACACAATGGACGCATACTGATAGTAAATTATAAGGAAAAAGAGACTTATTCAAGTAACGGCCGACATGAGAG	75	308	8	87.32	304	1	0
N/A	GGGACACAATGGACGAATACATGCTATTGATGATCCAGCATAACGGTTTCAAGCGTAACGGCCGACATGAGAG	73	308	8	87.32	335	1	0
N/A	GGGACACAATGGACGTTGTAACGCTTAACAACTCACCAGAATTAGTTGGCTATCATAACGGCCGACATGAGAG	73	308	8	87.32	309	1	0
N/A	GGGACACAATGGACGCTAGTTTCGGAATTTAATTTGAAGCAGATATTCGAAGCGGTAACGGCCGACATGAGAG	73	308	8	87.32	380	1	0
N/A	GGGACACAATGGACGAATAAACATTAATGTATTAACGGCCGACATGAGAG	50	308	8	87.32	104	2	6
N/A	GGGACACAATGGACGGAGATGACTAATCACCAACAATGCGAATTGTATGTAATAGTAACGGCCGACATGAGAG	73	308	8	87.32	354	1	0
N/A	GGGACACAATGGACGTATGTCTCATACCTTATTAAATAGTGTAGGAATGTACATATAACGGCCGACATGAGAG	73	308	8	87.32	384	1	0
N/A	GGGACACAATGGACGCCAAACAATACATTACATGAACATAATCGGGCATGTTTGATAACGGCCGACATGAGAG	73	308	8	87.32	298	1	0
N/A	GGGACACAATGGACGTAATTCACTTAATCTATGGTACAGTAGTATATAACGGCCGACATGAGAG	64	308	8	87.32	407	1	0
N/A	GGGACACAATGGACGGTTTTCTGAAGAGTCAAGGAATATGCTTGTACAATACGGTAACGGCCGACATGAGAG	72	308	8	87.32	382	1	0
N/A	GGGACACAATGGACGCTTACCTACTGAAGTTTTTATATAGTGTAGGCTATAACGGCCGACATGAGAG	67	308	8	87.32	404	1	0
N/A	GGGACACAATGGACGGAAGTATTAAATATTGATCTAACACCGCAAGGAAGAATACTAACGGCCGACATGAGAG	73	308	8	87.32	314	1	0
N/A	GGGACACAATGGACGTATTAATGAACTTGTCAACCGAACTTAATGTTGTAACCGGTAACGGCCGACATGAGAG	73	308	8	87.32	370	1	0
N/A	GGGACACAATGGACGTGCAATTAATTGAAATGAAGGACAACTGTGCTGGCCATATTAACGGCCGACATGAGAG	73	308	8	87.32	405	1	0
N/A	GGGACACAATGGACGCATGATAATTAACACTTGAAGCAAAATATACATAGGTTATTAACGGCCGACATGAGAG	73	308	8	87.32	339	1	0
N/A	GGGACACAATGGACGCTGCTCTATCCAGGAACTTATTTTGAAGTAGGGAGGAGGTAACGGCCGACATGAGAG	72	308	8	87.32	408	1	0
N/A	GGGACACAATGGACGGACCGCACACTCTAGATATAAACACAATTATACATCTTGATAACGGCCGACATGAGAG	73	308	8	87.32	300	1	0
N/A	GGGACACAATGGACGTAATAGTGGAGAAACTGATGTACAGTTACAGCCGGGTTAATAACGGCCGACATGAGAG	73	308	8	87.32	363	1	0
N/A	GGGACACAATGGACGCCTTTATCTAAAACGCTATATTCTGATTGAGTATAAAACGTAACGGCCGACATGAGAG	73	308	8	87.32	306	1	0
N/A	GGGACACAATGGACGATAATGCTTATGAGTTTGTTTAGAGGCTCAGACTCACGAGTAACGGCCGACATGAGAG	73	308	8	87.32	351	1	0
N/A	GGGACACAATGGACGTTGAGCCTAACTGATAAATCATACATATAAATGATCTTAGTAACGGCCGACATGAGAG	73	308	8	87.32	371	1	0
N/A	GGGACACAATGGACGGATACGTTTCAATACTTACTTGTACTAACGGCCGACATGAGAG	58	308	8	87.32	378	1	0
N/A	GGGACACAATGGACGTGCTGTAAACTATAAGGAAATATGATTCGTACTAGTTAGCTAACGGCCGACATGAGAG	73	308	8	87.32	352	1	0
N/A	GGAACACAATGGACGCTTAGTTCTATTGAGTGCGAGCGGCTAAATAAGTTAGTAGTAACGGCCGACATGAGAG	73	308	8	87.32	343	1	0
N/A	GGGACACAATGGACGACAGAGTAGCTGTGTTTTACGGGCCGCGTATTTATTTCACGGTAACGGCCGACATGAGAG	75	308	8	87.32	311	1	0
N/A	GGGACACAATGGACGCTAGAGTTTTATCTCAACAATCAGCAATGAAAGGACGGTATAACGGCCGACATGAGAG	73	308	8	87.32	332	1	0
N/A	GGGACACAATGGACGCTTGGTGACAGTTGTAGATATAAGATGTTACAACCTTGATTAACGGCCGACATGAGAG	73	308	8	87.32	323	1	0
N/A	GGGACACAATGGACGATAGTTGGTTGCAGATATATAAACAGTTATCTAACGGCCGACATGAGAG	64	308	8	87.32	316	1	0
N/A	GGGACACAATGGACGGAAATACAGATATAGAACGCAATCAATGGTCGTTAGGACATAACGGCCGACATGAGAG	73	308	8	87.32	333	1	0
N/A	GGGACACAATGGACGTTATATCACATATGGTATCCGTTAGATATCTCTAATTTAGTAACGGCCGACATGAGAG	73	308	8	87.32	338	1	0
N/A	GGGACACAATGGACGATTATTCGGCTAGATACCAATTTGCAAAGTAGCTTCTGGTTAACGGCCGACATGAGCG	73	308	8	87.32	308	1	0
N/A	GGGACACAATGGACGGACCGCGAGTATACTATAGAGTATCAAATCATATAATGCTAACGGCCGACATGAGAG	72	308	8	87.32	301	1	0
N/A	GGGACACAATGGACGAGAAAACTATATTAATTCTAAGACATAGTCGAAGTGGGCTTAACGGCCGACATGAGAG	73	308	8	87.32	388	1	0
N/A	GGGACACAATGGACGTGAAATAAGAAGCATAGACCTAATAGTCACCAAGTGGGCTAACGGCCGACATGAGAG	72	308	8	87.32	393	1	0
N/A	GGGACACAATGGACGACCAATACCAATTTGCCATACCATTTAATAAAGTAATTGGTAACGGCCGACATGAGAG	73	308	8	87.32	368	1	0
N/A	GGGACACAATGGACGCTTAACAGGATTACTACATAATTAAAACTTTAGTTGAAGCCTAACGGCCGACATGAGAG	74	308	8	87.32	350	1	0
N/A	GGGACACAATGGACGTATAACAGTTGTCTGTTTAACCTGGTCGGTATTTTACTGATAACGGCCGACATGAGAG	73	308	8	87.32	336	1	0
N/A	GGGACACAATGGACGATGGAGGTGCTTATGAATATTTACAATGAATGATATGTTCTAACGGCCGACATGAGAG	73	308	8	87.32	313	1	0
N/A	GGGACACAATGGACGACAGAGTAGCAAGCAATGGTAATGTTTACTTTAACGGCCGACATGAGAG	64	308	8	87.32	390	1	0
N/A	GGGACACAATGGACGGTACTGGAGCTATAGTAACTAAAATCAACTAGAACCATAGTAACGGCCGACATGAGAG	73	308	8	87.32	391	1	0
N/A	GGGACACAATGGACGTTTCACTACGTGGAACATGATAATGGCTTGATAATTTAGCTAACGGCCGACATGAGAG	73	308	8	87.32	367	1	0

N/A	GGGACACAATGGACGATAAAACAATCAATCCACCAATGCTAATCTTAAGTCAGCTTAACGGCCGACATGAGAG	73	308	8	87.32	334	1	0							
N/A	GGGACACAATGGACGCTGGGTTACAGATGTCAGATATTAGACACCATGTATCATATAACGGCCGACATGAGAG	73	308	8	87.32	386	1	0							
N/A	GGGACACAATGGACGGTAGTTGGACACAAAGGAGTTTATTACATCTTGAATGAGCTAACGGCCGACATGAGAG	73	308	8	87.32	325	1	0							
N/A	GGGACACAATGGACGGACCTTATAAAAATACGCTTTATCAGGCAGTAGCAATTTATAACGGCCGACATGAGAG	73	308	8	87.32	344	1	0							
N/A	GGGACACAATGGACGAATGTAGGAATCTTTGTACTTAACTTACAATTAAACCACATAACGGCCGACATGAGAG	73	308	8	87.32	315	1	0							
N/A	GGGACACAATGGACGCACGTTGCATACGTAAAGGGATATTTCAACCTAACTACGCTTAACGGCCGACATGAGAG	74	308	8	87.32	331	1	0							
N/A	GGGACACAATGGACGATATTATGCAAGTAAGTAGTTTTAAATTACGATGACGTAACGGCCGACATGAGAG	70	308	8	87.32	340	1	0							
N/A	GGGACACAATGGACGAAACATGTAAACCACACAATTAGTATATCGTAAACGGCAGTAACGGCCGACATGAGAG	73	308	8	87.32	361	1	0							
N/A	GGGACACAATGGACGGAAACGATACTTTACAAAATAACAGTAACGGCCGACATGAGAG	58	308	8	87.32	299	1	0							
N/A	GGGACACAATGGACGAAAATTATTGTAAATTGTAGACGTATATAGTGGGGCTGGTAACGGCCGACATGAGAG	72	308	8	87.32	410	1	0	161	13	89.04	153	1	0	1.0197
N/A	GGGACACAATGGACGAACCAATTATGTAGGGTACGATTATAGGACTTTAGACTGGTAACGGCCGACATGAGAG	73	426	7	76.41	495	1	0							
N/A	GGGACACAATGGACGTGGCGGGTATGCATTTATTTATTAAGAACATAACGGCCGACATGAGAG	63	426	7	76.41	464	1	0							
N/A	GGGACACAATGGACGTCGTTAGGTAACATTCTGATAAATTACATGTCGAATTAACTAACGGCCGACATGAGAG	73	426	7	76.41	438	1	0							
N/A	GGGACACAATGGACGTGCTATAAAACAATGGAGCTTGTGAGAGCTAGCTGTTGTTATAACGGCCGACATGAGAG	74	426	7	76.41	419	1	0							
N/A	GGGACACAATGGACGTTAGAGTATGGGTAGATTATTGAACACATTTACAGATCTCTAACGGCCGACATGAGAG	73	426	7	76.41	456	1	0							
N/A	GGGACACAATGGACGTAACGTAAAATAAAATTCGATAACGGCCGACATGAGAG	53	426	7	76.41	546	1	0							
N/A	GGGACACAATGGACGTTCATTCATACCTTTGGCGTGAATCTGTATTTTAACGGTATAACGGCCGACATGAGAG	73	426	7	76.41	463	1	0							
N/A	GGGACACAATGGACGCGCTAGTTTAAACCACTTATAATAAACTTTGGTGGATGCATAACGGCCGACATGAGAG	73	426	7	76.41	545	1	0							
N/A	GGGACACAATGGACGAAAGGCCGAGTATACAAGATTTATTGGAAAGCTGTTCGCATAACGGCCGACATGAGAG	73	426	7	76.41	559	1	0							
N/A	GGGACACAATGGACGCTGAATTTTACTAAGACGAGGGATAGTTTTCTGGGACTAGTAACGGCCGACATGAGAG	73	426	7	76.41	499	1	0							
N/A	GGGACACAATGGACGCCAAGATGGAATATAAGTGATAATACTTACTTGATTGAATTAACGGCCGACATGAGAG	73	426	7	76.41	531	1	0							
N/A	GGGACACAATGGACGTATTTTAGATACTTCTACTGAACCTATAGAACGGTCGGTCTAACGGCCGACATGAGAG	73	426	7	76.41	529	1	0							
N/A	GGGACACAATGGACGTAAAAAGAAGTGTATGTATTAAACACTATACTACTATCTGTAACGGCCGACATGAGAG	73	426	7	76.41	431	1	0							
N/A	GGGACACAATGGACGTATTAGAATACTACTTCACATCAGGCGTTTGACGATTAGTTAACGGCCGACATGAGAG	73	426	7	76.41	482	1	0							
N/A	GGGACACAATGGACGTGTTGGGTTATTCAAAACGCAACGAAAAGCGTTTAGGCTAACGGCCGACATGAGAG	71	426	7	76.41	532	1	0							
N/A	GGGACACAATGGACGACTGAGATGCAGTATATCGGATATTAATAGATGTGAGGCTAACGGCCGACATGAGAG	72	426	7	76.41	496	1	0							
N/A	GGGACACAATGGACGGTAACGCTAAATAACACTAACGGCCGACATGAGAG	50	426	7	76.41	507	1	0							
N/A	GGGACACAATGGACGGTAGTAAAGGGGTTCCTATTTTATTTGAATCGAGGTGGCGTAACGGCCGACATGAGAG	73	426	7	76.41	565	1	0							
N/A	GGGACACAATGGACGTTTTGGGTAAGAATGATTGGCTCTTTATACAATTCACTGTTAACGGCCGACATGAGAG	73	426	7	76.41	520	1	0							
N/A	GGGACACAATGGACGGCTGGAACAAAAGATCATTTTTAACATGTTTCTTGAGTATAACGGCCGACATGAGAG	72	426	7	76.41	458	1	0							
N/A	GGGACACAATGGACGATTATGTAGGCCCGATTAACTATGATTTTATTTTGCGTAGTAACGGCCGACATGAGAG	73	426	7	76.41	460	1	0							
N/A	GGGACACAATGGACGTAAGGGCGATTAAGGTGTAAGAAGATATTATTCTACATGATAACGGCCGACATGAGAG	73	426	7	76.41	550	1	0							
N/A	GGGACACAATGGACGCAAATTAAATATAGTATTCGAGTGCCATCAATGAGGTCGATAACGGCCGACATGAGAG	73	426	7	76.41	440	1	0							
N/A	GGGACACAATGGACGTTATAGAATATAATAATTAAACAGTAACGGCCGACATGAGAG	57	426	7	76.41	554	1	0							
N/A	GGGACACAATGGACGTTAGTCGAGTTTTACTATGATTTCGAAAAGACACATCGGTTAACGGCCGACATGAGAG	73	426	7	76.41	445	1	0							
N/A	GGGACACAATGGACGTTAAAGCACATAAATCTTATAGATGACAAAGCTTGGATAGTAACGGCCGACATGAGAG	73	426	7	76.41	542	1	0							
N/A	GGGACACAATGGACGGAAACGTAGAACATTTACATCAAAATATATTTGGTAAGGCTAACGGCCGACATGAGAG	73	426	7	76.41	413	1	0							
N/A	GGGACACAATGGACGAATTAGTCTTTTAACCTTGGCTGTTCACCAGTTTGTACGGTAACGGCCGACATGAGAG	73	426	7	76.41	462	1	0							
N/A	GGGACACAATGGACGGGATTACCAAATCTGCACTAGATAAATCTATAAACTAGAGTAACGGCCGACATGAGAG	73	426	7	76.41	474	1	0							
N/A	GGGACACAATGGACGGAGTGTTACAAGAAACTGAGATTTAAAATATGAACTCAGTGTAACGGCCGACATGAGAG	74	426	7	76.41	498	1	0							
N/A	GGGACACAATGGACGGTAGAACTTGATGTCGTACGAAATGTAGTAGAAAGACGTAACGGCCGACATGAGAG	71	426	7	76.41	459	1	0							
N/A	GGGACACAATGGACGTTGCATAGCCAAGGTTTTTAACGGCCGACATGAGAG	51	426	7	76.41	480	1	0							
N/A	GGGACACAATGGACGCTAATAACTGATAGTAATCACTAACGGCCGACATGAGAG	54	426	7	76.41	441	1	0							
N/A	GGGACACAATGGACGTATGAAAGTGAAACAGAATCTGGGGCCAATAGAACAAGAGTAACGGCCGACATGAGAG	73	426	7	76.41	558	1	0							
N/A	GGGACACAATGGACGAGTAAATATGTATATTGAGAACCTGGTTGGACGAAATGGGTAACGGCCGACATGAGAG	73	426	7	76.41	447	1	0							
N/A	GGGACACAATGGACGATACATTATAAGTCAGTGTTGAACAAGGTCGCTATAGTATTAACGGCCGACATGAGAG	73	426	7	76.41	500	1	0							
N/A	GGGACACAATGGACGCTTTATGCTAACAACCAAAGATACTGGATTAGATGACGTAACGGCCGACATGAGAG	71	426	7	76.41	553	1	0							
N/A	GGGACACAATGGACGTTATGTATCTTGCATGAGTCGAAATGACTGTATGAAAGACTAACGGCCGACATGAGAG	73	426	7	76.41	541	1	0							
N/A	GGGACACAATGGACGTAGCATGTTTGGACGCACAGAATTTATTTCTTGTGAGGGCTAACGGCCGACATGAGAG	73	426	7	76.41	566	1	0							
N/A	GGGACACAATGGACGTTGTAATATTCCTTAAATGGAGTCTAGGGGCTAGTTTTAGTAACGGCCGACATGAGAG	73	426	7	76.41	479	1	0							
N/A	GGGACACAATGGACGTAGAAGAGGATTTTTTACAATGACGGAAACTTACCTTCGATAACGGCCGACATGAGAG	73	426	7	76.41	422	1	0							
N/A	GGGACACAATGGACGGTGATGTATGACAAAATGTAGTAACAGATTCGTTACACTGTAACGGCCGACATGAGAG	73	426	7	76.41	425	1	0							
N/A	GGGACACAATGGACGCTGTGCGCAATTCTGATAGATATATTATCCACTCGAAAGCTAACGGCCGACATGAGAG	73	426	7	76.41	556	1	0							
N/A	GGGACACAATGGACGAGTAGGAATGTACTTAACATGATGTCATAACTACAGGGCTTAACGGCCGACATGAGAG	73	426	7	76.41	525	1	0							
N/A	GGGACACAATGGACGGATCAATAAACCATAAGCAACAAATAGCTGAATTTGGATATAACGGCCGACATGAGAG	73	426	7	76.41	494	1	0							
N/A	GGGACACAATGGACGTGCTTTATAATACCCTACGTGGAACTATAAACATTCGGAGTAACGGCCGACATGAGAG	73	426	7	76.41	418	1	0							
N/A	GGGACACAATGGACGCACGTTAAACATTTAAAATTCGTTGGAATACCTGGGGTCTTAACGGCCGACATGAGAG	73	426	7	76.41	517	1	0							
N/A	GGGACACAATGGACGGTACAATAGCTTGATTCTCAGTTAAACGAAGGGATTGATAACGGCCGACATGAGAG	71	426	7	76.41	449	1	0							
N/A	GGGACACAATGGACGTAAGAGTTATCGACTATTCACAAATAATCGTATCGCTTGGTAACGGCCGACATGAGAG	73	426	7	76.41	483	1	0							
N/A	GGGACACAATGGACGTTTTGTAAAATTTGAACCTAACGGCCGACATGAGAG	51	426	7	76.41	457	1	0							

N/A	GGGACACAATGGACGCTAAATGTTGAGACCGGTTTATATAAAGTATTTCATTTATAACGGCCGACATGAGAG	72	426	7	76.41	506	1	0
N/A	GGACACAATGGACGATCAGTCGGGCACTGATGCATAAGGCGTATTTGTTTTATGTAACGGCCGACATGAGAG	72	426	7	76.41	511	1	0
N/A	GGGACACAATGGACGTCGTTAAAGAGATATAGTTTTGGAATTGTCAACACTGACTAACGGCCGACATGAGAG	72	426	7	76.41	526	1	0
N/A	GGGACACAATGGACGTTGACAGATTGAAATGTGAAATACAAAACCAGAACCTGTATAACGGCCGACATGAGAG	73	426	7	76.41	564	1	0
N/A	GGGACACAATGGACGCAATGGCCAATTAATGTACCTATAAAAAGTAATGTGGGAGTAACGGCCGACATGAGAG	73	426	7	76.41	443	1	0
N/A	GGGACACAATGGACGGTACAGTGATGAACTAACGTCATAATAGATACATGAGGGCTAACGGCCGACATGAGAG	73	426	7	76.41	521	1	0
N/A	GGGACACAATGGACGAAGTACCTTGATTTTTATATACAATTCCATGGGGACGAGATAACGGCCGACATGAGAG	73	426	7	76.41	472	1	0
N/A	GGGACACAATGGACGACAGATTAATGACAGATCTATTTGTTCAACATGGTTGGCTTAACGGCCGACATGAGAG	73	426	7	76.41	516	1	0
N/A	GGGACACAATGGACGTTTTAATTAGCATCGTGAGCGCACAATTAGGCTGAATAGATAACGGCCGACATGAGAG	73	426	7	76.41	522	1	0
N/A	GGGACACAATGGACGAATACTGTTGTTATATATTTATGTGGGACTGGTTAGACGATAACGGCCGACATGAGAG	73	426	7	76.41	568	1	0
N/A	GGGACACAATGGACGCTTAAATAGACGATAGTTTAAATCACAAATGAATGCTAGTTAACGGCCGACATGAGAG	73	426	7	76.41	487	1	0
N/A	GGGACACAATGGACGCAATCAAATCATAACCAAACCATAGCGGTAATTACTGTAGTAACGGCCGACATGAGAG	73	426	7	76.41	455	1	0
N/A	GGGACACAATGGACGAATCTACCATTTGATTGGCTTTATGGGACTAACAATACAATAACGGCCGACATGAGAG	73	426	7	76.41	538	1	0
N/A	GGGACACAATGGACGCAATTATTGATATATTAATGACAAGTGGCTTATGGCTGGCTAACGGCCGACATGAGAG	73	426	7	76.41	560	1	0
N/A	GGGACACAATGGACGGAACGTAAAGATATGTATACGGGAAACTTAATATATACCATAACGGCCGACATGAGAG	73	426	7	76.41	515	1	0
N/A	GGGACACAATGGACGTTGATGAAATAGTATTGTAAAACGTAACTGATGCGGGCCTAACGGCCGACATGAGAG	72	426	7	76.41	489	1	0
N/A	GGGACACAATGGACGGATCAAAGATTGGTCGCCACATAGTATATTGGCACAAACTAACGGCCGACATGAGAG	72	426	7	76.41	476	1	0
N/A	GGGACACAATGGACGATACGAACTAAATGGAATTATAAAAATAGCCCTTATAGTAACGGCCGACATGAGAG	71	426	7	76.41	561	1	0
N/A	GGGACACAATGGACGTAAAGGTCGGGCTGTATTAATTTAACGGCCGACATGAGAG	55	426	7	76.41	535	1	0
N/A	GGGACACAATGGACGTATATAATAATCAATCGAGGTCGATAGCATAATTATGGCATAACGGCCGACATGAGAG	73	426	7	76.41	475	1	0
N/A	GGGACACAATGGACGAACCGCGAGACTAACGAGAATTTATAAGCTAACCGTAGTTTAACGGCCGACATGAGAG	73	426	7	76.41	530	1	0
N/A	GGGACACAATGGACGATACTAAAGTTTCCTTACACCAATAATGTATAACGGCCGACATGAGAG	63	426	7	76.41	488	1	0
N/A	GGGACACAATGGACGGAGGACAATGAAGCGTGAATGATAATAAAGGCACTCTTAATAACGGCCGACATGAGAG	73	426	7	76.41	519	1	0
N/A	GGGACACAATGGACGTTGTATTTCGAACTATTTATGTAGCGACGATTCGATTATCTAACGGCCGACATGAGAG	73	426	7	76.41	429	1	0
N/A	GGGACACAATGGACGTTAAGAGATTAAGTTATGAATTTAAGATTGACCTTGAACTTAACGGCCGACATGAGAG	73	426	7	76.41	543	1	0
N/A	GGGACACAATGGACGACCTGTCCTGATGATATGTTAGATATGATATGTTAGCTGGTAACGGCCGACATGAGAG	73	426	7	76.41	468	1	0
N/A	GGGACACAATGGACGGTTATGACTAATGTCACATTTTAAATAGTATGTGATATGCTAACGGCCGACATGAGAG	73	426	7	76.41	421	1	0
N/A	GGGACACAATGGACGACATTTCAGCCGGAATGAATACTGTAGAAGGAGAATGCATAACGGCCGACATGAGAG	72	426	7	76.41	446	1	0
N/A	GGGACACAATGGACGTAGTCTTGAAACATGTGACGTAAGTTACATAGTAAATGGGTAACGGCCGACATGAGAG	73	426	7	76.41	540	1	0
N/A	GGGACACAATGGACGACTAATAAAATGTCATCTAGGTCACCGTGCGGTTGTAGAATAACGGCCGACATGAGAG	73	426	7	76.41	469	1	0
N/A	GGGACACAATGGACGTAATAGTGAAGTATTACTTATATCTGTACCACGGACGCATTAACGGCCGACATGAGAG	73	426	7	76.41	552	1	0
N/A	GGGACACAATGGACGCCAGATAATGAGGTTGTTGTATGCATCAGCTTTGACTATATAACGGCCGACATGAGAG	73	426	7	76.41	473	1	0
N/A	GGGACACAATGGACGATTATTAAACATCTGTACATCTATACCCTACGTGAGGCTGTAACGGCCGACATGAGAG	73	426	7	76.41	437	1	0
N/A	GGGACACAATGGACGCGCCATAAATTAGGGTAGAAGTAATGTAAATTCCTGGGATTAACGGCCGACATGAGAG	73	426	7	76.41	466	1	0
N/A	GGGACACAATGGACGGTGCAAAGATAGGTATATTTATTGTACGCAACACTTTAGCTAACGGCCGCATGAGAG	72	426	7	76.41	478	1	0
N/A	GGGACACAATGGACGGCGATAATAGATATCATGAATTATTTTGAACTTAATCTAACGGCCGACATGAGAG	70	426	7	76.41	439	1	0
N/A	GGGACACAATGGACGAAGATTAATAAATTAGAAACACCATGAAAGGCCAAAAGTATAACGGCCGACATGAGAG	73	426	7	76.41	486	1	0
N/A	GGGACACAATGGACGATTAGACGGATGTCATCATTATGTAGGACGTATTAGACAATAACGGCCGACATGAGAG	73	426	7	76.41	504	1	0
N/A	GGGACACAATGGACGGAGTATAAAAATTTGGCTTACAATTTGAGCAGTTATAAACTAACGGCCGACATGAGAG	73	426	7	76.41	544	1	0
N/A	GGGACACAATGGACGCAATGATGCATCCGATGAAATATTAAATTGGCCAGATATGTAACGGCCGACATGAGAG	73	426	7	76.41	450	1	0
N/A	GGGACACAATGGACGCTGCAATAGTTGAGAGTAGTAGAACATATAACATTCACTATAACGGCCGACATGAGAG	73	426	7	76.41	461	1	0
N/A	GGGACACAATGGACGCTACTTAAACAATGGGATTATACAATATGGATATGACCAGTAACGGCCGACATGAGAG	73	426	7	76.41	433	1	0
N/A	GGGACACAATGGACGGATGCGTTATGAAATTGTATATGAAAAATACCAATATTTGTAACGGCCGACATGAGAG	73	426	7	76.41	569	1	0
N/A	GGGACACAATGGACGAATGTCATGCGTTGCGGATGATCTCAAACATAGACATCATAACGGCCGACATGAGAG	72	426	7	76.41	549	1	0
N/A	GGGACACAATGGACGCTGTATATATAGTCAATACCTTTGAGGGTGATCAACATAACGGCCGACATGAGAG	70	426	7	76.41	424	1	0
N/A	GGGACACAATGGACGGTGAGCACGATAAGTATGTGAATTTCTACAAAACTCTTCGTAACGGCCGACATGAGAG	73	426	7	76.41	492	1	0
N/A	GGGACACAATGGACGGTTAGGCATAACGCTTACGAAACGAAAACGTTTTAGAAGTAACGGCCGACATGAGAG	72	426	7	76.41	493	1	0
N/A	GGGACACAATGGACGCAAATAGAAAGGGAAAATGTTTTCGAATGATCCGATTCTTAACGGCCGACATGAGAG	72	426	7	76.41	426	1	0
N/A	GGGACACAATGGACGTGATATTAAATTGAATATGTTAGCTAGACTGCAGGAGCGGTAACGGCCGACATGAGAG	73	426	7	76.41	534	1	0
N/A	GGGACACAATGGACGTAGCGAGCATAGGTACCATCTTAAAATGATTATATGATAGGTAACGGCCGACATGAGAG	74	426	7	76.41	451	1	0
N/A	GGGACACAATGGACGCAATTATGTATTAAATAACTAACGGCCGACATGAGAG	52	426	7	76.41	420	1	0
N/A	GGGACACAATGGACGGCTACATTTTAATTTAAGGCTGGAATGAGAGTTTTAGGCTTAACGGCCGACATGAGAG	73	426	7	76.41	490	1	0
N/A	GGGACACAATGGACGTAATAGAACGCTCTAGACAGCCAAGTTGAATTCAATCAATAACGGCCGACATGAGAG	72	426	7	76.41	563	1	0
N/A	GGGACACAATGGACGGTCAGGGCTTTTTTGATACGCAAATGGACTTAAGAAAGCATAACGGCCGACATGAGAG	73	426	7	76.41	491	1	0
N/A	GGGACACAATGGACGCCTATTATATCAAGAAGACGTACTTACAACGTACTTAGATTAACGGCCGACATGAGAG	73	426	7	76.41	555	1	0
N/A	GGGACACAATGGACGCTGTTATGATGTATTATACTCTGTAGCTTAGGGTAAAGTTTAACGGCCGACATGAGAG	73	426	7	76.41	477	1	0
N/A	GGGACACAATGGACGCAACAGGTTTCTAGACATGGAAATATATTTGTCTAGCGATTAACGGCCGACATGAGAG	73	426	7	76.41	465	1	0
N/A	GGGACACAATGGACGCTGATTAAGATTTTGACTGTGACAACAAATAAGTCACGGATAACGGCCGACATGAGAG	73	426	7	76.41	518	1	0
N/A	GGGACACAATGGACGGTTTAGAACATATCGAAAACATCAGTTATATATCTGGTGTAACGGCCGACATGAGAG	72	426	7	76.41	453	1	0
N/A	GGGACACAATGGACGGTTCAAGTTAGAATCACTGTTATCGAGAATGACAGACATATAACGGCCGACATGAGAG	73	426	7	76.41	536	1	0

N/A	GGGACACAATGGACGAATAAATAACGCTATGAGTTACCCTGATATAACGGCCGACATGAGAG	62	426	7	76.41	452	1	0							
N/A	GGGACACAATGGACGTTGGAAGATTATTTTAACGGCCGACATGAGAG	47	426	7	76.41	7	3	5							
N/A	GGGACACAATGGACGCATAGAAGGTTAGGGTTTTGTGTAGCATCATAACGGAGTAACGGCCGACATGAGAG	71	426	7	76.41	537	1	0							
N/A	GGGACACAATGGACGCCTAATCGGGATGTTATTTAAACACATTTCTTAATACTCGTAACGGCCGACATGAGAG	73	426	7	76.41	485	1	0							
N/A	GGGACACAATGGACGGATTAGTTTGTAGTTTATATTTCAGGAATTAGGAGCTAGATAACGGCCGACATGAGAG	73	426	7	76.41	414	1	0							
N/A	GGGACACAATGGACGATTGGCTTAATTTAAATCTCTACAGTGCGTTTTCTGCCGGTAACGGCCGACATGAGAG	73	426	7	76.41	497	1	0							
N/A	GGGACACAATGGACGTAACATAGAATTTATATTAACGGCCGACATGAGAG	50	426	7	76.41	415	1	0							
N/A	GGGACACAATGGACGCTGGAGCTTTGATCTTGTCGAAATTATACAGGATTTATGTAACGGCCGACATGAGAG	72	426	7	76.41	432	1	0							
N/A	GGGACACAATGGACGTAATATAGTAAACAGGTGTAAGGCTCAAAGGGGATTACTCTAACGGCCGACATGAGAG	73	426	7	76.41	503	1	0							
N/A	GGGACACAATGGACGGTACCTATAGAAGCACTAGTTATAATTTTTAACCGCTTTGTAACGGCCGACATGAGAG	73	426	7	76.41	510	1	0							
N/A	GGGACACAATGGACGTTTAAGTTTATATAACATGGTGGAACCAAACATGGCGTGGTAACGGCCGACATGAGAG	73	426	7	76.41	454	1	0							
N/A	GGGACACAATGGACGTTAGGGTACAGTATAACAGATATTTACATGTTACGGTAGATAACGGCCGACATGAGAG	73	426	7	76.41	523	1	0							
N/A	GGGACACAATGGACGGTGCCGATTATTATTCAGTAACGGCCGACATGAGAG	51	426	7	76.41	428	1	0							
N/A	GGGACACAATGGACGGCTATAATTTATGGTTGTAGTAGATAACAATACACTACTAACGGCCGACATGAGAG	71	426	7	76.41	509	1	0							
N/A	GGGACACAATGGACGGTATTGACTAAATATAAATTTACATAATCAAGTTAGGCGATAACGGCCGACATGAGAG	73	426	7	76.41	551	1	0							
N/A	GGGACACAATGGACGACACGTAAATATCACATCAAAAGTTGATTCTAGCGATGGATAACGGCCGACATGAGAG	73	426	7	76.41	524	1	0							
N/A	GGGACACAATGGACGGGACATTTTCATCTTGATATATTTAAGGTAACGGCCGACATGAGAG	61	426	7	76.41	481	1	0							
N/A	GAGACACAATGGACGTTAGATATCAGAAAATTCATTTCTAGAGCTGATACATAGATAACGGCCGACATGAGAG	73	426	7	76.41	528	1	0							
N/A	GGGACACAATGGACGATTACATCAAATAGAGCAATTAACTAATCATGCGTCGGGCTAACGGCCGACATGAGAG	73	426	7	76.41	435	1	0							
N/A	GGGACACAATGGACGGACGCCAACAACATAGGATAATGATTTTTTCCTACACGGGAGTAACGGCCGACATGAGAG	75	426	7	76.41	557	1	0							
N/A	GGGACACAATGGACGTATTAAATAGACGGAATATTGATCCTATACACTATGGCCATAACGGCCGACATGAGAG	73	426	7	76.41	471	1	0							
N/A	GGGACACAATGGACGGGACTAAAATTCTAGTGACGAAATGATAGACACTGCGAGTTAACGGCCGACATGAGAG	73	426	7	76.41	505	1	0							
N/A	GGGACACAATGGACGTTTCTGGCACATGAGAATTATTTCGCACATGACATCGAGGTAACGGCCGACATGAGAG	73	426	7	76.41	430	1	0							
N/A	GGGACACAATGGACGCTTAACTGCAGATTTTTGCTAACGGCCGACATGAGAG	52	426	7	76.41	470	1	0							
N/A	GGGACACAATGGACGAACAAGACATGGATAACGGTTTAAAATATTATAAAATGTTAACGGCCGACATGAGAG	72	426	7	76.41	484	1	0							
N/A	GGGACACAATGGACGGACAAATACGAACTAGAGTAATATATTTTAGATTTTAGCGTAACGGCCGACATGAGAG	73	426	7	76.41	547	1	0							
N/A	GGGACACAATGGACGTAAATTCAGAAGTTTTATTCAGATTAACGGCCGACATGAGAG	57	426	7	76.41	512	1	0							
N/A	GGGACACAATGGACGCAGGATTGTTCTGAAGCGGAGAATATAATTTCTGAACAGATAACGGCCGACATGAGAG	73	426	7	76.41	423	1	0							
N/A	GGGACACAATGGACGTTGATAGATTTAATTGAAGGCTCTTAAGCTGGAATGAGTCTAACGGCCGACATGAGAG	73	426	7	76.41	514	1	0							
N/A	GGGACACAATGGACGGTATTCCTGATTATTCATTTTGATAGATTAACTTAGGGTATAACGGCCGACATGAGAG	73	426	7	76.41	427	1	0							
N/A	GGGACACAATGGACGGTGTAGTTCTATACAGATTACTAATAAATCTTCTTGGGACTAACGGCCGACATGAGAG	73	426	7	76.41	448	1	0							
N/A	GGGACACAATGGACGGTTATAGTTGAGCACCGAAACGAATTTTAGTTGCGACTCTTAACGGCCGACATGAGAG	73	426	7	76.41	442	1	0							
N/A	GGGACACAATGGACGTAGTAATATTTATTCATAACAGTATTGAGCCCGGGGCCACTAACGGCCGACATGAGAG	73	426	7	76.41	508	1	0							
N/A	GGGACACAATGGACGCCGATAAAATTACAAGATTATGTTTATAATATCTCGGGAATAACGGCCGACATGAGAG	73	426	7	76.41	539	1	0							
N/A	GGGACACAATGGACGTAATTGTTAGCTTACGAATTTCGAATGACTGGCATGATTAACGGCCGACATGAGAG	71	426	7	76.41	533	1	0							
N/A	GGGACACAATGGACGAAACGATAGAAAATTGTATAATGACACGGAGTTATGTTAATAACGGCCGACATGAGAG	73	426	7	76.41	501	1	0							
N/A	GGGACACAATGGACGTATGTTTCAAAATGGAAGGTTCGGAGTTGCAGTTGCAACATAACGGCCGACATGAGAG	73	426	7	76.41	567	1	0							
N/A	GGGACACAATGGACGTTAATTGGCTTCAATGTATGATTCTTATACGAAGTTACAATAACGGCCGACATGAGAG	73	426	7	76.41	513	1	0							
N/A	GGGGCACAATGGACGCCGGATCAGAAACAATAAGATAAATTAATTGGCTGTAGGCTAACGGCCGACATGAGAG	73	426	7	76.41	436	1	0							
N/A	GGGACACAATGGACGTAAGGCTATTAAGTCAGAGCAACTTTTGACTCGCTGACAATAACGGCCGACATGAGAG	73	426	7	76.41	548	1	0							
N/A	GGGACACAATGGACGAACGTAGTGATTAAAATATATATACTAACGGCCGACATGAGAG	58	426	7	76.41	502	1	0							
N/A	GGGACACAATGGACGGAATCGATAAGTTATTAACGGCCGACATGAGAG	48	426	7	76.41	434	1	0							
N/A	GGGACACAATGGACGTAATAGGTAAGGAAATTTTAAATAATCCAAATTCCGTAACTAACGGCCGACATGAGAG	73	426	7	76.41	467	1	0							
N/A	GGGACACAATGGACGGTGACAATACTAAATTTATCGTAGAAGACAAATTATAGGGTAACGGCCGACATGAGAG	73	426	7	76.41	417	1	0							
N/A	GGGACACAATGGACGGCAAAGTGTTAAACTGAATTATATTAGACTATTCAAATGATAACGGCCGACATGAGAG	73	426	7	76.41	416	1	0							
N/A	GGGACACAATGGACGTATGATACGCATGGAGGGCATTACTGTGATTATTTTTCTGTAACGGCCGACATGAGAG	73	426	7	76.41	527	1	0	2055	4	27.4	2926	1	0	0.3586
N/A	GGGACACAATGGACGA	16	426	7	76.41	3	12	2	2055	4	27.4	1	25	2	0.3586
N/A	GGGACACAATGGACGATACAGATTTTAACTGGGTAATCAAAATGTAACCAGGGCTTAACGGCCGACATGAGAG	73	426	7	76.41	444	1	0	1398	5	34.25	1355	1	0	0.4482
N/A	GGGACACAATGGACGAATTAATATTTAGTTAATGGGTTATATGCCCAGTTGGCTTTAACGGCCGACATGAGAG	73	426	7	76.41	562	1	0	995	6	41.09	1117	1	0	0.5378
N/A	GGGACACAATGGACGATTATACCAACGATGTACTAGTATGTAGGCTGTGCAAACATAACGGCCGACATGAGAG	73	585	6	65.49	640	1	0							
N/A	GGGACACAATGGACGTATAAATATATTTTGTCAAGGACGCCTTTAGAGGGGACTGTAACGGCCGACATGAGAG	73	585	6	65.49	713	1	0							
N/A	GGGACACAATGGACGTATGAATTATATCGTATAGTACCACTACATTGGCCTGGGCTAACGGCCGACATGAGAG	73	585	6	65.49	653	1	0							
N/A	GGGACACAATGGACGTAATTTCAATTTAACGGCCGACATGAGAGTAACGGCCGACATGAGAG	62	585	6	65.49	789	1	0							
N/A	GGGACACAATGGACGATATAATATCAGATAATTAGAAGTGGTGGAATCCAGGCAATAACGGCCGACATGAGAG	73	585	6	65.49	634	1	0							
N/A	GGGACACAATGGACGATACGCCGTGAATTATATAGTGAGACATGAAGTTCTCGATTAACGGCCGACATGAGAG	73	585	6	65.49	603	1	0							
N/A	GGGACACAATGGACGTATAACCTGTTATGCCGATCAGAAGGTGTAATAGAGTGATAACGGCCGACATGAGAG	72	585	6	65.49	747	1	0							
N/A	GGGACACAATGGACGAAATCTATATGTAAGTTACAAAGAATCAAGTGTTAGGGCGTAACGGCCGACATGAGAG	73	585	6	65.49	740	1	0							
N/A	GGGACACAATGGACGGAATGAATCTTTATTAAGCAATTAATACGCTTATAAGCATAACGGCCGACATGAGAG	72	585	6	65.49	724	1	0							
N/A	GGGACACAATGGACGTATGGAGGACAGTTAGTAGAAGATATTATTTTTGCCAAACTAACGGCCGACATGAGAG	73	585	6	65.49	651	1	0							
N/A	GGGACACAATGGACGGTAGCTAACGCATAGCGGGTTATGATGTATTATCACAGTATAACGGCCGACATGAGAG	73	585	6	65.49	631	1	0							

N/A	GGGACACAATGGACGAATTGACGAATTATCATTACAACTATGAATAAACCGATAATAACGGCCGACATGAGAG	73	585	6	65.49	694	1	0
N/A	GGGACACAATGGACGTTAAATGAAATTGGCTTTGTAGAAATAAAACGAGGCAATGTAACGGCCGACATGAGAG	73	585	6	65.49	768	1	0
N/A	GGGACACAATGGACGGTTGGACGTTATAAGCATCAGTTTTATTTCATGTTTTCGTAACGGCCGACATGAGAG	72	585	6	65.49	664	1	0
N/A	GGGACACAATGGACGGATGCGTAATGTGTATGTTATAATAACTAATGAACTTGCATAACGGCCGACATGAGAG	73	585	6	65.49	753	1	0
N/A	GGGACACAATGGACGCAAGACTTATTGAAATTAACGTTGCCTATGACGGCAGGTTTAACGGCCGACATGAGAG	73	585	6	65.49	775	1	0
N/A	GGGACACAATGGACGCCTTTACTCACCAAGGTATTAGATAACTGGAAGTATAGATAACGGCCGACATGAGAG	72	585	6	65.49	687	1	0
N/A	GGGACACAATGGACGGATAATGAAGTATCGACTGGTAAAATAATAGCAGAAAGTATAACGGCCGACATGAGAG	73	585	6	65.49	670	1	0
N/A	GGGACACAATGGACGCGTTGCTGATCCTATAAGTTAACTGGGGTTTGTATAAGAATAACGGCCGACATGAGAG	73	585	6	65.49	590	1	0
N/A	GGGACACAATGGACGGTGTAAGAGACGTATTCTCTATAAGAGCGTATGACTAATAACGGCCGACATGAGAG	71	585	6	65.49	580	1	0
N/A	GGGACACAATGGACGTTAAAAAAGTGATTAAATTAGATAACGGCCGACATGAGAG	55	585	6	65.49	629	1	0
N/A	GGGACACAATGGACGTAACCGCAAAATGTGAGTTTTAATAGACCTAAATTTGAGCTAACGGCCGACATGAGAG	73	585	6	65.49	581	1	0
N/A	GGGACACAATGGACGTCATCATTTATATGGATGAGCATTTTGAGGCCCTGAGGCCTAACGGCCGACATGAGAG	73	585	6	65.49	693	1	0
N/A	GGGACACAATGGACGTAATTAAGACTATGATAACGAAACTCGTTGAGGCTCTGGCTAACGGCCGACATGAGAG	73	585	6	65.49	624	1	0
N/A	GAGACACAATGGACGTAGGCCTTGGGTTAAGATGGTTATATTCTTTATCCATTTTCTAACGGCCGACATGAGAG	74	585	6	65.49	706	1	0
N/A	GGGACACAATGGACGGTCACAATGTAGACAAATATTAGAATTAATTATGGGTTTGTAACGGCCGACATGAGAG	73	585	6	65.49	742	1	0
N/A	GGGACACAATGGACGGTAGATACTATTATTGTAAATTAAGAGATTGGAATGATGGTAACGGCCGACATGAGAG	73	585	6	65.49	577	1	0
N/A	GGGACACAATGGACGCACAGAAACATATCATTATTATGCTAAACGGCCGACATGAGAG	58	585	6	65.49	399	2	1
N/A	GGGACACAATGGACGGAATACTTAAACGGCAAACTTATTACAAGGAGTTTACCACTAACGGCCGACATGAGAG	73	585	6	65.49	607	1	0
N/A	GGGACACAATGGACGCAAGGTACTTGAAACTGATGTATTTGGTAACTTCATCAAGTAACGGCCGACATGAGAG	73	585	6	65.49	766	1	0
N/A	GGGACACAATGGACGTTAGAGCTGCTATAAGGTAAATAGGAAATTACATAAGCGTTAACGGCCGACATGAGAG	73	585	6	65.49	655	1	0
N/A	GGGACACAATGGACGTAGGAAATGACGTTTAACCTTTCGAAAAGATATCATAACCTAACGGCCGACATGAGAG	73	585	6	65.49	689	1	0
N/A	GGGACACAATGGACGTATATATGTAAATAATGAAGGCCTAAGTACGATATAAGTATAACGGCCGACATGAGAG	73	585	6	65.49	593	1	0
N/A	GGGACACAATGGACGGTAAAATAAAGTTATAAGTGGAACCCATATAGGGCCGGGATAACGGCCGACATGAGAG	73	585	6	65.49	699	1	0
N/A	GGGACACAATGGACGAGAGAGAAGAGTAAGAGCAATTAATAAATTACATTACTTCTAACGGCCGACATGAGAG	73	585	6	65.49	682	1	0
N/A	GGGACACAATGGACGGATAAACGGAAGCTTATTAGACAAATATTAAGTCTGAGCCTAACGGCCGACATGAGAG	73	585	6	65.49	620	1	0
N/A	GGGACACAATGGACGGTGTTATATCATAAGGGGCACGTAGTGAAAATAGTTGACTTAACGGCCGACATGAGAG	73	585	6	65.49	697	1	0
N/A	GGGACACAATGGACGTATCTTGATCTAACCAAGTGAATATACTTGAGATTGGTGATAACGGCCGACATGAGAG	73	585	6	65.49	714	1	0
N/A	GGGACACAATGGACGGCATTCAGTTTTTTTGACATGACATAAGTTATCGCACAAGGTAACGGCCGACATGAGAG	74	585	6	65.49	786	1	0
N/A	GGGACACAATGGACGTTATGGCCGATACATATGTTAAACACATCGAAAACTTTGATAACGGCCGACATGAGAG	73	585	6	65.49	765	1	0
N/A	GGGACACAATGGACGTC	17	585	6	65.49	3	15	3
N/A	GGGACACAATGGACGTAAAGGTAACTTTTAGTGCAAATTGATGCCATATCGGATATAACGGCCGACATGAGAG	73	585	6	65.49	715	1	0
N/A	GGGACACAATGGACGTTTTAGTAATTATGACCGGCGGAACTTTAGCCATTAATACTAACGGCCGACATGAGAG	73	585	6	65.49	675	1	0
N/A	GGGACACAATGGACGTTAAGACGAACGTATAAAGGTATCGAAGTCATTTGATGACTAACGGCCGACATGAGAG	73	585	6	65.49	770	1	0
N/A	GGGACACAATGGACGTGTAGGGATATTTGATAATTATAAAGGTGTAATACACCCTAACGGCCGACATGAGAG	72	585	6	65.49	597	1	0
N/A	GGGACACAATGGACGATCAGGTAACATTGGCCAGGCAGTAGAACTTATTTTTAGCTTAACGGCCGACATGAGAG	74	585	6	65.49	762	1	0
N/A	GGGACACAATGGACGATTAAGATTTACATCTATTGACATAACCCTATGCGGTTGGTAACGGCCGACATGAGAG	73	585	6	65.49	755	1	0
N/A	GGGACACAATGGACGTGAGAAGACCAGAGCTGTTATTAGTTGTATTTACTAACTCTAACGGCCGACATGAGAG	73	585	6	65.49	659	1	0
N/A	GGGACACAATGGACGATTAATACTTAGTTGAATTGAGTATGTCAGAATGTAGGCTTAACGGCCGACATGAGAG	73	585	6	65.49	738	1	0
N/A	GGGACACAATGGACGGACAAGAGATAAGAATATATAATAACTTTTTCTAACGGCCGACATGAGAG	65	585	6	65.49	636	1	0
N/A	GGGACACAATGGACGTTGGAAGTAATTTCAGAGTGTTATTGTACAGCCTGCCGGTTAACGGCCGACATGAGAG	73	585	6	65.49	787	1	0
N/A	GGGACACAATGGACGGATACATCGTAGGCTAATATCTTTAAGGCAAGTAAAGGTCTAACGGCCGACATGAGAG	73	585	6	65.49	754	1	0
N/A	GGGACACAATGGACGTTAAGTTTTTGGGTTAAGATCTTTTTGGAACAACCACACATAACGGCCGACATGAGAG	73	585	6	65.49	648	1	0
N/A	GGGACACAATGGACGTAAGACCGGTGACAAATGGAGGTTCGTATATTACTTGAATAACGGCCGACATGAGAG	72	585	6	65.49	625	1	0
N/A	GGGACACAATGGACGGTAGGTCAATCGATGAGATGTATATAACGGCCGACATGAGAG	57	585	6	65.49	598	1	0
N/A	GGGACACAATGGACGAGAATTTAAATCGACTGATTGCACATACAATGCTAGGCTATAACGGCCGACATGAGAG	73	585	6	65.49	622	1	0
N/A	GGGACACAATGGACGTTAACAATCGTAAAATATATACTAACACGTAACGGCCGACATGAGAG	62	585	6	65.49	570	1	0
N/A	GGGACACAATGGACGGGCGAACTCTAAACTATTTAATTTACAAATCAGGAAGGGGTAACGGCCGACATGAGAG	73	585	6	65.49	716	1	0
N/A	GGGACACAATGGACGAACCGTTACTATCAATATTTAATGCATCATAACAATCTGGTAACGGCCGACATGAGAG	73	585	6	65.49	717	1	0
N/A	GGGACACAATGGACGTCGGTGCTGGACTAGAAGGTAAAGGCATTATCAACGGGGATAACGGCCGACATGAGAG	73	585	6	65.49	657	1	0
N/A	GGGACACAATGGACGTAACCAAAACAATCAGGATAGACGAAATTTTGATGGGCAATAACGGCCGACATGAGAG	73	585	6	65.49	643	1	0
N/A	GGGACACAATGGACGTTTACAAAATGTAGTGATGCTTCATGGCTAGCTGCATATATAACGGCCGACATGAGAG	73	585	6	65.49	746	1	0
N/A	GGGACACAATGGACGTTACTGTAACAATATGTTTTTGCATGATTCAATAAGCAATGTAACGGCCGACATGAGAG	74	585	6	65.49	723	1	0
N/A	GGGACACAATGGACGAACGGGTAAGATTATGAGCGATTAGTTAACTTCATGATCCTAACGGCCGACATGAGAG	73	585	6	65.49	646	1	0
N/A	GGGACACAATGGACGCTCTATGAAATACATACGGAATTATGGTTTATAAGATAACGGCCGACATGAGAG	69	585	6	65.49	572	1	0
N/A	GGGACACAATGGACGCTATGTGAAATCCAAGGGTAATACAATAACCTACCGAGATTAACGGCCGACATGAGAG	73	585	6	65.49	576	1	0
N/A	GGGACACAATGGACGAACACGCATAATGTTATATAGTCACACTTGTAGGAAGGCTTAACGGCCGACATGAGAG	73	585	6	65.49	728	1	0
N/A	GGGACACAATGGACGAGGGCGGAGTTTAATGATACACAATCTAATTCCCTTTGCATAACGGCCGACATGAGAG	73	585	6	65.49	635	1	0
N/A	GGGACACAATGGACGTAACGTACTGATAAGAAATAATACGATGCCGTTAATACCATAACGGCCGACATGAGAG	73	585	6	65.49	573	1	0
N/A	GGGACACAATGGACGAACATGCAAGTAGGCACGGATTATATTGCAACATTTAATATAACGGCCGACATGAGAG	73	585	6	65.49	704	1	0
N/A	GGGACACAATGGACGCATGGTTAATAATATCAGTGAAAAGGAGACTTCTTCAAATTAACGGCCGACATGAGAG	73	585	6	65.49	771	1	0

N/A	GGGACACAATGGACGCCTAATTATTTATAAGGATCTAATTCTTACGATACTTGTATAACGGCCGACATGAGAG	73	585	6	65.49	725	1	0
N/A	GGGACACAATGGACGGTAAAGAAGTAATGTACTTTTATATATAGAGCATTACAGATAACGGCCGACATGAGAG	73	585	6	65.49	594	1	0
N/A	GGGACACAATGGACGATAATATATAATATGAACGGTCGATAATTTTCTTAGCGGATAACGGCCGACATGAGAG	73	585	6	65.49	584	1	0
N/A	GGGACACAATGGACGAGACCAAGCGTTATGAGTATATATAAACGACCGTAACAGCTAACGGCCGACATGAGAG	73	585	6	65.49	592	1	0
N/A	GGGACACAATGGACGACTGTAATAATAATTTAGGCTAGATAGAACTGATAAAGCTAACGGCCGACATGAGAG	72	585	6	65.49	720	1	0
N/A	GGGACACAATGGACGTTCTATATCCATAATAAACATATATGGGATCTAACGGCCGACATGAGAG	64	585	6	65.49	721	1	0
N/A	GGGACACAATGGACGTAAATTTGACTGAGTAACAGCATTGATCCACTAGTGAGGCTAACGGCCGACATGAGAG	73	585	6	65.49	661	1	0
N/A	GGGACACAATGGACGGATTTAACCGGTAACATATTGATATACTGGAATGGGCTGGTAACGGCCGACATGAGAG	73	585	6	65.49	677	1	0
N/A	GGGACACAATGGACGTTAACAGACTAAACGACGGAGTGTGATGTTGGTTTTACACTTAACGGCCGACATGAGAG	74	585	6	65.49	663	1	0
N/A	GGGACACAATGGACGGTAGAAATCAAATAATTCGAGAAATGGTTAAGAGTTGAAGTAACGGCCGACATGAGAG	73	585	6	65.49	642	1	0
N/A	GGGACACAATGGACGCGTAGTATGAAGACACTGATATTTCAGACAGTATGCAACTTAACGGCCGACATGAGAG	73	585	6	65.49	654	1	0
N/A	GGGACACAATGGACGAAACCGACAATATAATGGATTATTATTTTATTGACTAAGGTAACGGCCGACATGAGAG	73	585	6	65.49	650	1	0
N/A	GGGACACAATGGACGCTACGTACGAAATAGTCAGCTGCAATATGCATATAAGAGTAACGGCCGACATGAGAG	72	585	6	65.49	588	1	0
N/A	GGGACACAATGGACGTAGTCTATAAACTGACGCTTTCATAGTACCTACAGGCCAATAACGGCCGACATGAGAG	73	585	6	65.49	790	1	0
N/A	GGGACACAATGGACGTTGTATGTTTCTTTATGTACTAAAAGAAGGAACTCAGTGATAACGGCCGACATGAGAG	73	585	6	65.49	739	1	0
N/A	GGGACACAATGGACGCCGACTATAACTATAATAATGAAGGAGTTAGGTTAGCATAACGGCCGACATGAGAG	71	585	6	65.49	571	1	0
N/A	GGGACACAATGGACGAGTTGTAAGTAGGTATCAATGGATAATATGGTACTGACCTTAACGGCCGACATGAGAG	73	585	6	65.49	604	1	0
N/A	GGGACACAATGGACGTGCTTTTACACAACTAGTGATTATGTGGATCGTTGGTTTATAACGGCCGACATGAGAG	73	585	6	65.49	729	1	0
N/A	GGGACACAATGGACGTTAACAGTTATTCAGTATTCTTATTTGTCTGATCGCGAGTTAACGGCCGACATGAGAG	73	585	6	65.49	743	1	0
N/A	GGGACACAATGGACGGTATAATGTCGGGGAGTCAGATTTAACTAAGCTCTAAACCTAACGGCCGACATGAGAG	73	585	6	65.49	600	1	0
N/A	GGGACACAATGGACGTACAGTAAGCAAAGGTTTTTTGTCACAATTATTACCTAACGGCCGACATGAGAG	69	585	6	65.49	793	1	0
N/A	GGGACACAATGGACGTTGTTGAATGCGTAAAGTTATATAGTAGTAATGTTAAGGCTAACGGCCGACATGAGAG	73	585	6	65.49	734	1	0
N/A	GGGACACAATGGACGATGAAGTTGGCTAACGGTATTAGGACAGTTTACTGTTCATTAACGGCCGACATGAGAG	73	585	6	65.49	644	1	0
N/A	GAGACACAATGGACGATACTTATTATATCACAACGTGGACTCAAAAGTTGGATGTAACGGCCGACATGAGAG	72	585	6	65.49	759	1	0
N/A	GGGACACAATGGACGCCACAAGAGAACATTACTTAGTATTAACCGGTAAGTTTTAGTAACGGCCGACATGAGAG	74	585	6	65.49	751	1	0
N/A	GGGACACAATGGACGGCAAACGAAGAGAGAATGACTAATTCGAGATTCTTCGCTATAACGGCCGACATGAGAG	73	585	6	65.49	630	1	0
N/A	GGGACACAATGGACGGTATTGAGAAATGAGGAGTTTATTCTTCTTACTTCACAGTAACGGCCGACATGAGAG	72	585	6	65.49	619	1	0
N/A	GGGACACAATGGACGCTTTTTCACGAGAATCAAGGCAATAACCGACATTTTCAGGTAACGGCCGACATGAGAG	73	585	6	65.49	783	1	0
N/A	GGGACACAATGGACGGTACTAGGATTAGGAAGGTAAATACTTCAATATTTTTGCCTAACGGCCGACATGAGAG	73	585	6	65.49	773	1	0
N/A	GGGACACAATGGACGTAATCATCTTAGGATAACAAACACAATTATTCTGTAGAAGTAACGGCCGACATGAGAG	73	585	6	65.49	679	1	0
N/A	GGGACACAATGGACGTTCTAAACTGAATGACGGACCTATATCAATAACGGCCGACATGAGAG	62	585	6	65.49	673	1	0
N/A	GGGACACAATGGACGTATAATCAAAGGCTGGATATTCATCAGACTAATAGTGAGTAACGGCCGACATGAGAG	72	585	6	65.49	579	1	0
N/A	GGGACACAATGGACGCAGATACACATAATGATCATAGTGAAGTGAGTTATTAAGGTAACGGCCGACATGAGAG	73	585	6	65.49	585	1	0
N/A	GGGACACAATGGACGTGAACATACAGTGAAGATACTATGAGAAACAACTGTAAATAACGGCCGACATGAGAG	72	585	6	65.49	658	1	0
N/A	GGGACACAATGGACGTGAGGCACGTGGATGAACAACTGGTTATTTAAACGATTGGTAACGGCCGACATGAGAG	73	585	6	65.49	703	1	0
N/A	GGGACACAATGGACGATGGGGGTAGGTTTGTAATAATAAAGAATTGAAGCACTAACGGCCGACATGAGAG	70	585	6	65.49	595	1	0
N/A	GGGACACAATGGACGGAAGAAACAAGTTCAGGTAAGGTATATCTGTAGTTGATTATAACGGCCGACATGAGAG	73	585	6	65.49	730	1	0
N/A	GGGACACAATGGACGATGTTGCGGCAAAAAGCAAGTAATAACCTTACTATCAGCTAACGGCCGACATGAGAG	72	585	6	65.49	785	1	0
N/A	GGGACACAATGGACGCACAGCGATAGACTATCAATTACATGAGAACCTAACGACGTAACGGCCGACATGAGAG	73	585	6	65.49	744	1	0
N/A	GGGACACAATGGACGCAATATTTTATTACAAGACCACTTATCCTGGCTGGAAATGTAACGGCCGACATGAGAG	73	585	6	65.49	761	1	0
N/A	GGGACACAATGGACGTCATTAGGCAGATTAAAGTATTAAGCAATAATCTTAAGGCTAACGGCCGACATGAGAG	73	585	6	65.49	613	1	0
N/A	GGGACACAATGGACGCTTGAAGGTCATTAAACAGAAACTTAGAATATGATTTGATTAACGGCCGACATGAGAG	73	585	6	65.49	767	1	0
N/A	GGGACACAATGGACGGTAAAGACGTTTTAAAGATCCCTATAGGCCTAACGGCCGACATGAGAG	63	585	6	65.49	741	1	0
N/A	GGGACACAATGGACGAACTATTATAGAAAATGTATTAGTGCAGATGTTCTGAGGCTAACGGCCGACATGAGAG	73	585	6	65.49	666	1	0
N/A	GGGACACAATGGACGTACTCATCGGATAACCTACAATATTGTTAGCTGGTGGAAATAACGGCCGACATGAGAG	73	585	6	65.49	776	1	0
N/A	GGGACACAATGGACGGAAGTTTGTAATATTAATAGAACCACAATGGCGACTATGATAACGGCCGACATGAGAG	73	585	6	65.49	582	1	0
N/A	GGGACACAATGGACGGTTACGATTTTTAACACTAGTCATTACAAGGGCCACGTAACGGCCGACATGAGAG	70	585	6	65.49	780	1	0
N/A	GGGACACAATGGACGACTAAGAGTCAGGAGAATAAATTTGAGTTTTGTACTGATTAACGGCCGACATGAGAG	72	585	6	65.49	626	1	0
N/A	GGGACACAATGGACGTGCATAGTTAATTGTAGACATTAGCAATTTGATCGACAAGTAACGGCCGACATGAGAG	73	585	6	65.49	671	1	0
N/A	GGGACACAATGGACGGCTGAATTATAAGTAAAAACATAATTAGTAAAATCGCTACTTAACGGCCGACATGAGAG	74	585	6	65.49	575	1	0
N/A	GGGACACAATGGACGTCGCCTATTAAGTGAAGGTATCAAATTATTCATTAAACCGTAACGGCCGACATGAGAG	73	585	6	65.49	709	1	0
N/A	GGGACACAATGGACGTATTCATCTATGATCATTAGATCCTATGACGACCGATGACTAACGGCCGACATGAGAG	73	585	6	65.49	726	1	0
N/A	GGGACACAATGGACGACAAAACAACATTCGGCATCACATAAACATTAAGCAATTATAACGGCCGACATGAGAG	73	585	6	65.49	662	1	0
N/A	GGGACACAATGGACGGAGTTAAGTAAGCGATATTTTAACATATCAGTTTCGCTGATAACGGCCGACATGAGAG	73	585	6	65.49	784	1	0
N/A	GGGACACAATGGACGGATGAGTATATTGTACCACAGTTAGGGCTATTTATTAAGATAACGGCCGACATGAGAG	73	585	6	65.49	764	1	0
N/A	GGGACACAATGGACGCGACGAAAGAG	26	585	6	65.49	756	1	0
N/A	GGGACACAATGGACGTAACAAATAGTATCACCTAAATTCATGATTTGTGCTATAATAACGGCCGACATGAGAG	73	585	6	65.49	601	1	0
N/A	GGGACACAATGGACGAAGTGAAGGCCTTAATTAGTTTGTTTATAAAGCTATTTGGTAACGGCCGACATGAGAG	73	585	6	65.49	691	1	0
N/A	GGGACACAATGGACGGTCAAT	21	585	6	65.49	707	1	0
N/A	GGGACACAATGGACGGTTGGAAGATTAAAGATATGAAAGTTATGAAGATAGACAGTAACGGCCGACATGAGAG	73	585	6	65.49	758	1	0

N/A	GGGACACAATGGACGTTTTCACGCAAGATACCAATTTCAACGGTAAGTTTGAGCATAACGGCCGACATGAGAG	73	585	6	65.49	608	1	0
N/A	GGGACACAATGGACGGATGTAATTAGCAGAAACCAAAAATCAGAGTTTGCCTGGCTAACGGCCGACATGAGAG	73	585	6	65.49	605	1	0
N/A	GGGACACAATGGACGATCAAAAATCGGAGATGTAGTGAACAATGTATTTGCATAACTAACGGCCGACATGAGAG	74	585	6	65.49	774	1	0
N/A	GGGACACAATGGACGGACATTCATATATAGTAGCTTATGTGCTAGCATTATAGGGTAACGGCCGACATGAGAG	73	585	6	65.49	695	1	0
N/A	GGGACACAATGGACGGCATGGCATAGTCATTATACATTAGAATAAGCTGAGAAGTTAACGGCCGACATGAGAG	73	585	6	65.49	583	1	0
N/A	GGGACACAATGGACGTTACTGATATCATAGCAACTTTTCGCGCGGCAAACATAGATAACGGCCGACATGAGAG	73	585	6	65.49	745	1	0
N/A	GGGACACAATGGACGTAAACTTTTGCGTGTTGAACAGATCTTGATGGTCGTACGGTAACGGCCGACATGAGAG	73	585	6	65.49	684	1	0
N/A	GGGACACAATGGACGAGAGTTATTTAAAACATGAATTCACCCTTGTTAGCCGATATAACGGCCGACATGAGAG	73	585	6	65.49	708	1	0
N/A	GGGACACAATGGACGTAAAAGTTACTTAGATAGGCTAATTTATTCTAACTTGATTTAACGGCCGACATGAGAG	73	585	6	65.49	639	1	0
N/A	GGGACACAATGGACGAATGTTCTTTATACACATTCTGACGACCAAGTCACGGCTTTAACGGCCGACATGAGAG	73	585	6	65.49	698	1	0
N/A	GGGACACAATGGACGACAATGTACACTAATGCATGCAAGATTGACATTAAAGATGTAACGGCCGACATGAGAG	73	585	6	65.49	589	1	0
N/A	GGGACACAATGGACGGACTTGCCGATCTAACAGGAAAATAAATCTGAGTAGCGAGTAACGGCCGACATGAGAG	73	585	6	65.49	656	1	0
N/A	GGGACACAATGGACGTGTTGGAACCAAGGTCGTAACTTACATCTTATATTTGGGGTAACGGCCGACATGAGAG	73	585	6	65.49	757	1	0
N/A	GGGACACAATGGACGAATATTATAGGAATAACGAGAGTAGTCGGAATCTTTTAAGTAACGGCCGACATGAGAG	73	585	6	65.49	614	1	0
N/A	GGGACACAATGGACGGAAATTGATAATGGTTATCACTATGAATAATAGTTAAGCCTAACGGCCGACATGAGAG	73	585	6	65.49	791	1	0
N/A	GGGACACAATGGACGGTAGTGAGTAGAGATGCAAAAGAGTTTATCACATGTATACTAACGGCCGACATGAGAG	73	585	6	65.49	628	1	0
N/A	GGGACACAATGGACGTTATAAATGACAGAGTGGCAATATACAAAATTCATAATGTTAACGGCCGACATGAGAG	73	585	6	65.49	632	1	0
N/A	GGGACACAATGGACGTATAATTTTACTTTGACGACTATCTCAAGGTATGGTTGGTAACGGCCGACATGAGAG	72	585	6	65.49	672	1	0
N/A	GGGACACAATGGACGCCTATTAATGCTGTTAAAAACTGAATGTTCAGGTAATGGATAACGGCCGACATGAGAG	73	585	6	65.49	779	1	0
N/A	GGGACACAATGGACGCACGAATACCAAAGACGGATACTTTAACGTCTGACGAACATAACGGCCGACATGAGAG	73	585	6	65.49	788	1	0
N/A	GGGACACAATGGACGTTAAATACATCATAAGTTTTACAGACAATATTGGTAGATATAACGGCCGACATGAGAG	73	585	6	65.49	712	1	0
N/A	GGGACACAATGGACGTGTAATATTCAAATTTGCAGAGTTCTAGTCGCCTCAGGGCTAACGGCCGACATGAGAG	73	585	6	65.49	633	1	0
N/A	GGGACACAATGGACGGAATGACGATCAAAGGATATAAATTAACCGGAGACCTGCATAACGGCCGACATGAGAG	73	585	6	65.49	733	1	0
N/A	GGGACACAATGGACGCGCTGGGAGTAACGAAGACAATATAAGTAATCAGTTAACCTAACGGCCGACATGAGAG	73	585	6	65.49	710	1	0
N/A	GGGACACAATGGACGTTAGTTATAGGAATGGTCTGCGTGATTTATGTATTATGGTTAACGGCCGACATGAGAG	73	585	6	65.49	638	1	0
N/A	GGGACACAATGGACGAACGTTTGATTTTGAACACGGATGTCATAAGCTGTGAGGCTAACGGCCGACATGAGAG	73	585	6	65.49	696	1	0
N/A	GGGACACAATGGACGTAAGTCTAGAAAGTACTAGGCAAAATAAAGCGCTCATGTAACGGCCGACATGAGAG	71	585	6	65.49	615	1	0
N/A	GGGACACAATGGACGAACTTGAGAAAAATATAGTATAACGGCCGACATGAGAG	53	585	6	65.49	700	1	0
N/A	GGGACACAATGGACGTAGATTTCAATATCCAAATCATCGACTTAGTGCAAGGCTTAACGGCCGACATGAGAG	72	585	6	65.49	727	1	0
N/A	GGGACACAATGGACGACTGATTACATCACGAAACGTTTTATCCTTAGTGCGGTATAACGGCCGACATGAGAG	72	585	6	65.49	665	1	0
N/A	GGGACACAATGGACGAACGTATTAATAACGGCCGACATGAGAG	43	585	6	65.49	279	2	5
N/A	GGGACACAATGGACGTGCTAGTACAAAGGAAGATAAGTACGATTTATAAAGACTGTAACGGCCGACATGAGAG	73	585	6	65.49	781	1	0
N/A	GGGACACAATGGACGAGGGGAATTTCAGACGGCAAAAGCATACAGGATTTAGATGTAACGGCCGACATGAGAG	73	585	6	65.49	599	1	0
N/A	GGGACACAATGGACGGCAGGAATATAAAATGCTGATGAATATAATCGCTACTATGTAACGGCCGACATGAGAG	73	585	6	65.49	782	1	0
N/A	GGGACACAATGGACGGACTACGCTAAACTCATCAAATCAAGTAAATTGCTGAGGCTAACGGCCGACATGAGAG	73	585	6	65.49	587	1	0
N/A	GGGACACAATGGACGGAGTTAAAGTGAAACGTATATTTATACGCTCATACTATGTTAACGGCCGACATGAGAG	73	585	6	65.49	792	1	0
N/A	GGGACACAATGGACGTTAAGCAGTTATCTAAAATCTTTCTGATATAGAACGCCTAACGGCCGACATGAGAG	71	585	6	65.49	683	1	0
N/A	GGGACACAATGGACGTCGATATGACTGTAATTTTACAAAAATCATCATCATGGCATAACGGCCGACATGAGAG	73	585	6	65.49	718	1	0
N/A	GGGACACAATGGACGGTTATGATTAGACATATGATGATGGAAATCTCAGTATAGTTAACGGCCGACATGAGAG	73	585	6	65.49	731	1	0
N/A	GGGACACAATGGACGCTTAACAAGATAAAGAACCAAATGTCAACAAATGTAGGTCTAACGGCCGACATGAGAG	73	585	6	65.49	596	1	0
N/A	GGGACACAATGGACGAATTTGAGATCCAAGAATAACGGCCGACATGAGAG	50	585	6	65.49	688	1	0
N/A	GGGACACAATGGACGCTTGCTTCTAAGTATATTATAAGACGGCTAATGGGCTAACGGCCGACATGAGAG	69	585	6	65.49	772	1	0
N/A	GGGACACAATGGACGCAGTAACGAATAACAAGTCAAGCCGATTCTTATTATGTAATAACGGCCGACATGAGAG	73	585	6	65.49	769	1	0
N/A	GGGACACAATGGACGCGGAGCTTGGTCAAGATATAAAAATCGATAAAAAAAGCCGAGATAACGGCCGACATGAG/	76	585	6	65.49	652	1	0
N/A	GGGACACAATGGACGTTGATTAATAATCCAAGACGTATAGTATCTTTGAAGAGGTTAACGGCCGACATGAGAG	73	585	6	65.49	690	1	0
N/A	GGGACACAATGGACGTAGGATTAAGCAATTGTATGAATTATACAGGCTGATTCCCTAACGGCCGACATGAGAG	73	585	6	65.49	736	1	0
N/A	GGGACACAATGGACGCTAGAGTGCCGGTGAAACAATAAAAAGATATTTCAACCAGTAACGGCCGACATGAGAG	73	585	6	65.49	778	1	0
N/A	GGGACACAATGGACGATTATGAAACAATTTGAAGGCTTTTGATGGTTTAGACCTTAACGGCCGACATGAGAG	72	585	6	65.49	617	1	0
N/A	GGGACACAATGGACGCTGAGATGTTTTTAGAGATGAATAACCTGAAATATCGTAACGGCCGACATGAGAG	70	585	6	65.49	705	1	0
N/A	GGGACACAATGGACGCTATAGGGAGTTATAAAGTTATTAAACTAGGACAACACCTAACGGCCGACATGAGAG	72	585	6	65.49	591	1	0
N/A	GGGACACAATGGACGTGTTACTGGGACGATTATGATATAACGGCCGACATGAGAG	55	585	6	65.49	637	1	0
N/A	GGGACACAATGGACGTTAGTAATTATCTTGGTCAGAATCTATATATACTGATCACTAACGGCCGACATGAGAG	73	585	6	65.49	676	1	0
N/A	GGGACACAATGGACGCATAAAGGCAAAATACAGTACTATTTGTTTAATCTAACTGTAACGGCCGACATGAGAG	73	585	6	65.49	578	1	0
N/A	GGGACACAATGGACGATTTGTTCTATAACGGCCGACATGAGAG	43	585	6	65.49	279	3	6
N/A	GGGACACAATGGACGGTAGTACTCAAAAAATTGATTATTATAACAATACTGAGATAACGGCCGACATGAGAG	72	585	6	65.49	674	1	0
N/A	GGGACACAATGGACGTAACATTCAAATTGAAAGTTCAAAGATGGCTGGAATTAAATAACGGCCGACATGAGAG	73	585	6	65.49	735	1	0
N/A	GGGACACAATGGACGTACGGCAAAATTGGTAGCTAATGGGATAATAATTTAGTTTCTAACGGCCGACATGAGAG	74	585	6	65.49	732	1	0
N/A	GGGACACAATGGACGATACATGCAGGTAACTATTAAAAACAATGTAACGGCCGACATGAGAG	62	585	6	65.49	667	1	0
N/A	GGGACACAATGGACGGACTTGACGACTGCCTTAGAGAATACACTATATAGTAGTCTAACGGCCGACATGAGAG	73	585	6	65.49	669	1	0
N/A	GGGACACAATGGACGCCGAAAATGATACTATAATTATTAAACATGGTAATTCACCTAACGGCCGACATGAGAG	73	585	6	65.49	645	1	0

N/A	GGGACACAATGGACGCAAAACGTATTTTAAACACCGCGTGTAGGCTGTTAACAATAACGGCCGACATGAGAG	72	585	6	65.49	702	1	0							
N/A	GGGACACAATGGACGATAGATGGTATTAGTAGATATACTAGAATAACTTTTCATCTAACGGCCGACATGAGAG	73	585	6	65.49	750	1	0							
N/A	GGGACACAATGGACGTATAATCGGGTCGTGAAAACGGCATTAAATAACGGCCGACATGAGAG	62	585	6	65.49	722	1	0							
N/A	GGGACACAATGGACGATTATACAGATTTGAGACCAGGGTCCTATAGAATTAGGCTTAACGGCCGACATGAGAG	73	585	6	65.49	641	1	0							
N/A	GGGACACAATGGACGCCAAGCGACATGAGTAGATTATATGATTTATGTCTGATGTAACGGCCGACATGAGAG	72	585	6	65.49	602	1	0							
N/A	GGGACACAATGGACGAGGAGGCGGTTATATTTATAAATAACGGCCGACATGAGAG	55	585	6	65.49	686	1	0							
N/A	GGGACACAATGGACGCAGTTGGTATAACTAGATGTATAGTGATATTAGGCTAGGCTAACGGCCGACATGAGAG	73	585	6	65.49	763	1	0							
N/A	GGGACACAATGGACGTCTTAGACGATGAATGAAGTATTTAAACAAAATCATCAACTAACGGCCGACATGAGAG	73	585	6	65.49	795	1	0							
N/A	GGGACACAATGGACGATTACGCAAGTTTGAATTTGAAAGTAACGGCCGACATGAGAG	57	585	6	65.49	748	1	0							
N/A	GGGACACAATGGACGGAAAACCTTCAACGAGTTATCTATGTTAATAACTGGTTTGTAACGGCCGACATGAGAG	73	585	6	65.49	752	1	0							
N/A	GGGACACAATGGACGACATCAGAGGTAGAAAGTGGATATTAGAACTTCACTCCGATAACGGCCGACATGAGAG	73	585	6	65.49	685	1	0							
N/A	GGGACACAATGGACGTATGGTTAAACTGGGGATATGGTAATCTAGCCATTCACTATAACGGCCGACATGAGAG	73	585	6	65.49	749	1	0							
N/A	GGGACACAATGGACGATGACGGGATTAACTTCTTTAACGGCCGACATGAGAG	52	585	6	65.49	794	1	0							
N/A	GGGACACAGTGGACGCTAGTAATAAAATACACATCTTAGTATCCGCTTGAAGGCGTAACGGCCGACATGAGAG	73	585	6	65.49	701	1	0							
N/A	GGGACACAATGGACGGAATAAATCATATAAGGGGAATCTAACGGCCGACATGAGAG	56	585	6	65.49	621	1	0							
N/A	GGGACACAATGGACGTGAGTTATAATACAAATACATATGTCAGGGTCTACAGGCTTAACGGCCGACATGAGAG	73	585	6	65.49	647	1	0							
N/A	GGGACACAATGGACGGAGTACTAATCGAATGAACTGGGTTTATGTTCTTGAATGGTAACGGCCGACATGAGAG	73	585	6	65.49	606	1	0							
N/A	GGGACACAATGGACGCGATGTGTATATTGAGTACCAAATATAGATGAGTTGACCTTAACGGCCGACATGAGAG	73	585	6	65.49	692	1	0							
N/A	GGGACACAATGGACGCTGTGGATATTTTCAAGACTGTTATGAAGACGTAATGAATTAACGGCCGACATGAGAG	73	585	6	65.49	574	1	0							
N/A	GGGACACAATGGACGTTTGTATTAATTGATGTGAAACACTTGCAGTGTTGAGCTATAACGGCCGACATGAGAG	73	585	6	65.49	627	1	0							
N/A	GGGACACAATGGACGTATTACACATTACATTAAATGACCTAACACATGAGCAGGCTAACGGCCGACATGAGAG	73	585	6	65.49	609	1	0							
N/A	GGGACACAATGGACGGCTTCAAAATGATTCTGTGATGATTGATTAAGTCATCTTGTAACGGCCGACATGAGAG	73	585	6	65.49	777	1	0							
N/A	GGGACACAATGGACGTATGGCTTTTTTATGTTATATGGATTCGAATGTAGGGCTATAACGGCCGACATGAGAG	73	585	6	65.49	668	1	0							
N/A	GGGACACAATGGACGATATAAATATGCTAAGTGGAATCAACTCGAATGCAGGGCTTAACGGCCGACATGAGAG	73	585	6	65.49	616	1	0							
N/A	GGGACACAATGGACGGAAGCATAGGGTATACAGTTTTTGTTATAAGTATGTAAGCTAACGGCCGACATGAGAG	73	585	6	65.49	611	1	0							
N/A	GGGACACAATGGACGCAGTCTTTGAAGTAAATTGGACATGAAGAATAAGAACGACTAACGGCCGACATGAGAG	73	585	6	65.49	796	1	0							
N/A	GGGACACAATGGACGCAATACTATAATGATAATTTGGTAATAAAAATGACATATATAACGGCCGACATGAGAG	73	585	6	65.49	649	1	0							
N/A	GGGACACAATGGACGATACTCATTCACGCATTTGAACTATATTTACTTGAGGACGTAACGGCCGACATGAGAG	73	585	6	65.49	586	1	0							
N/A	GGGACACAATGGACGTTTAAGTCAGTATGAAGGTATCAATAACGTAAACTGCACATAACGGCCGACATGAGAG	73	585	6	65.49	711	1	0							
N/A	GGGACACAATGGACGAATAAGATATTTAGTTTAGCGTGAACATGGTGATGATGGCTAACGGCCGACATGAGAG	73	585	6	65.49	680	1	0							
N/A	GGGACACAATGGACGTTTTCATATTTTCGAACAATCAACTCAAAGAGAAGCTGGGTAACGGCCGACATGAGAG	73	585	6	65.49	719	1	0							
N/A	GGGACACAATGGACGATTAGGTTTGAGTGAATGTTATTTCCGGGAATCAAGGCGATAACGGCCGACATGAGAG	73	585	6	65.49	678	1	0							
N/A	GGGACACAATGGACGTATTACAGTAAATATATGCTATTGCCACACGTCAGACCGGTAACGGCCGACATGAGAG	73	585	6	65.49	760	1	0							
N/A	GGGACACAATGGACGGTTGTTATGCACCAAATTATGGACGGAAGGTTTAGATTATTAACGGCCGACATGAGAG	73	585	6	65.49	612	1	0							
N/A	GGGACACAATGGACGTATTTAATATTGAGTTGTCGTAACGGCCCGTGAGTAAATCTAACGGCCGACATGAGAG	73	585	6	65.49	618	1	0							
N/A	GGGACACAATGGACGCATAACGTAAATTCGAACATGTAACACCAAAAGATTGGCTTAACGGCCGACATGAGAG	73	585	6	65.49	610	1	0							
N/A	GGGACACAATGGACGTATATTTTAATGGTGTGAGAATACCAGACAAGTTGGGCTTTAACGGCCGACATGAGAG	73	585	6	65.49	623	1	0	2055	4	27.4	2685	1	0	0.4184
N/A	GGGACACAATGGACGGA	17	585	6	65.49	3	13	3	1398	5	34.25	1	17	3	0.523
N/A	GGGACACAATGGACGT	16	585	6	65.49	3	14	2	756	7	47.94	1	14	2	0.732
N/A	GGGACACAATGGACGTATAAACACTTTGTATCAAATCATAGAAGCTGTTAGCGGATAACGGCCGACATGAGAG	73	585	6	65.49	660	1	0	568	8	54.79	555	1	0	0.8366
N/A	GGGACACAATGGACGTGTAGATGATAACTAAAATTGTATATCCGATGTTTGGGCTTAACGGCCGACATGAGAG	73	585	6	65.49	737	1	0	324	10	68.49	378	1	0	1.0458
N/A	GGGACACAATGGACGCTTATGATTAATATAAGAGTTGGTATCTGTCACGAAGGCTTAACGGCCGACATGAGAG	73	585	6	65.49	681	1	0	196	12	82.19	244	1	0	1.255
N/A	GGGACACAATGGACGATGGATTATTTACACAACATAAGATCTCAGATAGGGACGGTAACGGCCGACATGAGAG	73	818	5	54.58	917	1	0							
N/A	GGGACACAATGGACGAGTTTATTCTAAATTAGCGTCTCGTTGGCTATAACGGCCGACATGAGAG	64	818	5	54.58	1121	1	0							
N/A	GGGACACAATGGACGCAAGTTAGCCTAGCAGTTTATATATTAGCTGGAAAACGTAACGGCCGACATGAGAG	71	818	5	54.58	842	1	0							
N/A	GGGACACAATGGACGCTGTAAAAAATCCTTGTATATAAAATCTTAATGCCTGGCGTAACGGCCGACATGAGAG	73	818	5	54.58	981	1	0							
N/A	GGGACACAATGGACGGTACGAGGCTATATTAGAGTTTATTTATATGTAGTACAGATAACGGCCGACATGAGAG	73	818	5	54.58	851	1	0							
N/A	GGGACACAATGGACGGACTCGTAGTTCTACATGAATACAGAAGCACATGGAATGCTAACGGCCGACATGAGAG	73	818	5	54.58	869	1	0							
N/A	GGGACACAATGGACGAGTTGTAGAATCTACAGTGTATTTAGAGGACGTAGATAGGTAACGGCCGACATGAGAG	73	818	5	54.58	959	1	0							
N/A	GGGACACAATGGACGTTTTAGAGCGACAAAACCAACATATATATCCCTGTATGGCTTAACGGCCGACATGAGAG	74	818	5	54.58	1143	1	0							
N/A	GGGACACAATGGACGTTGGCATTTGAAGAGATGGTAGATTAATAGTAGCCATACATAACGGCCGACATGAGAG	73	818	5	54.58	982	1	0							
N/A	GGGACACAATGGACGCAAACACCTAAATCACGGTTAAATCACTACTTGAATGGTGTAACGGCCGACATGAGAG	73	818	5	54.58	965	1	0							
N/A	GGGACACAATGGACGTTGGATTTAAAGGCAGAAATTAGCTAACGGCCGACATGAGAG	57	818	5	54.58	1043	1	0							
N/A	GGGACACAATGGACGATATAGTTGGCTGTTTCTGAGGATAATATAGCTTTCTTTATAACGGCCGACATGAGAG	73	818	5	54.58	811	1	0							
N/A	GGGACACAATGGACGGTGATATACGAATTTAATCATAGGCAAGATTATACGAATATAACGGCCGACATGAGAG	73	818	5	54.58	1050	1	0							
N/A	GGGACACAATGGACGAGACAATTGATTAAGATCTGAGTGGAAAATTAAGTTGGGCTAACGGCCGACATGAGAG	73	818	5	54.58	1097	1	0							
N/A	GGGACACAATGGACGGAAGTCGTCAATAGAGAGCAATTAGAGACATTTATGGGCGTAACGGCCGACATGAGAG	73	818	5	54.58	1079	1	0							
N/A	GGGACACAATGGACGGTATTATGGACAAAGATGCAAATTGACAACTAAGAGTCATAACGGCCGACATGAGAG	72	818	5	54.58	1019	1	0							
N/A	GGGACACAATGGACGGTATTCGTACAACGCCGATGATTATTATTTTAGGGCATGTAACGGCCGACATGAGAG	72	818	5	54.58	990	1	0							
N/A	GGGACACAATGGACGGGAGTTAAAAGGTATATAAGTTGAACTGAACTTGAAGCACTAACGGCCGACATGAGAG	73	818	5	54.58	886	1	0							

N/A	GGGACACAATGGACGGCTAATTATTAAGTTGTATTATTAAACGATTAACGGCCGACATGAGAG	63	818	5	54.58	863	1	0
N/A	GGGACACAATGGACGTAAGCATAAAAACGTGATATTACTTAGCTGTATTAGAGGCTAACGGCCGACATGAGAG	73	818	5	54.58	967	1	0
N/A	GGGACACAATGGACGATGATGAATCAATTGATACTATAACGGCCGACATGAGAG	54	818	5	54.58	899	1	0
N/A	GGGACACAATGGACGTTTTACGAAAACACTCTAAGGTATCTACGTGGATGGCACTTAACGGCCGACATGAGAG	73	818	5	54.58	825	1	0
N/A	GGGACACAATGGACGAAAAGACAAGAGTAAAAATTTTCATATTGACAGCGTCATCTTAACGGCCGACATGAGAG	74	818	5	54.58	1028	1	0
N/A	GGGACACAATGGACGTTAAAATATACATCTGTTCATCCTTCAAGTGTGCAGGTATAACGGCCGACATGGAGAG	73	818	5	54.58	925	1	0
N/A	GGGACACAATGGACGTAGACGGGTAATTTAATGATTAACAAGTATAAACAAACGTTAACGGCCGACATGAGAG	73	818	5	54.58	1063	1	0
N/A	GGGACACAATGGACGGTTTTAATGTAGAAGGTATACGATTATTACGAGACTTCGTTAACGGCCGACATGAGAG	73	818	5	54.58	820	1	0
N/A	GGGACACAATGGACGCAGATATTATGCTAGATGTATATTGTTAAGCCTTGGCCATAACGGCCGACATGAGAG	72	818	5	54.58	808	1	0
N/A	GGGACACAATGGACGGGGTTAATATATGTCTGCTCGAGATTGAACAGAGTTAAGATAACGGCCGACATGAGAG	73	818	5	54.58	1014	1	0
N/A	GGGACACAATGGACGGAGTAGAAGTTTGCGAGTTTAATTCAACTGCATTTGTTCGTAACGGCCGACATGAGAG	73	818	5	54.58	1144	1	0
N/A	GGGACACAATGGACGGACTATATAACTGCGTACAAGTAATAAATGTCGCTTTAGGTAACGGCCGACATGAGAG	73	818	5	54.58	1045	1	0
N/A	GGGACACAATGGACGGTCAGAGACAAATTGAGTTATTTCAAAACGTAACGGCCGACATGAGAG	63	818	5	54.58	985	1	0
N/A	GGGACACAATGGACGGTTTTACGGCATATTTTATGGATAACGGCCGACATGAGAG	55	818	5	54.58	1075	1	0
N/A	GGGACACAATGGACGGTGTTAATCAGGATTAAATAAATGCTTTACGAGGCGCTGGTAACGGCCGACATGAGAG	73	818	5	54.58	1127	1	0
N/A	GGGACACAATGGACGTCGAAAGCGGGAAAGACGAGTAAAAATGTTAAATCTTGGCTAACGGCCGACATGAGAG	73	818	5	54.58	908	1	0
N/A	GGGACACAATGGACGATATTACTAACTAATATAACACACGAAGTGGATCAAGGCTTAACGGCCGACATGAGAG	73	818	5	54.58	960	1	0
N/A	GGGACACAATGGACGGAACAATACGCATGTAATAGCTGGAAAACTGATTAAGCGGTAACGGCCGACATGAGAG	73	818	5	54.58	856	1	0
N/A	GGGACACAATGGACGCGGAGCTTGGTCAAGATATAAAAATCGATAAAAAAAAGCCGAGATAACGGCCGACATGAG	77	818	5	54.58	652	2	1
N/A	GGGACACAATGGACGAGTAATTGAGTCACTTATTAAGAGTGGCCAGAGTGCCGCTAACGGCCGACATGAGAG	72	818	5	54.58	1123	1	0
N/A	GGGACACAATGGACGTTAACCTAGATCGCATAACTTATTGCTTTTAGTGCGCAGGTAACGGCCGACATGAGAG	73	818	5	54.58	992	1	0
N/A	GGGACACAATGGACGAGTAAAACGTAGAGTATCAATATTGTGTCGATTCTGCTGGTAACGGCCGACATGAGAG	73	818	5	54.58	1137	1	0
N/A	GGGACACAATGGACGGAGAGGTTTATGTTAGGCAATCAAAATATCGTAGCATATATAACGGCCGACATGAGAG	73	818	5	54.58	830	1	0
N/A	GGGACACAATGGACGTAAGTTATTACAATCGTTGCTGTTGATACCAGTTGGCACATAACGGCCGACATGAGAG	73	818	5	54.58	1118	1	0
N/A	GGGACACAATGGACGGTAAAGTAAAATTAGAATGTAAAGGTCGCTTGACTACAGTTAACGGCCGACATGAGAG	73	818	5	54.58	900	1	0
N/A	GGGACACAATGGACGTATTAATTATATATCAACTATGTCTGATGAACGGGCGGACTAACGGCCGACATGAGAG	73	818	5	54.58	852	1	0
N/A	GGGACACAATGGACGATACTAGGAACAAACGAATGAAGACAATTTTGGGTTTGAGTAACGGCCGACATGAGAG	73	818	5	54.58	803	1	0
N/A	GGGACACAATGGACGAGACCTTAGATAACCAAATTTAAAGAAGATAATTGGTGGCTAACGGCCGACATGAGAG	73	818	5	54.58	1035	1	0
N/A	GGGACACAATGGACGGTTTATAACGGCCGACATGAGAG	38	818	5	54.58	279	6	4
N/A	GGGACACAATGGACGCTTCTATGATGTACTACAATACTGATGGCACTATTTAAATAACGGCCGACATGAGAG	72	818	5	54.58	944	1	0
N/A	GGGACACAATGGACGCGAAATGATTGAATTTACTACGTGGATATTGTCCGTCGGATAACGGCCGACATGAGAG	73	818	5	54.58	1024	1	0
N/A	GGGACACAATGGACGAAATCAAATTCAAGTAACGATTATAACCCGCCCCGGCCTTAACGGCCGACATGAGAG	72	818	5	54.58	1021	1	0
N/A	GGGACACAATGGACGGACTATGTTGGTAAAGTATTTAATGAACCTTTCGAAGACGTAACGGCCGACATGAGAG	73	818	5	54.58	988	1	0
N/A	GGGACACAATGGACGGTCGGAGATAAATAGCGAGATATTATTTAAAGCAAGCTGCTAACGGCCGACATGAGAG	73	818	5	54.58	958	1	0
N/A	GGGACACAATGGACGAGAATATATACAAAAGTGGTTTAATTGACTGTATAAGTACTAACGGCCGACATGAGAG	73	818	5	54.58	1108	1	0
N/A	GGGACACAATGGACGCAGGAACTGACCAATTCGTAAGAATTGCAGATTTAAATACTAACGGCCGACATGAGAG	73	818	5	54.58	923	1	0
N/A	GGGACACAATGGACGCTATCAGTCGGAATTTATGTAGATATTAATACAACTATGGTAACGGCCGACATGAGAG	73	818	5	54.58	1064	1	0
N/A	GGGACACAATGGACGCAGAAAAAAAAAGATTGGTAAATTATCTTAACGGCCGACATGAGAG	61	818	5	54.58	978	2	1
N/A	GGGACACAATGGACGTAATAAATATACATGGCGAATGAAAGAACGATGAAAGTACTAACGGCCGACATGAGAG	73	818	5	54.58	1009	1	0
N/A	GGGACACAATGGACGAGTTGGCTTTAGACAGATAACAGTTATTATACTGTAACGGCCGACATGAGAG	67	818	5	54.58	1116	1	0
N/A	GGGACACAATGGACGCTAATGAAGTGTGGATGCTATTATGCAGGATCTATATCTATAACGGCCGACATGAGAG	73	818	5	54.58	932	1	0
N/A	GGGACACAATGGACGTTACATGCAGTACTTTTATAGATCATCACCTGTTATGGTGTAACGGCCGACATGAGAG	73	818	5	54.58	1015	1	0
N/A	GGGACACAATGGACGGTTGACC	22	818	5	54.58	707	4	4
N/A	GGGACACAATGGACGCATTAGAACTTACACTCACTTATAGGAGTATAACAGAGGCTAACGGCCGACATGAGAG	73	818	5	54.58	913	1	0
N/A	GGGACACAATGGACGGATAGAAACTTAGATATGTCGAATGAAAGGCATTTTAGGGTAACGGCCGACATGAGAG	73	818	5	54.58	914	1	0
N/A	GGGACACAATGGACGTACAACAACTCTTGATACTATGGAACAATGGGAGTTTGAATAACGGCCGACATGAGAG	73	818	5	54.58	947	1	0
N/A	GGGACACAATGGACGGGAACTTAATCTATTGCCTTGCGGCTATGATATCAAAAGGTAACGGCCGACATGAGAG	73	818	5	54.58	1124	1	0
N/A	GGGACACAATGGACGAAGATTTGACCATAGAGGTGCGTACCAAAAAAAAAAGAGATGGTAACGGCCGACATGAGA	76	818	5	54.58	840	1	0
N/A	GGGACACAATGGACGATGGAATAAAAACACACTGTTGGCATGTGATATGCAAGTATAACGGCCGACATGAGAG	73	818	5	54.58	1067	1	0
N/A	GGGACACAATGGACGGTATAGGATTTGTATGATTGTATAGTTACTTTATCAATCTAACGGCCGACATGAGAG	72	818	5	54.58	841	1	0
N/A	GGGACACAATGGACGGAAGCGGTGACAAAATAACTAACAGAATATATAACCTGCTAACGGCCGACATGAGAG	72	818	5	54.58	866	1	0
N/A	GGGACACAATGGACGGTAATTAGTCATCAAAATTTTATTATGGACTGTTTGGGCTAACGGCCGACATGAGAG	72	818	5	54.58	922	1	0
N/A	GGGACACAATGGACGAGACAATTTCAACATACAACTAAAACACCTATGGTGGCTGTAACGGCCGACATGAGAG	73	818	5	54.58	1052	1	0
N/A	GGGACACAATGGACGGTACGTATGAACGGGGCAACGAAGTAATTTTCACTTCTAGTAACGGCCGACATGAGAG	73	818	5	54.58	889	1	0
N/A	GGGACACAATGGACGCTGCACCTCGATTGAAGATACTAACTACATCTCAAATCAGTAACGGCCGACATGAGAG	73	818	5	54.58	1087	1	0
N/A	GGGACACAATGGACGGTTGGTACAGAATACCTTGTTAATTATCGCAAGACTACCGTAACGGCCGACATGAGAG	73	818	5	54.58	837	1	0
N/A	GGGACACAATGGACGCTTGTATTTAATGAAACGATCTCAAATGAAGAGATGCGAATAACGGCCGACATGAGAG	73	818	5	54.58	1068	1	0
N/A	GGGACACAATGGACGGAATGGCATTATTTCATCTATACGGTAGACCAAAGTTGGCTAACGGCCGACATGAGAG	73	818	5	54.58	1140	1	0
N/A	GGGACACAATGGACGGCTGAATAGATGAAAGCACTATAGTTTTTGGATTGACCAATAACGGCCGACATGAGAG	73	818	5	54.58	834	1	0
N/A	GGGACACAATGGACGCAAGAAAATTGAAGATAAAGGCTGGACTGTAACTATTTGATAACGGCCGACATGAGAG	73	818	5	54.58	1141	1	0

N/A	GGGACACAATGGACGGATTCTAATTATATAGGGGTCTGGTTGAATGTTGAGCTGGTAACGGCCGACATGAGAG	73	818	5	54.58	1051	1	0
N/A	GGGACACAATGGACGAATTAACAATATGTCACTGTACAAGCAGATTGGCTGAAGATAACGGCCGACATGAGAG	73	818	5	54.58	857	1	0
N/A	GGGACACAATGGACGTTTTACTTAGCAATGAGTAATGATGATATCGTTTTAGGTATAACGGCCGACATGAGAG	73	818	5	54.58	940	1	0
N/A	GGGACACAATGGACGGTGACAATGAACTGTGATAAACACAAATATATCATAAGTTAACGGCCGACATGAGAG	72	818	5	54.58	1109	1	0
N/A	GGGACACAATGGACGATTACGAGCTACAGGAGTTTATATTACTTCAATGTTACCGTAACGGCCGACATGAGAG	73	818	5	54.58	997	1	0
N/A	GGACACAATGGACGCTGTATTACTATATATTTAGAAGTAACGGCCGACATGAGAG	55	818	5	54.58	1101	1	0
N/A	GGGACACAATGGACGACTATTCAATTTCGAATAACTCTACATTATGAGTCAGGCGTAACGGCCGACATGAGAG	73	818	5	54.58	813	1	0
N/A	GGGACACAATGGACGTATAGTTTGAAGGTTTGGTAAGACTAACGGCCGACATGAGAG	57	818	5	54.58	935	1	0
N/A	GGGACACAATGGACGTTTTAAAATAGCACTCTGACTAAGAATAGCTAAGTAGAAGTAACGGCCGACATGAGAG	73	818	5	54.58	1018	1	0
N/A	GGGACACAATGGACGCGGAAACTATTTAATTGATGACCGTAAGGATTTATCGGAGGTAACGGCCGACATGAGAG	74	818	5	54.58	1154	1	0
N/A	GGGACACAATGGACGCAGTTAATAATTTCCTCGGCTATATTAGCGTGAGATAAGTATAACGGCCGACATGAGAG	74	818	5	54.58	1074	1	0
N/A	GGGACACAATGGACGCGACGATTATTTAATTTAAAGTATAACGGCCGACATGAGAG	56	818	5	54.58	1122	1	0
N/A	GGGACACAATGGACGGAGAAGAAATTTTAGCAGGTGCAAAGCACATCTGGTTATCTAACGGCCGACATGAGAG	73	818	5	54.58	877	1	0
N/A	GGGACACAATGGACGTATAATTTAATTAGAAATGGGTTATCATAGGTCGTTTTAGTAACGGCCGACATGAGAG	73	818	5	54.58	1086	1	0
N/A	GGGACACAATGGACGGATTCTGGATCTAAGCTAAAAATTCAAGAGCTATGAACACTAACGGCCGACATGAGAG	73	818	5	54.58	929	1	0
N/A	GGGACACAATGGACGTATGTTATCTTATTGACGATAACGGCCGACATGAGAG	52	818	5	54.58	81	2	6
N/A	GGGACACAATGGACGTAGGAAATAATGACAATTGTATACCAGTATGCATGAGGCTAACGGCCGACATGAGAG	72	818	5	54.58	1138	1	0
N/A	GGGACACAATGGACGGAACAGCTTTACCAACTTAGTCTATATACTATAGGAGGTGTAACGGCCGACATGAGAG	73	818	5	54.58	1025	1	0
N/A	GGGACACAATGGACGTATCCACTATATATCATATCTTTTGAGACGTCATATAGGCTAACGGCCGACATGAGAG	73	818	5	54.58	1010	1	0
N/A	GGGACACAATGGACGGTTCAAATATAGAAAGCGTGGATACCACACGTTCTTACTATAACGGCCGACATGAGAG	73	818	5	54.58	921	1	0
N/A	GGGACACAATGGACGCTACTACATGATTTATACAATAGCACTAGATGTCAGGCTCTAACGGCCGACATGAGAG	73	818	5	54.58	989	1	0
N/A	GGGACACAATGGACGGTTGGTAACATGCATTTCTATTGAAATGAATTAGACTATGCTAACGGCCGACATGAGAG	74	818	5	54.58	879	1	0
N/A	GGGACACAATGGACGGTATGAATACTAAATTAGATTTATTAATGTGGGAAGACATAACGGCCGACATGAGAG	72	818	5	54.58	979	1	0
N/A	GGGACACAATGGACGAAGGATACAGCTAACCAATAAGGGTTTTTATGCATGATCTTAACGGCCGACATGAGAG	73	818	5	54.58	848	1	0
N/A	GGGACACAATGGACGTTTGCTTTAATAACGGTGTCTCGCAGCTGTTACCTAGTTGTAACGGCCGACATGAGAG	73	818	5	54.58	1066	1	0
N/A	GGGACACAATGGACGCCTAGCATGTATGGTACACAATAATAGTACATAACGGCCGACATGAGAG	64	818	5	54.58	1001	1	0
N/A	GGGACACAATGGACGTGAACACCGCATGTTTATTCATTTGTTTGAATGGGCAAGTAACGGCCGACATGAGAG	72	818	5	54.58	1105	1	0
N/A	GGGACACAATGGACGCATCACTTTTACAAATAAGATGTGAATCAATCACAGCCGGTAACGGCCGACATGAGAG	73	818	5	54.58	839	1	0
N/A	GGGACACAATGGACGGACGCAGACAATGAATTATATACAAACTCAAGATACTGACTAACGGCCGACATGAGAG	73	818	5	54.58	1042	1	0
N/A	GGGACACAATGGACGTGAAACGAATATCAAGGCTTACTACTCGCTAAACGATGGTAACGGCCGACATGAGAG	72	818	5	54.58	1078	1	0
N/A	GGGACACAATGGACGGACTAATCAATTAACTAGAGTTTTTAGTGACCGAGTAATGATAACGGCCGACATGAGAG	74	818	5	54.58	1102	1	0
N/A	GGGACACAATGGACGGTTAGTATTATGAATAAGAGTAACGGCCGACATGAGAG	53	818	5	54.58	826	1	0
N/A	GGGACACAATGGACGGTGGTAAGCTAGATTATGTATATGTTAAAGTCATTTTCATACTAACGGCCGACATGAGAG	75	818	5	54.58	1000	1	0
N/A	GGGACACAATGGACGTTAGATATTTTGACGTAACCAATTATGTGAGCGGCACAAGTAACGGCCGACATGAGAG	73	818	5	54.58	798	1	0
N/A	GGGACACAATGGACGCTAATATGTGCCGACAGATTGTATGAATAAGATCGGAAGGTAACGGCCGACATGAGAG	73	818	5	54.58	984	1	0
N/A	GGGACACAATGGACGAAACGTTCTGGCCGAATAATGATTAGTATTTATATCACTAACGGCCGACATGAGAG	71	818	5	54.58	1090	1	0
N/A	GGGACACAATGGACGATTCGAAATATGAATTAACATAAAGCCTGGCTTTACATAGTAACGGCCGACATGAGAG	73	818	5	54.58	996	1	0
N/A	GGGACACAATGGACGCTGATTGGTAACGAATGAGAAATAATATCTGTATTCTCCTAACGGCCGACATGAGAG	72	818	5	54.58	1038	1	0
N/A	GGGACACAATGGACGGTAGGTTATAACATGTTTGTATGAACAACTAATGTTGAATTAACGGCCGACATGAGAG	73	818	5	54.58	901	1	0
N/A	GGGGCACAATGGACGTTTTACATATATTGTAGAGGCAATTTTGATGAGTTGGGCATAACGGCCGACATGAGAG	73	818	5	54.58	797	1	0
N/A	GGGACACAATGGACGTAGAACTTGGAGTAAACGCATATATAGCGAAACTTGACGATAACGGCCGACATGAGAG	73	818	5	54.58	1020	1	0
N/A	GGGACACAATGGACGCAGTTAAATTGAGACATGGTACTATATATCACTGACGACTTAACGGCCGACATGAGAG	73	818	5	54.58	882	1	0
N/A	GGGACACAATGGACGTTACCTAACAATGGACTCGCTAACAGTTGGGTTCTGAATAACGGCCGACATGAGAG	71	818	5	54.58	928	1	0
N/A	GGGACACAATGGACGAAATCCAGTAACCCAACGGTTCGGATATATCAATTCGATATAACGGCCGACATGAGAG	73	818	5	54.58	964	1	0
N/A	GGGACACAATGGACGTAAATATATAATATTAACGGCCGACATGAGAG	47	818	5	54.58	415	2	5
N/A	GGGACACAATGGACGTTATACAAGAAG	27	818	5	54.58	756	2	6
N/A	GGGACACAATGGACGTACTATCAGGATTCTCTTAGGTTGTTAACTCTAAACGGGATAACGGCCGACATGAGAG	73	818	5	54.58	858	1	0
N/A	GGGACACAATGGACGTTATGTAAAATGAGATATTGAATATAGCCGGTTTTTGGAATAACGGCCGACATGAGAG	73	818	5	54.58	1033	1	0
N/A	GGGACACAATGGACGGAAATAAGATTTTTTTAATTTATAACGGCCGACATGAGAG	55	818	5	54.58	321	2	6
N/A	GGGACACAATGGACGTTCTTCTTTATGTGAAGCTAATATACCATTCTTAAATGGATAACGGCCGACATGAGAG	73	818	5	54.58	827	1	0
N/A	GGGACACAATGGACGTATATTCATCAATCCCATCGCATATTTAGGCTGGATTGGTAACGGCCGACATGAGAG	72	818	5	54.58	1003	1	0
N/A	GGGACACAATGGACGGCGATATTTTAGTATGATATCTCAACGAGACTGTGAAATAACGGCCGACATGAGAG	71	818	5	54.58	955	1	0
N/A	GGGACACAATGGACGTATCAATTAATGTGTATGTTGTAAGGCGCACGTAAAATGGTAACGGCCGACATGAGAG	73	818	5	54.58	845	1	0
N/A	GGGACACAATGGACGACATTCAATGATAAAAATGATGAACATATGGATTCTGGGCTTAACGGCCGACATGAGAG	74	818	5	54.58	814	1	0
N/A	GGGACACAATGGACGTTAATATGGATAAGAAACCAATTCGCATGGTAGTATTACTTAACGGCCGACATGAGAG	73	818	5	54.58	977	1	0
N/A	GGGACACAATGGACGCTGGTTTATGTAATACTTATAACGGCCGACATGAGAG	52	818	5	54.58	1059	1	0
N/A	GGGACACAATGGACGTCAAGTTGTCTGCCACTATTTAACATAATAGACCTATGGACAATAACGGCCGACATGAGAC	76	818	5	54.58	1113	1	0
N/A	GGGACACAATGGACGCTGTTGAATGACAAGGATTAAGTTTTAATAAGTTTCAGACTAACGGCCGACATGAGAG	73	818	5	54.58	971	1	0
N/A	GGGACACAATGGACGGTACCAAACTGCGGACGTAATGAAGTATAAAATTGCAAGCTAACGGCCGACATGAGAG	73	818	5	54.58	1002	1	0
N/A	GGGACACAATGGACGTTAGTATAAGCATTCACAACAACAGATCAAACTGGCACGCTAACGGCCGACATGAGAG	73	818	5	54.58	926	1	0

N/A	GGGACACAATGGACGTAGCAGAGTTTGAACGATGGAGTTTATAACACTCTTACTATAACGGCCGACATGAGAG	73	818	5	54.58	1017	1	0
N/A	GGGACACAATGGACGTAACAGCCGATATAAATTATATTAATTACAATATGGTAACGGCCGACATGAGAG	69	818	5	54.58	895	1	0
N/A	GGGACACAATGGACGAGGTTTATACATATGATTATTATCTTCAAAGGATCATGAGTAACGGCCGACATGAGAG	73	818	5	54.58	1119	1	0
N/A	GGGACACAATGGACGATAAAAGGATGATCTAAGTATAACATAGAGTCTACTAACGGCCGACATGAGAG	68	818	5	54.58	1060	1	0
N/A	GGGACACAATGGACGGTACAATTATTCTGCGATAGGGCAATTAGAGCGTTTACAATAACGGCCGACATGAGAG	73	818	5	54.58	884	1	0
N/A	GGGACACAATGGACGTGCTCGGCAATATATTCTGTAATATAAGAGATGTATTAACGGCCGACATGAGAG	69	818	5	54.58	1049	1	0
N/A	GGGACACAATGGACGTATTAGACAACACATGATGATTACTGTCAGGTGGGTTATATAACGGCCGACATGAGAG	73	818	5	54.58	966	1	0
N/A	GGGACACAATGGACGGTAGTACATATTATAATTATAACGGCCGACATGAGAG	52	818	5	54.58	860	1	0
N/A	GGGACACAATGGACGCTGGAGCTTTGGACTAAAATGTGCAAAGACGATATTTAATTAACGGCCGACATGAGAG	73	818	5	54.58	846	1	0
N/A	GGGACACAATGGACGAATATTAAGGAATTAGGTTTATTAGTGCCTGATGGTAGGCTAACGGCCGACATGAGAG	73	818	5	54.58	1088	1	0
N/A	GGGACACAATGGACGTTATTACCACAATTGATCTTAGCTTTAAAATGCGAAGAGATAACGGCCGACATGAGAG	73	818	5	54.58	1103	1	0
N/A	GGGACACAATGGACGGTCATTTATTACTTTGTAGGGTTTTCTACTTAAGAGTAACGGCCGACATGAGAG	69	818	5	54.58	853	1	0
N/A	GGGACACAATGGACGGACACGTAAGG	26	818	5	54.58	756	4	5
N/A	GGGACACAATGGACGTGCTAGTTTAGAGTAGGTTGTTAATTTAAATACTAGATCTAACGGCCGACATGAGAG	72	818	5	54.58	1004	1	0
N/A	GGGACACAATGGACGGAGTGAAGACGGAAGGTAAATTAGATACAATTCGTTACTGTAACGGCCGACATGAGAG	73	818	5	54.58	952	1	0
N/A	GGGACACAATGGACGTGATATTCCAGATCTGCCAATAACATGCTATCATAGAGTTTAACGGCCGACATGAGAG	73	818	5	54.58	1054	1	0
N/A	GGGACACAATGGACGTTTAGATGAAGAGTTTAGATTAAAATACTCGGAGAGCTACTAACGGCCGACATGAGAG	73	818	5	54.58	1112	1	0
N/A	GGGGCACAATGGACGAGTCTCGGAATTGGCTTATATATAACGGCCGACATGAGAG	55	818	5	54.58	951	1	0
N/A	GGGACACAATGGACGGACCGAATTAGTAGCAAGTTTTATATGGATAACTATAGGATAACGGCCGACATGAGAG	73	818	5	54.58	835	1	0
N/A	GGGACACAATGGACGACATCGTTAATATATCATCCGGAATCTTAGATAAATATGCTAACGGCCGACATGAGAG	73	818	5	54.58	861	1	0
N/A	GGGACACAATGGACGTAGTATGAAATTGTTTTAGATAGAGAACTGAGAAGGATCATAACGGCCGACATGAGAG	73	818	5	54.58	919	1	0
N/A	GGGACACAATGGACGTATAATTAACATTCAAATGAACCAGTAATGGGACGGTCGATAACGGCCGACATGAGAG	73	818	5	54.58	868	1	0
N/A	GGGACACAATGGACGCATGAATCAATGTTGATGGTACAATATACGCACCGACTGATAACGGCCGACATGAGAG	73	818	5	54.58	999	1	0
N/A	GGGACACAATGGACGGACACGTTGTAGATATTATATTTAACGGCCGACATGAGAG	55	818	5	54.58	822	1	0
N/A	GGGACACAATGGACGGTCTGGTAGGGTAATTTACTAGTTGGTTATACTTAGTACCTAACGGCCGACATGAGAG	73	818	5	54.58	1120	1	0
N/A	GGGACACAATGGACGTGACTTTTAAAATTGAGACTCAGAGCTGTTATTTTTGAGATAACGGCCGACATGAGAG	73	818	5	54.58	934	1	0
N/A	GGGACACAATGGACGGAATACATATGCTAACAATCTGCAGAAGATTGCACGTGATTAACGGCCGACATGAGAG	73	818	5	54.58	887	1	0
N/A	GGGACACAATGGACGTTTAAGACAAGTTCTGATAATATTTGAGAGGAACAACAATTAACGGCCGACATGAGAG	73	818	5	54.58	809	1	0
N/A	GGGACACAATGGACGATTTAAACCTATTTGTGAGTTGACAATGTTGAGATGCCGGTAACGGCCGACATGAGAG	73	818	5	54.58	931	1	0
N/A	GGGACACAATGGACGCTATATTTCACAACCTGCGGATTTTGGACAAAGGGAAGTGTAACGGCCGACATGAGAG	73	818	5	54.58	1125	1	0
N/A	GGGACACAATGGACGAATTAATATAGAAGGTATATATGAACCTTGTAGAGGCACTAACGGCCGACATGAGAG	72	818	5	54.58	1130	1	0
N/A	GGGACACAATGGACGAGTCGGAGACTGATACTGTATATATGAAGTACTTTCGGTTAACGGCCGACATGAGAG	72	818	5	54.58	939	1	0
N/A	GGGACACAATGGACGTCGAACAATGCATGCAGGTTTTATTAACAATCTAGCGAGGTAACGGCCGACATGAGAG	73	818	5	54.58	910	1	0
N/A	GGGACGCAATGGACGATAGTTAATGGACTTCTAGTTGTCACCATTCAGTGAGGCTTAACGGCCGACATGAGAG	73	818	5	54.58	865	1	0
N/A	GGGACACAATGGACGTAAACTAAGAACGCAACATTCGATTCCATGAATGGGCCTTAACGGCCGACATGAGAG	72	818	5	54.58	1155	1	0
N/A	GGGACACAATGGACGATACAGTAAAACCTGGTAATTGATATTACAAGACGAAGGTAACGGCCGACATGAGAG	72	818	5	54.58	844	1	0
N/A	GGGACACAATGGACGAGCTAGTAATTTTATACTAATTCCGGCAGTACGGTCAACGTAACGGCCGACATGAGAG	73	818	5	54.58	920	1	0
N/A	GGGACACAATGGACGAATGACGGACTGTTAGGTATGATTAATATACTATCTTTTCTAACGGCCGACATGAGAG	73	818	5	54.58	909	1	0
N/A	GGGACACAATGGACGCCAAAATAATTGAACAAAGTTAACGGCCGACATGAGAG	53	818	5	54.58	1072	1	0
N/A	GGGACACAATGGACGGGTTACCGCTAACTAATATATAGACAACATTAGCAAAGCTTAACGGCCGACATGAGAG	73	818	5	54.58	843	1	0
N/A	GGGACACAATGGACGACTGAATAAACTTCATGTATTCCAAACAATGTATGAGCCGTAACGGCCGACATGAGAG	73	818	5	54.58	1084	1	0
N/A	GGGACACAATGGACGTTATCGAAACAAAAATTATAGAGCTAACGGCCGACATGAGAG	57	818	5	54.58	963	1	0
N/A	GGGACACAATGGACGTTTCTTCAACCTAATATGGGCACTAAAGCGCATAATGGGATAACGGCCGACATGAGAG	73	818	5	54.58	810	1	0
N/A	GGGACACAATGGACGATGAATACAAGCAAATTTCTACGTTGTTAGTACTAACGGCCGACATGAGAG	66	818	5	54.58	902	1	0
N/A	GGGACACAATGGACGTAAGTAATGGTATTTAACTACACATGGACCAGTTCGGCACTAACGGCCGACATGAGAG	73	818	5	54.58	1091	1	0
N/A	GGGACACAATGGACGACAAAATTATTTATGTCTATACATACTCGATATGCGGGCTTAACGGCCGACATGAGAG	73	818	5	54.58	968	1	0
N/A	GGGACACAATGGACGTAAAGAAGTTATATTATACTCGTGAGAGGTAAAATCTTGTAACGGCCTGACATGAGAG	73	818	5	54.58	870	1	0
N/A	GGGACACAATGGACGATACAACTTAGTATGCTTATAAGATGATTATTAGCGAAGGTAACGGCCGACATGAGAG	73	818	5	54.58	821	1	0
N/A	GGGACACAATGGATGGTCGCATTTTTAGACAGCAAAAATATTTCTGCGGATTTCTAACGGCCGACATGAGAG	72	818	5	54.58	1153	1	0
N/A	GGGACACAATGGACGTTACACGATATTTTCTTTCCAGACTCTTGTTATGGAAGCGTAACGGCCGACATGAGAG	73	818	5	54.58	980	1	0
N/A	GGGACACAATGGACGGAGTTTCACTTACATGACTTGAACTAACGGCCGACATGAGAG	57	818	5	54.58	987	1	0
N/A	GGGACACAATGGACGCCGAAAAGTTAATTGGACGTTTTTCGTAGACGCGAAAATATAACGGCCGACATGAGAG	73	818	5	54.58	812	1	0
N/A	GGGACACAATGGACGCGACGAAAGAGAA	28	818	5	54.58	756	3	2
N/A	GGGACACAATGGACGTTAGAAATTAGTATATACATATTATGATAACATGGTACGCTAACGGCCGACATGAGAG	73	818	5	54.58	1047	1	0
N/A	GGGACACAATGGACGTACCAATTTGCTGAATAATAAACATTTCATCAAGGCAGGGTAACGGCCGACATGAGAG	73	818	5	54.58	880	1	0
N/A	GGGACACAATGGACGCAACGTATAT	25	818	5	54.58	707	3	6
N/A	GGGACACAATGGACGCTGAAAACTAAGAAAAATGAACTGTATATGTAATAACGGCCGACATGAGAG	66	818	5	54.58	1027	1	0
N/A	GGGACACAATGGACGCAGTTAAATGTATATTAGATCTAAGGTTGATACATGTATGTAACGGCCGACATGAGAG	73	818	5	54.58	1111	1	0
N/A	GGACACAATGGACGTGTAATATTATGTAACGGCCGACATGAGAG	44	818	5	54.58	995	1	0
N/A	GGGACACAATGGACGGGATTAGTATAAAGATAAGTTTACAAAACTATATCGATAGTAACGGCCGACATGAGAG	73	818	5	54.58	1040	1	0

N/A	GGGACACAATGGACGATTGTATTTAATATAACTAAGTCGTGGCCAACTGGGCTTTTAACGGCCGACATGAGAG	73	818	5	54.58	1095	1	0
N/A	GGGACACAATGGACGTTTTAATGTCAAGTGCGGGCGTAATATGTACAGAGATCATTAACGGCCGACATGAGAG	73	818	5	54.58	850	1	0
N/A	GGGACACAATGGACGCAATAGTTAATGTATAAGAGTAGAAGTTTCTTTTACAGCTAACGGCCGACATGAGAG	72	818	5	54.58	1007	1	0
N/A	GGGACACAATGGACGAACTGTTCTTGCGAGCTGTTATGGTTACTAGAATGTAACATAACGGCCGACATGAGAG	73	818	5	54.58	898	1	0
N/A	GGGACACAATGGACGGACCTTAGAAAGTTTAAGAAGGAATTTTTAAAGCGTACTATAACGGCCGACATGAGAG	73	818	5	54.58	976	1	0
N/A	GGGACACAATGGACGGAAAGTGAATATACAGTATACAAATCGTATACTTAGTAGTAACGGCCGACATGAGAG	72	818	5	54.58	918	1	0
N/A	GAGACACAATGGACGTCGAATGTAGGAAGGAAATATATTTGTACTTTTCTAAGGATAACGGCCGACATGAGAG	73	818	5	54.58	949	1	0
N/A	GGGACACAATGGACGACCGTGTATAATACATCTACTTAACACTTTTCATAGTAGTAACGGCCGACATGAGAG	72	818	5	54.58	1012	1	0
N/A	GGGACACAATGGACGATTGATTAAATGGGTCGAAAGACCTTATTGCTAACGGCCGACATGAGAG	64	818	5	54.58	801	1	0
N/A	GGGACACAATGGACGTAAAGTAAGGCAAAATTACATACAGAGAGATATTTGTAAGTAACGGCCGACATGAGAG	73	818	5	54.58	1037	1	0
N/A	GGGACACAATGGACGGAAGTTATTACTATATTTTAGCTAACGGCCGACATGAGAG	55	818	5	54.58	1031	1	0
N/A	GGGACACAATGGACGTTAATGAGTATTCAATGTTTTTACAGACGCTTGAAATGGCTAACGGCCGACATGAGAG	73	818	5	54.58	936	1	0
N/A	GGGACACAATGGACGGTACAATGTAGAACGGATGTGAGGTTAATTTAGCTTGCATAACGGCCGACATGAGAG	72	818	5	54.58	1139	1	0
N/A	GGGACACAATGGACGTCTTTATCAATAGGGCAATAGTAATATGATAGATCGAGGATAACGGCCGACATGAGAG	73	818	5	54.58	1080	1	0
N/A	GGGACACAATGGACGATAATGGTAGGCCAGTTTTATTGCATTAAGTATTTACTGCTAACGGCCGACATGAGAG	73	818	5	54.58	933	1	0
N/A	GGGACACAATGGACGATTGAAGGACGCATGGCGTATTGGTTATGTTTTAACGAGCTAACGGCCGACATGAGAG	73	818	5	54.58	1077	1	0
N/A	GGGACACAATGGACGTTAAACAGAAGTAGAGATCACGCTATAGGTATTTCAACTGTAACGGCCGACATGAGAG	73	818	5	54.58	1008	1	0
N/A	GGGACACAATGGACGTTGAGAGACAAATTAAATGGTGTATGCACACTTTAAAACTTAACGGCCGACATGAGAG	73	818	5	54.58	1104	1	0
N/A	GGGACACAATGGACGAACGATGAACAATATTAATCCAAAATGGAACTTAGAGGCTTAACGGCCGACATGAGAG	73	818	5	54.58	1011	1	0
N/A	GGGACACAATGGACGCGGGTTAATGAGATAAGGAACGAAGATCTCTTATTTGACGTAACGGCCGACATTAGAG	73	818	5	54.58	1089	1	0
N/A	GGGACACAATGGACGTTTTAAGAGTTTGACACTTGCGTTGTTCGGCTGGGAAAGTTAACGGCCGACATGAGAG	73	818	5	54.58	953	1	0
N/A	GGGACACAATGGACGTTGAAGATGGAGACTAAACATCAGGAAAATAGATTCATCATAACGGCCGACATGAGAG	73	818	5	54.58	1132	1	0
N/A	GGGACACAATGGACGCGAAGTTGATCAAATAAGTATAAAGACTCCCATTAATCGGTAACGGCCGACATGAGAG	73	818	5	54.58	1023	1	0
N/A	GGGACACAATGGACGGTTTTATTATGCAAACTCGGTGACGGGTAGTTTATTCAAATAACGGCCGACATGAGAG	73	818	5	54.58	1016	1	0
N/A	GGGACACAATGGACGTGGAGGTTTTAGTATTGGTGCCTTTAACTACTAAGTCATCATAACGGCCGACATGAGAG	74	818	5	54.58	1044	1	0
N/A	GGGACACAATGGACGTATCACATATATTATACAACATATCTGATGAGCCGCGCAATAACGGCCGACATGAGAG	73	818	5	54.58	912	1	0
N/A	GGGACACAATGGACGATATTAAAATGGCGGCTGAATAACCAGTATTTCTTATAGGTAACGGCCGACATGAGAG	73	818	5	54.58	1065	1	0
N/A	GGGACACAATGGACGAATGAATGACTTCTTTGGGTTCTCGATATAGTTGAGGCCCTAACGGCCGACATGAGAG	73	818	5	54.58	1100	1	0
N/A	GGGACACAATGGACGATAGTAATATTGGATAAACCCAAAATAGTAAGTTTAAGGCTTAACGGCCGACATGAGAG	74	818	5	54.58	816	1	0
N/A	GGGACACAATGGACGGTATTTATCAAGCCAAAATTATAACAATTCGCGAGGTAGTAACGGCCGACATGAGAG	72	818	5	54.58	824	1	0
N/A	GGGACACAATGGACGGTGTAGAGTTCTAATGATTATGTAACAATCGTAGATGAGTTAACGGCCGACATGAGAG	73	818	5	54.58	1006	1	0
N/A	GGGACACAATGGACGTTATCTAAGAAAATATGAAGAATATAGATCTGAAGTGATAACGGCCGACATGAGAG	71	818	5	54.58	1073	1	0
N/A	GGGACACAATGGACGTAAAAAGACACGAGGCCTTATATCGAAGAATTCATTCGGGTAACGGCCGACATGAGAG	73	818	5	54.58	1055	1	0
N/A	GGGACACAATGGACGTATCTTAATTAGACGTAATAATTGTACCGAATGTATGCCATAACGGCCGACATGAGAG	73	818	5	54.58	1131	1	0
N/A	GGGACACAATGGACGTTCTTTATATGTTAGTAATCCAACTGTAATGAGAACGAATAACGGCCGACATGAGAG	72	818	5	54.58	946	1	0
N/A	GGGACACAATGGACGATATTATTTTATGCACATCCCATGATTAATCGGCGGTAACTAACGGCCGACATGAGAG	73	818	5	54.58	1062	1	0
N/A	GGGACACAATGGACGGATAAGACATTAAATTAAGACCTTGCTCAAGTAGTCAGGTAACGGCCGACATGAGAG	72	818	5	54.58	906	1	0
N/A	GGGACACAATGGACGAGATGTCAGCTAATTTGTAGGGTATAATTTTATCTTGACTAACGGCCGACATGAGAG	72	818	5	54.58	1053	1	0
N/A	GGGACACAATGGACGCCACACTAAAGAACTAAAGATATAAAAGCCTTTAGGCAAGTAACGGCCGACATGAGAG	73	818	5	54.58	983	1	0
N/A	GGGACACAATGGACGAGTTGACCAAAGTGTAGAAGGCGTAGTTATAATGACTGTAACGGCCGACATGAGAG	71	818	5	54.58	1092	1	0
N/A	GGGACACAATGGACGGAACGGAAGGGTAATTCTTTAGTTAAAAGAAGCCGCTTATAACGGCCGACATGAGAG	72	818	5	54.58	954	1	0
N/A	GGGACACAATGGACGCTTGAAATAAATAACGCCGGATAAAGATTATGTTTGTTTATAACGGCCGACATGAGAG	73	818	5	54.58	957	1	0
N/A	GGGACACAATGGACGCGCTAATTATTTATAGTCGGTGAAGTAGTACATATTCACCTAACGGCCGACATGAGAG	73	818	5	54.58	881	1	0
N/A	GGGACACAATGGACGGGAAATGCGTAAGTTAGTTATTTATAGAAGTACTGACTGATAACGGCCGACATGAGAG	73	818	5	54.58	893	1	0
N/A	GGGACACAATGGACGTGATTTATTAACTTGACTGACGGAAGTGTTAGCGATGAACTAACGGCCGACATGAGAG	73	818	5	54.58	819	1	0
N/A	GGGACACAATGGACGTAGAAATATAAGCTAGAGATGTTTCGGAATTTGGTTGGCTTAACGGCCGACATGAGAG	73	818	5	54.58	1146	1	0
N/A	GGGACACAATGGACGGTAACGTAAGTATATCATATGTAATTTATATGACAGAAGATAACGGCCGACATGAGAG	73	818	5	54.58	915	1	0
N/A	GGGACACAATGGACGTGCAAGTCGGCTATTATTGAGGTATTTAGTATTGCTTGATTAACGGCCGACATGAGAG	73	818	5	54.58	805	1	0
N/A	GGGACACAATGGACGGTTTGGTCTTCAAGAACATTACAATAGGCTTGACTGACAGTAACGGCCGACATGAGAG	73	818	5	54.58	911	1	0
N/A	GGGACACAATGGACGGGAACGAACAACTGGGAGTTATATGATGTACTCTAAATTAGTAACGGCCGACATGAGAG	74	818	5	54.58	864	1	0
N/A	GGGACACAATGGACGAATGTCGATTCTGCATTAATGGCTTAATATCAACCAGTAATAACGGCCGACATGAGAG	73	818	5	54.58	888	1	0
N/A	GGGACACAATGGACGATATTCTTAAAAACTAAGGCGACTTATGGCACACGGCATGATAACGGCCGACATGAGAG	74	818	5	54.58	942	1	0
N/A	GGGACACAATGGACGAACAAAGATAAGGTTTGAACCCAAGTTGCACCAAAAAGGCTAACGGCCGACATGAGAG	73	818	5	54.58	970	1	0
N/A	GGGACACAATGGACGCAACCGATTAAGTTGGAAGAATACATAAGATTTTGATACATAACGGCCGACATGAGAG	73	818	5	54.58	1115	1	0
N/A	GGGACACAATGGACGGACAAAATCGGGTACGTTGTAAGCAAATATATTAACATGCTAACGGCCGACATGAGAG	73	818	5	54.58	883	1	0
N/A	GGGACACAATGGACGCTATAACTAATATGAAATTAATAAAGGTTAGAAACTAACGGCCGACATGAGAG	68	818	5	54.58	1076	1	0
N/A	GGGACACAATGGACGTAAATACTGCGAAAGAGTTGGACGGGTTTATAATACCGATAACGGCCGACATGAGAG	72	818	5	54.58	961	1	0
N/A	GGGACACAATGGACGTGGTATAAACGTGTGATTATGTGATACTCCCAATAGGCTATAACGGCCGACATGAGAG	73	818	5	54.58	974	1	0
N/A	GGGACACAATGGACGGTCGGAACAACTGTTTTAGTAAGATGTGACAGTGTTTACTTAACGGCCGACATGAGAG	73	818	5	54.58	838	1	0
N/A	GGGACACAATGGACGTCAACAACGATATAACGTACTAAATCTATAAGTATTGAGTTAACGGCCGACATGAGAG	73	818	5	54.58	994	1	0

N/A	GGGACACAATGGACGAACTTGAATCATTAAAAGTAGGTAGTAGCATTATAACTGCTAACGGCCGACATGAGAG	73	818	5	54.58	1041	1	0
N/A	GGGACACAATGGACGTTATAACTCTACATTTATTTCTTTACTTTTGGTGAGGGATAACGGCCGACATGAGAG	72	818	5	54.58	815	1	0
N/A	GGGACACAATGGACGCTATAAGCTGTTTAAATAATGAGAATTCGTTTAGAATGGCTAACGGCCGACATGAGAG	73	818	5	54.58	1032	1	0
N/A	GGGACACAATGGACGGTTTAGGTCAACCTACAACTTATGTAAAGCGGCTAGCATATAACGGCCGACATGAGAG	73	818	5	54.58	871	1	0
N/A	GGGACACAATGGACGTAATACTAACATGGGCGCAAATAATTAATATGCCGGTTAATAACGGCCGACATGAGAG	73	818	5	54.58	1048	1	0
N/A	GGGACACAATGGACGCAAAAGGGAGTACTTATAGCATTAGGAGCAAAGTATACCATAACGGCCGACATGAGAG	73	818	5	54.58	829	1	0
N/A	GGGACACAATGGACGTAGAGTACAATAGGGAATTAAATACTATTTCAACCATGTATAACGGCCGACATGAGAG	73	818	5	54.58	1030	1	0
N/A	GGGACACAATGGACGTAAACGGTTCGGAGAAACTTATTAAAAGGGTATCGAAAGACTAACGGCCGACATGAGAG	74	818	5	54.58	847	1	0
N/A	GGGACACAATGGACGTGTAAAGGATCGTTAATCGATTAATAACATTTATGATGAATAACGGCCGACATGAGAG	73	818	5	54.58	1069	1	0
N/A	GGGACACAATGGACGGCGAACTTATAGTGAAAAAATTATTAGAAATACCACGATAACGGCCGACATGAGAG	71	818	5	54.58	993	1	0
N/A	GGGACACAATGGACGATTAGATTAGTCTGATGTCGAGAGATTTGACATACGATACTAACGGCCGACATGAGAG	73	818	5	54.58	941	1	0
N/A	GGGACACAATGGACGCCTTTTACCGATGAAGCCTAAAAGCCAAACCGGTTATATAACGGCCGACATGAGAG	71	818	5	54.58	802	1	0
N/A	GGGACACAATGGACGATAGTAGAGTAACCGGAATGTCACTACAGACACATTCTACTAACGGCCGACATGAGAG	73	818	5	54.58	818	1	0
N/A	GGGACACAATGGACGCAACAAAACGGTTTATCGGAAAAGACAAATAGACGGTTATAACGGCCGACATGAGAG	72	818	5	54.58	1107	1	0
N/A	GGGACACAATGGACGCATTATTTGTAAATTGATGAAATACACCAGGAGTGCCGGTTAACGGCCGACATGAGAG	73	818	5	54.58	956	1	0
N/A	GGGACACAATGGACGCATCTACTGAATTCAGCAACATATTAACCTGTTTGCTCAGTAACGGCCGACATGAGAG	73	818	5	54.58	1114	1	0
N/A	GGGACACAATGGACGTCGCGCCAATGAATAATATTTGGTTAACGGCCGACATGAGAG	57	818	5	54.58	1081	1	0
N/A	GGGACACAATGGACGGGTAATTGCGTTTACCTTAGTAGAATAAGAAACTACGTGGTAACGGCCGACATGAGAG	73	818	5	54.58	1145	1	0
N/A	GGGACACAATGGACGGTGAAGAACTTATAGATATCATTAACGGTTTCGATATGGATAACGGCCGACATGAGAG	73	818	5	54.58	1126	1	0
N/A	GGGACACAATGGACGATATTTCGGTTAGTAGTGAACAGCAAATAACGGCCGACATGAGAG	60	818	5	54.58	1152	1	0
N/A	GGGACACAATGGACGGATAAGAATTAACGGCCGACATGAGAG	42	818	5	54.58	279	5	5
N/A	GGGACACAATGGACGCTAAAAAGATTCTTCTAATCAACTTTGATTCTGAAGTCGATAACGGCCGACATGAGAG	73	818	5	54.58	1057	1	0
N/A	GGGGGCTCGAGAAAAAATACAAAGCGTA	28	818	5	54.58	1096	1	0
N/A	GGGACACAATGGACGCAGTAAGTAAAATTAGAGCAGACATTTATGTCGTTGGCTTAACGGCCGACATGAGAG	72	818	5	54.58	1106	1	0
N/A	GGGACACAATGGACGAAAGCTTATATTGCATTAGTTTGGCGTGACAAAGTTGGCTTAACGGCCGACATGAGAG	73	818	5	54.58	836	1	0
N/A	GGGACACAATGGACGGAGAAGTTAGTGATTTTAAATAATAACGGCCGACATGAGAG	56	818	5	54.58	1058	1	0
N/A	GGGACACAATGGACGATGAACCATATTCAACCCAACATGAAGGAAGTTTTACACATAACGGCCGACATGAGAG	73	818	5	54.58	945	1	0
N/A	GGGACACAATGGACGGAAAGGCCTATTTTATTTCTACGATAACGGCCGACATGAGAG	57	818	5	54.58	972	1	0
N/A	GGGACACAATGGACGGTGTAGGCTAAATCTTTAACTATTTGGATTATGTTGTAGATAACGGCCGACATGAGAG	73	818	5	54.58	1026	1	0
N/A	GGGACACAATGGACGAATGAAGGCCAAAGATAACATATATAAGATGATGGAAGCTTAACGGCCGACATGAGAG	73	818	5	54.58	1142	1	0
N/A	GGGACACAATGGACGGAATAGTGACATTTCAATGAGCGAAGAGCTAATGAGGGTTTAACGGCCGACATGAGAG	73	818	5	54.58	1099	1	0
N/A	GGGACACAATGGACGTTGACTAATTGGCGACTTGTAAGACTTCGTGCAGTTGAGACTTTAACGGCCGACATGAGA	76	818	5	54.58	991	1	0
N/A	GGGACACAATGGACGTGGAGGCATCATATAACTAATTTATTTGACATAGTGCGAATAACGGCCGACATGAGAG	73	818	5	54.58	969	1	0
N/A	GGGACACAATGGACGTACAATATGACCTGACTATCGAACGGACGTATAAGACGTTTAACGGCCGACATGAGAG	73	818	5	54.58	1133	1	0
N/A	GGGACACAATGGACGGAAAGATTACAGGTACAAGACAATAGAATGGTTTACTTGTAACGGCCGACATGAGAG	72	818	5	54.58	875	1	0
N/A	GGGACACAATGGACGTTTGAATTAAATCAGATAACAACACAAAGTTGTTTGGTATAACGGCCGACATGAGAG	72	818	5	54.58	800	1	0
N/A	GGACACAATGGACGTATATTTCATTATCAACCCCAATGTGATGGCCAAACTTGGTAACGGCCGACATGAGAG	72	818	5	54.58	1029	1	0
N/A	GGGACACAATGGACGGTAAGATTACCATAGACATACTTAAACGAGAGTCTGCGTAACGGCCGACATGAGAG	71	818	5	54.58	1148	1	0
N/A	GGGACACAATGGACGGTATAATATAATTCATTGACCAACATTTTTGGTAAGAGCATAACGGCCGACATGAGAG	73	818	5	54.58	938	1	0
N/A	GGGACACAATGGACGCAATAAAAACGTCATGATCATTGAAGGGCTGTTGCAATCGTAACGGCCGACATGAGAG	73	818	5	54.58	1093	1	0
N/A	GGGACACAATGGACGCAATATCGACTGAATGACATGGTACAAAATCTTTTGGTGATAACGGCCGACATGAGAG	73	818	5	54.58	1134	1	0
N/A	GGGACACAATGGACGAGTCTTTACAATCGTTAATCTGCAAAGTATTAAAACGACGATAACGGCCGACATGAGAG	74	818	5	54.58	828	1	0
N/A	GGGACACAATGGACGACTTATTACAGATGCAATTATGAACTAGAGTAACGGCCGACATGAGAG	63	818	5	54.58	885	1	0
N/A	GGGACACAATGGACGTACTAACATGAATTATGATACAACATAGATGGATGGCTGGTAACGGCCGACATGAGAG	73	818	5	54.58	1110	1	0
N/A	GGGACACAATGGACGTTCACATAACGGCCGACATGAGAG	39	818	5	54.58	948	1	0
N/A	GGGACACAATGGACGCTAGAAAATGACTACAGTAATTAACCATACTAATAGTACATAACGGCCGACATGAGAG	73	818	5	54.58	896	1	0
N/A	GGGACACAATGGACGGGTCGGAAGGTTTTCTGTTAATGATGAATTCAGTGAGCTGTAACGGCCGACATGAGAG	73	818	5	54.58	1061	1	0
N/A	GGGACACAATGGACGACCCTTAATTTTATAATACCGGATATAGGAATGTTTGGTCTAACGGCCGACATGAGAG	73	818	5	54.58	907	1	0
N/A	GGGACACAATGGACGGTTGAACGAATAATTAAGGTAATCGCATGATCACTAAGAGTAACGGCCGACATGAGAG	73	818	5	54.58	986	1	0
N/A	GGGACACAATGGACGTTTGAAGGTTATAAAAGTAGAGATACATGAAATGAATGGATAACGGCCGACATGAGAG	73	818	5	54.58	950	1	0
N/A	GGGACACAATGGACGACCGCGCAAAACACAATGGTTATGAGGAAAACTTTAACTACTAACGGCCGACATGAGAG	74	818	5	54.58	1071	1	0
N/A	GGGACACAATGGACGAAGTAGGCTAGAAGTCATAACACAAAAAGTGCTTATATTATAACGGCCGACATGAGAG	73	818	5	54.58	962	1	0
N/A	GGGACACAATGGACGATCCTGTCGAGAGATACAAGCATAGAGAATATGACTGATGTAACGGCCGACATGAGAG	73	818	5	54.58	1056	1	0
N/A	GGGACACAATGGACGCTATGGTTAGAGTAAGTAATATATTCAAATTTATTTGTACTAACGGCCGACATGAGAG	73	818	5	54.58	930	1	0
N/A	GGGACACAATGGACGGTATTATAGTGACGAAGTACATGAAATAGAGTAATTCGGATAACGGCCGACATGAGAG	73	818	5	54.58	1150	1	0
N/A	GGGACACAATGGACGCTTGTAATTAAAGTTAAACAGTGTAGCCATGGGCCAGCAATAACGGCCGACATGAGAG	73	818	5	54.58	804	1	0
N/A	GGGACACAATGGACGCAAGGTAACAATTGGGTAACCATAGATTGGCTGTATAAGGTAACGGCCGACATGAGAG	73	818	5	54.58	878	1	0
N/A	GGGACACAATGGACGATTAGAACAGCAATAGCCCACTCGACCGAAGTTTAACATATAACGGCCGACATGAGAG	73	818	5	54.58	833	1	0
N/A	GGGACACAATGGACGAAGTAACACTCGACGGCATTAGTCAACTTCTATTAAGAGGTAACGGCCGACATGAGAG	73	818	5	54.58	1128	1	0
N/A	GGGACGCAATGGACGAAGATTTACATGATATTCGAAGATTAGAACCTCATTGGCTTAACGGCCGACATGAGAG	73	818	5	54.58	105	2	1

N/A	GGGACACAATGGACGTTTCAACATTTGCTTATAACGGCCGACATGAGAG	49	818	5	54.58	1117	1	0							
N/A	GGGACACAATGGACGCCATCTGGAACAATCGAGTAAAACAACAACCAAATTGTAATAACGGCCGACATGAGAG	73	818	5	54.58	1135	1	0							
N/A	GGGACACAATGGACGCTGAATGGAATATTAATTGGAACATCACAATGTTTTACCGTAACGGCCGACATGAGAG	73	818	5	54.58	1013	1	0							
N/A	GGGACACAATGGACGTCGGGACATCTCAAGCATGAATATTGCATACTTTAATTTGTAACGGCCGACATGAGAG	73	818	5	54.58	998	1	0							
N/A	GGGACACAATGGACGTATATAAGCTATTATAGTACAAATCAAATGTCATATAGGCTAACGGCCGACATGAGAG	73	818	5	54.58	859	1	0							
N/A	GGGACACAATGGACGTAAATTATAAGTATGTGCCCTATTGGGGTCGGTTATAGACTAACGGCCGACATGAGAG	73	818	5	54.58	1136	1	0							
N/A	GGGACACAATGGACGAGCCTTGATGTAAGTGAAGTTTATAATGACTTCTAGGACATAACGGCCGACATGAGAG	73	818	5	54.58	924	1	0							
N/A	GGGACACAATGGACGATTAGGCCGCGTGTAGCTTATTATTTTAAGCTAGGCTCATAACGGCCGACATGAGAG	72	818	5	54.58	927	1	0							
N/A	GGGACACAATGGACGCTGTATGGATTTTATGGGATTTATATATAACCAAAACCGGTAACGGCCGACATGAGAG	73	818	5	54.58	1085	1	0							
N/A	GGGACACAATGGACGAACGTGAACCTTGACAATATTTGAATAGAACTCGAGGGGTAACGGCCGACATGAGAG	72	818	5	54.58	943	1	0							
N/A	GGGACACAATGGACGGTTCTGAATATGTGTAATGTTGAATGATCGCAGAAGCACGTAACGGCCGACATGAGAG	73	818	5	54.58	937	1	0							
N/A	GGGACACAATGGACGCCTAATGATTTTTATCTAACGGCCGACATGAGAG	49	818	5	54.58	163	2	6							
N/A	GGGACACAATGGACGCCTCGCTAATCATACAATACGAAATTACATTCGTAGATAGTAACGGCCGATATGAGAG	73	818	5	54.58	876	1	0							
N/A	GGGACACAATGGACGAAATATCATTAATATAAATAACTCTAGTGGCTTAGGCGGATAACGGCCGACATGAGAG	73	818	5	54.58	891	1	0							
N/A	GGGACACAATGGACGCTGACTATTAGCAAAAGAGTATCTTGACATACGAGACGCTTAACGGCCGACATGAGAG	73	818	5	54.58	873	1	0							
N/A	GGGACACAATGGACGTAAGAACTAATTTGAGTGAACTTTCATTAAGCAGGCGTTTTAACGGCCGACATGAGAG	73	818	5	54.58	903	1	0							
N/A	GGGACACAATGGACGTAATAACTAACTTTGAGGTATGTAAAATACTCAAGTGTAATAACGGCCGACATGAGAG	73	818	5	54.58	1070	1	0							
N/A	GGGACACAATGGACGCAGAAAAAAAAGATTGGTAAATTATCTTAACGGCCGACATGAGAG	60	818	5	54.58	978	1	0							
N/A	GGGACACAATGGACGTAATCTCAAGCCGGGCAGAAAATTTAATCTTTTGCGTAACGGCCGACATGAGAG	69	818	5	54.58	831	1	0							
N/A	GGGACACAATGGACGAACATGTGGGGCTGAAGTAATATTTTGCTTATGTTGCAAATAACGGCCGACATGAGAG	73	818	5	54.58	1094	1	0							
N/A	GGGACACAATGGACGCCGGATACAGACTAAAATCTCTTAACATTTCTGGTGTGACTAACGGCCGACATGAGAG	73	818	5	54.58	1005	1	0							
N/A	GGGACACAATGGACGTTAAGCAAGAATTTGCAGTGTTATATAACGGCCGACATGAGAG	58	818	5	54.58	1022	1	0							
N/A	GGGACACAATGGACGAAATTATCGCCGGAACAGAATTACTTGGCTTTTATTCGTTTAACGGCCGACATGAGAG	73	818	5	54.58	807	1	0							
N/A	GGGACACAATGGACGATACAAAATACGTAATGGAACCAGGTGATAGATAGGCTATTAACGGCCGACATGAGAG	73	818	5	54.58	855	1	0							
N/A	GGGACACAATGGACGACATGAATGGCTGTAATAATTATGAGCATAACTTTTTATGTAACGGCCGACATGAGAG	73	818	5	54.58	975	1	0							
N/A	GGGACACAATGGACGACAATGAAACCATTTAAATCAGGCTGTTCTAGCAAATGTATAACGGCCGACATGAGAG	73	818	5	54.58	849	1	0							
N/A	GGGACACAATGGACGGAAATGCGTATTATTAAGATGGCAAGTACCTATTAAGATAACGGCCGACATGAGAG	71	818	5	54.58	905	1	0							
N/A	GGGACACAATGGACGCTGCTAGACTCAATATGGACATCTAAACATATAGTGGAGTTAACGGCCGACATGAGAG	73	818	5	54.58	1147	1	0							
N/A	GGGACACAATGGACGTATAGAAAACATATCATAAACAAAATTAATTTGCTGGCTATAACGGCCGACATGAGAG	73	818	5	54.58	799	1	0							
N/A	GGGACACAATGGACGTATAAATATTAACAAGCAGTATGCTGATAGCATAATGTGTTAACGGCCGACATGAGAG	73	818	5	54.58	894	1	0							
N/A	GGGACACAATGGACGGTGACGTAACAGGAGACATGATTTTAATGTAATTCTAACGTAACGGCCGACATGAGAG	73	818	5	54.58	1082	1	0							
N/A	GGGACACAATGGACGAAGGTTGATAGTGAGTAGTGAAGATAAAATAATTCAACGCTAACGGCCGACATGAGAG	73	818	5	54.58	1039	1	0							
N/A	GGGACACAATGGACGAACTATGGAGGGGGATTAGTGATATTCAAAAATAACTGATAACGGCCGACATGAGAG	72	818	5	54.58	854	1	0							
N/A	GGGACACAATGGACGAATAGGGAGGCATGGGATAATAAAATTTGTATACATATTATAACGGCCGACATGAGAG	73	818	5	54.58	806	1	0							
N/A	GGGACACAATGGACGATAAGTAGATTATTTTCTCTAGTCACTAATTCGAGGCGTAACGGCCGACATGAGAG	71	818	5	54.58	1083	1	0							
N/A	GGGACACAATGGACGGTAAGTTATTTTTTAAGTAAAAGGCTGGAAATGCCATAAGCTAACGGCCGACATGAGAG	74	818	5	54.58	1149	1	0							
N/A	GGGACACAATGGACGAACCAATATTAGTTACAGAAATTAGAGTACCTGCGGTCGGTAACGGCCGACATGAGAG	73	818	5	54.58	1034	1	0							
N/A	GGGACACAATGGACGTATTTGATATTTAAAGGTGTTGAACCACCGAATGTAGGCGTAACGGCCGACATGAGAG	73	818	5	54.58	1098	1	0							
N/A	GGGACACAATGGACGAAACTTCAATGCCCAAAAAACGTACTTTAGAGCTTTTGTAGGTAACGGCCGACATGAGAG	75	818	5	54.58	890	1	0							
N/A	GGGACACAATGGACGTAGCCATACTACAATGCATAATTATAGAAAAATGTAGTGCTAACGGCCGACATGAGAG	73	818	5	54.58	1046	1	0							
N/A	GGGACACAATGGACGACACTAAATGTTACTAATATGTTCCTTGCAAATGGGGCTATAACGGCCGACATGAGAG	73	818	5	54.58	904	1	0							
N/A	GGGACACAATGGACGTAGATCTATACTTTAAATGTAACCTGGAAATGTTAGCGCCTAACGGCCGACATGAGAG	73	818	5	54.58	872	1	0							
N/A	GGGACACAATGGACGCTTAAGTGCATGCTATAAGAAGCAAGTACGTTTAATAGTCTAACGGCCGACATGAGAG	73	818	5	54.58	897	1	0							
N/A	GAGACACAATGGACGGAGAAGAATTTGGTGGAAGTAATTATAACCCACTGATTATTAACGGCCGACATGAGAG	73	818	5	54.58	892	1	0							
N/A	GGGACACAATGGACGCTATTTAGTTAGACATTGATTTCACTACGCATTTTGGTCTTAACGGCCGACATGAGAG	73	818	5	54.58	1036	1	0							
N/A	GGGACACAATGGACGTTGCCTATTAGATCAATATTACTAACGGCCGACATGAGAG	55	818	5	54.58	823	1	0							
N/A	GGGACACAATGGACGGCATACAT	23	818	5	54.58	707	2	4							
N/A	GGGACACAATGGACGTAGGTCGGTATATAGATACAATTTTTAAGGTAACTTGAATATAACGGCCGACATGAGAG	74	818	5	54.58	916	1	0							
N/A	GGGACACAATGGACGTTCAACTTAATAACATGCCATTGCAACAACCATCAGGCTATAACGGCCGACATGAGAG	73	818	5	54.58	862	1	0							
N/A	GGGACACAATGGACGTGCATAACGGCCGACATGAGAG	37	818	5	54.58	279	4	6							
N/A	GGGACACAATGGACGGTTTTGTTACTTTTAGTGCGATGAGCGCAATAAATGCACCTAACGGCCGACATGAGAG	73	818	5	54.58	817	1	0							
N/A	GGGACACAATGGACGAGGCGGGTTCTAATAGATAATTTCACAGGTCTGAAGGACGTAACGGCCGACATGAGAG	73	818	5	54.58	874	1	0							
N/A	GGGACACAATGGACGATTTGAATCACTTTAAGGTTCGACAAACTTAGTGGCGGGCTAACGGCCGACATGAGAG	73	818	5	54.58	1151	1	0							
N/A	GGGACACAATGGACGCAGGTATGAGATTTAACTTTATTTTAACAGATAAATGTTCTAACGGCCGACATGAGAG	73	818	5	54.58	867	1	0							
N/A	GGGACACAATGGACGGCGAGAAGAGCTATGGCATATCAGGATTAGATAAACAAGGTAACGGCCGACATGAGAG	73	818	5	54.58	1129	1	0							
N/A	GGGACACAATGAACGTATAGTAACATAAGTGAGGCCGTATGAAAATAGTAATGGGTAACGGCCGACATGAGAG	73	818	5	54.58	832	1	0							
N/A	CTCAACGACCATTACGATCAAGCCTAGCTAAACTTGCCAGACGACTCGCCCG	52	818	5	54.58	973	1	0							
N/A	GGGACACAATGGACGTT	17	818	5	54.58	3	16	3	1398	5	34.25	1	15	3	0.6275
N/A	GGGACACAATGGACGCAATTGGATTAACTAAGCGGTTTATTGTAATGGCCGCGCAATAACGGCCGACATGAGAG	74							2	46	315.06	2	1	0	
N/A	GGGACACAATGGACGTATGGATATTCGGAGGACTAGACAAGAGAACTTGAATACTAACGGCCGACATGAGAG	72							4	35	239.72	4	1	0	

N/A	GGGACACAATGGACGGTATAACGGCCGACATGAGAG	36	5	32	219.17	5	1	0
N/A	GGGACACAATGGACGGTACAGAGAGATTGCACTGATTCAGAGTTGTAAGATGGCTTAACGGCCGACATGAGAG	73	7	28	191.78	8	1	0
N/A	GGGACACAATGGACGGATCGAGTACTGAAAATATAAGTAAATAATGTTGGACCGGTAACGGCCGACATGAGAG	73	7	28	191.78	6	1	0
N/A	GGGACACAATGGACGTGTATTATGATTAATAGAAGGCGTGTTTCATTATAAGCCATAACGGCCGACATGAGAG	73	7	28	191.78	7	1	0
N/A	GGGACACAATGGACGAGTAATCTAACAACTAATTCAAACATCACATGAGCAAGGCGTAACGGCCGACATGAGAG	74	11	27	184.93	9	1	0
N/A	GGGACACAATGGACGCCAAAGTTAAAATACAATGTATCCTACATCACGGTTGTTGTAACGGCCGACATGAGAG	73	12	26	178.08	10	1	0
N/A	GGGACACAATGGACGATCAGTCAGGCACTGATGCATAAAGTGTATTTGTTTTATGTAACGGCCGACATGAGAG	73	13	25	171.23	11	1	0
N/A	GGGACACAATGGACGCATTAGGCTGTATAGAAATTGCAAAACCGGCTGCTGATAATAACGGCCGACATGAGAG	73	16	24	164.38	15	1	0
N/A	GGGACACAATGGACGTGCTGAAGCGAAACTTCAAGGACTTAAAATTTTGGTTGGGCTAACGGCCGACATGAGAG	74	16	24	164.38	13	1	0
N/A	GGGACACAATGGACGTTAACTATCGAATATATTAAGGGGAAAGACTTAGACGGGATAACGGCCGACATGAGAG	73	16	24	164.38	12	1	0
N/A	GGGACACAATGGACGGTCAGATAGGCCTTATAGAGAATATTACGAATATCTATGGTAACGGCCGACATGAGAG	73	20	23	157.53	18	1	0
N/A	GGGACACAATGGACGCCGCAATATTTAATGATGCAGAGTTTATAACAGCATACTCTAACGGCCGACATGAGAG	73	20	23	157.53	16	1	0
N/A	GGGACACAATGGACGAAATAACAAGCTATTCGAGATATACGGTATTATAGCCTTATAACGGCCGACATGAGAG	73	20	23	157.53	17	1	0
N/A	GGGACACAATGGACGTATTTATAACGGCCGACATGAGAG	39	20	23	157.53	5	2	4
N/A	GGGACACAATGGACGCCGAATCTTTAGAACTATAACCTACACAAGAGTATTAGGGTAACGGCCGACATGAGAG	73	24	22	150.68	20	1	0
N/A	GGGACACAATGGACGATCAAATTTGTTGCTGTTGAACTACATCAAGTGGTTGGCTTAACGGCCGACATGAGAG	73	24	22	150.68	23	1	0
N/A	GGGACACAATGGACGTTAAGTAACTGTGGATCAAATGTTGGCTTTTAGATACTGTAACGGCCGACATGAGAG	72	24	22	150.68	21	1	0
N/A	GGGACACAATGGACGTAAGTTATTTTAATTAGCTAACGGCCGACATGAGAG	51	24	22	150.68	19	1	0
N/A	GGGACACAATGGACGAAGAGGCGTAAGTACTATGTGGAGCAGGTAATGTATGGCGTAACGGCCGACATGAGAG	73	24	22	150.68	22	1	0
N/A	GGGACACAATGGACGATCCGATGGATTTTAGTAATATTCGCTGAATGTGACATCCTAACGGCCGACATGAGAG	73	30	21	143.83	24	1	0
N/A	GGGACACAATGGACGATGCGTGATAGCATACTTCTAGCATTTAGAGGACTGCAATTAACGGCAGACATGAGAG	73	30	21	143.83	28	1	0
N/A	GGGACACAATGGACGTAAACGAATAACATTCGTAATGCTGTTTCAATGACAGGCTTAACGGCCGACATGAGAG	73	30	21	143.83	26	1	0
N/A	GGGACACAATGGACGGTAAAATATACTGAAAACAGTGGAGACGATGTTGTTGGCATAACGGCCGACATGAGAG	73	30	21	143.83	25	1	0
N/A	GGGACACAATGGACGTATCAAGAGAATTTTTTAAGTAACGGCCGACATGAGAG	53	30	21	143.83	27	1	0
N/A	GGGACACAATGGACGGAAGAAAGCCAAAATAGTGGAGGGCGGTTTAGTTTGTATCTAACGGCCGACATGAGAG	73	35	20	136.98	32	1	0
N/A	GGGACACAATGGACGAGACATTTTCATCTTATTGGACTCTATTGAGCCAATGTATAACGGCCGACATGAGAG	72	35	20	136.98	34	1	0
N/A	GGGACACAATGGACGGTATTTGAAGGTTGAGATAGTATGATATATACTAACGAACTAACGGCCGACATGAGAG	73	35	20	136.98	29	1	0
N/A	GGGACACAATGGACGGATTCACCGTATAGATGATAACGGCCGACATGAGAG	51	35	20	136.98	30	1	0
N/A	GGGACACAATGGACGTGCACTTTGATAATAACGGCCGACATGAGAG	46	35	20	136.98	33	1	0
N/A	GGGACACAATGGACGTGAACTGTCACTATAACAACGAGAGTAACAATGGTGAAGCTAACGGCCGACATGAGAG	73	35	20	136.98	35	1	0
N/A	GGGACACAATGGACGATAGTGAGTTTAATATCGACAGTATATACAAATGGGGGCTTAACGGCCGACATGAGAG	73	35	20	136.98	31	1	0
N/A	GGGACACAATGGACGTTGGATAACATCATTTTTCTATCGAGATGATGAACTAAGCTAACGGCCGACATGAGAG	73	42	19	130.13	49	1	0
N/A	GGGACACAATGGACGATGTCGGGGCAAGTGGTTTATAGTAATATATCGAACGAGCTAACGGCCGACATGAGAG	73	42	19	130.13	43	1	0
N/A	GGGACACAATGGACGTTAGTGAGATCAATATTAACTACTGAGTGAATCGGGTCGGTAACGGCCGACATGAGAG	73	42	19	130.13	46	1	0
N/A	GGGACACAATGGACGAAAAAGGACTTTAGAGAGAATATATTTTTCTAGGATCCGGTAACGGCCGACATGAGAG	73	42	19	130.13	36	1	0
N/A	GGGACACAATGGACGAACGGTGGGCTCGTTGAGATTTAGCGTATTTTAGCACTATATAACGGCCGACATGAGAG	74	42	19	130.13	50	1	0
N/A	GGGACACAATGGACGCTTTAGTCAAAGTCATAGACACCTTATTTATGGCTCTAATTAACGGCCGACATGAGAG	73	42	19	130.13	44	1	0
N/A	GGGACACAATGGACGGTCAGAAGTGGATTCTTCAAAGCGAATACATGAGTTTGCTTAACGGCCGACATGAGAG	73	42	19	130.13	45	1	0
N/A	GGGACACAATGGACGGATGTTTTTATGCACTATGTCAATCGAAATGATGATAAGGATAACGGCCGACATGAGAG	74	42	19	130.13	40	1	0
N/A	GGGACACAATGGACGGAACGTGTTTTTATAACGGCCGACATGAGAG	46	42	19	130.13	38	1	0
N/A	GGGACACAATGGACGAGACCGCGAAATATTAGTAGGTTAACATCTATACGATGAGTAACGGCCGACATGAGAG	73	42	19	130.13	39	1	0
N/A	GGGACACAATGGACGGTAATGCAGGGCAAAAATAGAATTTTCAACGTATTAACGGCCGACATGAGAG	67	42	19	130.13	47	1	0
N/A	GGGACACAATGGACGTATGGTACATGTATCGGCATTTACGTTTGATAAACAGCAATAACGGCCGACATGAGAG	73	42	19	130.13	48	1	0
N/A	GGGACACAATGGACGCAAGTCGAATTGTAAAGTCGGTAGTTATTATTTGGCAATGTAACGGCCGACATGAGAG	73	42	19	130.13	41	1	0
N/A	GGGACACAATGGACGTTAACAAACATCAAATGTTACCTGAACTCGTCAGGCTATAACGGCCGACATGAGAG	71	42	19	130.13	42	1	0
N/A	GGGACACAATGGACGGTTATAAATGGCAAAGACTATAACGGCCGACATGAGAG	53	42	19	130.13	37	1	0
N/A	GGGACACAATGGACGGAACGGGCGTACGATTTAAATACGTTATTATATTTTAGCGTAACGGCCGACATGAGAG	73	58	18	123.28	58	1	0
N/A	GGGACACAATGGACGTAAATATAGAATCTCGTGACTGCATGGTAAAGAGTAATATTAACGGCCGACATGAGAG	73	58	18	123.28	59	1	0
N/A	GGGACACAATGGACGGACGTAAGCTAGCCATGTTATAATTTTGTAACGCAATAGGTAACGGCCGACATGAGAG	73	58	18	123.28	54	1	0
N/A	GGGACACAATGGACGGACCTTATTAATTTTATAATCGTAGTGGAAACACTGAGCGTAACGGCCGACATGAGAG	73	58	18	123.28	51	1	0
N/A	GGGACACAATGGACGGGAACTCAAGAAATGGCGAACTTAACGGCCGACATGAGAG	55	58	18	123.28	60	1	0
N/A	GGGACACAATGGACGACTTTATATTACGGGGAGAACCTAACGGTACAAACCGAGATAACGGCCGACATGAGAG	73	58	18	123.28	61	1	0
N/A	GGGACACAATGGACGGTTCATATCAGCATAGGCTTCTATTTTTGTAGCTCACCGCTAACGGCCGACATGAGAG	73	58	18	123.28	53	1	0
N/A	GGGACACAATGGACGTGCACAATTACGGAGTTAGAATTTATAAGCTTGCTGATTGTAACGGCCGACATGAGAG	73	58	18	123.28	55	1	0
N/A	GGGACACAATGGACGTTAGGTTGACACGTTGTTTTTACACACTCAAGATTCTGCGTAACGGCCGACATGAGAG	73	58	18	123.28	57	1	0
N/A	GGGACACAATGGACGATAAGCATACTAATATTGTACTATTATGATCAAGTGGGCTTAACGGCCGACATGAGAG	73	58	18	123.28	52	1	0
N/A	GGGACACAATGGACGAATCCATATGGAACTGATGGTTGACTAAGTGCATTTTGCATAACGGCCGACATGAGAG	73	58	18	123.28	56	1	0
N/A	GGGACACAATGGACGTAAGAAATGTAATGAGTTAACCAACCTGATCGGTGGCGATTAACGGCCGACATGAGAG	73	69	17	116.44	70	1	0
N/A	GGGACACAATGGACGGATAGTGAAACATATTCAACGACAGTATTGAAATGGAGTATAACGGCCGACATGAGAG	73	69	17	116.44	73	1	0
N/A	GGGACACAATGGACGTTTGAACTTTAATGACAATGGACCGAAATATGTAGGCACATAACGGCCGACATGAGAG	73	69	17	116.44	64	1	0

N/A	GGGACACAATGGACGGACGTTTAGCTTGGATTGATTCGATAACGGCCGACATGAGAG	57	69	17	116.44	76	1	0
N/A	GGGACACAATGGACGTGAGTTTATATATATACATACAAGCTGTTAGAGGGAATGCTAACGGCCGACATGAGAG	73	69	17	116.44	65	1	0
N/A	GGGACACAATGGACGCGTACTGCTACGATTGATTATGATCAGAATGTATGCCTTCTAACGGCCGACATGAGAG	73	69	17	116.44	69	1	0
N/A	GGGACACAATGGACGCTTACACACTATATATTGCTCGGATTACAATTGCCAGTGATAACGGCCGACATGAGAG	73	69	17	116.44	68	1	0
N/A	GGGACACAATGGACGGGACGAAAGACGTAATGGGTATATAAAAACGCAGTGGTTCTTAACGGCCGACATGAGAG	74	69	17	116.44	72	1	0
N/A	GGGACACAATGGACGAACGGTAACAAAACTACCATCAATTCGTCTGTAGGCCAAATAACGGCCGACATGAGAG	73	69	17	116.44	66	1	0
N/A	GGGACACAATGGACGCTAGCAATAATTTGATCTAATAATCGAACGACTTTGCATCTAACGGCCGACATGAGAG	73	69	17	116.44	75	1	0
N/A	GGGACACAATGGACGTTGCCGTGAATATAAAGTAAGTAGATAACTAACGGCCGACATGAGAG	62	69	17	116.44	71	1	0
N/A	GGGACACAATGGACGTAGAGATGAATGCTTTTTTGATTAAGCTGAGACATAACGGCCGACATGAGAG	67	69	17	116.44	78	1	0
N/A	GGGACACAATGGACGCTAAAGTTTCAGCTACGCTATGTAAATGGGTAAGCATGCATAACGGCCGACATGAGAG	73	69	17	116.44	62	1	0
N/A	GGGACACAATGGACGATTTGAATTTACCAAGGACACAATCACAAAACGGTCAGTATAACGGCCGACATGAGAG	73	69	17	116.44	74	1	0
N/A	GGGACACAATGGACGGCACTTTCTTGATTATGCAGGTAACGGCCGACATGAGAG	54	69	17	116.44	77	1	0
N/A	GGGACACAATGGACGTATTCTTATATTAATATCCAAAGACCGGCATCAGTTGGGCTAACGGCCGACATGAGAG	73	69	17	116.44	63	1	0
N/A	GGGACACAATGGACGGTAAAAATAAGTACCACGTTTCAGAAATATGAGGGGTAACGGCCGACATGAGAG	69	69	17	116.44	67	1	0
N/A	GGGACACAATGGACGAATGTCGAGTGCTAGTTTGCTTAATAACGGTTTGTATAGCTAACGGCCGACATGAGAG	73	86	16	109.59	85	1	0
N/A	GGGACACAATGGACGTATTTCAAAATCTATGAGAAAGAATCAAGATGAAGCAATAACGGCCGACATGAGAG	71	86	16	109.59	87	1	0
N/A	GGGACACAATGGACGTGACCTCTAGTTTATGTGATATATTCGTCAACTAAGGAAGTAACGGCCGACATGAGAG	73	86	16	109.59	83	1	0
N/A	GGGACACAATGGACGAATAACATATATTTACGTACTATAACACCGAGTGGGGCCATAACGGCCGACATGAGAG	73	86	16	109.59	84	1	0
N/A	GGGACACAATGGACGTTGGTATTAAATTAGAAGCTATATAAGACACTTCAAAGCCTAACGGCCGACATGAGAG	73	86	16	109.59	89	1	0
N/A	GGGACACAATGGACGAGTCTTACAGTAAAGCCGAATACAAAATGAGGTTTAAGCGTAACGGCCGACATGAGAG	73	86	16	109.59	88	1	0
N/A	GGGACACAATGGACGATTGAATAGGCACGGTACGATTGTAGAATGATACATTAGTAACGGCCGACATGAGAG	72	86	16	109.59	90	1	0
N/A	GGGACACAATGGACGTGATTTTGGGAGGAACTTATATAGGATGAGAACCACAAACTAACGGCCGACATGAGAG	73	86	16	109.59	91	1	0
N/A	GGGACACAATGGACGCTGAGAGGAATGGACAAAGTTTTAATACGATGTGAAGGACTAACGGCCGACATGAGAG	73	86	16	109.59	86	1	0
N/A	GGGACACAATGGACGCTATTGTTTAAGAGAATGAAGGTGCTAACAGTATAGCTGCTAACGGCCGACATGAGAG	73	86	16	109.59	92	1	0
N/A	GGGACACAATGGACGGTGGTAAGACACTGAATAAAATAGTGCGGCTTCAGATTATTAACGGCCGACATGAGAG	73	86	16	109.59	93	1	0
N/A	GGGACACAATGGACGCTGTGAAATTTAAATATCGTGAATATAACGATGGTGAGTATAACGGCCGACATGAGAG	73	86	16	109.59	80	1	0
N/A	GGGACACAATGGACGAATTTAATAGACATAAGAAATGACTGGCCAAATAACTAGATAACGGCCGACATGAGAG	73	86	16	109.59	81	1	0
N/A	GGGACACAATGGACGTATTGACATATCAAGTTCATATTGACTCAGGACATGAGGCTAACGGCCGACATGAGAG	73	86	16	109.59	79	1	0
N/A	GGGACACAATTGACGGAGAATAATATTAATAGATGTGTAAGTACTACAGATCGCATAACGGCCGACATGAGAG	73	86	16	109.59	94	1	0
N/A	GGGACACAATGGACGATGCTACAATAAGCTAAAGTTATATGAACCTATCGGGGGCTAACGGCCGACATGAGAG	73	86	16	109.59	82	1	0
N/A	GGGACACAATGGACGGAAACCTAATGACCGAAGTTATTACAAAAGACTAAATGGCTAACGGCCGACATGAGAG	73	103	15	102.74	107	1	0
N/A	GGGACACAATGGACGGCATGTTGAACTAGTATCGGGAATCAATATCCTTTATGAGTAACGGCCGACATGAGAG	73	103	15	102.74	105	1	0
N/A	GGGACACAATGGACGATGGTAATTGATGTAGACATACTACTTGTATGGAGGTCGCTAACGGCCGACATGAGAG	73	103	15	102.74	115	1	0
N/A	GGGACACAATGGACGGTAAGTTTAATAAAGTAATCAAAGTGAAAACACTTATACTTAACGGCCGACATGAGAG	73	103	15	102.74	97	1	0
N/A	GGGACACAATGGACGCATGTATGATATTATATCGCAGCCTGTTATATCGCAAGAATAACGGCCGACATGAGAG	73	103	15	102.74	112	1	0
N/A	GGGACACAATGGACGTGACGGACTTAGATTACGAATTTATTACGAAGTAAGAACCTAACGGCCGACATGAGAG	73	103	15	102.74	108	1	0
N/A	GGGACACAATGGACGCACGTAGAAAAATGATACAAATAACGGCCGACATGAGAG	54	103	15	102.74	111	1	0
N/A	GGGACACAATGGACGTATAGTGTATTGATGATAAAGGTCGTTGACAGTTGAATGATAACGGCCGACATGAGAG	73	103	15	102.74	116	1	0
N/A	GGGACACAATGGACGATAATAGGATGTCAATGACGTGAAGGCCTGATTTATGATTTAACGGCCGACATGAGAG	73	103	15	102.74	102	1	0
N/A	GGGACACAATGGACGTAAGATTAAATGAACTGACGAAAAGGATACCTGGAAGGCATAACGGCCGACATGAGAG	73	103	15	102.74	109	1	0
N/A	GGGACACAATGGACGTAGAAGATTATACAAATTTTGATGACCTTAGGGCCACCATAACGGCCGACATGAGAG	72	103	15	102.74	99	1	0
N/A	GGGACACAATGGACGGTGACATGTTTTTGTTTCTCCTGGGGTCATTGGCGTGCTGGTAACGGCCGACATGAGAG	74	103	15	102.74	113	1	0
N/A	GGGACACAATGGACGTCGCTTACAAATAGATGCATATTATCATCGATATTCGAGTAACGGCCGACATGAGAG	72	103	15	102.74	104	1	0
N/A	GGGACACAATGGACGAAAATAACTGTTGCACTTTGTCGCACTACGCATTAGGCTGTAACGGCCGACATGAGAG	73	103	15	102.74	95	1	0
N/A	GGGACACAATGGACGGACAATTATATCGACATATATAAGATAGCATGTGCAGTGCTAACGGCCGACATGAGAG	73	103	15	102.74	103	1	0
N/A	GGGACACAATGGACGTAGTATGATGTACATGGTTTGGACCGTGAGAATGATGGCTTAACGGCCGACATGAGAG	73	103	15	102.74	101	1	0
N/A	GGGACACAATGGACGGCGCAATACATGAAAGGAATGATTAATTCCATGTTAAGGATAACGGCCGACATGAGAG	73	103	15	102.74	96	1	0
N/A	GGGACACAATGGACGTAAATCATAATGTAAGCTGTGTGCTTGTGAGGTGACAAGTAACGGCCGACATGAGAG	72	103	15	102.74	106	1	0
N/A	GGGACACAATGGACGCATAGATTTAATTTGATCGCTATATTGGTTACAGGGTCGGTAACGGCCGACATGAGAG	73	103	15	102.74	110	1	0
N/A	GGGACACAATGGACGCCGGGATGATATTCACAAATATTTAAAACCTCAAAGGGTTAACGGCCGACATGAGAG	72	103	15	102.74	100	1	0
N/A	GGGACACAATGGACGTTCCACCGGCCAAATAAGTATAGGAATTAACACGCTTAAATAACGGCCGACATGAGAG	73	103	15	102.74	114	1	0
N/A	GGGACACAATGGACGCAATGTTTATTTGAATAGCCAGAGTGATGGAACGGTCATATAACGGCCGACATGAGAG	73	125	14	95.89	119	1	0
N/A	GGGACACAATGGACGCTGTGGTATTCAGTCTGATATAAGGACGTATGTCAAGTACTAACGGCCGACATGAGAG	73	125	14	95.89	151	1	0
N/A	GGGACACAATGGACGACCTGAATTATAAATACACACTTATGGTATGTAAGACGATAACGGCCGACATGAGAG	72	125	14	95.89	139	1	0
N/A	GGGACACAATGGACGGACCACAACATGAAAAGCTATTTAAGAGAATGCTTACACATAACGGCCGACATGAGAG	73	125	14	95.89	132	1	0
N/A	GGGACACAATGGACGGAAAAGTTAACCTAAGAGACTGTGCTAACACAGGGTTTGCTAACGGCCGACATGAGAG	73	125	14	95.89	145	1	0
N/A	GGGACACAATGGACGTAATTATGTATGTATCTGTGAGGCACGAACTGCTAACAGTAACGGCCGACATGAGAG	72	125	14	95.89	147	1	0
N/A	GGGACACAATGGACGACAATTATAATCATGAACTATTGATCTCGAGTTCGAGGCTTAACGGCCGACATGAGAG	73	125	14	95.89	134	1	0
N/A	GGGACACAATGGACGTACAAATTCTTATCCAAAGTTATCTGATGTTTGGGCTGCGTAACGGCCGACATGAGAG	73	125	14	95.89	125	1	0
N/A	GGGACACAATGGACGCAATATCACGCTATAAGGTTAATTAAACTGTATGAGTAACGGCCGACATGAGAG	69	125	14	95.89	137	1	0

N/A	GGGACACAATGGACGTTACCTAGTTGTATTAGATTTAATTAACAGCTGGTTTCAGTAACGGCCGACATGAGAG	73	125	14	95.89	133	1	0
N/A	GGGACACAATGGACGCTGGAAATCATAGATAATTAAGACAAATATATGAAAAGAGTAACGGCCGACATGAGAG	73	125	14	95.89	148	1	0
N/A	GGGACACAATGGACGGTAAATACGAACACGAAGTAACAGTAGTTTGCTTACCACGTAACGGCCGACATGAGAG	73	125	14	95.89	122	1	0
N/A	GGGACACAATGGACGGTACGTGAAAGCAGTACAAAGCATATGACTATATTGTCAGTAACGGCCGACATGAGAG	73	125	14	95.89	136	1	0
N/A	GGGACACAATGGACGGATGACATTTTATGAAATTCGAGCTAGCATATAGGCCAGATAACGGCCGACATGAGAG	73	125	14	95.89	118	1	0
N/A	GGGACACAATGGACGGCTAACTACATCTGCACCTATGACTGTGTGCACAATTATATAACGGCCGACATGAGAG	73	125	14	95.89	152	1	0
N/A	GGGACACAATGGACGTATTGAAAAGGTTGACCGGTATCTATGAGGGTTTTACTAGTAACGGCCGACATGAGAG	73	125	14	95.89	138	1	0
N/A	GGGACACAATGGACGGATTACAAATAAATAAATCGTAACGGCCGACATGAGAG	53	125	14	95.89	131	1	0
N/A	GGGACACAATGGACGTTTTTTCACACATAGTAAATTGGATGACTGAGTGCTGGCCTAACGGCCGACATGAGAG	73	125	14	95.89	141	1	0
N/A	GGGACACAATGGACGGTGCAGTTTACAAGTTTTATCTTTTCGTAACGGCCGACATGAGAG	60	125	14	95.89	143	1	0
N/A	GGGACACAATGGACGCTGAACTTAAAGGGTAAGTAATGATTTAACTGACGAGCCCTAACGGCCGACATGAGAG	73	125	14	95.89	124	1	0
N/A	GGGACACAATGGACGTTTAATTTGAGCTTCCACATGAGGTTGATAGCGATAGGGCTAACGGCCGACATGAGAG	73	125	14	95.89	135	1	0
N/A	GGGACACAATGGACGTAGGAATACTGGAGGTCTTTAAACTGAAACCATCCAGGGATAACGGCCGACATGAGAG	73	125	14	95.89	130	1	0
N/A	GGGACACAATGGACGTTTAATACGCTAAATAATGAGGAATCAAAAGTGGCCGAGATAACGGCCGACATGAGAG	73	125	14	95.89	144	1	0
N/A	GGGACACAATGGACGAAACTTTGAACGTTAGTCGCAGTTAGCAATTTAGGCTAAGTAACGGCCGACATGAGAG	73	125	14	95.89	129	1	0
N/A	GGGACACAATGGACGGTTAATAAGTGGGATTATTATAAATGAGAATCTACCCAGTTAACGGCCGACATGAGAG	73	125	14	95.89	128	1	0
N/A	GGGACACAATGGACGCTAAACTATAAGGTTATTAAGTAGTATAGTACTCTGGCCTTAACGGCCGACATGAGAG	73	125	14	95.89	117	1	0
N/A	GGGACACAATGGACGAGGTACTACATGGACGAAGATTTAAAAGATCATTTAGAACTAACGGCCGACATGAGAG	73	125	14	95.89	149	1	0
N/A	GGGACACAATGGACGTTAAACATTTTGTCATTTACGTAACGGCCGACATGAGAG	54	125	14	95.89	146	1	0
N/A	GGGACACAATGGACGGCCTAATTTACAGTGACCAAATATTAAGGATTCACACGGGTAACGGCCGACATGAGAG	73	125	14	95.89	120	1	0
N/A	GGGACACAATGGACGTTTGTTAGAGCCGCATAGTATTTACAGTATCTTTGGCTATTAACGGCCGACATGAGAG	73	125	14	95.89	121	1	0
N/A	GGGACACAATGGACGAATAGTATACATATAGAAAATAGTCACCTCGGTCGTGGCTTAACGGCCGACATGAGAG	73	125	14	95.89	127	1	0
N/A	GGGACACAATGGACGCAAATACGCAGAGCCAATCATCAGGTTTGTATAGTAACGGCCGACATGAGAG	67	125	14	95.89	123	1	0
N/A	GGGACACAATGGACGCCATAGTTTTATAAGAATGTAAGCTAACGGCCGACATGAGAG	57	125	14	95.89	140	1	0
N/A	GGGACACAATGGACGCGCACGGATTTAAGGATACATATGAGAGATCAAAAAGTACTAACGGCCGACATGAGAG	73	125	14	95.89	126	1	0
N/A	GGGACACAATGGACGTATACTACACATTAATATCTATTGCTTGACCTATAACGGTTAACGGCCGACATGAGAG	73	125	14	95.89	142	1	0
N/A	GGGACACAATGGACGAATCAAAAGCTAACACAATTTGAATAACGGCCGACATGAGAG	57	125	14	95.89	150	1	0
N/A	GGGACACAATGGACGTTATAAGAACTACAATTAAACGCTACTGGTTACCGACGGCTAACGGCCGACATGAGAG	73	161	13	89.04	159	1	0
N/A	GGGACACAATGGACGATATGTTTTTCAATATGCGTAGTGAACCTGATGTAGGGCTTAACGGCCGACATGAGAG	73	161	13	89.04	168	1	0
N/A	GGGACACAATGGACGACCAAAGAGTATACAAGCATAGATCTGCGTTGTAAAGGCTAACGGCCGACATGAGAG	72	161	13	89.04	166	1	0
N/A	GGGACACAATGGACGTTACCGATTAGAATAGCATGGAGCTGGTATAGTGAATACTAACGGCCGACATGAGAG	72	161	13	89.04	163	1	0
N/A	GGGACACAATGGACGGTACATAATACTAAGAGAAAGTACAAAATTTCAAAGTAGGTAACGGCCGACATGAGAG	73	161	13	89.04	167	1	0
N/A	GGGACACAATGGACGGATACTGTATAAGGAGGTTTGTTTGTCCAATGGCATATAATAACGGCCGACATGAGAG	73	161	13	89.04	162	1	0
N/A	GGGACACAATGGACGAAGGTTACAACGACAAAATCTTGGCGGGAATGTAAGTTACTAACGGCCGACATGAGAG	73	161	13	89.04	174	1	0
N/A	GGGACACAATGGACGGACAAGTCTGTTTAGTAATGTCCACATAGTGTATGGCCAATAACGGCCGACATGAGAG	73	161	13	89.04	160	1	0
N/A	GGGACACAATGGACGCGCGAGCATGAAGAGCACATAACGCTTAAACATCATTTGCTAACGGCCGACATGAGAG	73	161	13	89.04	183	1	0
N/A	GGGACACAATGGACGATTTTATATCAGGAGTGAGTTGATGAACTGATGTTATGGCTAACGGCCGACATGAGAG	73	161	13	89.04	180	1	0
N/A	GGGACACAATGGACGTCGTTTACTTGAATTGGCTTTTGTTAAAGTTAAGTAGGGGTAACGGCCGACATGAGAG	73	161	13	89.04	157	1	0
N/A	GGGACACAATGGACGATCAATGAATATACTTTCAATGTAGCGAGATGTTAGGCTCTAACGGCCGACATGAGAG	73	161	13	89.04	187	1	0
N/A	GGGACACAATGGACGAATGCTGAAAATCTATAATGGATGAAGTAACGGCCGACATGAGAG	60	161	13	89.04	161	1	0
N/A	GGGACACAATGGACGTTGTTGGCATCAGGCATAATTAAATGTAATGAACCTATACTAACGGCCGACATGAGAG	73	161	13	89.04	165	1	0
N/A	GGGACACAATGGACGTAATCAGAATCGTGGCTTATACTGGAGGGTATTTATCTAGTAACGGCCGACTGAGAG	72	161	13	89.04	170	1	0
N/A	GGGACACAATGGACGAAAAAGGTGGATTGGCCGAAGTAAGGATTTTATAACACTATAACGGCCGACATGAGAG	73	161	13	89.04	175	1	0
N/A	GGGACACAATGGACGGACCACTTAAAGTAGGAACGGTTTATAACTACTTTGTATATAACGGCCGACATGAGAG	73	161	13	89.04	185	1	0
N/A	GGGACACAATGGACGAGTCGATAAATAACAATAGGTAAGAGTACCAATCAAAGGCTAACGGCCGACATGAGAG	73	161	13	89.04	186	1	0
N/A	GGGACACAATGGACGCATACATGATTCGGGCATATTATGAACAGTACAACTTACATAACGGCCGACATGAGAG	73	161	13	89.04	164	1	0
N/A	GGGACACAATGGACGCACAAGCACATACCAAGATTTAATGACTAATGGGTAGCGCTAACGGCCGACATGAGAG	73	161	13	89.04	177	1	0
N/A	GGGACACAATGGACGAGCAATGAAGGATGGATACTTACACGTAACCTATAAATTCTAACGGCCGACATGAGAG	73	161	13	89.04	179	1	0
N/A	GGGACACAATGGACGGTCAATCGTTTGAAATATTTAAACTTAACGGCCGACATGAGAG	58	161	13	89.04	156	1	0
N/A	GGGACACAATGGACGGTGACAAAATCGGAGTGAAGGCAGATTAGCTTTTAACTAGTAACGGCCGACATGAGAG	73	161	13	89.04	158	1	0
N/A	GGGACACAATGGACGCTGTGTAAAGGCGATTTCATATATAGTTTAACGGCCGACATGAGAG	61	161	13	89.04	182	1	0
N/A	GGGACACAATGGACGCATTATCATTATTAACAGATGTTCCAAATGGTAGGGCTCTAACGGCCGACATGAGAG	72	161	13	89.04	181	1	0
N/A	GGGACACAATGGACGATAGCAATATCTAATTTAATGTGGAACGGGTCGTTATTAGTAACGGCCGACATGAGAG	73	161	13	89.04	172	1	0
N/A	GGGACACAATGGACGTCATAGTACATGATTGGAAATAGTTTGGTTGGCATGATTCTAACGGCCGACATGAGAG	73	161	13	89.04	169	1	0
N/A	GGGACACAATGGACGACAATATATAAAATTTTCCTATGGCAAAAAACGTGGGCTGCAATAACGGCCGACATGAGA[illegible]	76	161	13	89.04	176	1	0
N/A	GGGACACAATGGACGAGTATCATTGAGATAACGGGGATTGAGGCCTTATGAAAGCTAACGGCCGACATGAGAG	73	161	13	89.04	178	1	0
N/A	GGGACACAATGGACGCCGAAGGGTTTAGTTAGTGATTTAGAATTAGAATTGACCTAACGGCCGACATGAGAG	72	161	13	89.04	171	1	0
N/A	GGGACACAATGGACGATAATGGATGGCTTATGACCAATCTAGAAATGGGTTGATATAACGGCCGACATGAGAG	73	161	13	89.04	154	1	0
N/A	GGGACACAATGGACGACAATCATAATTTTGGGCCATGTGCTAGCTTATAGGCTTTTAACGGCCGACATGAGAG	73	161	13	89.04	184	1	0
N/A	GGGACACAATGGACGCCGAAATTTTTCAGAATTCATCGAGATTAGAGTTTGGATGTAACGGCCGACATGAGAG	73	161	13	89.04	173	1	0

N/A	GGGACACAATGGACGCAAGGCTGGTATAAATTAAGATATAGATATGTTATTTAATATTAACGGCCGACATGAGAG	75	196	12	82.19	238	1	0
N/A	GGGACACAATGGACGCTAAAACGTTTCTTAGTAAGCCCCGGCATAACGGCCGACATGAGAG	61	196	12	82.19	254	1	0
N/A	GGGACACAATGGACGTGCAGCATTTGATTATGTATAGTGCTTGGCTGGTTAGAAATAACGGCCGACATGAGAG	73	196	12	82.19	212	1	0
N/A	GGGACACAATGGACGATTTGTACCTGGTGAACTGATGTAATTAATGAAAGCCGGTTAACGGCCGACATGAGAG	73	196	12	82.19	249	1	0
N/A	GGGACACAATGGACGAGTCATTAGTTGAAGATCTGTTGTAAATTCGCGATCGGCGTAACGGCCGACATGAGAG	73	196	12	82.19	217	1	0
N/A	GGGACACAATGGACGTATAAAATTAAGTGCGGCGCGAACTGTATAACTGTTTACATAACGGCCGACATGAGAG	73	196	12	82.19	237	1	0
N/A	GGGACACAATGGACGTATTAAGCCTATTTGTAAGGAGATGGATACCCTTATGACGGTGTAACGGCCGACATGAGA	76	196	12	82.19	229	1	0
N/A	GGGACACAATGGACGATATGTTATTGGTGCGTAACGAAGATAGCAATATCTTATGTAACGGCCGACATGAGAG	73	196	12	82.19	203	1	0
N/A	GGGACACAATGGACGCAGTTAGACTAGTTCCTATTTATATTCAAAAGATGGGAGGTAACGGCCGACATGAGAG	73	196	12	82.19	206	1	0
N/A	GGGACACAATGGACGCTTTTACAAATAACGGCCGACATGAGAG	43	196	12	82.19	221	1	0
N/A	GGGACACAATGGACGCGATGAGTTCAACTGGATAAGTAATCTGGTACTAAAGAATAACGGCCGACATGAGAG	72	196	12	82.19	228	1	0
N/A	GGGACACAATGGACGGTTTGTGCTAGTGTATAACGTTTTTCTACTTTTACGGGGGTAACGGCCGACATGAGAG	73	196	12	82.19	222	1	0
N/A	GGGACACAATGGACGCCAGAAATCGTACCGAGTGGATTAACGCAGTGCGAGTTTATAACGGCCGACATGAGAG	73	196	12	82.19	214	1	0
N/A	GGGACACAATGGACGACTTCTATTTAGAACATGAGGGAACTAGCTGATGTTTGGCTAACGGCCGACATGAGAG	73	196	12	82.19	216	1	0
N/A	GGGACACAATGGACGCCTAACTGGAATAATGATTACATATTTCATGATTCTAACCTAACGGCCGACATGAGAG	73	196	12	82.19	211	1	0
N/A	GGGACACAATGGACGTACGTAACACATGTAGATCACTATGTTAGGTTGACTAATGTAACGGCCGACATGAGAG	73	196	12	82.19	195	1	0
N/A	GGGACACAATGGACGTTAGAATATATGATAGCAGATTGTTGATGAATCCTTAGGCTAACGGCCGACATGAGAG	73	196	12	82.19	196	1	0
N/A	GGGACACAATGGACGATTTAAGTTACATATGTATTACCGAGCGTTGACCGCGAAGTAACGGCCGACATGAGAG	73	196	12	82.19	219	1	0
N/A	GGGACACAATGGACGCACCTGCAAATATAAAAACAACAATAAGGAAAGAAATGGCTAACGGCCGACATGAGAG	73	196	12	82.19	199	1	0
N/A	GGGACACAATGGACGCCAGTAACTGAATTAGCTTAAAATAAAGCAACATAACGTATAACGGCCGACATGAGAG	73	196	12	82.19	251	1	0
N/A	GGGACACAATGGACGGAACAGTTGTTGTATATTGAATAAACGAGGCGATAACGTAACGGCCGACATGAGAG	71	196	12	82.19	204	1	0
N/A	GGGACACAATGGACGACTTTAGTACTCCGGGAGGTGTAACGGCCGACATGAGAG	54	196	12	82.19	223	1	0
N/A	GGGACACAATGGACGTAAGCAACGATTGAAACAATCATTTCGATGTGAACATAGCTAACGGCCGACATGAGAG	73	196	12	82.19	220	1	0
N/A	GGGACACAATGGACGTATGTTGAACTGATGAGTGAATTTGGAATCAACAATGAGGTAACGGCCGACATGAGAG	73	196	12	82.19	246	1	0
N/A	GGGACACAATGGACGCCAGAATGATAGCAGGTTTTATGCAACTACAAAGTATTCATAACGGCCGACATGAGAG	73	196	12	82.19	236	1	0
N/A	GGGACACAATGGACGCTTAAACAAAGCAGTACCACAAGATTCCTTGAATAAGGCTAACGGCCGACATGAGAG	72	196	12	82.19	218	1	0
N/A	GGGACACAATGGACGACCGATTTGTTTTTACGGAATAACGGCCGACATGAGAG	53	196	12	82.19	225	1	0
N/A	GGGACACAATGGACGACCAATATAATATATCTATAACGGCCGACATGAGAG	51	196	12	82.19	245	1	0
N/A	GGGACACAATGGACGTGACTACTGGGCAAGAATAAATATTGAAGTTTTAGCAGTATAACGGCCGACATGAGAG	73	196	12	82.19	241	1	0
N/A	GGGACACAATGGACGGAAATGTATCTAACTGGGTAAAAAAACCGAGCTCTTACGTTAACGGCCGACATGAGAG	73	196	12	82.19	213	1	0
N/A	GGGACACAATGGACGCATAGGTATCCTTTAGATGATTTGAGATCTAAGGGGAACGTAACGGCCGACATGAGAG	73	196	12	82.19	239	1	0
N/A	GGGACACAATGGACGCTTAGTTACAGATAGTATACAAATAACATGCAAATGTTGGTAACGGCCGACATGAGAG	73	196	12	82.19	193	1	0
N/A	GGGACACAATGGACGGCAGACATTAAACAATTATATGTGCATATAGCACTGTACGTAACGGCCGACATGAGAG	73	196	12	82.19	224	1	0
N/A	GGGACACAATGGACGGCAACATAAAACTTGTATTTTTTTTAACGGCCGACATGAGAG	57	196	12	82.19	233	1	0
N/A	GGGACACAATGGACGCGCTGAAAATTACGATAACCTTAAACACATTATAGGGCACTAACGGCCGACATGAGAG	73	196	12	82.19	209	1	0
N/A	GGGACACAATGGACGAATACATTATTACCTGATGATGTAAATATCAGGGATTACGTAACGGCCGACATGAGAG	73	196	12	82.19	234	1	0
N/A	GGGACACAATGGACGGTTACTGATGCTAATAGTTGATTCAAAACAATGAAGCTAACGGCCGACATGAGAG	70	196	12	82.19	189	1	0
N/A	GGGACACAATGGACGTCGTATAGAGAAACAATTTATAATTAAATATCTTGGTACTAACGGCCGACATGAGAG	72	196	12	82.19	210	1	0
N/A	GGGACACAATGGACGGGGATGAAAATATAAGATATTCATTATTAAGACGACTTATTAACGGCCGACATGAGAG	73	196	12	82.19	194	1	0
N/A	GGGACACAATGGACGCTGGGATGAGATTTATAAACACATCTATCTTTATAGATCCTAACGGCCGACATGAGAG	73	196	12	82.19	232	1	0
N/A	GGGACACAATGGACGACAGCGGCCTTACCATAGACGATAAATTTATATGCTAGGATAACGGCCGACATGAGAG	73	196	12	82.19	201	1	0
N/A	GGGACACAATGGACGGTACGTGTATATATTGGTACGATACTTATATCGATTAACGGCCGACATGAGAG	68	196	12	82.19	191	1	0
N/A	GGGACACAATGGACGTTATCTAACCATATAATGTTGTACTAATTTCGCAACACGATAACGGCCGACATGAGAG	73	196	12	82.19	207	1	0
N/A	GGGACACAATGGACGTTGTATCGATCTATTAGAGAAATAACAACTCTTAGATGAGTAACGGCCGACATGAGAG	73	196	12	82.19	253	1	0
N/A	GGGACACAATGGACGTATATCTTCTGAATTATGGCATTGAAAAGGACCTATGGCGTAACGGCCGACATGAGAG	73	196	12	82.19	247	1	0
N/A	GGGACACAATGGACGGCAACAGGGTATAGTCAAGATAGTGAGTATTCTACTTAGCTAACGGCCGACATGAGAG	73	196	12	82.19	227	1	0
N/A	GGGACACAATGGACGACCTGATAGACAAGTATTTACATTTGCTAACGGCCGACATGAGAG	60	196	12	82.19	208	1	0
N/A	GGGACACAATGGACGTCGTATAAAACGAACTTAAGAAGAATAGAAAGCTGGTTGGTAACGGCCGACATGAGAG	73	196	12	82.19	188	1	0
N/A	GGGACACAATGGACGGTCTTATTTAACACTATAACGGCCGACATGAGAG	49	196	12	82.19	235	1	0
N/A	GGGACACAATGGACGAACGTTTGGATTCAGTACAATATGTGAAACTCAAGGCTATTAACGGCCGACATGAGAG	73	196	12	82.19	197	1	0
N/A	GGGACACAATGGACGGGACGGAACGAGGAATAGGAAACAAATTATGCCTTGGGTTTAACGGCCGACATGAGAG	73	196	12	82.19	226	1	0
N/A	GGGACACAATGGACGATACCATGCTATAATAATTAACGGCCGACATGAGAG	51	196	12	82.19	243	1	0
N/A	GGGACACAATGGACGTACAAATAATATCGCAAACAATAAATGAAACTTGTCGAGCTAACGGCCGACATGAGAG	73	196	12	82.19	200	1	0
N/A	GGGACACAATGGACGATTTTTGACTAATGCGTGAGGCCTGATATACCACCTGTAGTAACGGCCGACATGAGAG	73	196	12	82.19	192	1	0
N/A	GGGACACAATGGACGTATTTGATTAATCTAAGGGCTAAAAGCGTTGTAATATCGCTAACGGCCGACATGAGAG	73	196	12	82.19	242	1	0
N/A	GGGACACAATGGACGGTACACCGCGTATTAATTTTCTTGGACATAACCATAATCGTAACGGCCGACATGAGAG	73	196	12	82.19	230	1	0
N/A	GGGACACAATGGACGATTAAAGGATTTAGTTCCGGATGATGAACCTATGGCCACGTAACGGCCGACATGAGAG	73	196	12	82.19	190	1	0
N/A	GGGACACAATGGACGACTGATTGTGAGTAGAGTGAATTTTGGAAGCTTTTTACAGTAACGGCCGACATGAGAG	73	196	12	82.19	231	1	0
N/A	GGGACACAATGGACGGAATCGTATTTGGGAGAATGTAAGGTTTTACACCAATTGTAACGGCCGACATGAGAG	72	196	12	82.19	205	1	0
N/A	GGGACACAATGGACGACATCAGATTCATTAGAGATTCCTGAGCTTAGTCAGCCTAACGGCCGACATGAGAG	71	196	12	82.19	198	1	0

N/A	GGGACACAATGGACGCCAATCTTGCCGTATAGATTAATAATATGGTTTAGAACAGTAACGGCCGACATGAGAG	73	196	12	82.19	202	1	0
N/A	GGGACACAATGGACGCTAGTATCAAATTTTATGCATAACGGCCGACATGAGAG	53	196	12	82.19	252	1	0
N/A	GGGACACAATGGACGATGGAGGCTGTTATATTTAATACTATGTTAATGTTGACTATAACGGCCGACATGAGAG	73	196	12	82.19	240	1	0
N/A	GGGACACAATGGACGCAACATAATTTAGAACCGTATACTTTTCAAATCAGGGTTTTAACGGCCGACATGAGAG	73	196	12	82.19	215	1	0
N/A	GGGACACAATGGACGCCATAAGTTATAAGCCATAGTTTAATCAGGGTGAAGTGCTTAACGGCCGACATGAGAG	73	196	12	82.19	248	1	0
N/A	GGGACACAATGGACGTTAGTTTATTTATAGAACAGGTCAGAGTTGACTGGGCTGCTAACGGCCGACATGAGAG	73	263	11	75.34	286	1	0
N/A	GGGACACAATGGACGCTGTAGGAAAGTATATTAGGAGCGAAAGCTTCTCTACACATAACGGCCGACATGAGAG	73	263	11	75.34	264	1	0
N/A	GGGACACAATGGACGATAACAAAACATCGTAATTATACCAACGCAGTCGCGCAGATAACGGCCGACATGAGAG	73	263	11	75.34	314	1	0
N/A	GGGACACAATGGACGCCGAAATATGTAAACGTATAGTATTATAGGTACGATACGATAACGGCCGACATGAGAG	73	263	11	75.34	273	1	0
N/A	GGGACACAATGGACGTTAATACCTAAAAATTTATAACGGCCGACATGAGAG	51	263	11	75.34	287	1	0
N/A	GGGACACAATGGACGTCACCGCACAAAGTACTGGCTGAGATATCATATAGATATATAACGGCCGACATGAGAG	73	263	11	75.34	302	1	0
N/A	GGGACACAATGGACGTTTACAACGACTAATAGAGACCCAGTCGAGCTGGAAATAACGGCCGACATGAGAG	70	263	11	75.34	279	1	0
N/A	GGGACACAATGGACGTTAATACATACCAATAACGGCCGACATGAGAG	47	263	11	75.34	259	1	0
N/A	GGGACACAATGGACGTATGTGTATAACCATGGCCTAACGGCCGACATGAGAG	52	263	11	75.34	265	1	0
N/A	GGGACACAATGGACGCTTGTATAATTAAGAACAGTTTTGGTTGAACTCTTAGGAGTAACGGCCGACATGAGAG	73	263	11	75.34	288	1	0
N/A	GGGACACAATGGACGGATAAACAACCAATTGGGTAACTATATGCTTGGTACGTATTAACGGCCGACATGAGAG	73	263	11	75.34	284	1	0
N/A	GGGACACAATGGACGTTATACGATTAACATGTGCATTTGTTATCCTAGGTGGGCGTAACGGCCGACATGAGAG	73	263	11	75.34	262	1	0
N/A	GGGACACAATGGACGTATACTGTGAGATGGCACATAACGGCCGACATGAGAG	52	263	11	75.34	311	1	0
N/A	GGGACACAATGGACGTACAAATAGTATGCGACTGTTTTGGATACCACAAGATCGGTAACGGCCGACATGAGAG	73	263	11	75.34	305	1	0
N/A	GGGACACAATGGACGCTATTTCACAAATATACCAGATGTACACCTAGTTGAGGCCTAACGGCCGACATGAGAG	73	263	11	75.34	257	1	0
N/A	GGGACACAATGGACGATAGAAATATAAACGATTAACCATTCACCACCAAGGCTTAGTAACGGCCGACATGAGAG	74	263	11	75.34	298	1	0
N/A	GGGACACAATGGACGACCGGAGATAGGTTTTTACAAATAAATGACGTATCGTTTTCTAACGGCCGACATGAGAG	74	263	11	75.34	275	1	0
N/A	GGGACACAATGGACGATAAATATTTGTTACCAGGTCGGTTGATGTAAGGGATGCATAACGGCCGACATGAGAG	73	263	11	75.34	278	1	0
N/A	GGGACACAATGGACGAACCAATATATGCCAATGACTGCATGACAGGAGGTCATATAACGGCCGACATGAGAG	72	263	11	75.34	297	1	0
N/A	GGGACACAATGGACGCTAGTACTGTAAAATTATCTGGATTGAGACAGATTCATGGTAACGGCCGACATGAGAG	73	263	11	75.34	289	1	0
N/A	GGGACACAATGGACGCATAAGGCATGTGAAGGATAAAATTTTACCAACTTTTACCTAACGGCCGACATGAGAG	73	263	11	75.34	313	1	0
N/A	GGGACACAATGGACGAGATCGTATTATGACAGGATACAAAGATAATAACGGCCGACATGAGAG	63	263	11	75.34	301	1	0
N/A	GGGACACAATGGACGCATATTATAGTGAGATCCTGAGAAACCCACATTCGTTGGCTTAACGGCCGACATGAGAG	74	263	11	75.34	295	1	0
N/A	GGGACACAATGGACGTAGGAGTCGACCGAAGTTTATATAAGAACGTAATATAGCTAACGGCCGACATGAGAG	72	263	11	75.34	261	1	0
N/A	GGGACACAATGGACGGAAATCCAATACTGTACCGAGTGTAATGTCATATTAAGCCTAACGGCCGACATGAGAG	73	263	11	75.34	263	1	0
N/A	GGGACACAATGGACGTACCGTGATTATATAACATGTGCTTAAACTACATGAGTGGTAACGGCCGACATGAGAG	73	263	11	75.34	268	1	0
N/A	GGGACACAATGGACGTAAAGTCTATATATAATCGAGTGTGAGGGCGGTTAGCAATAACGGCCGACATGAGAG	72	263	11	75.34	309	1	0
N/A	GGGACACAATGGACGTTGGGATTCAAGGTGCTTCCGTATAAATGATATGAAGGCATAACGGCCGACATGAGAG	73	263	11	75.34	272	1	0
N/A	GGGACACAATGGACGATTTATACAGAAAGTGAATTGGAAATAATCCTAGGGGCTTAACGGCCGACATGAGAG	72	263	11	75.34	269	1	0
N/A	GGGACACAATGGACGTGAACTATATTGGAGATAATCATGTATGAAGGCAAGGTATAACGGCCGACATGAGAG	72	263	11	75.34	303	1	0
N/A	GGGACACAATGGACGTCACTAACACAAGGATTAAAGATTCGTAGGTTGGTAACGGCCGACATGAGAG	67	263	11	75.34	281	1	0
N/A	GGGACACAATGGACGTTCCCATGTAGGTTATTCTGTTAGTTGAGATTATTTACGCTAACGGCCGACATGAGAG	73	263	11	75.34	293	1	0
N/A	GGGACACAATGGACGTAAAGGAGGCAATTATTATGTTCGTAGCGATATGAGCACTTAACGGCCGACATGAGAG	73	263	11	75.34	304	1	0
N/A	GGGACACAATGGACGGATGCTATAACAATAGAAGTTACGATAACTAACTTATCGATAACGGCCGACATGAGAG	73	263	11	75.34	307	1	0
N/A	GGGACACAATGGACGTATAGAAATAAATTCGAAATCAAATTGCTGAATGAAGGCTTAACGGCCGACATGAGAG	73	263	11	75.34	296	1	0
N/A	GGGACACAATGGACGGAAGATATTTATTTCAGACAGGTAACGAATGTTGAGGCTTAACGGCCGACATGAGAG	72	263	11	75.34	290	1	0
N/A	GGGACACAATGGACGATACGGGATAAAGAGACCAAAGATAACTATATTGGTAAAGTAACGGCCGACATGAGAG	73	263	11	75.34	308	1	0
N/A	GGGACACAATGGACGCGTGATTTTATAAGACTTTAATGCACCATAACGTACGATATAACGGCCGACATGAGAG	73	263	11	75.34	299	1	0
N/A	GGGACACAATGGACGATGAAAAGATATATGTATGTTTGGCTGATTGATTTAATTATAACGGCCGACATGAGAG	73	263	11	75.34	282	1	0
N/A	GGGACACAATGGACGATTAGAAAATTGACTATATGGTTGAACCAGCTTTGTTGGCTAACGGCCGACATGAGAG	73	263	11	75.34	277	1	0
N/A	GGGACACAATGGACGTGCCAGTATCTAGGGAAAGTGTTTAAGGATACCTATAGTATAACGGCCGACATGAGAG	73	263	11	75.34	258	1	0
N/A	GGGACACAATGGACGTATTACTCAGGTCTAGCTATAACGCTAAAGATTACATGCTTAACGGCCGACATGAGAG	73	263	11	75.34	271	1	0
N/A	GGGACACAATGGACGTAGTTTATATTTAAACCAATCTATGGTGGTTATACCACGGCTAACGGCCGACATGAGAG	74	263	11	75.34	274	1	0
N/A	GGGACACAATGGACGTGATCAAAATGGAAGGCCTAATGTTATATACTGTTTTGTATAACGGCCGACATGAGAG	73	263	11	75.34	255	1	0
N/A	GGGACACAATGGACGATGATTAGAGTTAATGACCAATGTTGAAGTAATGTGAGGCTAACGGCCGACATGAGAG	73	263	11	75.34	285	1	0
N/A	GGGACACAATGGACGTAGCGTTTAAAATGGCGGAGATAGAATGTAAGTTTAACATAACGGCCGACATGAGAG	72	263	11	75.34	294	1	0
N/A	GGGACACAATGGACGTACCGAACACGAACAATTTGCCCAAGACATATTGGGCTCGTAACGGCCGACATGAGAG	73	263	11	75.34	276	1	0
N/A	GGGACACAATGGACGAGCTGGTGAATACAGTAAGGTCTTCGTAAGAAGCATAAACTAACGGCCGACATGAGAG	73	263	11	75.34	270	1	0
N/A	GGGACACAATGGACGAGGCAAAATAGTAGTAGAGTCAATTATATGAATTGCTGTATAACGGCCGACATGAGAG	73	263	11	75.34	306	1	0
N/A	GGGACACAATGGACGTACAAAGAACTCTTGCCTGAACCACAATTATGATCAGCTCTAACGGCCGACATGAGAG	73	263	11	75.34	266	1	0
N/A	GGGACACAATGGACGCACAAAATCATATAAGTGCACTTTAGTGTAGAGGCCCGATAACGGCCGACATGAGAG	72	263	11	75.34	267	1	0
N/A	GGGACACAATGGACGAACAAATAATCGTGAATACTACATCTTGTTAGCCAACAAATAACGGCCGACATGAGAG	73	263	11	75.34	310	1	0
N/A	GGGACACAATGGACGTGAACCAAATGGGCGTGGACGATTAACTTTAATGATTGACTAACGGCCGACATGAGAG	73	263	11	75.34	300	1	0
N/A	GGGACACAATGGACGTATGTTTTCTTACACACTTTCAATAGGCACGCGATTGAGCTAACGGCCGACATGAGAG	73	263	11	75.34	292	1	0
N/A	GGGACACAATGGACGGATTATAGAGCAATACTTACAGTAAATGTGAGAAGGAAGCCTAACGGCCGACATGAGAG	74	263	11	75.34	256	1	0

N/A	GGGACACAATGGACGACCTCAATGTGGGATAAACGGCAAAGTATGATTTCTTTAACGGCCGACATGAGAG	70	263	11	75.34	291	1	0
N/A	GGGACACAATGGACGCTGTCTGCAATTAAACGCTGATTTGGGCGGTATTAGAGCTTAACGGCCGACATGAGAG	73	263	11	75.34	260	1	0
N/A	GGGACACAATGGACGAATGGTTCAAATAGTTGATCGACGTAACAATAGGTTAGGCTAACGGCCGACATGAGAG	73	263	11	75.34	280	1	0
N/A	GGGACACAATGGACGCAAGTAGATCTATTAGAACCTCAGATAGGCTTTAATACTCTAACGGCCGACATGAGAG	73	263	11	75.34	312	1	0
N/A	GGGACACAGTGGACGGCAAAATCAAAGTGAGTAAAAATATATCAGTGATGACCACTAACGGCCGACATGAGAG	73	263	11	75.34	283	1	0
N/A	GGGACACAATGGACGCCTTCTTACATCACGTCGATAATTATTTATCTATTGGAACGTAACGGCCGACATGAGAG	74	324	10	68.49	381	1	0
N/A	GGGACACAATGGACGTGAAAATAGTCACAACAAACAATTCAAATTTACACACGGCGTAACGGCCGACATGAGAG	74	324	10	68.49	387	1	0
N/A	GGGACACAATGGACGTAACGAAAGTAACTAGTATAATTTGATGATATTAGTGTGATAACGGCCGACATGAGAG	73	324	10	68.49	317	1	0
N/A	GGGACACAATGGACGACATTAAGTTTTAACGCTTAGTACACTATTGTCTCGGGGCTAACGGCCGACATGAGAG	73	324	10	68.49	399	1	0
N/A	GGGACACAATGGACGAGCAAAGATACCACGCTATTAAAGATATTAGGTAGGGTAACGGCCGACATGAGAG	70	324	10	68.49	331	1	0
N/A	GGGACACAATGGACGATCTAATCTTACATTGAGAGCTAAGTGTTGGGTAGTGTAATAACGGCCGACATGAGAG	73	324	10	68.49	346	1	0
N/A	GGGACACAATGGACGTGAAACGTAACCAAACACCGAAAAGGTCGATAACGGCCGACATGAGAG	63	324	10	68.49	324	1	0
N/A	GGGACACAATGGACGTGTAGGTCAATAGTAGGTATTACAATTTATCTTGCTATCTAACGGCCGACATGAGAG	72	324	10	68.49	373	1	0
N/A	GGGACACAATGGACGTATATATAATAACGCACATTTAGACACTATGGCGACTGGATAACGGCCGACATGAGAG	73	324	10	68.49	389	1	0
N/A	GGGACACAATGGACGACATTGCATATCCAGAACACTTAAATGTAAGCCACAGATGTAACGGCCGACATGAGAG	73	324	10	68.49	405	1	0
N/A	GGGACACAATGGACGGATAGAATATTGAGATACCCGCATGGATGGCATAGTAACTTAACGGCCGACATGAGAG	73	324	10	68.49	350	1	0
N/A	GGGACACAATGGACGCTGTTAATTATGTGAAGGTATTAACTCCTGCTTTAGGATAACGGCCGACATGAGAG	71	324	10	68.49	336	1	0
N/A	GGGACACAATGGACGGAACAATTTTCTTGTAATTACTTATGTAGGACGGTGAACGTAACGGCCGACATGAGAG	73	324	10	68.49	340	1	0
N/A	GGGACACAATGGACGTCGGTTATTTATAACGGCCGACATGAGAG	44	324	10	68.49	38	2	5
N/A	GGGACACAATGGACGATGATATGTTTTAAATATGTGGAACAACTTATGTACGGAATAACGGCCGACATGAGAG	73	324	10	68.49	388	1	0
N/A	GGGACACAATGGACGGTTCGCGCTTACAATTAAGACTAAAAAGGATGAGCTTAAAGTAACGGCCGACATGAGAG	74	324	10	68.49	356	1	0
N/A	GGGACACAATGGACGCTCAACCTTGAGTTAAGTGATATACAATCTTTATAAAGGGTAACGGCCGACATGAGAG	73	324	10	68.49	332	1	0
N/A	GGGACACAATGGACGGACGGATATAATGGCGTAGCGTTTTATATGTTATGCAGCTAACGGCCGACATGAGAG	72	324	10	68.49	385	1	0
N/A	GGGACACAATGGACGTAATTATCATGAAGGAGAGTATAGAATGCTTGAACACTGTATAACGGCCGACATGAGAG	74	324	10	68.49	347	1	0
N/A	GGGACACAATGGACGTGCTTATACGTATGGTAGACATTATATGAGGCCAAACTATAACGGCCGACATGAGAG	72	324	10	68.49	355	1	0
N/A	GGGACACAATGGACGACAACGTAAAGAAATAGTACATTATACTATTGTTAAGACGTAACGGCCGACATGAGAG	73	324	10	68.49	329	1	0
N/A	GGGACACAATGGACGTATAACACTCAAATGACGGAGTATACATTACGACGACCGGTAACGGCCGACATGAGAG	73	324	10	68.49	353	1	0
N/A	GGGACACAATGGACGATATTTGACATACAAGTTTCCTAATCATAGTGAGAGGCTCTAACGGCCGACATGAGAG	73	324	10	68.49	384	1	0
N/A	GGGACACAATGGACGGTTATTTGACGAATTTAACACGTGCCCTAGAGTGGAAGGCTAACGGCCGACATGAGAG	73	324	10	68.49	323	1	0
N/A	GGGACACAATGGACGATATAATATTTACAGAACGACTTTCTCTATGAAACGAGCGTAACGGCCGACATGAGAG	73	324	10	68.49	383	1	0
N/A	GGGACACAATGGACGATCAGATAGAGTTAACGGCCGACATGAGAG	45	324	10	68.49	363	1	0
N/A	GGGACACAATGGACGTTACAGCTTTTTGCGTACTAATTAGAGCGCGATAACTAGATAACGGCCGACATGAGAG	73	324	10	68.49	326	1	0
N/A	GGGACACAATGGACGTTACTTCATTAATGGAAAGGGTAACGGCCGACATGAGAG	54	324	10	68.49	333	1	0
N/A	GGGACACAATGGACGCTAAAACATAATTGAAGGCACATTCCCAAGGCACGAGGAATAACGGCCGACATGAGAG	73	324	10	68.49	401	1	0
N/A	GGGACACAATGGACGTTAAGAATGGGTATATAATATGTACGCAGACATAGGGACTTAACGGCCGACATGAGAG	73	324	10	68.49	376	1	0
N/A	GGGACACAATGGACGGTAGCTCAATTAAAGAATAGAGAAGGTTTGCCGATCTAACGGCCGACATGAGAG	69	324	10	68.49	339	1	0
N/A	GGGACACAATGGACGGTTGATTTGAGATTGGATAGAGTTAGTAACCAATTTGACATAACGGCCGACATGAGAG	73	324	10	68.49	404	1	0
N/A	GGGACACAATGGACGGGCATAGCTAGACACAAAACTAACGGCCGACATGAGAG	53	324	10	68.49	358	1	0
N/A	GGGACACAATGGACGACATAGTGCCTACATATTCTGGGGCTAGAGTTATTTATACTAACGGCCGACATGAGAG	73	324	10	68.49	325	1	0
N/A	GGGACACAATGGACGAATCAACAAATTATTTCATATGTGTATCTCGTCGCAGCCTTAACGGCCGACATGAGAG	73	324	10	68.49	397	1	0
N/A	GGGACACAATGGACGGATATGAACATATTCCTTGGCGAATATAACGGCCGACATGAGAG	59	324	10	68.49	344	1	0
N/A	GGGACACAATGGACGGATTGAAGCTACGGAATGGTAGTGAAATAACGGCCGACATGAGAG	60	324	10	68.49	398	1	0
N/A	GGGACACAATGGACGGTGATAGGTGGTTTCTGCAAGTAATTGCACTACCATTTGTAACGGCCGACATGAGAG	72	324	10	68.49	341	1	0
N/A	GGGACACAATGGACGTGCTAGTTAATTTGACTTCTTAAGTGAACTGAGTCAGCAATAACGGCCGACATGAGAG	73	324	10	68.49	319	1	0
N/A	GGGACACAATGGACGCTGATAAACTGAAAATCAATAAACAGATCGAACAGTATATTAACGGCCGACATGAGAG	73	324	10	68.49	403	1	0
N/A	GGGACACAATGGACGACATTAATTAGATGACAAGGATGGCTGCCTAACGTGATGTTAACGGCCGACATGAGAG	73	324	10	68.49	380	1	0
N/A	GGGACACAATGGACGATGACGGCAGATCATTTGCTTGATTAAAACGAGAAATAGTAACGGCCGACATGAGAG	72	324	10	68.49	366	1	0
N/A	GGGACACAATGGACGTAAACTAATGCTAAATGACCAGCCTATAGCTGATACCAGTTAACGGCCGACATGAGAG	73	324	10	68.49	334	1	0
N/A	GGGACACAATGGACGCCGTATAATAAAACAACTTTGAATTAAATAACATCGAAGCTAACGGCCGACATGAGAG	73	324	10	68.49	321	1	0
N/A	GGGACACAATGGACGTGAGGGCGTATTTAAGTGATTGATGTAGTCAGTCACCACATAACGGCCGACATGAGAG	73	324	10	68.49	374	1	0
N/A	GGGACACAATGGACGAATGTCATTTGTTCTCGGGCCTTATAAGTTACAGTTGACATAACGGCCGACATGAGAG	73	324	10	68.49	369	1	0
N/A	GGGACACAATGGACGAGGTATGCAGAATAACTGTTAATTGTTTGGACCGGTGGCATAACGGCCGACATGAGAG	73	324	10	68.49	368	1	0
N/A	GGGACACAATGGACGTGAGAATAGATAGTATCATCGTGAACGAGAGTGACGGCATTAACGGCCGACATGAGAG	73	324	10	68.49	357	1	0
N/A	GGGACACAATGGACGGTGAAACACTGAGAAAAAGAGTCGTTACATGTTGTAACGGTAACGGCCGACATGAGAG	73	324	10	68.49	394	1	0
N/A	GGGACACAATGGACGTATATTAGTACGTTGATGTAACAATGACAATCTAGTTGGTTAACGGCCGACATGAGAG	73	324	10	68.49	371	1	0
N/A	GGGACACAATGGACGGCTATGTGAATATTTAACACTACGAGACGATGTACGTACGTAACGGCCGACATGAGAG	73	324	10	68.49	382	1	0
N/A	GGGACACAATGGACGAATATCTAGAACTGGACCAATTATAGGCCTATTAAATTAGTAACGGCCGACATGAGAG	73	324	10	68.49	370	1	0
N/A	GGGACACAATGGACGAAATAGAATTAAACATACTGAGAATATGTTAGGATGACGATAACGGCCGACATGAGAG	73	324	10	68.49	360	1	0
N/A	GGGCACAATGGACGGTAAACCACTTGGAGATACTATATCAAATGCATCACAGGTTAACGGCCGACATGAGAG	72	324	10	68.49	352	1	0
N/A	GGGACACAATGGACGTTTTAAATCATAAATATAATGGTTAAATGCTATGAAGGCATAACGGCCGACATGAGAG	73	324	10	68.49	335	1	0

N/A	GGGACACAATGGACGCTATCGAATTACCTATGCTCGAATACAAGGCAGAAGGACATAACGGCCGACATGAGAG	73	324	10	68.49	390	1	0
N/A	GGGACACAATGGACGTGCCGAGCTTGACTTAGATGATTAAAAATGCTAGGATTGCTAACGGCCGACATGAGAG	73	324	10	68.49	367	1	0
N/A	GGGACACAATGGACGGTCAATTTATATAAGAATACATAACGGCCGACATGAGAG	54	324	10	68.49	362	1	0
N/A	GGGACACAATGGACGTTTCACGGCTAAAAAAGACATTCGGTTAATAACGAAGTGTCTAACGGCCGACATGAGAG	74	324	10	68.49	343	1	0
N/A	GGGACACAATGGACGTGAAATTTAAATAATATCAAGTGAGGCGTGTGTTTGAACCTAACGGCCGACATGAGAG	73	324	10	68.49	315	1	0
N/A	GGGACACAATGGACGTGATGACCTATGTATGCCGTGTATAGATTATATTGATATGTAACGGCCGACATGAGAG	73	324	10	68.49	328	1	0
N/A	GGGACACAATGGACGCTAAGCATACGATTTACGATTTGAAAATTTGTAGGATGTTTAACGGCCGACATGAGAG	73	324	10	68.49	318	1	0
N/A	GGGACACAATGGACGTCCGTATATTATTCAAATTAGTAACGGCCGACATGAGAG	54	324	10	68.49	351	1	0
N/A	GGGACACAATGGACGTAACGGCCGACATGAGAG	33	324	10	68.49	5	4	3
N/A	GGGACACAATGGACGCATGAGACCGTAAGGATAACAAAAAATATTTAATTGAGCCATAACGGCCGACATGAGAG	74	324	10	68.49	327	1	0
N/A	GGGACACAATGGACGCGATTAAACGACCTTGGAACTACTACATATGTTAGGCAAGTAACGGCCGACATGAGAG	73	324	10	68.49	320	1	0
N/A	GGGACACAATGGACGGATAACACTAATAAATGCAGACATTGTATATGGAGGTCAGTAACGGCCGACATGAGAG	73	324	10	68.49	375	1	0
N/A	GGGACACAATGGACGTTTAACGACAGCATAATTTACAAAAGATGATGTTGAGTACTAACGGCCGACATGAGAG	73	324	10	68.49	392	1	0
N/A	GGGACACAATGGACGCGGTAAATTATTGACAATCTATTAACATGATGAGTGCAGTAACGGCCGACATGAGAG	72	324	10	68.49	400	1	0
N/A	GGGACACAATGGACGATCCTGTTTTAGGAGGATGTCTTGACGTTTCCAAACGGCATAACGGCCGACATGAGAG	73	324	10	68.49	316	1	0
N/A	GGGACACAATGGACGGCGCATACTCCTAGCTGGAAACAGAATAGAATAATACGGGTAACGGCCGACATGAGAG	73	324	10	68.49	377	1	0
N/A	GGGACACAATGGACGCTTAAACTTATATGTAACGGCCGACATGAGAG	47	324	10	68.49	391	1	0
N/A	GGGACACAATGGACGTATACTAATAATTTCCGGATATATAAACAGATCTTTTAGCTAACGGCCGACATGAGAG	73	324	10	68.49	338	1	0
N/A	GGGACACAATGGACGGCTAACAATGGTATTGTAGAATTCTCAGACTTTTGCTGGGTAACGGCCGACATGAGAG	73	324	10	68.49	372	1	0
N/A	GGGACACAATGGACGTTGGAACCTTTAAAATAAGAGATACATGGAGACCTTGATCTAACGGCCGACATGAGAG	73	324	10	68.49	396	1	0
N/A	GGGACACAATGGACGATTATTAAAGTAGTGGATTGAAAACCATATTCTGTGGGCTTAACGGCCGACATGAGAG	73	324	10	68.49	359	1	0
N/A	GGGACACAATGGACGCAATTATGTAAGGTCGTGCACATGACAATCAACTTTATGTAACGGCCGACATGAGAG	72	324	10	68.49	365	1	0
N/A	GGGACACAATGGACGCTATAGATTAGATAGATTGATGTAAAAATGTGGTCGCGCTAACGGCCGACATGAGAG	72	324	10	68.49	342	1	0
N/A	GGGACACAATGGACGTTTAGTACTTTATGCCTACAGGCTACACCACATTAAGTGGTAACGGCCGACATGAGAG	73	324	10	68.49	361	1	0
N/A	GGGACACAATGGACGACTAAATAATGATATGTTGATCTATCCATGCATAATTCAATAACGGCCGACATGAGAG	73	324	10	68.49	354	1	0
N/A	GGGACACAATGGACGCCAGTTTAATTGTAGCGGCTTCGATGTTTAAAAGAACGAGTAACGGCCGACATGAGAG	73	324	10	68.49	402	1	0
N/A	GGGACACAATGGACGAGGTATTATTTCTGTTCGGAGGATTGATAGTCCCGTGGCTTAACGGCCGACATGAGAG	73	324	10	68.49	393	1	0
N/A	GGGACACAATGGACGTTTTTAATAAAGCACCAATCTGCTTAAAGAGAGTAGCTGGTAACGGCCGACATGAGAG	73	324	10	68.49	395	1	0
N/A	GGGACACAATGGACGTTAACCAAGCTAGACGATGTAAGTTGGAAGGTTTTGGAGTAACGGCCGACATGAGAG	72	324	10	68.49	364	1	0
N/A	GGGACACAATGGACGAATATTTAACGGCCGACATGAGAG	39	324	10	68.49	5	3	4
N/A	GGGACACAATGGACGAGGTTGGGCTTCTATGAGTAAAAGCGATAAGCGATTACTGTAACGGCCGACATGAGAG	73	324	10	68.49	322	1	0
N/A	GGGACACAATGGACGGACACTCTGAACTATTAACAAATAGGAGATCTTGAGGATATAACGGCCGACATGAGAG	73	324	10	68.49	345	1	0
N/A	GGGACACAATGGACGTATTTATATAGGGACATTGAACCTGCCGGTCAATAGCATCTAACGGCCGACATGAGAG	73	324	10	68.49	348	1	0
N/A	GGGACACAATGGACGTTCTCATCGATTATTTAACATAGTTGGAGACGTGAAGGCCGTAACGGCCGACATGAGAG	74	324	10	68.49	337	1	0
N/A	GGGACACAATGGACGGATTTTACCTTTTGAAGTGACAAACCTGGAGTATTGGCCTTAACGGCCGACATGAGAG	73	324	10	68.49	379	1	0
N/A	GGGACACAATGGACGTACTAACACATAACTATATTACCTATACACTGCATGGGCTTAACGGCCGACATGACGAG	74	324	10	68.49	330	1	0
N/A	GGGACACAATGGACGAACCTATGTGAAATTCTAAAGGCTGTTTAAACTACTGGTAACGGCCGACATGAGAG	71	324	10	68.49	349	1	0
N/A	GGGACACAATGGACGGAGGGTAAATACATTTTGACATAGCAAGACGGTTGCATGTTAACGGCCGACATGAGAG	73	418	9	61.64	449	1	0
N/A	GGGACACAATGGACGCTGAACTTATTTTATTACAACATCTATGAAGGCTAGATGATAACGGCCGACATGAGAG	73	418	9	61.64	480	1	0
N/A	GGGACACAATGGACGTATTAAGTTTTTGTATGAACCAGAATCTGTGACGGACCGGTAACGGCCGACATGAGAG	73	418	9	61.64	494	1	0
N/A	GGGACACAATGGACGATATTAATGAACAGGTGCCAGTCACAATTCTTGACAGGTCTAACGGCCGACATGAGAG	73	418	9	61.64	491	1	0
N/A	GGGACACAATGGACGTAACACGTAAAGGTTGACTTAGAAAAGTACACAATATGCGTAACGGCCGACATGAGAG	73	418	9	61.64	408	1	0
N/A	GGGACACAATGGACGCCAGTAACATCAGAGATATTTTTCATCGGTGTAGACGATATAACGGCCGACATGAGAG	73	418	9	61.64	498	1	0
N/A	GGGACACAATGGACGGGATAATATTTTTTACCTGTACCATCCGTTATAGTAGACATAACGGCCGACATGAGAG	73	418	9	61.64	463	1	0
N/A	GGGACACAATGGACGTTATATAATACATATACATCTTGAGCGTGGACATTAGGCTTAACGGCCGACATGAGAG	73	418	9	61.64	466	1	0
N/A	GGGACACAATGGACGTTAAATTATCACCTGAACCTGAGACATGGGATAGGATGTATAACGGCCGACATGAGAG	73	418	9	61.64	470	1	0
N/A	GGGACACAATGGACGACCAAATCGTGGTAAAATATCTAAATTCACTACACGCTAATAACGGCCGACATGAGAG	73	418	9	61.64	473	1	0
N/A	GGGACACAATGGACGCTAATTTTTGTGCAAACATGGGAGCCTATATACATACACGTAACGGCCGACATGAGAG	73	418	9	61.64	523	1	0
N/A	GGGACACAATGGACGACATAATAAATATGTAGACGCACTTTGAAGACCTACGGCTTAACGGCCGACATGAGAG	73	418	9	61.64	465	1	0
N/A	GGGACACAATGGACGTATGAAGATGATTTATTGCATAACAGATCAATGTAGGGCTTAACGGCCGACATGAGAG	73	418	9	61.64	472	1	0
N/A	GGGACACAATGGACGGTTATGTTACTCATTTCGTGTGGGGTTTATACCCAATGCGTAACGGCCGACATGAGAG	73	418	9	61.64	450	1	0
N/A	GGGACACAATGGACGTAATGCCAACCGGGCCTACTAATTACACCTTGTAGTGTATAACGGCCGACATGAGAG	72	418	9	61.64	504	1	0
N/A	GGGACACAATGGACGTAATGACTATTGCCATCAAAGCTAGGCGTTTGATTCAGCATAACGGCCGACATGAGAG	73	418	9	61.64	508	1	0
N/A	GGGACACAATGGACGCATTTAAACCACATCACTAAAACAATTAAGAGATACATGGTAACGGCCGACATGAGAG	73	418	9	61.64	531	1	0
N/A	GGGACACAATGGACGGTCATGTTATGTTAATTCGTTTTTAACTAAGGGTACAAGCATAACGGCCGACATGAGAG	74	418	9	61.64	500	1	0
N/A	GGGACACAATGGACGTTAGATTAGAGACCTCGAGATATTTTAAATCGGGGAGTATTAACGGCCGACATGAGAG	73	418	9	61.64	446	1	0
N/A	GGGACACAATGGACGGGAAGTTGCAGGACGATGATTATAATTCTTATGATTCCGGTAACGGCCGACATGAGAG	73	418	9	61.64	415	1	0
N/A	GGGACACAATGGACGGAAAAAAATGCGGTATATTAGGTGCCGAAAGGTTCTAACCTAACGGCCGACATGAGAG	73	418	9	61.64	464	1	0
N/A	GGGACACAATGGACGACTTTATCTTTAGATGCTGTGATATAGGCACATCAGAAGGTAACGGCCGACATGAGAG	73	418	9	61.64	520	1	0
N/A	GGGACACAATGGACGGGACTGCTACGATATATATTTTAGAATTATAGACGGATGGTAACGGCCGACATGAGAG	73	418	9	61.64	421	1	0

N/A	GGGACACAATGGACGACCTTTACAAATATGAATTAGCAGAAACGGCTATTACGATGTAACGGCCGACATGAGAG	74	418	9	61.64	545	1	0
N/A	GGGACACAATGGACGTCATTTTAAACGTACAGTGGCATCTGTGTTGGCAGTAAATTAACGGCCGACATGAGAG	73	418	9	61.64	536	1	0
N/A	GGGACACAATGGACGTTTAGCAAGATTTATTGATACTTAACGGCCGACATGAGAG	55	418	9	61.64	516	1	0
N/A	GGGACACAATGGACGCAAAGATAATTTTAGCACTATTAACCAGCATGAGGGCTGATAACGGCCGACATGAGAG	73	418	9	61.64	503	1	0
N/A	GGGACACAATGGACGTGGTATGATATATTTCCATGTTACTTAGATCCAGGCTGAACTAACGGCCGACATGAGAG	74	418	9	61.64	479	1	0
N/A	GGGACACAATGGACGGACGTGTGCGGCTGATATATATTAATAATCTGCAAATGCATAACGGCCGACATGAGAG	73	418	9	61.64	477	1	0
N/A	GGGACACAATGGACGTAAAAGTACGAAACACCTATGGTACCGCGGCATATATACATAACGGCCGACATGAGAG	73	418	9	61.64	430	1	0
N/A	GGGACACAATGGACGTATCAAACGATGATGGGTGACATGCGTTCAAGCCGAATTCTAACGGCCGACATGAGAG	73	418	9	61.64	544	1	0
N/A	GGGACACAATGGACGATAACATCAATTGAAGGTATTTTACTAGAAAGAACAAGAGTAACGGCCGACATGAGAG	73	418	9	61.64	502	1	0
N/A	GGGACACAATGGACGCTTGGGATGACAGTATGTGAATGGATTATTACGTACATCATAACGGCCGACATGAGAG	73	418	9	61.64	549	1	0
N/A	GGGACACAATGGACGATACTAAGACTACCTTACCAACTTACATGTAGTCAGGTAATAACGGCCGACATGAGAG	73	418	9	61.64	471	1	0
N/A	GGGACACAATGGACGTTAAGGTTTAACAATAAATAAGATGTGAGTCTTTATGGACTAACGGCCGACATGAGAG	73	418	9	61.64	493	1	0
N/A	GGGACACAATGGACGGTACGTGAATTGATGTTGGAACTGTTTATCAGACCAGGTATAACGGCCGACATGAGAG	73	418	9	61.64	436	1	0
N/A	GGGACACAATGGACGTGACACGTCGGATACAGGTTTGAGATAATATAACACAGACTAACGGCCGACATGAGAG	73	418	9	61.64	433	1	0
N/A	GGGACACAATGGACGGACTTTAATGTAATTATTTGCTTAACGGCCGACATGAGAG	55	418	9	61.64	460	1	0
N/A	GGGACACAATGGACGACCAGTATTAACTACGAGATATATTTAAGATAGCGGGTGGTAACGGCCGACATGAGAG	73	418	9	61.64	455	1	0
N/A	GGGACACAATGGACGCTAGAGTTACTAGAAGATCAAGCAGATCGCATCTTTAACATAACGGCCGACATGAGAG	73	418	9	61.64	474	1	0
N/A	GGGACACAATGGACGATGATAAGTTACGTATGTATTTCCAGGGCTGTGAAGTGACTAACGGCCGACATGAGAG	73	418	9	61.64	443	1	0
N/A	GGGACACAATGGACGACAAGTCATTAATCCAATCGAAGTGTGGGGTTGTTGATGATAACGGCCGACATGAGAG	73	418	9	61.64	420	1	0
N/A	GGGACACAATGGACGGCATGTACGTAAATACTAACAGGAGTTAAAACTGGGATACTAACGGCCGACATGAGAG	73	418	9	61.64	489	1	0
N/A	GGGACACAATGGACGGACCTTAAGACAGCACATATGAACGTAATATTATGCAGTGTAACGGCCGACATGAGAG	73	418	9	61.64	451	1	0
N/A	GGGACACAATGGACGATAAAGAATCAAATAGTTATCCATAGTCTACCATGGGGCGTAACGGCCGACATGAGAG	73	418	9	61.64	490	1	0
N/A	GGGACACAATGGACGTAACTCAAATATCAATAGGCGGGAGCTAAACTTAATTGAACTAACGGCCGACATGAGAG	74	418	9	61.64	537	1	0
N/A	GGGACACAATGGACGTTTCAATATTATGATAACATTCATGCTATTCTGTTGGGCTTAACGGCCGACATGAGAG	73	418	9	61.64	487	1	0
N/A	GGGACACAATGGACGACTGGCTAAATTCAATGATTTGGAAATAACGGCCGACATGAGAG	59	418	9	61.64	511	1	0
N/A	GGGACACAATGGACGTTATATGACGGCAGAATGAATGTTTTGATCTACTACAGTATAACGGCCGACATGAGAG	73	418	9	61.64	407	1	0
N/A	GGGACACAATGGACGTATTACATTTCTTTGGGCGGTGTACGCTATTTCGAAAGGGTAACGGCCGACATGAGAG	73	418	9	61.64	441	1	0
N/A	GGGACACAATGGACGTACCAGGCTAGAAATATCAAAGCATAAATTACTTTAGCTGTAACGGCCGACATGAGAG	73	418	9	61.64	453	1	0
N/A	GGGACACAATGGACGTCTTTAAACGTTAACTGACATACTAGTCGTATATTCAGGCGCTAACGGCCGACATGAGAG	75	418	9	61.64	525	1	0
N/A	GGGACACAATGGACGTAATAGAAGGTTAGAAATCAAACAGTAGATACCTAATACTAACGGCCGACATGAGAG	72	418	9	61.64	475	1	0
N/A	GGGACACAATGGACGTGGACGCCGATATGTGATTAGATATTGTTTTTAGTCTTCGTAACGGCCGACATGAGAG	73	418	9	61.64	507	1	0
N/A	GGGACACAATGGACGGATTTTATGGTATTCAGATCTAGACTGTCTGGCGACCGGTAACGGCCGACATGAGAG	72	418	9	61.64	444	1	0
N/A	GGGACACAATGGACGGAAGTTAACAATTATAGGTAGTAAATAACTGTAGCTAGTATAACGGCCGACATGAGAG	73	418	9	61.64	517	1	0
N/A	GGGACACAATGGACGAGCAATTTATGAGACTATGTTAACTTTACAGAGCTATAAATAACGGCCGACATGAGAG	73	418	9	61.64	540	1	0
N/A	GGGACACAATGGACGTATAAACAAAAGTACTGCAAAAATCAACTGTAGACTTGGTTAACGGCCGACATGAGAG	73	418	9	61.64	505	1	0
N/A	GGGACACAATGGACGATTAGATATACACTGTGAGAGATATAACGGCCGACATGAGAG	57	418	9	61.64	526	1	0
N/A	GGGACACAATGGACGACTGATATTATAGGGATGAGACAATATCTCATTGAAGGGATAACGGCCGACATGAGAG	73	418	9	61.64	522	1	0
N/A	GGGACACAATGGACGAAGCAAATGTTGACCAAGATTACAAGCGAAACATGAGCCTAACGGCCGACATGAGAG	72	418	9	61.64	428	1	0
N/A	GGGACACAATGGACGTTGTTAGACTTTGTAGTTAGCACACCAGCTACTATGCATCTAACGGCCGACATGAGAG	73	418	9	61.64	406	1	0
N/A	GGGACACAATGGACGGAAGATAAATAATTCATACAAGAAACCACTGTTTGGGCCTTAACGGCCGACATGAGAG	73	418	9	61.64	539	1	0
N/A	GGGACACAATGGACGGTACAAATGTGATTTAATAGATCTTTGCTAACGGCCGACATGAGAG	61	418	9	61.64	409	1	0
N/A	GGGACACAATGGACGTGAGCATACAACAAATTCAGCAACTTCGAGTGCGTAAGCTAACGGCCGACATGAGAG	72	418	9	61.64	440	1	0
N/A	GGGACACAATGGACGCGCTTGTACCAGGAATTAATGGATAGTCTTAGGTAACGGCCGACATGAGAG	66	418	9	61.64	410	1	0
N/A	GGGACACAATGGACGAATGTTACTTTATTTCCCAGTGAGTTGGTCGATAGGATGCTAACGGCCGACATGAGAG	73	418	9	61.64	418	1	0
N/A	GGGACACAATGGACGGTATCATAATGGAAAGACTCATTTATATGGAGGAATTCAGTAACGGCCGACATGAGAG	73	418	9	61.64	499	1	0
N/A	GGGACACAATGGACGTAATGAATTATCCATCCACTTTTAATGAAGGAGCGGAAATAACGGCCGACATGAGAG	72	418	9	61.64	481	1	0
N/A	GGGACACAATGGACGGTTCTTTATCGATAACGGCCGACATGAGAG	45	418	9	61.64	33	2	6
N/A	GGGACACAATGGACGTAAATCTATAGTTAACACAAGACGGTCATTTATATTTGAATAACGGCCGACATGAGAG	73	418	9	61.64	548	1	0
N/A	GGGACACAATGGACGCTGTGAAAATTATCTAACGGCCGACATGAGAG	47	418	9	61.64	391	2	6
N/A	GGGACACAATGGACGTAAACGACATTTTAATGACGAATGTTAACGGCCGACATGAGAG	58	418	9	61.64	417	1	0
N/A	GGGACACAATGGACGGAATTACAAACAAATTGAAGCTCTATAACTTGAGATGGCATAACGGCCGACATGAGAG	73	418	9	61.64	501	1	0
N/A	GGGACACAATGGACGCGTTAGAATATATTTACTAAACTAACGGCCGACATGAGAG	55	418	9	61.64	485	1	0
N/A	GGGACACAATGGACGTACCAATATAGTTTAAGTGGGAATGGAATATATTCCCTGGTAACGGCCGACATGAGAG	73	418	9	61.64	546	1	0
N/A	GGGACACAATGGACGTAATATCAACTGAAGGTCTAACGGCCGACATGAGAG	51	418	9	61.64	427	1	0
N/A	GGGACACAATGGACGCAAAAGCAAAACTAAGATGTAATGATTCTAAGTAAGTGACTAACGGCCGACATGAGAG	73	418	9	61.64	527	1	0
N/A	GGGACACAATGGACGTATAGACTAAGATTATTAGTCAACAAGTAAGTAGGCTTAATAACGGCCGACATGAGAG	73	418	9	61.64	492	1	0
N/A	GGGACACAATGGACGTGAAAGGTTGTTGAAGATTAGCACAGATAAGTTGATCTAACGGCCGACATGAGAG	70	418	9	61.64	542	1	0
N/A	GGGACACAATGGACGGTAATAATTTAACGGCCGACATGAGAG	42	418	9	61.64	5	5	6
N/A	GGGACACAATGGACGCCAAAACACTTTAACTCAGAAAATAAAAGATTTTCACTATAACGGCCGACATGAGAG	72	418	9	61.64	462	1	0
N/A	GGGACACAATGGACGTCATGATTCGGAGTATAATAATACAGCAACTTGGATAAGCTAACGGCCGACATGAGAG	73	418	9	61.64	510	1	0

N/A	GGGACACAATGGACGTATCGGATTTAATAGTACCAATATAGGAATGACGGCACTTAACGGCCGACATGAGAG	72	418	9	61.64	509	1	0
N/A	GGGACACAATGGACGTGAGTTCGGAGAGTTAAAGTCAAATGTATGGCAGTATGATAACGGCCGACATGAGAG	72	418	9	61.64	426	1	0
N/A	GGGACACAATGGACGTCAGATCAATTCACCGCAATGTAAAATTGAGGAAGGAGACTAACGGCCGACATGAGAG	73	418	9	61.64	512	1	0
N/A	GGGACACAATGGACGGATGAAGAGATATTTTATTTTAACGGCCGACATGAGAG	53	418	9	61.64	416	1	0
N/A	GGGACACAATGGACGGATTGGGATCTAGAAACACTGGATAACGGCCGACATGAGAG	56	418	9	61.64	534	1	0
N/A	GGGACACAATGGACGCGAACGAAGAAATTCAAAGTTACGGAATACAATTCGATTAACGGCCGACATGAGAG	71	418	9	61.64	541	1	0
N/A	GGGACACAATGGACGTAATACTATGATAAATAACGAATGGGGCCAATATGGATGATAACGGCCGACATGAGAG	73	418	9	61.64	483	1	0
N/A	GGGACACAATGGACGGAAGTTATGGTATTTGATGCCTGGAGAAGGGAAAGAATAGTAACGGCCGACATGAGAG	73	418	9	61.64	476	1	0
N/A	GGGACACAATGGACGGTGAGATATGCACTATGAAATTAAAACATTTATTGGTACATAACGGCCGACATGAGAG	73	418	9	61.64	424	1	0
N/A	GGGACACAATGGACGGGATTTAACTCGTAGACATAAAGGATGTGCGGTGCGTATGTAACGGCCGACATGAGAG	73	418	9	61.64	447	1	0
N/A	GGGACACAATGGACGGCAAAACCCTTATTAATACTACATAACGGCCGACATGAGAG	56	418	9	61.64	458	1	0
N/A	GGGACACAATGGACGTTATACGATCAAAACAGTACAAGTTAGGAATGATATTCCGATAACGGCCGACATGAGAG	74	418	9	61.64	435	1	0
N/A	GGGACACAATGGACGATACCTTCATTGTCAACTCGATAGTAGGACCGTTTTTAACTAACGGCCGACATGAGAG	74	418	9	61.64	467	1	0
N/A	GGGACACAATGGACGCTTACTTAGTAATGAGAAGAAATGTTGTTCACTCGCGGCATAACGGCCGACATGAGAG	73	418	9	61.64	432	1	0
N/A	GGGACACAATGGACGGCAATGTTTAATAATGGTTAACGGCCGACATGAGAG	51	418	9	61.64	497	1	0
N/A	GGGACACAATGGACGTAACACATTTGAGATGATTACAGATTGGGAACCGTCGGAGTAACGGCCGACATGAGAG	73	418	9	61.64	550	1	0
N/A	GGGACACAATGGACGGTTGAAACTAGGTACAATAAATGTAGTAACGGCCGACATGAGAG	59	418	9	61.64	429	1	0
N/A	GGGACACAATGGACGAATCTATATAAATGAACTCTGAGGAAGAGACCTGTAAAGGTAACGGCCGACATGAGAG	73	418	9	61.64	484	1	0
N/A	GGGACACAATGGACGCAAAATGACTTATCAATTTATCCTAATCAATGGGCTGTGCTAACGGCCGACATGAGAG	73	418	9	61.64	532	1	0
N/A	GGGACACAATGGACGGTCAGTAACGTGATATTAAGCAATTAACTGGTTAACGGCCGACATGAGAG	65	418	9	61.64	457	1	0
N/A	GGGACACAATGGACGGATCGAACAACTTTTCGGCTAGATATTCTATAGCCTGTAGTAACGGCCGACATGAGAG	73	418	9	61.64	469	1	0
N/A	GGGACACAATGGACGTTTGACACGAACTTCGAGCAGAGACTAGATAGTAGATTCATAACGGCCGACATGAGAG	73	418	9	61.64	506	1	0
N/A	GGGACACAATGGACGAATAATGGTATTAGAATTTTATGTGAGGCTTGTTTTAAGATAACGGCCGACATGAGAG	73	418	9	61.64	411	1	0
N/A	GGGACACAATGGACGATTAATAAAAGAATCCGAGATTAAGCAAATAAAGAGGCTGTAACGGCCGACATGAGAG	73	418	9	61.64	439	1	0
N/A	GGGACACAATGGACGTGAAACTAGTAAATAGCCTAGCGGTGTATTACATGGGCCGTAACGGCCGACATGAGAG	73	418	9	61.64	538	1	0
N/A	GGGACACAATGGACGCCTGAGCTATTCTGGATAAACTTATACATACCAGCAAAAGTTAACGGCCGACATGAGAG	74	418	9	61.64	445	1	0
N/A	GGGACACAATGGACGTGACCAGAATAGACAATTAATTATATTGGCTGATATAACGGCCGACATGAGAG	68	418	9	61.64	437	1	0
N/A	GGGACACAATGGACGAAGATTACATATATCCGGTCACCTTGGTCGTAGTAATTGACTAACGGCCGACATGAGAG	74	418	9	61.64	442	1	0
N/A	GGGACACAATGGACGCAATATGATAGAACGGCAAAGACTTTAACTGACGAGCTTCTAACGGCCGACATGAGAG	73	418	9	61.64	461	1	0
N/A	GGGACACAATGGACGCTAAATTATAACGTACTGAGTCGTCTGGGCTGTTTATCAATAACGGCCGACATGAGAG	73	418	9	61.64	419	1	0
N/A	GGGACACAATGGACGCATGTAGACAATATTGGATTAAATTGATACCGCATTAGGCTAACGGCCGACATGAGAG	73	418	9	61.64	434	1	0
N/A	GGGACACAATGGACGTAAAGGCGAAGAAGGCAAGTATTGACTATACTAGCTAGACTAACGGCCGACATGAGAG	73	418	9	61.64	518	1	0
N/A	GGGACACAATGGACGGGCGGTCTATATGAGTAAGAGCACACATATTTTACAACAATAACGGCCGACATGAGAG	73	418	9	61.64	459	1	0
N/A	GGGACACAATGGACGTTTATATCTAAGCAAGAAATAAGTGAACTGATGAGGCATATAACGGCCGACATGAGAG	73	418	9	61.64	496	1	0
N/A	GGGACACAATGGACGACGGGCAATAAAACGGAATTCAGTATGTAAATCCTAAGGCTAACGGCCGACATGAGAG	73	418	9	61.64	524	1	0
N/A	GGGACACAATGGACGGACTTACGGGACTTTTCATATTACAAAGAAGAGCTACGCATAACGGCCGACATGAGAG	73	418	9	61.64	413	1	0
N/A	GGGACACAATGGACGGTAATCATAATGGAAAGCTAGTATCCGAGCATTTAGCGGATAACGGCCGACATGAGAG	73	418	9	61.64	454	1	0
N/A	GGGACACAATGGACGGTAGGTTACATATCAAGGATTAAGCATAAGTTTCCTCACCTAACGGCCGACATGAGAG	73	418	9	61.64	530	1	0
N/A	GGGACACAATGGACGAGTGGTACTGTTTGGATTTGTTAGGTACAAATAGATTACTTAACGGCCGACATGAGAG	73	418	9	61.64	423	1	0
N/A	GGGACACAATGGACGCTGAACGGGGTTTTATTCGAATTATAACGATGATCTTGGATAACGGCCGACATGAGAG	73	418	9	61.64	422	1	0
N/A	GGGACACAATGGACGGTACATAAATGCGAGACGGATATTAACGAGTCTTTCAATTAACGGCCGACATGAGAG	72	418	9	61.64	468	1	0
N/A	GGGACACAATGGACGGAGTGAAACCGTGAGGCAGAGCTAGGAATATTTAAACTAGTAACGGCCGACATGAGAG	73	418	9	61.64	452	1	0
N/A	GGGACACAATGGACGTTAATCAGAAGATGTGAATCTACTGTACGCCTTTAGGATTAACGGCCGACATGAGAG	72	418	9	61.64	514	1	0
N/A	GGGACACAATGGACGCACGACTACAGTACTTATAACTCATTGAGAGCTAGAGTCTTAACGGCCGACATGAGAG	73	418	9	61.64	438	1	0
N/A	GGGACACAATGGACGTTACTCTTAGTTACATAAGTACTAACACGATGATCGGTAACGGCCGACATGAGAG	70	418	9	61.64	533	1	0
N/A	GGGACACAATGGACGCAAAGTTAAAACACATAATAACGGCCGACATGAGAG	51	418	9	61.64	448	1	0
N/A	GGGACACAATGGACGGAAAAATGTACTAATTACCGAAACCAACTCAAATGATGGCTTAACGGCCGACATGAGAG	74	418	9	61.64	486	1	0
N/A	GGGACACAATGGACGATGTAAGAAATTCAATCCTTATCCTGATGCGGCAATATACTAACGGCCGACATGAGAG	73	418	9	61.64	425	1	0
N/A	GGGACACAATGGACGTTTGTTATATTTGAAGAGGTCGCTAACCCTATGGAGGCTTTAACGGCCGACATGAGAG	73	418	9	61.64	431	1	0
N/A	GGGACACAATGGACGGTACTGCGCTACATATGAAGGAAAGACTAACATATAAGGATAACGGCCGACATGAGAG	73	418	9	61.64	521	1	0
N/A	GGGACACAATGGACGCTATAAACATTTAATGACATATTACAGTTGATTAAGATGATAACGGCCGACATGAGAG	73	418	9	61.64	515	1	0
N/A	GGGACACAATGGACGGGTTTTAGAAGACAGCAATAAAATTATCAGCTAAGTACTCTAACGGCCGACATGAGAG	73	418	9	61.64	414	1	0
N/A	GGGACACAATGGACGAGTAACGTGTGAACAAATAGTGGGCTTTGATTAATTAACATAACGGCCGACATGAGAG	73	418	9	61.64	513	1	0
N/A	GGGACACAATGGACGTTAAAAGCCGATGAACAACAGCAATTATTCTAGGGTTGGTTAACGGCCGACATGAGAG	73	418	9	61.64	547	1	0
N/A	GGGACACAATGGACGGTAATAGTTTGTTGATAACGGTGTATGACAAGAAATCATATAACGGCCGACATGAGAG	73	418	9	61.64	535	1	0
N/A	GGGACACAATGGACGTATAAAAGGAATATGGGCTATTGCACCACACAGTTGGCGATAACGGCCGACATGAGAG	73	418	9	61.64	412	1	0
N/A	GGGACACAATGGACGTAATAAATAATTGACATCGAATCTCAAGGAACGAGTGCCATAACGGCCGACATGAGAG	73	418	9	61.64	529	1	0
N/A	GGGACACAATGGACGTTTTATTTGATAACGGCCGACATGAGAG	43	418	9	61.64	221	2	5
N/A	GGGACACAATGGACGCAACGTATTTAGCAGGAGATAATTATAATATTTTCAAGATAACGGCCGACATGAGAG	72	418	9	61.64	478	1	0
N/A	GGGACACAATGGACGGTATGCGGGGAATGATGTCAATTATTAAACATCGAAGACCTAACGGCCGACATGAGAG	73	418	9	61.64	456	1	0

N/A	GGGACACAATGGACGAAGGAATTATTCATACCATCATAAGATGCCCTAAAACGGCTAACGGCCGACATGAGAG	73	418	9	61.64	488	1	0
N/A	GGGACACAATGGACGTAATAATCGATTTTGTATATAATACTCAATATGGAAGCTAACGGCCGACATGAGAG	71	418	9	61.64	519	1	0
N/A	GGGACACAATGGACGTAACGAAAATGGATAAATAATAAATGTTAACCTGATTAGTTAACGGCCGACATGAGAG	73	418	9	61.64	543	1	0
N/A	GGGACACAATGGACGGACTGAATAAAACAATTGTACACTGGCTTAATCGCCATGGTAACGGCCGACATGAGAG	73	418	9	61.64	482	1	0
N/A	GGGACACAATGGACGGATAAAGAATCAAATATAACGGCCGACATGAGAG	49	418	9	61.64	495	1	0
N/A	GGGACACAATGGACGCAAAGACGCCTTCACATCAGTTTGATAATGAACGACAGTCTAACGGCCGACATGAGAG	73	568	8	54.79	657	1	0
N/A	GGGACACAATGGACGCTTGTGAGGTATTCTCAACACTTAAATACATTGAGATGCCTAACGGCCGACATGAGAG	73	568	8	54.79	720	1	0
N/A	GGGACACAATGGACGCTACAACAATAAGAAGAACGAGAGTTCCGCACGGTAGTTTAACGGCCGACATGAGAG	72	568	8	54.79	591	1	0
N/A	GGGACACAATGGACGCCTATCAGAAACTGAGAGTTATATGAATATCTCTTAGTAGTAACGGCCGACATGAGAG	73	568	8	54.79	644	1	0
N/A	GGGACACAATGGACGGAAAAGCAATTATGAGGGCATATGGAGCTACTTTATAGGGTAACGGCCGACATGAGAG	73	568	8	54.79	655	1	0
N/A	GGGACACAATGGACGAAGAGTGATGCCTTATGAGAGATATTCTAGTATGGGAGACTAACGGCCGACATGAGAG	73	568	8	54.79	625	1	0
N/A	GGGACACAATGGACGGATCGAACA	24	568	8	54.79	697	1	0
N/A	GGGACACAATGGACGAGACAATTTACTGTAACGTACACTTTAACAATGAAGGGCTTAACGGCCGACATGAGAG	73	568	8	54.79	707	1	0
N/A	GGAGCACAATGGACGGACCGACAAGATAAAGGAACGTATGATATTCCTATTGTAATAACGGCCGACATGAGAG	73	568	8	54.79	646	1	0
N/A	GGGACACAATGGACGTTACCTAAATGTATTAGGCATGAGTATCGAAATTATTAGATAACGGCCGACATGAGAG	73	568	8	54.79	596	1	0
N/A	GGGACACAATGGACGAACTTGTGACTAGAAAATTTAGGTCGAGATTTAATTAATATAACGGCCGACATGAGAG	73	568	8	54.79	653	1	0
N/A	GGGACACAATGGACGGACATAGTATATGTTAACCATAAACTAGTCGGAATAACGGCCGACATGAGAG	67	568	8	54.79	689	1	0
N/A	GGGACACAATGGACGATAATTCTACTATTATACAATCTCGCGGCTTTAGTAAGTTTAACGGCCGACATGAGAG	73	568	8	54.79	562	1	0
N/A	GGGACACAATGGACGGAAGTGGTTGAATGAATATAAACCAATGCTATTAATCGACTAACGGCCGACATGAGAG	73	568	8	54.79	661	1	0
N/A	GGGACACAATGGACGATCAAGTGGCTTGTTATAGTGAGGAATAGTTTATCGCAAGTAACGGCCGACATGAGAG	73	568	8	54.79	615	1	0
N/A	GGGACACAATGGACGTAGTTCAGACGGGTGCGATGGATCTGTACTGTTTGTAACATAACGGCCGACATGAGAG	73	568	8	54.79	609	1	0
N/A	GGGACACAATGGACGGTAATACGTTATGAGAGTTTGTTAGTTTCAACACTACGAGTAACGGCCGACATGAGAG	73	568	8	54.79	575	1	0
N/A	GGGACACAATGGACGCCATCTAAGAACAGAGAAATCTAAATCACTGATCAAGGTATAACGGCCGACATGAGAG	73	568	8	54.79	652	1	0
N/A	GGGACACAATGGACGCTGGATTTAGTGACGATTAGTTAAATGACTTACTAACTATTAACGGCCGACATGAGAG	73	568	8	54.79	602	1	0
N/A	GGGACACAATGGACGAATTAAATACTAAGAAATCCGCAATATCGCGTGATCGGGCTAACGGCCGACATGAGAG	73	568	8	54.79	570	1	0
N/A	GGGACACAATGGACGGATCTTTAGAAGTGACGATATCTCGTTACGTAACGGCCGACATGAGAG	63	568	8	54.79	588	1	0
N/A	GGGACACAATGGACAGTGAAGCATTTACTGAAGTACTAGTTTAACTGACGCGTACTAACGGCCGACATGAGAG	73	568	8	54.79	668	1	0
N/A	GGGACACAATGGACGATGACTTGATTGTAATAGAAGCTGTGAACTCTTTTAGGGTTAACGGCCGACATGAGAG	73	568	8	54.79	710	1	0
N/A	GGGACACAATGGACGTGCCGATCGAGATACATATAGGAGACTCAATTTCGAATCGTAACGGCCGACATGAGAG	73	568	8	54.79	631	1	0
N/A	GGGACACAATGGACGTATCAAAGTGTATGGAGCTAATATCAAACTATTACACGTAACGGCCGACATGAGAG	71	568	8	54.79	714	1	0
N/A	GGGACACAATGGACGAAATGACAATGATGGTAACCTGTCGGAGATAAACGATTTGTAACGGCCGACATGAGAG	73	568	8	54.79	634	1	0
N/A	GGGACACAATGGACGTGAACGTATATGAGATTAATTAAATCTATGTAACGGCCGACATGAGAG	63	568	8	54.79	620	1	0
N/A	GGGACACAATGGACGTAACGAAGATCAAAACAGACGAAAATGATTGAATTGAGCTTAACGGCCGACATGAGAG	73	568	8	54.79	675	1	0
N/A	GGGACACAATGGACGAATAGTCGGAATTATAAATGTGTTAACTAGCAGGTTGGGCTAACGGCCGACATGAGAG	73	568	8	54.79	673	1	0
N/A	GGGACACAATGGACGTATGTTTTAAATATGAGCTACTGAGTCGAGTCAGCCAACGTAACGGCCGACATGAGAG	73	568	8	54.79	554	1	0
N/A	GGGACACAATGGACGATCAAAGGCCTTGTTAATCTAAGTGTAGTAAACAATTTGATAACGGCCGACATGAGAG	73	568	8	54.79	658	1	0
N/A	GGGACACAATGGACGTAATTAGTATCTTGGAAAGAGGTCTCAGAAGTTCGGAACTTAACGGCCGACATGAGAG	73	568	8	54.79	660	1	0
N/A	GGGACACAATGGACGAGATGTAGCTATGGTGTATAAGTTATAAATTATACTCGTGCTAACGGCCGACATGAGAG	74	568	8	54.79	610	1	0
N/A	GGGACACAATGGACGTGCCATTATTATTTAAGATTATAACGGCCGACATGAGAG	54	568	8	54.79	351	2	6
N/A	GGGACACAATGGACGGACCGTGGTTTTAACGGCCGACATGAGAG	44	568	8	54.79	38	3	4
N/A	GGGACACAATGGACGTTCAAATGCGTTAAAGGTCTAAAGTTAACTATTTGGTAACTAACGGCCGACATGAGAG	73	568	8	54.79	553	1	0
N/A	GGGACACAATGGACGCCTATAAATTAACAATGTGGTAAAGACGGATCGATATAACTAACGGCCGACATGAGAG	73	568	8	54.79	556	1	0
N/A	GGGACACAATGGACGGTTATATACAAGTAATGATATCCTATGAACGATGGGAGGCTAACGGCCGACATGAGAG	73	568	8	54.79	651	1	0
N/A	GGGACACAATGGACGTGAGAACAAATGACTTCCAATCTCTGTATATAGGGCATAACGGCCGACATGAGAG	70	568	8	54.79	728	1	0
N/A	GGGACACAATGGACGGCAAAGTAGTAAATTTAGTCATCTCAATAATGAGAAGCAGTAACGGCCGACATGAGAG	73	568	8	54.79	642	1	0
N/A	GGGACACAATGGACGCGTATTTTAATACTAACTTGAATGGGCAGGTGAGTTGTTATAACGGCCGACATGAGAG	73	568	8	54.79	719	1	0
N/A	GGGACACAATGGACGGGATTTATACATCAAATGTTAATTTGTCATCCCTGCGGCGTAACGGCCGACATGAGAG	73	568	8	54.79	727	1	0
N/A	GGGACACAATGGACGATGTATTTAACGAATGTGTTTGCACACAAATGTTTGGTCATAACGGCCGACATGAGAG	73	568	8	54.79	565	1	0
N/A	GGGACACAATGGACGGTAACAGGACACGGATGGTTATTATAAACAATACGAGTAGTAACGGCCGACATGAGAG	73	568	8	54.79	703	1	0
N/A	GGGACACAATGGACGAAAGGAAGTAATGATAGTTCTGCGTGAATGCATGAGGGCTTAACGGCCGACATGAGAG	73	568	8	54.79	705	1	0
N/A	GGGACACAATGGACGTTTAGAAACATGATGCTTTTCATAGTTGACAATATTATACTAACGGCCGACATGAGAG	73	568	8	54.79	698	1	0
N/A	GGGACACAATGGACGTAATGGGAGCTTACTGTTTTATACATTCTATATAAGACGTTAACGGCCGACATGAGAG	73	568	8	54.79	611	1	0
N/A	GGGACACAATGGACGTACTGTTTAAGTAAATAAGATCGATGATGGTCTCGAGGCCTAACGGCCGACATGAGAG	73	568	8	54.79	557	1	0
N/A	GGGACACAATGGACGGAGAGTGCTTCTAGTAAACTAACATAATAGGTTATAGGGTAACGGCCGACATGAGAG	72	568	8	54.79	731	1	0
N/A	GGGACACAATGGACGACGGAAATTTACTCTTAACTGAATATATTATTAAAGTAATTAACGGCCGACATGAGAG	73	568	8	54.79	552	1	0
N/A	GGGACACAATGGACGGTAACATTAATAAAAGAGAGCCATTAGAATATGGCTGTTGTAACGGCCGACATGAGAG	73	568	8	54.79	641	1	0
N/A	GGGACACAATGGACGAAGTTATTAAAACAGGATGTTCGCTGAATTGTCTTGGGCGTAACGGCCGACATGAGAG	73	568	8	54.79	687	1	0
N/A	GGGACACAATGGACGATATAGGAGTTTAAATGATATTAGTGTTCGTATGGGGTCATAACGGCCGACATGAGAG	73	568	8	54.79	638	1	0
N/A	GGGACACAATGGACGTTTGAATAGTACTGACGGCTTACTGATAGATCATAAAAGTAACGGCCGACATGAGAG	72	568	8	54.79	580	1	0
N/A	GGGACACAATGGACGCCGAACAAAACTTATTAACAAGTAAACACTCGTAAATAGCTAACGGCCGACATGAGAG	73	568	8	54.79	607	1	0

N/A	GGGACACAATGGACGTTATTCGAGTATTCTGATCTTATTTTAGCGAGAGCAGCGATAACGGCCGACATGAGAG	73	568	8	54.79	699	1	0
N/A	GGGACACAATGGACGTGGGGCAGTTTTGAAGTTTTTGAACATACAGTTCAGAGCCTAACGGCCGACATGAGAG	73	568	8	54.79	628	1	0
N/A	GGGACACAATGGACGTTTAGTAAAAATACAGGGTTTACCGCTAGTCGTGTAGGCGTAACGGCCGACATGAGAG	73	568	8	54.79	583	1	0
N/A	GGGACACAATGGACGGTGGTTATAACAATTATATGATAACGGCCGACATGAGAG	54	568	8	54.79	592	1	0
N/A	GGGACACAATGGACGTTTATGTTGACCAAATCCGAAAAGGAGTGGACTGTAATGATAACGGCCGACATGAGAG	73	568	8	54.79	587	1	0
N/A	GGGACACAATGGACGTCATTAGAATTAAACTAGAAGACACCATGCTGGATGGCTATAACGGCCGACATGAGAG	73	568	8	54.79	616	1	0
N/A	GGGACACAATGGACGGAATTTTTGACATCACTCTTAAGGATGCGGTAACGGCCGACATGAGAG	63	568	8	54.79	630	1	0
N/A	GGGACACAATGGACGATTTTTTCCTAAGTTAATGATACTCCTATCGAGGTACAAAATAACGGCCGACCTGAGAG	74	568	8	54.79	650	1	0
N/A	GGGACACAATGGACGGTCAAGTACCAGATAGGAAAGCAAAACTTTTGGATCACAGTAACGGCCGACATGAGAG	73	568	8	54.79	618	1	0
N/A	GGGACACAATGGACGAAAGAACAAGAACAGATTTACTTAGTACACAGTCATCATATAACGGCCGACATGAGAG	73	568	8	54.79	639	1	0
N/A	GGGACACAATGGACGAAACATATATAATACAGTTCCAACGGCCTATGGCATGACCTAACGGCCGACATGAGAG	73	568	8	54.79	577	1	0
N/A	GGGACACAATGGACGTAAGATGATGTATTCTCAGGTTCTTAGTGAGATGGGCACATAACGGCCGACATGAGAG	73	568	8	54.79	560	1	0
N/A	GGGACACAATGGACGGTATTGATTGAACGGACGAAGTATGATATTAAGTAATTCGTAACGGCCGACATGAGAG	73	568	8	54.79	571	1	0
N/A	GGGACACAATGGACGTTATTTCGACATCGATACTATTTGTCAGCACTTGTTGGGCTAACGGCCGACATGAGAG	73	568	8	54.79	621	1	0
N/A	GGGACACAATGGACGGTATGGAAATATGTTCATAACCAATGTCTGGCATAGTTTATAACGGCCGACATGAGAG	73	568	8	54.79	695	1	0
N/A	GGGACACAATGGACGGTCGGAATGTAAAGAAAGAATGTTTATAAGTTCTGCAGATTAACGGCCGACATGAGAG	73	568	8	54.79	678	1	0
N/A	GGGACACAATGGACGTTATACTAAGACTACCAATATTCTAAGTGGTAATACGCGTAACGGCCGACATGAGAG	72	568	8	54.79	648	1	0
N/A	GGGACACAATGGACGTTTAAAACCACGACTTAGGAAATAACTTGAAGGTTGGAGAATAACGGCCGACATGAGAG	74	568	8	54.79	578	1	0
N/A	GGGACACAATGGACGATATTGCTGACCTTTGAGAAAATAACGGCCGACATGAGAG	55	568	8	54.79	696	1	0
N/A	GGGACACAATGGACGTTACTTACTATAACTATGACGAAGAAATTCTACATATAGTAACGGCCGACATGAGAG	72	568	8	54.79	593	1	0
N/A	GGGACACAATGGACGTATGGAATAGGATATAGAACGTTAATTCTACTAAACTCTATAACGGCCGACATGAGAG	73	568	8	54.79	701	1	0
N/A	GGGACACAATGGACGAATAGAAAATTGATATATAATGACCGCTACATTTTAGGCTTAACGGCCGACATGAGAG	73	568	8	54.79	680	1	0
N/A	GGGACACAATGGACGAGTACGAACATCACCACACTATGTATAATGTTGCGAATGATAACGGCCGACATGAGAG	73	568	8	54.79	645	1	0
N/A	GGGACACAATGGACGAGTAGGTCAGTTTATAATATTACAATAGACTTTCACAACCTAACGGCCGACATGAGAG	73	568	8	54.79	605	1	0
N/A	GGGACACAATGGACGTATTCCACTTACAAACCTTGCTCGTAATAGATAACGCGCCTAACGGCCGACATGAGAG	73	568	8	54.79	666	1	0
N/A	GGGACACAATGGACGAACTAAAGCTTTTGTGGTAATACTAGAAGGCACGGATAAATAACGGCCGACATGAGAG	73	568	8	54.79	690	1	0
N/A	GGGACACAATGGACGATGTAGGTCGTATGCTTAATATTACAAATGTTAATGACTGTAACGGCCGACATGAGAG	73	568	8	54.79	664	1	0
N/A	GGGACACAATGGACGCCGGTTGAACTTAACGGCCGACATGAGAG	44	568	8	54.79	601	1	0
N/A	GGGACACAATGGACGTAATTAAAGTTGATGAATGAGTGACGGAAGTTATTAAAGATAACGGCCGACATGAGAG	73	568	8	54.79	694	1	0
N/A	GGGACACAATGGACGCAATAGAACAGTCGCATATTGATAATCCTATCGTTTCGGTTAACGGCCGACATGAGAG	73	568	8	54.79	576	1	0
N/A	GGGACACAATGGACGCATATCGGTTAATATGGTAGTCGACATAACACACTGGCTATAACGGCCGACATGAGAG	73	568	8	54.79	590	1	0
N/A	GGGACACAATGGACGATAGTCTTTTGTAGGAAATATAGAGGCTGATTTGAATTTGTAACGGCCGACATGAGAG	73	568	8	54.79	700	1	0
N/A	GGGACACAATGGACGAGGCCCAAAGTTAACAATAACGCGAGAGCTATATGGATAATAACGGCCGACATGAGAG	73	568	8	54.79	559	1	0
N/A	GGGACACAATGGACGGATGATGTTATTAGGGGCCAAAGAAGGTTTAAAATAACTGTAACGGCCGACATGAGAG	73	568	8	54.79	721	1	0
N/A	GGGACACAATGGACGGTCGCATACATACTGAGTTTGATTTGGATTCAAAGGGAGGTAACGGCCGACATGAGAG	73	568	8	54.79	730	1	0
N/A	GGGACACAATGGACGTTGTCATTTAAAGAATACGCTCTTGGGCGTACTGAGTAGGTAACGGCCGACATGAGAG	73	568	8	54.79	564	1	0
N/A	GGGACACAATGGACGAATACTGATAATTTATTAACGGCCGACATGAGAG	49	568	8	54.79	287	2	6
N/A	GGACACAATGGACGGGCGGATAACTACATCGCATTAAATTATGTAACGAATTGGTAACGGCCGACATGAGAG	72	568	8	54.79	563	1	0
N/A	GGGACACAATGGACGAGATACGAATGATCGGATTTCCGTAGGGTAGGATTTTACCTAACGGCCGACATGAGAG	73	568	8	54.79	627	1	0
N/A	GGGACACAATGGACGGAACCATGATCTAGATGAAGGCGACTTATACTACATAGCATAACGGCCGACATGAGAG	73	568	8	54.79	725	1	0
N/A	GGGACACAATGGACGGATTACAACTGGAGGTGTTAACATTTTAGGAAACTGATTTTAACGGCCGACATGAGAG	73	568	8	54.79	594	1	0
N/A	GGGACACAATGGACGCCGAAATTACTTAAACAGTCGATTACAGAATCATACTGGCTAACGGCCGACATGAGAG	73	568	8	54.79	600	1	0
N/A	GGGACACAATGGACGAGTAGAAATTAATAACCTATTATATTGGGACCATAAGGCCTAACGGCCGACATGAGAG	73	568	8	54.79	667	1	0
N/A	GGGACACAATGGACGAATATTAATTTGATGGACCGACATGGGTAGTTTTAGAATAACGGCCGACATGAGAG	71	568	8	54.79	686	1	0
N/A	GGGACACAATGGACGCTTACTGATTAGGTCTGATTAAATTTATTGGGCGTAATACTAACGGCCGACATGAGAG	73	568	8	54.79	716	1	0
N/A	GGGACACAATGGACGTCTAAGCCTATAAAAAAACGGCCTAGATACCTTCTAGTTCTAACGGCCGACATGAGAG	73	568	8	54.79	706	1	0
N/A	GGGACACAATGGACGATTACTAATTTATGAGTGTATATAATTACAACACTTAGTTTAACGGCCGACATGAGAG	73	568	8	54.79	608	1	0
N/A	GGGACACAATGGACGATGCACACACGCAAGTGGATCTAATAGGATCTAATATGGGTAACGGCCGACATGAGAG	73	568	8	54.79	711	1	0
N/A	GGGACACAATGGACGTAGAAGCTTTAAGGAGGCGTGAAAAATATTCGCACCTATGGTAACGGCCGACATGAGAG	74	568	8	54.79	624	1	0
N/A	GGGACACAATGGACGGAAACAAACTAACAATATCTGAAAGATTGTAATTTGAAGTTAACGGCCGACATGAGAG	73	568	8	54.79	717	1	0
N/A	GGGACACAATGGACGACATAGTAATTAAAGTTTATGTACCTAATCAGACGGGCGGTAACGGCCGACATGAGAG	73	568	8	54.79	558	1	0
N/A	GGGACACAATGGACGGCGATTCTATCTAGGAAATATCATTCTGGATCGGGAACGATAACGGCCGACATGAGAG	73	568	8	54.79	568	1	0
N/A	GGGACACAATGGACGCTTAAGTTATAAAAGGCGAGTTACTAAACGACTGCTTGCATAACGGCCGACATGAGAG	73	568	8	54.79	561	1	0
N/A	GGGACACAATGGACGCTAGGACTTTCCGATAGGATTAAGAATGTAAACTATTCGTAACGGCCGACATGAGAG	72	568	8	54.79	604	1	0
N/A	GGGACACAATGGACGTACCAATATTTAGGGTTGAACAATTGGTTCCTATAGGCTGTAACGGCCGACATGAGAG	73	568	8	54.79	649	1	0
N/A	GGGACACAATGGACGTTGTTATATAGTAAATAGGGTGAAACAATATTTGACACGATAACGGCCGACATGAGAG	73	568	8	54.79	635	1	0
N/A	GGGACACAATGGACGTTTGTTCGTTCTGAACCGGCTTAACGGCCGACATGAGAG	54	568	8	54.79	598	1	0
N/A	GGGACACAATGGACGGTCTAAATACACATCAGTGAGATATACACAAAGATTGTTATAACGGCCGTACATGAGAG	74	568	8	54.79	676	1	0
N/A	AGGGACACAATGGACGGACCAATTTGACGGAGTTAAAATTAATTAACACACGCATAGTAACGGCCGACATGAGAG	75	568	8	54.79	551	1	0
N/A	GGGACACAATGGACGTATATGCTTAAAATTTGATACCCTTAATAGTTGGACGATATAACGGCCGACATGAGAG	73	568	8	54.79	691	1	0

N/A	GGGACACAATGGACGTTTCAAAGGCTGATGCGAACTATTATTCAAGTTAGCGTAACGGCCGACATGAGAG	70	568	8	54.79	595	1	0
N/A	GGGACACAATGGACGAGCCAGCCAATCTACTATACTAGAGTAACGGCCGACATGAGAG	58	568	8	54.79	614	1	0
N/A	GGGACACAATGGACGTAAAATTATGTAAGGCAATTAGACACTGTATATAACGTGATAACGGCCGACATGAGAG	73	568	8	54.79	692	1	0
N/A	GGGACACAATGGACGTTAGGAAATTGTATAGGAATACAAAACTGAGGCACGTTTGATAACGGCCGACATGAGAG	74	568	8	54.79	726	1	0
N/A	GGGACACAATGGACGGTACAACAGGCATTAAATATGATCACCTTTATTTAGCACCTAACGGCCGACATGAGAG	73	568	8	54.79	671	1	0
N/A	GGGACACAATGGACGTCAAATCCAGAACTAATTAAATAGATGAGTCACACGGCATAACGGCCGACATGAGAG	72	568	8	54.79	722	1	0
N/A	GGGACACAATGGACGTTAAGTATTTCGACTTTGTACAAAACCTGTATGTTGGCAATAACGGCCGACATGAGAG	73	568	8	54.79	647	1	0
N/A	GGGACACAATGGACGTGAGTCGGACGTAAAGCGTTAATCTGTATTCCATAATGGCTAACGGCCGACATGAGAG	73	568	8	54.79	569	1	0
N/A	GGGACACAATGGACGGATGTTAATTAAATGAGACTGATGAGAAGAATCATATCAATAACGGCCGACATGAGAG	73	568	8	54.79	674	1	0
N/A	GGGACACAATGGACGTATAAATACCTATAGTGAACGCATTTCTGATGCGAACCGGTAACGGCCGACATGAGAG	73	568	8	54.79	597	1	0
N/A	GGGACACAATGGACGCTTATAACCCGATTTTCAGCATGCATAATGCTTCTCAGGTTAACGGCCGACATGAGAG	73	568	8	54.79	681	1	0
N/A	GGGACACAATGGACGGTACAAACAGGGTTATATTGACAAATAAGATTGACCTAGTAACGGCCGACATGAGAG	72	568	8	54.79	633	1	0
N/A	GGGACACAATGGACGCTTGTATTTAACGGCCGACATGAGAG	41	568	8	54.79	5	7	5
N/A	GGGACACAATGGACGAAGGTACGGACGTTAAGCTATTATGACTGATGAACGGATCTAACGGCCGACATGAGAG	73	568	8	54.79	643	1	0
N/A	GGGACACAATGGACGCCAAGTATTTATTTAAGTAGTATTTAGTAATGAGTACTTCTAACGGCCGACATGAGAG	73	568	8	54.79	589	1	0
N/A	GGGACACAATGGACGGCGATTTAATATTACTAACGGCCGACATGAGAG	48	568	8	54.79	235	2	6
N/A	GGGACACAATGGACGCACAATATATGGCAACCAATAGTAAAAATTTCCCGTAGGCCTAACGGCCGACATGAGAG	74	568	8	54.79	637	1	0
N/A	GGGACACAATGGACGACTATTTTGCGAATCATGTGAATTGCTGATGTTTATGGCTTAACGGCCGACATGAGAG	73	568	8	54.79	654	1	0
N/A	GGGACACAATGGACGATTTCATATTAACAAACACATTATACCTATGTTGGGCCAATAACGGCCGACATGAGAG	73	568	8	54.79	677	1	0
N/A	GGGACACAATGGACGCCGAGAATTATCTATTTAGGAATAACGGCCGACATGAGAG	55	568	8	54.79	659	1	0
N/A	GGGACACAATGGACGGTGAGTTAAAATGTTTACATATAACGGCCGACATGAGAG	54	568	8	54.79	712	1	0
N/A	GGGACACAATGGACGTATTGAACGTTTTGTTAAGAACCGAATAGTGTTTTATGGCTTAACGGCCGACATGAGAG	74	568	8	54.79	566	1	0
N/A	GGGACACAATGGACGCCACGGAGTTAAAGCGAATTTAAAAAATAACGTATATGACTAACGGCCGACATGAGAG	73	568	8	54.79	682	1	0
N/A	GGGACACAATGGACGTTTTCGAGAACTTCTCGCTGATTTGCGGTAGTACTTATTGTAACGGCCGACATGAGAG	73	568	8	54.79	622	1	0
N/A	GGGACACAATGGACGATTGTATTAATTATGTAGGTGATGTTATACTATATTCAGATAACGGCCGACATGAGAG	73	568	8	54.79	704	1	0
N/A	GGGACACAATGGACGGACCGGGACATGAAAATTAAGCAATTTAAGCAGTATGCACTAACGGCCGACATGAGAG	73	568	8	54.79	684	1	0
N/A	GGGACACAATGGACGGTTTGAGTTATGATATATATAACGGCCGACATGAGAG	52	568	8	54.79	636	1	0
N/A	GGGACACAATGGACGATAATAGTCGATTAAATATAGTTCTAGTGTAGACCAGGCTTAACGGCCGACATGAGAG	73	568	8	54.79	617	1	0
N/A	GGGACACAATGGACGTTATAGGCAAACTTTAGAGTGAAATAGCTAGAAGCAAGCGTAACGGCCGACATGAGAG	73	568	8	54.79	729	1	0
N/A	GGGACACAATGGACGTAAAGTAACTAATTTTATTAACGGCCGACATGAGAG	51	568	8	54.79	685	1	0
N/A	GGGACACAATGGACGTGCTGCTTTTAACGGCCGACATGAGAG	42	568	8	54.79	613	1	0
N/A	GGGACACAATGGACGCCAAATGCAGACGTAAACTAAAATGATGAAACTAGGGATGTAACGGCCGACATGAGAG	73	568	8	54.79	713	1	0
N/A	GGGACACAATGGACGTTTTTATACTATAATATATAACGGCCGACATGAGAG	51	568	8	54.79	619	1	0
N/A	GGGACACAATGGACGTGGAGGCCAAAGACTTGCGCATTATTTTAACTGTGTAGAGTAACGGCCGACATGAGAG	73	568	8	54.79	656	1	0
N/A	GGGACACAATGGACGAAGTTTTAGATCTACAAGAAGGACCAACTAGTAATGACGGTAACGGCCGACATGAGAG	73	568	8	54.79	670	1	0
N/A	GGGACACAATGGACGTATGTTACTACAATAAAGGTGCGATAGCTAATATCATTAGTAACGGCCGACATGAGAG	73	568	8	54.79	672	1	0
N/A	GGGACACAATGGACGATAAACTATATGGTTGTACCTATATGAAGGCGAGTTTAGATAACGGCCGACATGAGAG	73	568	8	54.79	581	1	0
N/A	GGGACACAATGGACGAAACTTATTACAATAGAAGTGTAAGTAAATGTTAGCCACTTAACGGCCGACATGAGAG	73	568	8	54.79	579	1	0
N/A	GGAACACAATGGACGGAGGTGATAATATTAAATGACTAACGGCCGACATGAGAG	54	568	8	54.79	603	1	0
N/A	GGGACACAATGGACGATATATATGCGGATTTTAGTTTGAATGTTACAGGTATTCATAACGGCCGACATGAGAG	73	568	8	54.79	723	1	0
N/A	GGGACACAATGGACGGATTTAAGCGAAGCAGACATATAGAAGTTGCGATGCAAAATAACGGCCGACATGAGAG	73	568	8	54.79	702	1	0
N/A	GGGACACAATGGACGTAATGAAAATTGTCGTATGAATGTAAGATAGGCAGTGAGGTAACGGCCGACATGAGAG	73	568	8	54.79	573	1	0
N/A	GGGACACAATGGACGCTATGACGGAGTAGACTGCATATTTTCAAATGCGAAATCGTAACGGCCGACATGAGAG	73	568	8	54.79	632	1	0
N/A	GGGACACAATGGACGGTGTTATCTATTACTCTGTTAAATAATAATGCAGGTACGGTAACGGCCGACATGAGAG	73	568	8	54.79	715	1	0
N/A	GGGACACAATGGACGAGCAATTATTACTAAGTATAACGGCCGACATGAGAG	51	568	8	54.79	245	2	6
N/A	GGGACACAATGGACGGTAACAGATAGTGAGTTCTGAAAGCATTGACTTTCTTGCTTAACGGCCGACATGAGAG	73	568	8	54.79	626	1	0
N/A	GGGACACAATGGACGGCAATTAATCATATGTTACCAAGAATCAAAATGGAGGATGTAACGGCCGACATGAGAG	73	568	8	54.79	688	1	0
N/A	GGGACACAATGGACGCTATAGTATATGGAGTTGAATCTATATTTAGACCCGTAGACTAACGGCCGACATGAGAG	74	568	8	54.79	683	1	0
N/A	GGGACATAATGGACGTATTTACACAATAAGATTTTCATCACGGGCGCTTAGCGAGTAACGGCCGACATGAGAG	73	568	8	54.79	679	1	0
N/A	GGGACACAATGGACGGTAAATTGTATGTAAATTACCAATAACGGCCGACATGAGAG	56	568	8	54.79	574	1	0
N/A	GGGACACAATGGACGGGCCAACCACCGAACGATATATTGAATGATTATTAGTTGATAACGGCCGACATGAGAG	73	568	8	54.79	584	1	0
N/A	GGGACACAATGGACGTCGGTAATGATGGCAAAAATTAATGAATACTTGTCAAAGCTAACGGCCGACATGAGAG	73	568	8	54.79	669	1	0
N/A	GGGACACAATGGACGCTAATAATAACAACCAACATTAACGGCCGACATGAGAG	53	568	8	54.79	724	1	0
N/A	GGGACACAATGGACGGAGTAAACGTTAAGCTGGAGTATTTATTGGATGCTGACGATAACGGCCGACATGAGAG	73	568	8	54.79	586	1	0
N/A	GGGACACAATGGACGTTTAACCTAACGGCCGACATGAGAG	40	568	8	54.79	5	6	5
N/A	GGGACACAATGGACGCGCTGTGAGGTTCTATTATATGATAATACATATAAGTACGTAACGGCCGACATGAGAG	73	568	8	54.79	709	1	0
N/A	GGGACACAATGGACGAACATTTTATAATCTGTGAAAGCTATTAGCTACGGTTAGCTAACGGCCGACATGAGAG	73	568	8	54.79	662	1	0
N/A	GGGACACAATGGACGTAAGAAGATTTTCATATGAACAAGGTGCACTAAGTGGCTATAACGGCCGACATGAGAG	73	568	8	54.79	582	1	0
N/A	GGGACACAATGGACGGCTGAAGTACTATATACGTTGATCGTTGAAGCTATACTATATAACGGCCGACATGAGAG	74	568	8	54.79	585	1	0
N/A	GGGACACAATGGACGTTGCGTTTCAATTTTCGACATAACGGCCGACATGAGAG	53	568	8	54.79	663	1	0

N/A	GGGACACAATGGACGAAGTACAATGAGAACATAGTATGTTTCCAAATGTTGGCGCATAACGGCCGACATGAGAG	74	568	8	54.79	606	1	0
N/A	GGGACACAATGGACGTTACATATAGTTAAATTGAAGGTAATACTGATTATACCAGTAACGGCCGACATGAGAG	73	568	8	54.79	599	1	0
N/A	GGGACACAATGGACGGTCTTATATATTGAAGTTAAATCAGTAACTAACGGCCGACATGAGAG	62	568	8	54.79	567	1	0
N/A	GGGACACAATGGACGCTTATACGCAATTTACTAACTGGTAGACGGTACGTAATAGTAACGGCCGACATGAGAG	73	568	8	54.79	623	1	0
N/A	GGGACACAATGGACGTCAAAAGCGTTGGAAACAACAATAGTCAGTCGATAATACTAACGGCCGACATGAGAG	72	568	8	54.79	629	1	0
N/A	GGGACACAATGGACGAAACCATGCTGAGAAGACCAGGCAATTCTAAACAATAGATAACGGCCGACATGAGAG	72	568	8	54.79	665	1	0
N/A	GGGACACAATGGACGAGATAACTTACGGCTTATGAATTAATTGGTCGTGCAGTATTAACGGCCGACATGAGAG	73	568	8	54.79	572	1	0
N/A	GGGACACAATGGACGATAAGTAGTTAAGAGTGTAGTATTTTGGCCGATTACAAACTAACGGCCGACATGAGAG	73	568	8	54.79	612	1	0
N/A	GGGACACAATGGACGTGACATCGGTCGAATGGTTATGCTAGTAATATAAACAGTGTAACGGCCGACATGAGAG	73	568	8	54.79	640	1	0
N/A	GGGACACAATGGACGTATAGACATAAAAGAGATCATAATTTGGTTTCACAGCACTAACGGCCGACATGAGAG	72	568	8	54.79	708	1	0
N/A	GGGACACAATGGACGCGCGGTAGATAAAGAAGATAGCAATTGTATGCTACAGATTTAACGGCCGACATGAGAG	73	568	8	54.79	718	1	0

Data set to x and y were obtained from VLPs of CHIKV and JEV, respectively, as described in Materials and Methods.

[a] N/E; not eligible, the sequences less than 70 nt in length and less than 0.5 in enrichment score were not analyzed as ineligible sequences. N/A; not analyzed, the sequences were not analyzed.

[b] Read per million (RPM). Sequences with more than 50 RPM were shown.
